# Development of a novel series of thiazole-based compounds with enhanced antiproliferative properties as tubulin polymerization inhibitors

**DOI:** 10.3389/fchem.2026.1814119

**Published:** 2026-04-22

**Authors:** Lamya H. Al-Wahaibi, Ali M. Elshamsy, Taha F. S. Ali, Bahaa G. M. Youssif, Stefan Bräse, Mohamed Abdel-Aziz, Nawal A. El-Koussi

**Affiliations:** 1 Department of Chemistry, College of Sciences, Princess Nourah Bint Abdulrahman University, Riyadh, Saudi Arabia; 2 Pharmceutical Chemistry Department, Faculty of Pharmacy, Deraya University, Minia, Egypt; 3 Medicinal Chemistry Department, Faculty of Pharmacy, Minia University, Minia, Egypt; 4 Department of Pharmaceutical Organic Chemistry, Faculty of Pharmacy, Assiut University, Assiut, Egypt; 5 Institute of Biological and Chemical Systems, IBCS-FMS, Karlsruhe Institute of Technology, Karlsruhe, Germany; 6 Medicinal Chemistry Department, Faculty of Pharmacy, Minia National University, Minia, Egypt; 7 Department of Pharmaceutical Medicinal Chemistry, Faculty of Pharmacy, Assiut University, Assiut, Egypt

**Keywords:** apoptosis, cell cycle, colchicine, NCI, tubulin, Western blot

## Abstract

**Introduction:**

In cancer therapy, inhibiting tubulin polymerization is a key approach for modifying microtubule dynamics required for cell survival and proliferation. Microtubule destabilizing agents (MDAs), also known as tubulin polymerization inhibitors, prevent tubulin heterodimers from forming microtubules, resulting in catastrophic cellular collapse.

**Methods:**

A novel series of thiazole-based compounds **8a-o** was developed to inhibit tubulin polymerization and assess for its antiproliferative efficacy against the NCI 60 cell line. The structures of the newly synthesized compounds were confirmed using ^1^H NMR, ^13^C NMR, and elemental microanalyses. All 15 compounds (**8a-o**) were assessed for antiproliferative action at a single dosage (10 μM) and analyzed against the comprehensive 60-cell panel at five concentrations (0.01, 0.1, 1, 10, and 100 μM).

**Results and Discussion:**

The results from the one-dose and five-dose studies demonstrate that **8b**, **8c**, **8d**, **8m**, and **8o** are the most prominent antiproliferative agents, exhibiting the most favourable low-micromolar GI_50_ values across various cell lines, frequently advancing to low-micromolar TGI, and, in numerous sensitive cell lines, achieving LC_50_ values within the single-digit micromolar range. Compounds **8b**, **8d** and **8m** showed significant anti-tubulin activity, with IC50 values ranging from 3.86 to 7.19 μM, compared to the reference CA-4 (IC50 = 2.40 μM). In the MCF-7 breast cancer cell line, compound **8m** drove a significant accumulation of cells in the G2/M phase, increasing from 13.74% to 45.35%. G2/M arrest is frequently associated with DNA damage or the inhibition of microtubule dynamics, which aligns with Western blot results demonstrating a decrease in tubulin (50 kDa) expression following treatment with **8m**. Apoptotic and necrotic experiments indicate that **8m** stimulates a defined programmed cell death pathway rather than inducing non-specific toxic necrosis. Molecular docking corroborated their binding at the colchicine site, while in silico ADMET profiling indicated a promising drug-like profile for compound **8m**.

## 1 Introduction

The fundamental components of the cytoskeleton are microtubules, composed of α and β-tubulin heterodimers, along with microtubule-associated proteins (Wethekam and Moore, 2026). These proteins exhibit the dynamic characteristics of polymerization and depolymerization (Abdelbaset et al., 2018; Tarantino et al., 2026). They are essential for preserving normal cell shape, mitosis, signal transduction, and material transport, among other biological processes (Abdelbaset et al., 2021; Zhang et al., 2026). Because of the successful studies on microtubules, they have become a primary target for anticancer medicines. Several recognized anticancer drugs, including as paclitaxel, taxanes, and vinca alkaloids, are currently commercially available (Salinas et al., 2025; Zheng et al., 2025), and numerous therapy candidates are in clinical trials (Assunção et al., 2025).

Colchicine binding site inhibitors, a category of microtubule-disrupting agents, exhibited significant anticancer effects, such as destabilizing the tumor cytoskeleton, inhibiting tumor cell division, and promoting the G2/M phase (Aviles et al., 2026; Xu et al., 2025). They demonstrated potential in resolving various challenges associated with taxanes and vinca alkaloids (Reed et al., 2025; Shan et al., 2025). Several potent inhibitors of the colchicine binding site have been identified. among these, compounds such as Fosbretabulin disodium **(CA-4P)** (Yang et al., 2025), **OXI-4503** (Wanniarachchi et al., 2025), **ABT-751** (Mahgoub et al., 2024), and **VERU-111** (Mamun et al., 2026), Figure 1, have received approval for clinical trials aimed at treating human cancers. Nonetheless, the advancement of novel tubulin-targeted anticancer therapies remains motivated by systemic toxicities and multiorgan dysfunction observed in clinical studies involving colchicine binding site inhibitors (Lafanechère, 2022; Sun et al., 2021).

**FIGURE 1:**
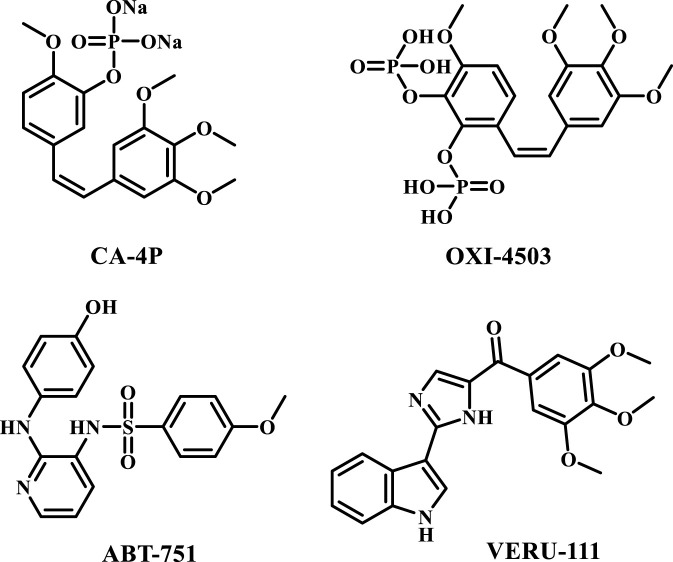
Structures of compounds CA-4P, OXI-4503, ABT-751, and VERU-111.

**FIGURE 2:**
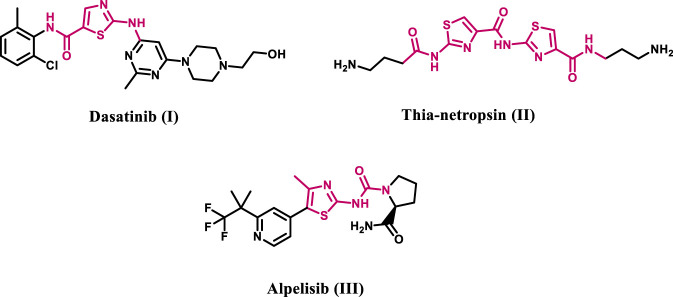
Structures of 2-aminothiazole-based compounds I-III.

Heterocyclic compounds are important because they are widely used in chemistry, industry, and medical research, serving as essential building blocks for many useful materials. Their particular structural architecture enables precise changes in biological activity and chemical stability, establishing them as critical to modern drug development projects (Joule and Mills, 2024; Liolios et al., 2020; He et al., 2024). Thiazole-based derivatives constitute a significant family of heterocyclic compounds recognized for their anticancer activities due to their strong affinity for many biological targets associated with cancer growth (Al-Salmi et al., 2023; Kaur and Jaitak, 2022), such as Tiazofurin (Tricot et al., 1990) and Bleomycin (Hecht, 2000). Initiatives have been undertaken to enhance the antitumor efficacy of the 2-aminothiazole core in cancer treatments, including dasatinib (**I**) (Saha et al., 2016), thia-netropsin (**II**) (Plouvier et al., 1989), and alpelisib (**III**) (Juric et al., 2019), as depicted in Figure 2, which obtained medical approval in 2019.

**FIGURE 3:**
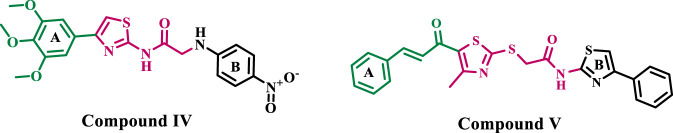
Structures of some representative thiazole-based antitubulin compounds IV and V.

Furthermore, studies have demonstrated that a number of 2-amino-4-phenylthiazole derivatives impede cellular division, tubulin polymerization, and microtubule assembly (Alizadeh and Hashemi, 2021; Farouk Elsadek et al., 2021). A novel class of thiazole-derived tubulin inhibitors has been designed and evaluated (El-Abd et al., 2022). Compound **IV**, illustrated in Figure 3, was the most efficient tubulin inhibitor. In this series, the authors substitute the cis-alkene linker in CA-4 with a thiazole-2-acetamide molecule, serving as a stiff heterocyclic linker, while retaining the trimethoxy phenyl moiety (ring A) present in CA-4. The amidic NH and sulfur atoms of the thiazole ring formed hydrogen bonds with the residue ThrB353, essential for receptor site engagement, as indicated by the docking analysis of compound IV into the colchicine binding site. Moreover, amidic NH demonstrated the essential function of this group in activity by forming a hydrogen bond with the GlnB247 amino acid residue.

We recently (Al-Wahaibi et al., 2025) disclosed the developing of novel thiazole-based molecules that inhibit tubulin polymerization. The most effective antitubulin derivative, compound **V** (Figure 3), exhibited an IC_50_ value of 2.69 µM, surpassing combretastatin A-4, which had an IC_50_ value of 8.33 µM. At a concentration of 50 μM, compound **V** preserved approximately 85% cell viability without adversely affecting normal cells. Compound **V** was established to downregulate the anti-apoptotic protein Bcl-2 while activating caspases 3, 9, and Bax. Molecular docking investigations revealed superior binding affinities for **V** at the colchicine binding site of tubulin, where it established significant hydrogen bonds and hydrophobic interactions that enhance its activity.

**FIGURE 4:**
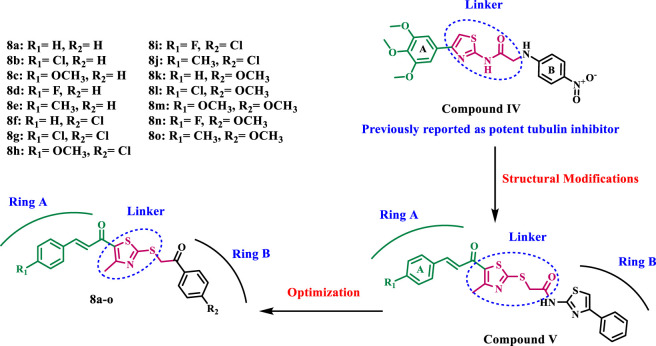
Structures of previously reported compounds IV, V, and new compounds 8a-o.

Motivated by the promising antitubulin properties of compounds **IV** and **V**, we continue our efforts to develop targeted anticancer drugs as tubulin inhibitors (Abdelbaset et al., 2018; Abdelbaset et al., 2021; Al-Wahaibi et al., 2025; Mahmoud et al., 2025) by designing and synthesizing a new series of thiazole-based inhibitors **8a-o**, as shown in Figure 4. The newly synthesized compounds are made up of three main components: ring A, a chalcone moiety that has been found to improve binding to the colchicine binding site through a sequence of hydrophobic interactions, as demonstrated by compound **V**. The second component is the linker, namely, the thiazole-2-acetamide moiety. The final component is ring B, a phenyl ketone moiety designed to increase activity by forming both hydrophobic and hydrophilic attachments.

Novel compounds **8a-o** were submitted to the National Cancer Institute (NCI) for evaluation of their antiproliferative properties, with all compounds selected for both single-dose and five-dose assays. The most efficient compounds were further evaluated for *in vitro* anti-tubulin activity. The most potent derivative was evaluated using Western blot assay, cell cycle analysis, and apoptosis induction. Ultimately, *in silico* analyses were performed for some selected compounds.

## 2 Materials and methods

### 2.1 Chemistry

General Details: Refer to (Supplementary Appendix A).

3-Chloroacetylacetone (Abo-Ashour et al., 2018), 1-(2-mercapto-4-methylthiazol-5-yl)ethan-1-one and (**5a-e**) (Hashem et al., 2024) and phenacyl bromides **(7a-c)** (Gangwar and Mandal, 2026) were prepared according to reported procedures.

#### 2.1.1 Synthesis of the target compounds 8a-o

A solution containing the appropriate chalcone derivatives **5a–e** (1 mmol) and the corresponding phenacyl bromides **7a–c** (1 mmol) was treated with anhydrous sodium carbonate (1.5 mmol) and sodium iodide (2 mmol) in acetone and stirred at room temperature for 6 h. Upon completion of the reaction, the solvent was removed *in vacuo*, and the resulting residue was washed successively with 10% aqueous sodium thiosulfate followed by distilled water, then purified by recrystallization from acetonitrile.

##### 2.1.1.1 (*E*)-1-{4-Methyl-2-[(2-oxo-2-phenylethyl)thio]thiazol-5-yl}-3-phenylprop-2-en-1-one (8a)

Yellow powder; 0.325 g, 85.6% yield; mp 134 °C–137 °C; ^1^H NMR (500 MHz, DMSO-*d*_6_) δ 8.02 (dd, *J* = 8.2, 1.0 Hz, 2H, Ar-H), 7.74 (dd, *J* = 6.5, 2.9 Hz, 2H, Ar-H), 7.65 (t, *J* = 7.4 Hz, 1H, Ar-H), 7.62 (d, *J* = 15.5 Hz, 1H, *CH* = CH), 7.53 (t, *J* = 7.7 Hz, 2H, Ar-H), 7.41–7.38 (m, 3H, Ar-H), 7.31 (d, *J* = 15.5 Hz, 1H, CH = *CH*), 5.07 (s, 2H, CH_2_), 2.57 (s, 3H, CH_3_); ^13^C NMR (125 MHz, DMSO-d_6_) δ 193.30, 181.87, 168.98, 158.46, 144.15, 135.82, 134.67, 134.41, 132.37, 131.39, 129.52, 129.39, 129.33, 129.01, 124.84, 42.16, 18.79; Anal. Calcd. For C_21_H_17_NO_2_S_2_: C, 66.47%; H, 4.52%; N, 3.69%. Found: C, 66.63%; H, 4.68%; N, 3.81%.

##### 2.1.1.2 (*E*)-3-(4-Chlorophenyl)-1-{4-methyl-2-[(2-oxo-2-phenylethyl)thio]thiazol-5-yl}prop-2-en-1-one (8b)

Yellow powder; 0.331 g, 80.0% yield; mp 144 °C–146 °C; ^1^H NMR (500 MHz, DMSO-*d*_6_) δ 8.04 (d, *J* = 7.7 Hz, 2H, Ar-H), 7.81 (d, *J* = 8.4 Hz, 2H, Ar-H), 7.68 (t, *J* = 7.4 Hz, 1H, Ar-H), 7.63 (d, *J* = 15.5 Hz, 1H, *CH* = CH), 7.56 (t, *J* = 7.7 Hz, 2H, Ar-H), 7.48 (d, *J* = 8.4 Hz, 2H, Ar-H), 7.35 (d, *J* = 15.5 Hz, 1H, CH = *CH*), 5.09 (s, 2H, CH_2_), 2.59 (s, 3H, CH_3_); ^13^C NMR (125 MHz, DMSO-*d*_6_) δ 193.29, 181.78, 169.16, 158.63, 142.69, 135.88, 135.80, 134.44, 133.64, 132.27, 131.04, 129.56, 129.41, 129.00, 125.54, 42.13, 18.79; Anal. Calcd. For C_21_H_16_ClNO_2_S_2_: C, 60.94%; H, 3.90%; N, 3.38%. Found: C, 61.09%; H, 4.12%; N, 3.45%.

##### 2.1.1.3 (*E*)-3-(4-Methoxyphenyl)-1-{4-methyl-2-[(2-oxo-2-phenylethyl)thio]thiazol-5-yl}prop-2-en-1-one (8c)

Yellow crystals; 0.362 g, 88.4% yield; mp 158 °C–160 °C; ^1^H NMR (500 MHz, DMSO-*d*_6_) δ 8.04 (d, *J* = 8.0 Hz, 2H, Ar-H), 7.73 (d, *J* = 8.4 Hz, 2H, Ar-H), 7.70–7.65 (m, 1H, Ar-H), 7.61 (d, *J* = 15.4 Hz, 1H, *CH* = CH), 7.56 (t, *J* = 7.4 Hz, 2H, Ar-H), 7.18 (d, *J* = 15.4 Hz, 1H, CH = *CH*), 6.97 (d, *J* = 8.1 Hz, 2H, Ar-H), 5.08 (s, 2H, CH_2_), 3.78 (s, 3H, OCH_3_), 2.58 (s, 3H, CH_3_); ^13^C NMR (125 MHz, DMSO-*d*_6_) δ 193.34, 181.77, 168.49, 162.12, 158.01, 144.26, 135.83, 134.41, 131.33, 129.40, 129.01, 127.28, 122.28, 115.04, 99.98, 55.93, 42.12, 18.73; Anal. Calcd. For C_22_H_19_NO_3_S_2_: C, 64.53%; H, 4.68%; N, 3.42%. Found: C, 64.37%; H, 4.89%; N, 3.68%.

##### 2.1.1.4 (*E*)-3-(4-Fluorophenyl)-1-{4-methyl-2-[(2-oxo-2-phenylethyl)thio]thiazol-5-yl}prop-2-en-1-one (8d)

Yellow powder; 0.358 g, 90.1% yield; mp 139 °C–142 °C; ^1^H NMR (500 MHz, DMSO-*d*_6_) δ 8.07–8.02 (m, 2H, Ar-H), 7.85 (dd, *J* = 8.7, 5.6 Hz, 2H, Ar-H), 7.68 (t, *J* = 6.9 Hz, 1H, Ar-H), 7.64 (d, *J* = 15.5 Hz, 1H, *CH* = CH), 7.56 (t, *J* = 7.7 Hz, 2H, Ar-H), 7.33–7.22 (m, 3H, Ar-H and CH = *CH*), 5.09 (s, 2H, CH_2_), 2.59 (s, 3H, CH_3_); ^13^C NMR (125 MHz, DMSO-*d*_6_) δ 193.29, 181.83, 169.01, 158.47, 142.97, 135.81, 134.43, 132.34, 131.74, 131.38, 129.40, 129.01, 124.73, 116.65, 116.47, 42.14, 18.79; Anal. Calcd. For C_21_H_16_FNO_2_S_2_: C, 63.46%; H, 4.06%; N, 3.52%. Found: C, 63.72%; H, 4.13%; N, 3.64%.

##### 2.1.1.5 (*E*)-1-{4-Methyl-2-[(2-oxo-2-phenylethyl)thio]thiazol-5-yl}-3-(*p*-tolyl)prop-2-en-1-one (8e)

Yellow powder; 0.330 g, 83.9% yield; mp 133 °C–135 °C; ^1^H NMR (500 MHz, DMSO-*d*_6_) δ 8.04 (d, *J* = 8.0 Hz, 2H, Ar-H), 7.69 (d, *J* = 7.7 Hz, 1H, Ar-H), 7.65 (d, *J* = 8.1 Hz, 2H, Ar-H), 7.61 (d, *J* = 15.5 Hz, 1H, *CH* = CH), 7.56 (t, *J* = 7.2 Hz, 2H, Ar-H), 7.31–7.21 (m, 3H, Ar-H and CH = *CH*), 5.09 (s, 2H, CH_2_), 2.58 (s, 3H, CH_3_), 2.31 (s, 3H, CH_3_); ^13^C NMR (125 MHz, DMSO-*d*_6_) δ 193.33, 181.86, 168.77, 158.28, 144.28, 141.58, 135.84, 134.41, 132.43, 131.96, 130.21, 130.16, 129.39, 129.01, 123.79, 42.12, 21.64, 18.75; Anal. Calcd. For C_22_H_19_NO_2_S_2_: C, 67.15%; H, 4.87%; N, 3.56%. Found: C, 67.41%; H, 5.03%; N, 3.79%.

##### 2.1.1.6 (*E*)-1-{2-[(2-(4-Chlorophenyl)-2-oxoethyl)thio]-4-methylthiazol-5-yl}-3-phenylprop-2-en-1-one (8f)

Yellow crystals; 0.357 g, 86.2% yield; mp 150 °C–152 °C; ^1^H NMR (500 MHz, DMSO-*d*_6_) δ 8.05 (d, *J* = 8.2 Hz, 2H, Ar-H), 7.80–7.73 (m, 2H, Ar-H), 7.67–7.61 (m, 3H, Ar-H and *CH* = CH), 7.46–7.40 (m, 3H, Ar-H), 7.32 (d, *J* = 15.5 Hz, 1H, CH = *CH*), 5.07 (s, 2H, CH_2_), 2.58 (s, 3H, CH_3_); ^13^C NMR (125 MHz, DMSO-*d*_6_) δ 192.50, 181.91, 168.79, 158.42, 144.18, 139.35, 134.67, 134.54, 132.44, 131.41, 130.93, 129.57, 129.52, 129.34, 124.83, 42.00, 18.77; Anal. Calcd. For C_21_H_16_ClNO_2_S_2_: C, 60.94%; H, 3.90%; N, 3.38%. Found: C, 61.21%; H, 4.12%; N, 3.56%.

##### 2.1.1.7 (*E*)-3-(4-Chlorophenyl)-1-{2-[(2-(4-chlorophenyl)-2-oxoethyl)thio]-4-methylthiazol-5-yl}prop-2-en-1-one (8g)

Brown powder; 0.414 g, 92.3% yield; mp 136 °C–138 °C; ^1^H NMR (500 MHz, DMSO-*d*_6_) δ 8.06 (d, *J* = 6.7 Hz, 2H, Ar-H), 7.81 (d, *J* = 6.9 Hz, 2H, Ar-H), 7.66–7.59 (m, 3H, Ar-H and *CH* = CH), 7.48 (d, *J* = 6.7 Hz, 2H, Ar-H), 7.34 (d, *J* = 16.7 Hz, 1H, CH = *CH*), 5.08 (s, 2H, CH_2_), 2.58 (s, 3H, CH_3_); ^13^C NMR (125 MHz, DMSO-*d*_6_) δ 192.47, 181.79, 168.95, 158.57, 142.71, 139.35, 135.89, 134.54, 133.65, 132.36, 131.06, 130.93, 129.56, 129.52, 125.54, 42.00, 18.78; Anal. Calcd. For C_21_H_15_Cl_2_NO_2_S_2_: C, 56.25%; H, 3.37%; N, 3.12%. Found: C, 56.43%; H, 3.49%; N, 3.30%.

##### 2.1.1.8 (*E*)-1-{2-[(2-(4-Chlorophenyl)-2-oxoethyl)thio]-4-methylthiazol-5-yl}-3-(4-methoxyphenyl)prop-2-en-1-one (8h)

Yellow powder; 0.388 g, 87.4% yield; mp 147 °C–149 °C; ^1^H NMR (500 MHz, DMSO-*d*_6_) δ 8.05 (d, *J* = 8.3 Hz, 2H, Ar-H), 7.73 (d, *J* = 8.4 Hz, 2H, Ar-H), 7.67–7.58 (m, 3H, Ar-H and *CH* = CH), 7.18 (d, *J* = 15.4 Hz, 1H, CH = *CH*), 6.97 (d, *J* = 8.4 Hz, 2H, Ar-H), 5.06 (s, 2H, CH_2_), 3.78 (s, 3H, OCH_3_), 2.57 (s, 3H, CH_3_); ^13^C NMR (125 MHz, DMSO-*d*_6_) δ 192.52, 181.77, 168.26, 162.13, 157.97, 144.28, 139.33, 134.56, 132.62, 131.33, 130.93, 129.51, 127.28, 122.26, 115.04, 55.97, 41.96, 18.72; Anal. Calcd. For C_22_H_18_ClNO_3_S_2_: C, 59.52%; H, 4.09%; N, 3.16%. Found: C, 59.68%; H, 4.17%; N, 3.28%.

##### 2.1.1.9 (*E*)-1-{2-[(2-(4-Chlorophenyl)-2-oxoethyl)thio]-4-methylthiazol-5-yl}-3-(4-fluorophenyl)prop-2-en-1-one (8i)

Yellow crystals; 0.365 g, 84.5% yield; mp 155 °C–158 °C; ^1^H NMR (500 MHz, DMSO-*d*_6_) δ 8.05 (d, *J* = 8.5 Hz, 2H, Ar-H), 7.89–7.82 (m, 2H, Ar-H), 7.68–7.60 (m, 3H, Ar-H and *CH* = CH), 7.32–7.23 (m, 3H, Ar-H and CH = *CH*), 5.07 (s, 2H, CH_2_), 2.58 (s, 3H, CH_3_); ^13^C NMR (125 MHz, DMSO-*d*_6_) δ 192.49, 181.85, 168.76, 158.42, 142.98, 139.34, 134.56, 132.41, 131.73, 131.36, 130.92, 129.51, 124.73, 116.65, 116.48, 41.97, 18.74; Anal. Calcd. For C_21_H_15_ClFNO_2_S_2_: C, 58.40%; H, 3.50%; N, 3.24%. Found: C, 58.61%; H, 3.62%; N, 3.43%.

##### 2.1.1.10 (*E*)-1-{2-[(2-(4-Chlorophenyl)-2-oxoethyl)thio]-4-methylthiazol-5-yl}-3-(*p*-tolyl)prop-2-en-1-one (8j)

Yellow powder; 0.389 g, 90.9% yield; mp 145 °C–147 °C; ^1^H NMR (500 MHz, DMSO-*d*_6_) δ 8.05 (d, *J* = 8.6 Hz, 2H, Ar-H), 7.66–7.62 (m, 4H, Ar-H), 7.61 (d, *J* = 11.9 Hz, 1H, *CH* = CH), 7.28–7.22 (m, 3H, Ar-H and CH = *CH*), 5.07 (s, 2H, CH_2_), 2.58 (s, 3H, CH_3_), 2.31 (s, 3H, CH_3_); ^13^C NMR (125 MHz, DMSO-*d*_6_) δ 192.52, 181.87, 168.57, 158.25, 144.30, 141.60, 139.34, 134.55, 132.49, 131.95, 130.93, 130.16, 129.51, 129.40, 123.76, 41.99, 21.65, 18.75; Anal. Calcd. For C_22_H_18_ClNO_2_S_2_: C, 61.74%; H, 4.24%; N, 3.27%. Found: C, 62.03%; H, 4.39%; N, 3.52%.

##### 2.1.1.11 (*E*)-1-{2-[(2-(4-Methoxyphenyl)-2-oxoethyl)thio]-4-methylthiazol-5-yl}-3-phenylprop-2-en-1-one (8k)

Yellow powder; 0.332 g, 81.1% yield; mp 151 °C–153 °C; ^1^H NMR (500 MHz, DMSO-*d*_6_) δ 8.03 (d, *J* = 8.9 Hz, 2H, Ar-H), 7.76 (dd, *J* = 6.7, 2.8 Hz, 2H, Ar-H), 7.64 (d, *J* = 15.5 Hz, 1H, *CH* = CH), 7.43 (dd, *J* = 4.9, 1.7 Hz, 3H, Ar-H), 7.33 (d, *J* = 15.5 Hz, 1H, CH = *CH*), 7.07 (d, *J* = 8.9 Hz, 2H, Ar-H), 5.03 (s, 2H, CH_2_), 3.84 (s, 3H, OCH_3_), 2.61 (s, 3H, CH_3_); ^13^C NMR (125 MHz, DMSO-*d*_6_) δ 191.52, 181.90, 169.27, 164.24, 158.50, 144.14, 134.68, 132.32, 131.47, 131.40, 129.54, 129.33, 128.62, 124.88, 114.63, 56.22, 41.95, 18.80; Anal. Calcd. For C_22_H_19_NO_3_S_2_: C, 64.53%; H, 4.68%; N, 3.42%. Found: C, 64.81%; H, 4.57%; N, 3.67%.

##### 2.1.1.12 (*E*)-3-(4-Chlorophenyl)-1-{2-[(2-(4-methoxyphenyl)-2-oxoethyl)thio]-4-methylthiazol-5-yl}prop-2-en-1-one (8l)

Yellow crystals; 0.398 g, 89.6% yield; mp 137 °C–140 °C; ^1^H NMR (500 MHz, DMSO-*d*_6_) δ 8.02 (d, *J* = 8.5 Hz, 2H, Ar-H), 7.80 (d, *J* = 7.6 Hz, 2H, Ar-H), 7.62 (d, *J* = 14.7 Hz, 1H, *CH* = CH), 7.48 (d, *J* = 7.2 Hz, 2H, Ar-H), 7.34 (d, *J* = 15.7 Hz, 1H, CH = *CH*), 7.06 (d, *J* = 7.7 Hz, 2H, Ar-H), 5.03 (s, 2H, CH_2_), 3.83 (s, 3H, OCH_3_), 2.60 (s, 3H, CH_3_); ^13^C NMR (125 MHz, DMSO-*d*_6_) δ 191.48, 181.75, 169.42, 164.24, 158.67, 142.66, 135.87, 133.66, 132.22, 131.46, 131.04, 129.55, 128.60, 125.56, 114.62, 56.18, 41.97, 18.81; Anal. Calcd. For C_22_H_18_ClNO_3_S_2_: C, 59.52%; H, 4.09%; N, 3.16%. Found: C, 59.73%; H, 4.23%; N, 3.29%.

##### 2.1.1.13 (*E*)-3-(4-Methoxyphenyl)-1-{2-[(2-(4-methoxyphenyl)-2-oxoethyl)thio]-4-methylthiazol-5-yl}prop-2-en-1-one (8m)

Brown powder; 0.362 g, 82.4% yield; mp 141 °C–143 °C; ^1^H NMR (500 MHz, DMSO-*d*_6_) δ 8.02 (d, *J* = 8.8 Hz, 2H, Ar-H), 7.73 (d, *J* = 8.8 Hz, 2H, Ar-H), 7.61 (d, *J* = 15.4 Hz, 1H, *CH* = CH), 7.18 (d, *J* = 15.4 Hz, 1H, CH = *CH*), 7.06 (d, *J* = 8.8 Hz, 2H, Ar-H), 6.97 (d, *J* = 8.8 Hz, 2H, Ar-H), 5.02 (s, 2H, CH_2_), 3.84 (s, 3H, OCH_3_), 3.78 (s, 3H, OCH_3_), 2.59 (s, 3H, CH_3_); ^13^C NMR (125 MHz, DMSO-*d*_6_) δ 191.56, 181.78, 168.76, 164.24, 162.12, 158.08, 144.24, 132.47, 131.46, 131.31, 128.61, 127.27, 122.29, 115.05, 114.63, 56.17, 55.91, 41.87, 18.73; Anal. Calcd. For C_23_H_21_NO_4_S_2_: C, 62.85%; H, 4.82%; N, 3.19%. Found: C, 63.02%; H, 4.91%; N, 3.41%.

##### 2.1.1.14 (*E*)-3-(4-Fluorophenyl)-1-{2-[(2-(4-methoxyphenyl)-2-oxoethyl)thio]-4-methylthiazol-5-yl}prop-2-en-1-one (8n)

Yellow powder; 84.9% yield; mp 157 °C–159 °C; ^1^H NMR (500 MHz, DMSO-*d*_6_) δ 8.02 (d, *J* = 8.8 Hz, 2H, Ar-H), 7.85 (dd, *J* = 8.2, 5.9 Hz, 2H, Ar-H), 7.64 (d, *J* = 15.5 Hz, 1H, *CH* = CH), 7.30–7.24 (m, 3H, Ar-H and CH = *CH*), 7.07 (d, *J* = 8.8 Hz, 2H, Ar-H), 5.03 (s, 2H, CH_2_), 3.84 (s, 3H, OCH_3_), 2.60 (s, 3H, CH_3_); ^13^C NMR (125 MHz, DMSO-*d*_6_) δ 191.48, 181.79, 169.27, 164.23, 158.51, 142.93, 132.27, 131.78, 131.73, 131.46, 128.59, 124.72, 116.65, 116.47, 114.62, 56.22, 41.99, 18.81; Anal. Calcd. For C_22_H_18_FNO_3_S_2_: C, 61.81%; H, 4.24%; N, 3.28%. Found: C, 62.09%; H, 4.45%; N, 3.40%.

##### 2.1.1.15 (*E*)-1-{2-[(2-(4-Methoxyphenyl)-2-oxoethyl)thio]-4-methylthiazol-5-yl}-3-(*p*-tolyl)prop-2-en-1-one (8o)

Yellow powder; 0.381 g, 90.0% yield; mp 148 °C–150 °C; ^1^H NMR (500 MHz, DMSO-*d*_6_) δ 8.02 (d, *J* = 8.8 Hz, 2H, Ar-H), 7.65 (d, *J* = 8.0 Hz, 2H, Ar-H), 7.61 (d, *J* = 15.5 Hz, 1H, *CH* = CH), 7.26 (d, *J* = 15.5 Hz, 1H, CH = *CH*), 7.23 (d, *J* = 7.9 Hz, 2H, Ar-H), 7.06 (d, *J* = 8.9 Hz, 2H, Ar-H), 5.02 (s, 2H, CH_2_), 3.84 (s, 3H, OCH_3_), 2.60 (s, 3H, CH_3_), 2.31 (s, 3H, CH_3_); ^13^C NMR (125 MHz, DMSO-*d*_6_) δ 191.53, 181.84, 169.06, 164.24, 158.35, 144.26, 141.58, 132.36, 131.96, 131.47, 130.16, 129.39, 128.61, 123.78, 114.62, 56.19, 41.94, 21.65, 18.79; Anal. Calcd. For C_23_H_21_NO_3_S_2_: C, 65.22%; H, 5.00%; N, 3.31%. Found: C, 65.43%; H, 5.17%; N, 3.57%.

### 2.2 Biology

#### 2.2.1 Assessment of *in vitro* antiproliferative efficacy of 8a-o by NCI

The NCI anticancer screening process has been thoroughly detailed (http://www.dtp.nci.nih.gov). The anticancer assay was conducted on roughly 60 human tumour cell lines generated from nine neoplastic disorders, following the procedure of the Drug Evaluation Branch, National Cancer Institute, Bethesda, United States. Comprehensive methodologies are delineated in the supplemental materials associated with this publication.

#### 2.2.2 Cell viability assay

The effects of derivatives **8b**, **8c**, **8d**, **8m**, and **8o** on cell viability were evaluated using the human mammary gland epithelial normal cell line (MCF-10A). The MTT assay was employed to ascertain the viability of the compounds under investigation after a 4-day incubation with MCF-10A cells (Alshammari et al., 2022). For further details, check Supplementary Appendix A.

#### 2.2.3 Assay for tubulin polymerization

The impact of substances on tubulin polymerization was examined using the Tubulin Polymerization Assay Kit (Cytoskeleton Inc., Denver, CO, United States) according to the supplier’s instructions, with details summarized in Supplementary Appendix A; (Al-Wahaibi et al., 2025).

#### 2.2.4 Western blot assay

The Western Blot assay for Tubulin analysis in MCF7 cells of compound **8m** was performed using the BCA Protein Assay Kit (Thermo Fisher Scientific, Cat# 23225) (Ubukata et al., 2026). Refer to Supplementary Appendix A for more details.

#### 2.2.5 Cell cycle analysis and apoptosis

The MCF-7 cell line was utilized to analyse the cell cycle and identify apoptosis. Assay was carried out as previously reported (El-Sherief et al., 2018; Supplementary Appendix A).

## 3 Results and discussion

### 3.1 Chemistry

**SCHEME 1:**
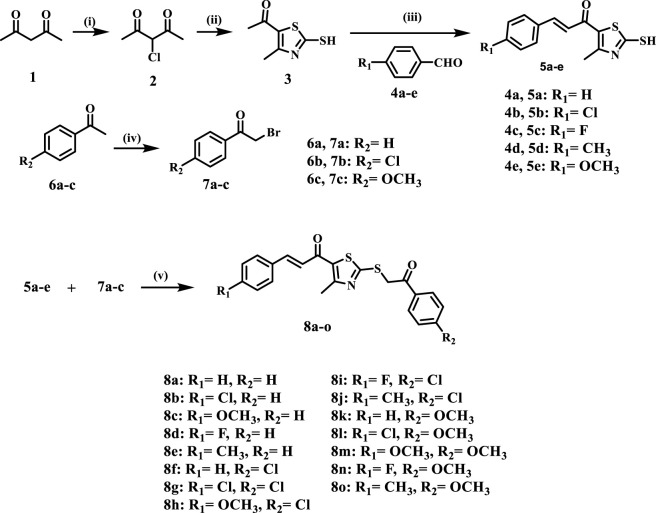
Synthesis of the target compounds 8a-o.

The synthetic pathway for the target **8a–o** is depicted in Scheme 1. Initially, pentane-2,4-dione (**1**) underwent selective α-chlorination with sulfuryl chloride in toluene, yielding the intermediate 3-chloro-pentane-2,4-dione (**2**) (Al-Wahaibi et al., 2025). The activated 1,3-dicarbonyl compound was subsequently transformed into the key 2-mercaptothiazole (**3**) through treatment with ammonia and carbon disulphide in ethanol at ambient temperature. The chalcone series (**5a–e**) was synthesised using Claisen–Schmidt condensation of compound **3** with aromatic aldehydes (**4a-d**) under basic conditions. In parallel, substituted phenacyl bromides (**7a-c**) were synthesised from acetophenone derivatives (**6a-c**) using N-bromosuccinimide (NBS) and *p*-toluenesulfonic acid monohydrate (PTSA) in refluxing acetonitrile. The final compounds **8a–o** were synthesised via nucleophilic substitution between chalcones (**5a–e**) and bromomethyl ketones (**7a–c**) using sodium carbonate and catalytic sodium iodide in acetone at ambient temperature.

Reagents and Conditions: (i) SO_2_Cl_2_, toluene, 0 °C, 12 h, 67%; (ii) NH_3_, CS_2_, EtOH, RT, 24 h 38%; (iii) appropriate aromatic aldehyde, 60% NaOH, EtOH, 0 °C, 18 h, 78%–89%; (iv) NBS, PTSA. H_2_O, acetonitrile, reflux, 10 h, 69%–83%; (v) Na_2_CO_3_, NaI, acetone, RT, 6 h, 80%–92%.

All final compounds **8a–o** were characterized by ^1^H NMR, ^13^C NMR, and elemental (CHN) analyses, confirming their structures. The ^1^H NMR spectra of the synthesized compounds showed a common set of characteristic signals with variations arising from the different aromatic substituents. The aromatic proton signals were observed in the region δ 6.97–8.07 ppm, appearing as overlapping signals depending on the substitution pattern of the phenyl rings. The α,β-unsaturated system was evident from two olefinic protons appearing as doublets at approximately δ 7.18–7.35 ppm and δ 7.61–7.64 ppm, each integrating for one proton and displaying large coupling constants (J ≈ 15.4–15.7 Hz), confirming the trans-configured double bond. A singlet integrating for two protons was consistently observed at δ 5.02–5.09 ppm, corresponding to the methylene group. The methyl group attached to the thiazole ring resonated as a singlet at δ 2.57–2.61 ppm in all derivatives. Compounds bearing methoxy substituents exhibited singlets at δ 3.78–3.84 ppm, while derivatives containing an additional methyl substituent on the aromatic ring showed a singlet at δ ∼2.31 ppm.

The ^13^C NMR spectra further supported the proposed structures. All compounds exhibited two characteristic carbonyl carbon resonances in the regions δ 191–193 ppm and δ 181–182 ppm, corresponding to the chalcone and phenacyl carbonyl groups. The methylene carbon consistently appeared at δ ∼41.9–42.1 ppm, and the thiazole methyl carbon was observed at δ ∼18.7–18.8 ppm. Methoxy-substituted derivatives displayed additional resonances at δ 55–56 ppm, whereas methyl-substituted compounds showed an additional signal near δ 21–22 ppm.

As a representative example, compound **8o** displayed all expected signals in both ^1^H and ^13^C NMR spectra. In its ^1^H NMR spectrum, the two olefinic protons appeared as doublets at δ 7.61 and 7.26 ppm with a coupling constant of J = 15.5 Hz. The methylene protons appeared as a singlet at δ 5.02 ppm, while singlets at δ 3.84, 2.60, and 2.31 ppm corresponded to the methoxy group, the thiazole methyl group, and the additional methyl substituent, respectively. The aromatic protons were observed between δ 7.06–8.02 ppm. The ^13^C NMR spectrum of **8o** showed two carbonyl signals at δ 191.53 and 181.84 ppm. Additional aromatic and olefinic carbons were observed across the expected chemical shift range, while the methoxy carbon resonated at δ 56.19 ppm, the methylene carbon at δ 41.94 ppm, and the methyl carbons at δ 21.65 and 18.79 ppm, in agreement with the assigned structure.

### 3.2 Biological evaluation

#### 3.2.1 Screening for anticancer activity

##### 3.2.1.1 *In Vitro* one-dose growth inhibition screening using NCI-60 cell line panel

The National Cancer Institute’s (NCI) Developmental Therapeutics Program accepted all 15 compounds **8a-o** and authorized them for use in the NCI-60 human cancer cell line screen. This screen examines small compounds in 60 human cancer cell lines representing nine major tumour types. Prior to advancing to multi-dose testing, each compound is assessed at a single concentration (10^−5^ M; 10 μM) over the complete panel during the preliminary one-dose phase. Results are shown here as Growth Inhibition (%), with 0 representing no effect, negative numbers representing increased growth, 100 representing total growth inhibition, and >100 representing cytotoxicity. Tables 1, 2 depict the full single-dose growth inhibition data for each compound on the NCI-60 panel.

**TABLE 1:** Growth inhibition percentages (GI%) of compounds **8a-h** at a single concentration (10 μM) across the NCI-60 human cancer cell line panel.

Cancer subpanel	Cancer cell line	Compound							
		8a	8b	8c	8d	8e	8f	8g	8h
Leukemia	CCRF-CEM	185.86	197.68	198.32	196.87	191.08	69.53	182.18	96.95
	HL-60(TB)	196.01	198.87	199.13	196.02	197.76	132	198.86	199.41
	K-562	71.43	111.88	177.88	107.89	6.26	108.15	80.27	109.57
	MOLT-4	198.91	199.17	199.46	198.66	195.1	85.93	167.35	161.01
	RPMI-8226	198.65	199	199.35	198.28	198.24	199.33	198.75	197.73
	SR	ND	ND	ND	ND	ND	146.18	ND	190.15
NSCLC	A549/ATCC	67.11	83.11	113.42	107.08	60.9	15.42	55.69	8.97
	EKVX	132.55	182.31	144.55	126.14	38.72	5.53	97.61	−6.77
	HOP-62	115.64	138.74	188.66	191.98	146.1	7.77	150.5	11.18
	HOP-92	88.02	123.05	101.45	88.19	77.04	35.32	101.66	−5.7
	NCI-H226	130.29	138.72	98.19	74.65	101.41	18.78	116.02	17.53
	NCI-H23	168.84	171.56	183.37	159.65	93.93	31.07	127.57	32.34
	NCI-H322M	69.89	74.88	115.05	111.28	56.25	−6.71	63.81	−10.21
	NCI-H460	103.1	192.78	121.47	134.14	129.7	14.12	86.94	8.98
	NCI-H522	90.61	182.93	194.58	176.19	143.23	12.03	115.83	15.52
Colon cancer	COLO 205	65.88	104.21	190.91	122.74	48.49	2.74	67.42	−9.23
	HCC-2998	194.28	192.43	198.08	198.06	162.54	116.64	193.71	72.64
	HCT-116	194.68	149.85	196.45	194.62	172.8	160.58	148.68	182.03
	HCT-15	173.14	187.87	191.66	182.99	175.24	71.33	190.68	90.1
	HT29	119.44	157.92	150.71	163.35	159.92	45.61	134.64	32.51
	KM12	135.67	149.3	191.98	188.23	137.93	128.47	165.25	91.63
	SW-620	186.9	186.58	191.38	191.82	170.68	76.31	150.08	86.52
CNS cancer	SF-268	65.2	129.02	149.9	104.97	116.96	52.17	97.62	51.04
	SF-295	67.78	137.68	140.81	103.51	49.17	14.29	61.85	−2.1
	SF-539	180.34	198.26	197.84	197.31	188.65	66.97	191.01	64.4
	SNB-19	92.84	135.3	−4.17	125.74	100.43	8.29	87.11	8.25
	SNB-75	108.87	103.79	123.13	113.82	118.88	−14.98	109.66	−16.46
	U251	170.84	179.36	185.45	184.05	147.01	74.95	173.08	69.8
Melanoma	LOX IMVI	190.24	183.92	198.17	196.15	168.24	124.77	154.38	137.4
	MALME-3M	128.51	164.43	190.21	162.05	71.34	23.79	109.6	−17.53
	M14	148.61	172.47	193.67	186.34	142.37	113.26	115.46	97.23
	MDA-MB-435	114.57	107.86	194.51	181.81	120.98	130.5	109.87	90.42
	SK-MEL-2	177.76	193	196.95	188.28	168.32	24.58	183.87	54.75
	SK-MEL-28	129.71	152.51	181.75	118.18	103.8	8.23	135.72	8.31
	SK-MEL-5	150.48	198.61	195.15	194.32	126.33	53.84	157.21	37.43
	UACC-257	176.79	179.04	190.73	173.92	133.49	79.96	170.9	63.94
	UACC-62	128.06	169.26	179.29	166.63	158.25	60.48	115.1	61.06
Ovarian cancer	IGROV1	152.48	140.1	183.51	179.8	109.68	22.61	125.44	16.81
	OVCAR-3	170.15	172.68	197.44	186.09	170.92	37.65	151.95	55.35
	OVCAR-4	85.02	127.39	93.45	72.6	81.82	21.36	99.19	35.44
	OVCAR-5	ND	ND	ND	ND	ND	−7.82	ND	−31.07
	OVCAR-8	110.91	127.42	160.88	109.4	129.17	31.27	124.61	19.93
	NCI/ADR-RES	125.18	190.43	189.08	170.24	74.08	20.19	126.29	12.56
	SK-OV-3	137.06	139.59	189.4	184.04	103.95	78.06	134.02	59.62
Renal cancer	786–0	154.07	178.56	184.47	167.47	134.89	ND	143.36	ND
	A498	131.9	175.42	151.87	148.8	161.7	71.47	130.04	16.24
	ACHN	80.59	171.31	176.89	137.72	62.55	8.83	67.32	0.77
	CAKI-1	138.4	161.64	169.83	162.28	90.02	15.96	81.69	17.19
	RXF 393	177.56	185.1	188.98	187.48	172.82	44.01	188.55	38.11
	SN12C	168.92	183.61	196.75	195.25	122.54	21.6	154.7	43.61
	TK-10	78.49	151.34	117.3	102.24	67.97	9.91	82.25	0.88
	UO-31	ND	ND	ND	ND	ND	23.11	ND	23.76
Prostate cancer	PC-3	127.01	180.45	173.22	154.99	90.79	19.57	122.51	25.91
	DU-145	68.09	84.71	84.1	86.5	77.02	18.77	59.17	5.27
Breast cancer	MCF7	195.65	149.33	194.33	194.23	127.79	99.62	149.03	93.23
	MDA-MB-231/ATCC	ND	ND	ND	ND	ND	17.67	ND	−6.41
	HS 578T	158.52	180.61	194.33	170.22	168.99	0.58	166.04	11.56
	BT-549	160.94	183.02	181.55	163.95	160.26	54.76	162.17	44.76
	T-47D	ND	ND	ND	ND	ND	24.07	ND	35.7
	MDA-MB-468	176.19	177.17	192.7	186.56	162.79	101.55	166.02	121.82
Mean	​	136.63	159.77	168.59	157.56	124.5	51.39	130.91	49.52

**TABLE 2:** Growth inhibition percentages (GI%) of compounds **8i–o** at a single concentration (10 μM) across the NCI-60 human cancer cell line panel.

Cancer subpanel	Cancer cell line	Compound						
		8i	8j	8k	8l	8m	8n	8o
Leukemia	CCRF-CEM	162.55	161.27	139.75	149.43	197.93	79.43	195.31
	HL-60(TB)	198.22	198.15	198.33	199.39	198.71	199.14	198.28
	K-562	47.31	54.06	34.96	13.75	179.83	−6.88	61.75
	MOLT-4	194.86	198	191.02	171.39	199.24	174.23	198.37
	RPMI-8226	198.05	197.45	199.03	199.5	199.14	199.19	198.08
	SR	ND	ND	ND	ND	ND	ND	ND
NSCLC	A549/ATCC	32.11	42.45	66.81	36.23	107.04	72.99	59.38
	EKVX	42.57	92.28	82.61	60.77	183.89	25.59	117.41
	HOP-62	149.26	120.29	48.57	135.69	128.46	135	108.4
	HOP-92	40.02	23.43	36.82	26.68	130.88	17.11	101.49
	NCI-H226	88.8	116.5	133.67	97.9	125.71	72.72	123.02
	NCI-H23	100.35	150.85	131	83.74	188.61	80.24	153.58
	NCI-H322M	33.01	39.88	41.29	13.94	98.23	0.81	80.02
	NCI-H460	72.7	137.52	100.89	64.16	192.22	68.69	139.53
	NCI-H522	73.91	77.15	41.92	49.11	192.24	34.59	129.98
Colon cancer	COLO 205	41.97	50.81	51.46	24.57	168.62	10.5	75
	HCC-2998	127.85	176.7	195.03	192.59	197.78	192.45	197.63
	HCT-116	185.24	189.68	135.95	163.98	194.68	166.78	192.28
	HCT-15	113.77	161.1	166.99	174.35	187.9	160.86	183.43
	HT29	81.18	101.28	175.52	145.06	137.47	150.48	133.97
	KM12	154.4	168.46	110.25	140.7	184.43	157.63	171.96
	SW-620	86.16	164.92	187.69	136.88	194.07	183.68	183.79
CNS cancer	SF-268	107.92	80.28	99.23	99.57	126.88	76.61	104.39
	SF-295	33.14	56.1	41.12	31.5	131.49	37.09	84.23
	SF-539	191.3	175.6	196.3	192.01	196.58	164.19	191.99
	SNB-19	87.66	88.63	109.24	92.9	111.1	80.45	101.32
	SNB-75	67.37	73.9	59.62	19.3	123.12	34.63	122.28
	U251	149.17	158.01	180.32	167.73	173.6	126.34	175.12
Melanoma	LOX IMVI	151.7	179.69	190.49	167.25	195.4	172.83	192.92
	MALME-3M	99.45	128.95	85.3	105.53	177.78	−37.23	130.84
	M14	96.87	169.24	187.61	175.12	190.48	166.05	176.55
	MDA-MB-435	126.03	99.28	151.43	147.72	123.52	154.63	156.18
	SK-MEL-2	166.18	170.75	175.77	170.21	195.72	138.12	187.07
	SK-MEL-28	148.16	153.01	160.3	143.58	175.36	95.69	139.6
	SK-MEL-5	162.11	191.43	166.15	170.41	198.54	42.95	194.04
	UACC-257	136.2	158.96	178.86	155.96	194.23	166.34	163.92
	UACC-62	144.74	134.74	150.11	123.14	181.16	81.91	161.74
Ovarian cancer	IGROV1	123.87	149.01	117.65	107.19	143.66	99.26	136.27
	OVCAR-3	98.55	151.36	101.62	87.84	177.51	65.1	172.03
	OVCAR-4	43.75	54.55	44.64	24.39	129.34	23.21	98.32
	OVCAR-5	ND	ND	ND	ND	ND	ND	ND
	OVCAR-8	159.68	131.01	158.5	144.86	133.64	156.39	109.24
	NCI/ADR-RES	95.27	81.93	83.8	68.44	191.25	34.15	134
	SK-OV-3	113.66	140.41	161.97	140.26	130.41	107.95	128.37
Renal cancer	786–0	139.95	161.8	175.61	119.04	171.94	91.2	164.8
	A498	72.07	167.41	171.25	81.22	149.7	83.21	153.71
	ACHN	43.14	71.11	25.72	34.91	161.38	22.83	80.89
	CAKI-1	61.01	103.83	67.57	56.58	162.19	36.67	131.2
	RXF 393	178.24	179.67	185.06	184.14	178.07	188.42	171.05
	SN12C	90.47	172.09	140.88	120.63	189.09	80.3	179.89
	TK-10	38.83	66.25	46.33	27.25	151.31	30.25	120.31
	UO-31	ND	ND	ND	ND	ND	ND	ND
Prostate cancer	PC-3	73.6	151.99	139.4	105.86	176.72	73.68	144.68
	DU-145	29.26	44.62	41.76	25.7	91.11	8.86	64.1
Breast cancer	MCF7	125.55	191.79	157.89	185.15	191.03	146.31	188.27
	MDA-MB-231/ATCC	ND	ND	ND	ND	ND	ND	ND
	HS 578T	171.31	173.24	127.29	183.63	146.41	183.38	171.31
	BT-549	155.35	187.41	176.15	179.61	185.81	182.21	155.35
	T-47D	ND	ND	ND	ND	ND	ND	ND
	MDA-MB-468	179.52	161.41	163.98	188.49	23.64	176.04	179.52
Mean	​	130.29	128.11	113.14	166.58	95.99	145.52	130.29

The average growth inhibition values throughout the entire NCI-60 panel offer an immediate, quantifiable assessment of breadth (the number of responsive lines) and depth (the intensity of their response) at 10 μM. Significantly, as these are single-dose data, the average should be regarded as a screening-level summary: elevated averages indicate generally robust antiproliferative efficacy at this concentration, while diminished averages may signify either actually weak overall activity or a profile influenced by a limited number of sensitive outliers among numerous non-responders.

The average values in this dataset sort the series into several performance levels. Compounds **8c** (168.59%) and **8m** (166.58%) exhibit the highest overall mean GI% values, followed by **8b** (159.77%) and **8d** (157.56%), suggesting that at the measured concentration, the predominant phenotype across the panel is complete cytotoxicity (>100) rather than partial growth inhibition. This interpretation is supported by the fact that these compounds consistently exceed the cytotoxicity threshold across evaluated lines: for example, **8m** is > 100 in 53/55 reported cell lines, while **8c** and **8d** are >100 in 51/55 and **8b** is > 100 in 52/55, indicating that their high averages are not “carried” by just a few of extreme values, but rather reflect broad, significant responses across the majority of the panel.

The second level, **8a** (136.63%), **8g** (130.91%), **8j** (130.29%), **8k** (128.11%), **8e** (124.50%), and **8o** (145.52%), exhibits strong overall activity, but with a more uneven distribution compared to the top tier. Although their average GI% values exceeded 100%, indicating frequent cytotoxicity, a larger number of individual cell lines exhibited partial inhibition (GI% between 0 and 100) compared to the most active compounds. For example, **8a** exhibits cytotoxicity in 42 out of 55 lines and partial effects in 13 out of 55, whereas **8e** shows cytotoxicity in 39 out of 55 lines with partial effects in 16 out of 55, suggesting widespread activity that is strong in many lines but not consistently cytotoxic along all lines.

The middle profiles are represented by **8i** (109.17%) and, more specifically, **8n** (95.99%). Compound **8i** averages slightly above the cytotoxic threshold, indicating a nearly equal distribution of cytotoxic (29/55) and partial (26/55) effects with substantial activity, however with a noteworthy variability between cell lines.

Conversely, **8n**, with a mean below 100, predominantly leans towards partial inhibition (30 out of 55 partial; 23 out of 55 cytotoxic), and also encompasses few growth-enhancing signals (negative values in 2 instances). This exemplifies a common single-dose “borderline” pattern: sensitive models are evident, while the overall panel average fails to attain the anticipated threshold for widespread cytotoxicity at 10 μM.

At the lower spectrum, **8f** (51.39%) and **8h** (49.52%) exhibit reduced overall efficacy by mean, and the configuration of their distributions elucidates the justification for this. Their median GI% values are low (about 31 and 35, respectively), suggesting that the majority of cell lines exhibit only mild to moderate inhibition at 10 μM, with the mean distorted by a limited number of high-response outliers. Furthermore, these two compounds are the most susceptible to growth-promoting signals: **8f** exhibits negative values in three lines, whereas **8h** displays them in nine lines, highlighting uneven performance throughout the panel and reinforcing the conclusion that their antiproliferative effect is quite mild under single-dose parameters.

Although mean values are effective in illustrating general trends, they might hide significant outliers and cell line-specific influences. For example, **8e**, with an average of 124.5%, displayed significant inhibition across multiple lines, including HCT-116 (172.8%), RXF 393 (172.82%), and UACC-257 (133.49%), indicating strong efficacy in colon, renal, and melanoma models, respectively. Likewise, **8m** surpassed 190% inhibition in HCT-116, NCI-H460, and MCF-7, indicating significantly potent effects in colon, lung, and breast cancer models.

In leukemia models, pronounced variability in sensitivity was evident across both cell lines and compounds. K-562 displayed generally low sensitivity and showed apparent growth stimulation only in response to **8n** (−6.88%). In contrast, RPMI-8226 was uniformly the most sensitive, with GI% values ranging from 197.45% to 199.50% across all 15 compounds. Another compound illustrating intermediate activity, compound **8f** exhibited only moderate effects in CCRF-CEM and MOLT-4 (69.53% and 85.93%, respectively), remaining well below the strong cytotoxic responses recorded for the most active derivatives, particularly **8c** and **8m**, which achieved GI% values of approximately 198%–199% in the same cell lines.

A consistent trend was noted in colon cancer models, wherein many compounds specifically **8c**, **8d**, and **8m** attained levels beyond 150% in HCT-116, HCC-2998, and SW-620. This tendency was observed in various epithelial tumors, with compounds such as **8d**, **8m**, and **8b** frequently achieving or above 100% inhibition in NSCLC and breast cancer cell lines, including NCI-H460, A-549, and MDA-MB-468. These data highlight that robust activity is not confined to singular cell types but is apparent across various histological subtypes.

Despite numerous drugs’ good overall performance, cell line-specific responses indicated incidences of apparent resistance or poor impact. For example, OVCAR-5, UO-31, and T-47D commonly displayed low or negative growth inhibition values, indicating lower susceptibility to most investigated compounds in these models. Conversely, specific models such RPMI-8226, SF-539, U251, and MDA-MB-468 shown consistently elevated sensitivity throughout the panel, with growth inhibition often above 175%. These cell lines, due to their consistent response, may function as valuable reference points for future comparisons or screening activities.

##### 3.2.1.2 *In Vitro* five-dose screening

In light of the significant and encouraging results noted in the initial one-dose NCI-60 screening, the National Cancer Institute chose all fifteen compounds **8a–o** for progression to the five-dose assay. In the NCI-60 five-dose screening, compounds that satisfy the one-dose activity criteria are subsequently evaluated against the complete 60-cell panel at five concentrations (0.01, 0.1, 1, 10, and 100 μM). The concentration–response data are employed to compute three standard endpoints for each cell line: GI_50_ (the concentration yielding 50% growth inhibition), TGI (the concentration resulting in total growth inhibition, i.e., no net growth), and LC_50_ (the concentration causing 50% net cell loss), as illustrated in Supplementary Tables S1–S5.

The full-panel mean GI_50_ (MIDa) indicated that the lowest values were recorded for **8m** (2.51 μM), **8d** (3.43 μM), and **8c** (4.42 μM), succeeded by **8o** (5.16 μM), 8b (5.22 μM), and **8a** (5.28 μM). Intermediate MIDa values were recorded for **8g** (7.51 μM), **8j** (7.96 μM), and **8e** (8.98 μM), followed by **8i** (10.86 μM) and **8l** (13.77 μM). Elevated MIDa values were observed for **8h** (17.13 μM) and **8f** (17.56 μM), but **8k** (24.01 μM) and **8n** (58.19 μM) exhibited significantly lower whole-panel averages.

**FIGURE 5:**
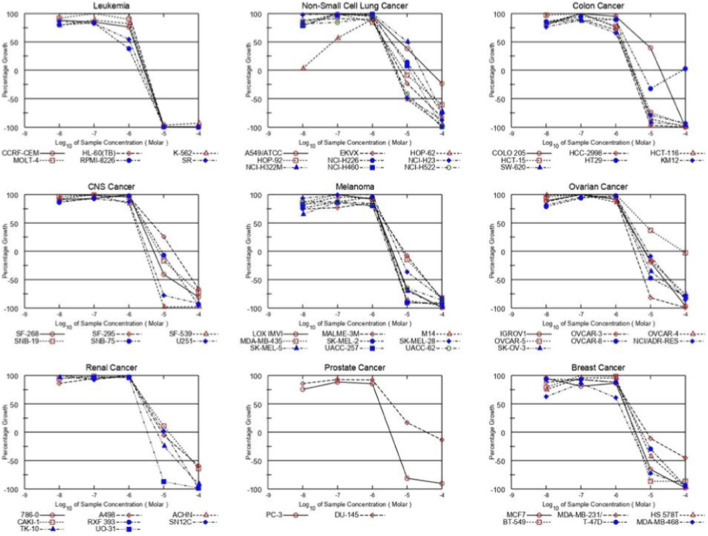
Dose-antiproliferative response of 8m against nine different cancer cell lines.

In addition to the MIDa rating, the five-dose tables illustrates distinct variations in the frequency with which the tested compounds transitioned from growth inhibition to growth arrest and subsequently to cytotoxicity. In 52 out of 56 documented cell lines, a GI_50_ of <5 μM was noted for compound **8m**, and TGI values were attained within the tested range across all reported lines (no TGI values above 100 μM were recorded), with LC_50_ values of ≤10 μM in 26 out of 57 lines, Figure 5. Compounds **8c** and **8d** exhibited broad low-micromolar GI_50_ activity (GI_50_ < 5 μM in 56/59 and 52/59 lines, respectively), with **8c** notably attaining cytotoxic levels more frequently in sensitive cells (LC_50_ ≤ 10 μM in 35/59 lines).

Conversely, compounds **8k** and **8n** often failed to achieve TGI and LC_50_ within the examined range, despite exhibiting low GI_50_ values in certain lines. For instance, **8k** exhibits TGI >100 μM in 38 out of 53 lines and LC_50_ ≥ 100 μM in 47 out of 56 lines, while **8n** exhibits TGI >100 μM in 37 out of 45 lines and LC_50_ ≥ 100 μM in 50 out of 55 lines. This shows that these analogs can limit growth in certain cells, however they typically fail to achieve complete growth inhibition or cytotoxicity.

Selectivity patterns are recognized in Supplementary Tables S1–S5 as MIDa/MIDb, with MIDb representing the mean GI_50_ for a certain tumor subpanel. A recurrent observation across the newly developed compounds is that leukemia exhibits the highest selectivity among subpanels for most analogs, with MIDa/MIDb ratios typically exceeding 2.0, whereas NSCLC often presents MIDa/MIDb values below 1.0.

Compound **8c** exhibits a selectivity of 2.93 for leukemia (MIDb = 1.49 μM; MIDa/MIDb = 2.93), 2.77 for colon cancer (MIDb = 1.59 μM; selectivity = 2.77), and 2.55 for breast cancer (MIDb = 1.73 μM; selectivity = 2.55), whereas its selectivity for ovarian cancer is markedly diminished (MIDb = 16.57 μM; selectivity = 0.27). Compound **8d** similarly demonstrates substantial selectivity for leukemia cells (2.30) and colon cancer (2.12) but exhibits diminished selectivity in ovarian cancer (0.54) and NSCLC (0.61). Conversely, **8m** exhibits diminished selectivity ratios, below 2, for leukemia, colon, and breast cancer lines, indicating that its decreased MIDa is supported by efficacy across several subpanels rather than being primarily driven by a single tumor type.

The highest selectivity ratios were observed for **8k** and **8n**. The **8k** ratios show elevated levels in breast cancer (11.67), leukemia (10.76), and colon cancer (9.31), but have reduced selectivity in other subpanels, such as prostate cancer (0.46). For **8n**, the selectivity for leukemia is exceptionally high (25.50), whereas the MIDb values for NSCLC and renal cancer exceed 100 μM, with a selectivity of 0.58, indicating low activity in those subpanels.

Cell line-specific data further confirm these subpanel findings by identifying individual models that exhibit pronounced cytostatic and cytotoxic responses to the tested compounds. Among these, the leukemia cell line RPMI-8226 consistently ranks among the most sensitive models across the most active compounds, exhibiting low GI_50_, TGI, and LC_50_ values. Specifically, **8c** (GI_50_ = 0.24 μM; TGI = 0.52 μM; and LC_50_ = 1.34 μM), **8o** (0.30/0.66/2.17 μM), **8m** (0.54/1.89/4.36 μM), and **8d** (0.71/1.96/4.45 μM). The SR leukemia line demonstrates consistent sensitivity, exemplified by **8m** (1.07/2.26/4.78 μM), **8o** (1.17/2.40/4.94 μM), **8c** (1.42/2.72/5.24 μM), and **8d** (1.52/2.85/5.37 μM).

In colon cancer, HCT-116, HCC-2998, and KM12 are frequent examples of low-micromolar growth inhibition with progression to TGI and LC_50_ for the leading compounds; for instance, **8d** in HCT-116 (1.41/2.74/5.33 μM), **8c** in HCT-116 (1.25/2.53/5.13 μM) and KM12 (1.11/2.41/5.20 μM), and **8m** in HCC-2998 (1.34/2.66/5.27 μM) and KM12 (1.28/2.84/6.28 μM). Breast cancer sensitivity is also evident in MCF-7 and MDA-MB-468 for several analogs, including **8d** in MCF-7 (1.31/2.63/5.30 μM), **8c** in MCF-7 (1.33/2.66/5.34 μM) and MDA-MB-468 (1.27/2.61/5.37 μM), and **8o** in MCF-7 (1.72/3.20/5.94 μM) and MDA-MB-468 (1.91/3.85/7.74 μM). Notably, some NSCLC lines can still be sensitive despite NSCLC being less responsive overall; EKVX shows low-micromolar values for **8d** (GI_50_ 1.35 μM; TGI 2.83 μM; LC_50_ 5.95 μM) and **8c** (GI_50_ 1.12 μM; TGI 2.40 μM; LC_50_ 5.15 μM).

At the same time, resistant models are clearly present and often show TGI and LC_50_ values that remain >100 μM even when GI_50_ is within the micromolar range. OVCAR-5 [Ovarian Cancer] is a prominent example: **8c**, **8k**, and **8n** are reported as >100 μM for GI_50_/TGI/LC_50_ in this line, while even more active analogs show limited cytotoxicity (for example, **8m**: GI_50_ 6.31 μM; TGI 84.8 μM; LC_50_ > 100 μM; **8d**: GI_50_ 28.4 μM; TGI >100 μM; LC_50_ > 100 μM). NSCLC lines A549/ATCC and NCI-H322M also illustrate incomplete progression to cytotoxicity for many compounds; for example, **8m** in A549/ATCC (GI_50_ 6.28 μM; TGI 41.6 μM; LC_50_ > 100 μM) and in NCI-H322M (GI_50_ 10.1 μM; TGI 25.4 μM; LC_50_ 64.3 μM), while **8k** and **8n** show complete lack of response in A549/ATCC and NCI-H322M (GI_50_/TGI/LC_50_ all >100 μM).

Upon analyzing the five-dose data concerning the substitutions on the two aryl rings of the scaffold (the aryl ring of the chalcone segment and the aryl ring derived from the phenacyl bromide), distinct structure–activity relationships become evident.

First, within the analogs bearing an unsubstituted phenacyl-derived aryl ring (**8a**–**8e**), changing only the chalcone aryl substituent shows that para-fluoro and para-methoxy are associated with improved whole-panel GI_50_ potency relative to the unsubstituted parent: **8d** (para-F) has MIDa 3.43 μM and **8c** (para-OCH_3_) has MIDa 4.42 μM, compared with **8a** (unsubstituted) at 5.28 μM. Para-chloro gives a similar MIDa (**8b**, 5.22 μM), while para-methyl reduces potency (**8e**, 8.98 μM), which is also reflected at the cell line level by higher GI_50_/TGI/LC_50_ values in several solid tumors (for example, **8e** in A549/ATCC: 16.5/34.5/72.3 μM; and in OVCAR-5: 27.8/65.9/>100 μM).

Second, introducing para-chloro on the phenacyl-derived aryl ring generally reduces activity when compared with the corresponding analogs lacking that substitution (**8a**→**8f**, 5.28→17.56 μM; **8c**→**8h**, 4.42→17.13 μM; **8d**→**8i**, 3.43→10.86 μM), although this effect is less severe when the chalcone ring bears para-chloro or para-methyl (**8b**→**8g**, 5.22→7.51 μM; and **8e**→**8j**, 8.98→7.96 μM). This pattern is consistent with the marked loss of activity of **8f** and **8h** in resistant solid tumor lines (for example, **8f** in OVCAR-5: 60.5/>100/>100 μM; and **8h** in A549/ATCC: 29.4/>100/>100 μM), while **8g** and **8j** retain low-micromolar activity in sensitive leukemia and colon lines (for example, **8j** in RPMI-8226: 1.01/2.22/4.86 μM; and in HCC-2998: 1.52/2.89/5.51 μM).

Third, *para*-methoxy on the phenacyl-derived aryl ring produces divergent effects that depend strongly on the chalcone substituent. With a para-methoxy group on both rings (**8m**), potency improves substantially (MIDa 2.51 μM) and several sensitive lines show low-micromolar GI_50_/TGI/LC_50_ values (for example, HCC-2998: 1.34/2.66/5.27 μM; and MDA-MB-468: 1.21/2.86/6.78 μM). In contrast, pairing para-methoxy phenacyl with para-fluoro on the chalcone ring (**8n**) leads to a very weak whole-panel mean (MIDa 58.19 μM), with many cell lines reported as >100 μM, while still retaining marked leukemia sensitivity (RPMI-8226: 1.30/2.58/5.12 μM) and a high leukemia selectivity ratio (MIDa/MIDb 25.50). A similar but less pronounced selectivity-driven pattern is seen for **8k** (chalcone unsubstituted; phenacyl para-methoxy), which maintains low-micromolar GI_50_ values in leukemia and some colon/breast models (for example, RPMI-8226: 0.56/1.63/4.05 μM; MCF-7: 1.46/3.15/6.80 μM) yet shows widespread lack of response in NSCLC and ovarian models (A549/ATCC and OVCAR-5: GI_50_/TGI/LC_50_ all >100 μM). Finally, combining chalcone para-methyl with phenacyl para-methoxy (**8o**) restores whole-panel potency (MIDa 5.16 μM) compared with the corresponding phenacyl para-methoxy analog with an unsubstituted chalcone ring (**8k**, 24.01 μM), indicating that this pair of substitutions is better tolerated in this scaffold.

Overall, the five-dose data indicate that **8m**, **8c**, **8d**, **8o** and **8b** provide the most favorable balance of low-micromolar GI_50_ values across many cell lines with frequent progression to low-micromolar TGI and, in many sensitive models, LC_50_ values in the single-digit micromolar range. Compounds **8k** and **8n**, while less active overall, show clear numerical selectivity for leukemia (and for **8k**, also breast and colon by MIDa/MIDb), but they commonly fail to reach TGI and LC_50_ in many solid tumor lines. These relationships between *para* substitution on the chalcone aryl ring and the phenacyl-derived aryl ring provide a practical basis for selecting representatives for follow-up mechanism studies.

#### 3.2.2 Cell viability assay

The safety of the synthesized compounds was evaluated by assessing the viability of the human mammary gland epithelial normal cell line (MCF-10A) in response to the novel compounds **8b**, **8c**, **8d**, **8m**, and **8o**. The cell viability of the new compounds was evaluated during a 4-day incubation with MCF-10A cells utilizing the MTT test (Alshammari et al., 2022). The results (Table 3) revealed that none of the evaluated compounds showed cytotoxicity in normal cells, as all compounds preserved cell viability above 90% at a concentration of 50 µM.

**TABLE 3:** Tubulin inhibitory assay of compounds **8b**, **8c**, **8d**, **8m**, and **8o**.

Compound	Cell viability%	R_1_	R_2_	Tubulin inhibition IC_50_ ± SEM (µM)
**8b**	91	Cl	H	7.19 ± 0.24
**8c**	90	OMe	H	16.54 ± 0.56
**8d**	93	F	H	4.10 ± 0.14
**8m**	90	OMe	OMe	3.86 ± 0.13
**8o**	92	Me	OMe	12.81 ± 0.43
**CA-4**	--	--	--	2.40 ± 0.08

--: not applicable.

#### 3.2.3 Tubulin inhibitory assay

The efficacy of compounds **8b**, **8c**, **8d**, **8m**, and **8o**, the most notable derivatives from five dosage trials, on tubulin polymerization (Al-Wahaibi et al., 2025), with CA-4 as a reference molecule, is presented in Table 3. Compounds **8b**, **8c**, **8d**, **8m**, and **8o** displayed potent anti-tubulin action, with IC_50_ values ranging from 3.86 to 16.54 µM, compared to the standard CA-4 (IC_50_ = 2.40 µM). In all instances, the examined compounds are less effective than the standard CA-4 as inhibitors of tubulin polymerization.

Compound **8m** (R_1_ = R_2_ = OMe) exhibited the highest potency as an inhibitor of tubulin polymerization, with an IC_50_ value of 3.86 µM. It exhibited 1.6-fold reduced potency relative to the standard CA-4. Compound **8d** (R_1_ = F, R_2_ = H) was ranked second in inhibiting tubulin polymerization, with an IC_50_ value of 4.10 µM. Compound **8d** had similar potency to compound **8m**, although displayed 1.7-fold reduced efficiency relative to CA-4. Compounds **8b**, **8c**, and **8o** exhibited moderate to weak inhibitory activity, with IC_50_ values of 7.19, 16.54, and 12.81 µM, respectively, demonstrating at least a threefold reduction in potency compared to the reference CA-4. These data demonstrated that compounds **8d** and **8m** exhibit the most advantageous equilibrium of low-micromolar GI_50_ values, low-micromolar TGI, and LC_50_ values within the single-digit micromolar range, suggesting their potential as tubulin polymerization inhibitors.

#### 3.2.4 Western blot analysis

**FIGURE 6:**
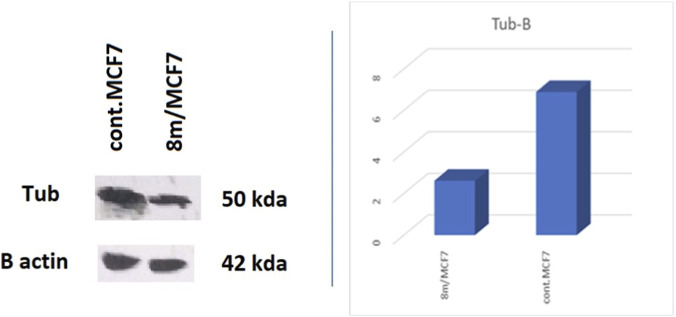
Effect of 8m on tubulin expression in MCF-7 cancer cell line.

The expression level of tubulin in the breast cancer cell line MCF-7 following treatment with compound **8m** was examined using the standard Western blot protocol (Ubukata et al., 2026). MCF-7 cells treated with **8m** showed a substantial reduction in tubulin expression compared to untreated control cells. Tubulin levels dropped by approximately 62% (2.6-fold), as seen in Table 4 and Figure 6. Because β-actin was used as a loading control, the data are considered semi-quantitative and normalized, indicating that the reduction is due to the compound’s effect. A reduction in tubulin, a structural component of microtubules, in MCF7 breast cancer cells frequently implies that the tested compound may act as a microtubule-destabilizing agent or impede cell division.

**TABLE 4:** Results of Western blot analysis of **8m** using MCF-7 cancer cell line.

Compound	Tubulin (OD)	β-actin
**8m**	2.62	√
Control	6.89	√

#### 3.2.5 Cell cycle analysis and apoptosis

Upon completion of the cell cycle, a cell produces two identical daughter cells. Each of the 2 cells derived from the initial cell can undergo this cell cycle again when additional cells are required (Dai et al., 2025). The cell cycle comprises four phases: G1 phase, S phase (synthesis), G2 phase, and M phase. During the G1 phase, cellular growth and preparation for DNA replication take place. The S phase is the period of DNA replication and chromatid duplication. During the G2 phase, the repair of newly synthesised DNA and further cellular growth transpire. Nuclear division occurs during the M phase (Liu, 2024; Li et al., 2026). We examine the effect of compound 8m on cell cycle progression and the induction of apoptosis in MCF-7 cells. MCF-7 cells were treated with an IC_50_ concentration of **8m** for 24 h. The cell line was stained with PI/Annexin V and subsequently analyzed using flow cytometry use the BD FACS Calibur (El-Sherief et al., 2018).

**FIGURE 7:**
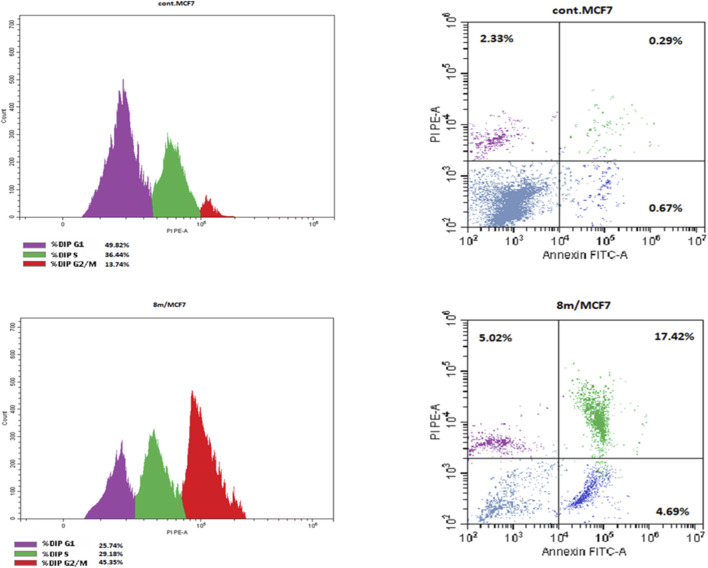
Cell cycle analysis and apoptotic detection of 8m in MCF-7 cancer cell line.

Treatment with compound **8m** resulted in a significant accumulation of cells in the G2/M phase, rising from 13.74% to 45.35% (about a 3.3-fold increase), Figure 7. This signifies that the substance inhibits cells from initiating or terminating mitosis. The transition is accompanied by an approximate 50% decrease in the G0/G1 population (from 49.82% to 25.47%), indicating that cells are advancing through the initial phase of the cycle but becoming “trapped” at the pre-mitotic stage. In MCF7 breast cancer cells, G2/M arrest is often linked to DNA damage or the suppression of microtubule dynamics. This corresponds precisely with Western blot findings indicating a reduction in tubulin (50 kDa) expression.

Additionally, treatment of MCF-7 cancer cell with compound **8m** markedly elevated overall apoptosis from 3.29% to 27.13%, Table 5. This signifies an eightfold augmentation in programmed cell death relative to the control group. The predominant occurrence of death is situated in the late apoptotic quadrant (17.42%). This shows that, at the time of the study, most cells progressed beyond the initial stages of apoptosis and began to lose membrane integrity. Despite a little increase in necrosis (from 2.33% to 5.02%), it remains a trivial component of total cell death. This signifies that **8m** activates a controlled programmed cell death pathway instead of non-specific toxic necrosis. The elevated incidence of late apoptosis is associated with the prior G2/M arrest (45.35%). This indicates that cells “trapped” in the G2/M phase by **8m** ultimately experience mitotic catastrophe, resulting in apoptosis.

**TABLE 5:** Apoptosis and necrosis induction of **8m** in MCF-7 cancer cell line.

Compound no.	Apoptosis			Necrosis
	Total	Early	Late	
**8m/MCF7**	27.13	4.69	17.42	5.02
**Cont. MCF7**	3.29	0.67	0.29	2.33

### 3.3 *In-silico* studies

#### 3.3.1 Molecular docking simulations

**FIGURE 8:**
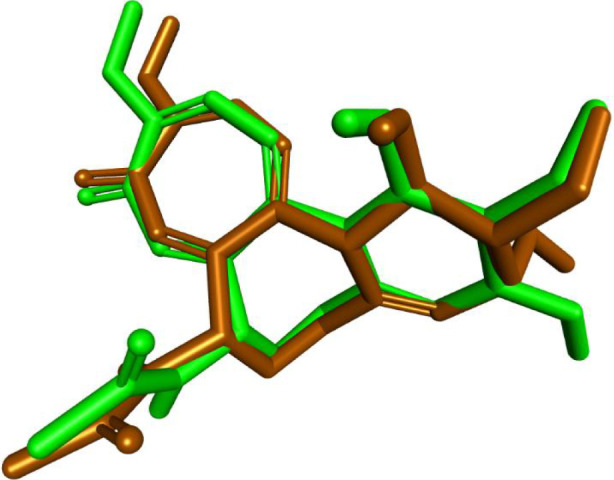
Superimposition of colchicine in the tubulin site: redocked (brown) vs. cocrystallized (green), RMSD = 0.8191 Å.

Molecular docking simulations were conducted using AutoDock Vina (Mukherjee et al., 2026) to investigate the binding interactions of compounds **8d** and **8m** with the colchicine binding site of tubulin (PDB ID: 4O2B) (Cheruku et al., 2025), and all docking poses were analyzed using Discovery Studio Visualizer (Wahab et al., 2026). To validate the docking protocol, colchicine was redocked into its crystallographic binding site, yielding a binding affinity of −9.6 kcal/mol and an RMSD of 0.8191 Å compared to the native pose. This RMSD is well within the accepted 2.0 Å threshold, confirming the reliability of the docking methodology. The superimposition of the redocked and crystallographic poses is presented in Figure 8.

Docking was subsequently accomplished for the two most potent derivatives, **8d** and **8m**, to clarify their binding modes and justify their enhanced biological profiles. Compound **8m**, with *para*-methoxy substituents on both aromatic rings, had a noteworthy binding affinity of −9.2 kcal/mol. This aligns closely with its strong antiproliferative efficacy and effective suppression of tubulin polymerization. The phenacyl methoxy moiety of **8m** develops a carbon-hydrogen link with Gln247 and π-alkyl interaction with Tyr224, whereas the aromatic system establishes π-sigma and π-Alkyl interactions between Leu248 and Ala250. The thiazole core establishes π-sigma and π-alkyl interactions with Leu248, Lys352, and Ala354, while its methyl substituent interacts with Ala316 and Leu255 in a lipophilic cavity.

**FIGURE 9:**
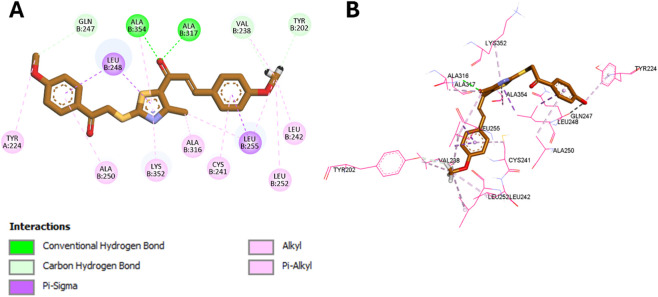
**(A)** 2D and **(B)** 3D binding interactions of compound 8m at the colchicine binding site.

The chalcone carbonyl forms dual hydrogen bonds with Ala317 and Ala354, which are critical for maintaining the colchicine binding site and preventing the curved-to-straight conformational shift of tubulin (Huang et al., 2026). Supplementary stabilizing interactions including π-alkyl and π-sigma contacts with Cys241 and Leu255, as well as carbon-hydrogen bonds with Val238 and Tyr202, all enhancing robust anchoring within the hydrophobic pocket. The interactions are illustrated in Figure 9.

**FIGURE 10:**
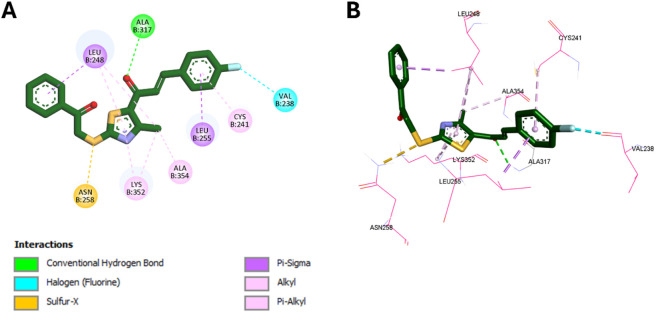
**(A)** 2D and **(B)** 3D binding interactions of compound 8d at the colchicine binding site.

In comparison, compound **8d** displayed a slightly lower binding affinity (−8.3 kcal/mol), consistent with its slightly reduced potency. It retains key interactions with Leu248, Lys352, and Ala354, and its thiazole methyl group forms hydrophobic contacts with these residues. A distinguishing feature of **8d** is a sulfur-X interaction between its thioether sulfur and Asn258, a residue known to engage in polar interactions with sulfur-containing ligands. Unlike **8m**, **8d** forms only a single hydrogen bond with Ala317, which may account for its reduced binding energy. Notably, its *para*-fluorine substituent participates in a halogen bond with Val238. The binding interactions of **8d** are illustrated in Figure 10.

#### 3.3.2 ADMET prediction

**FIGURE 11:**
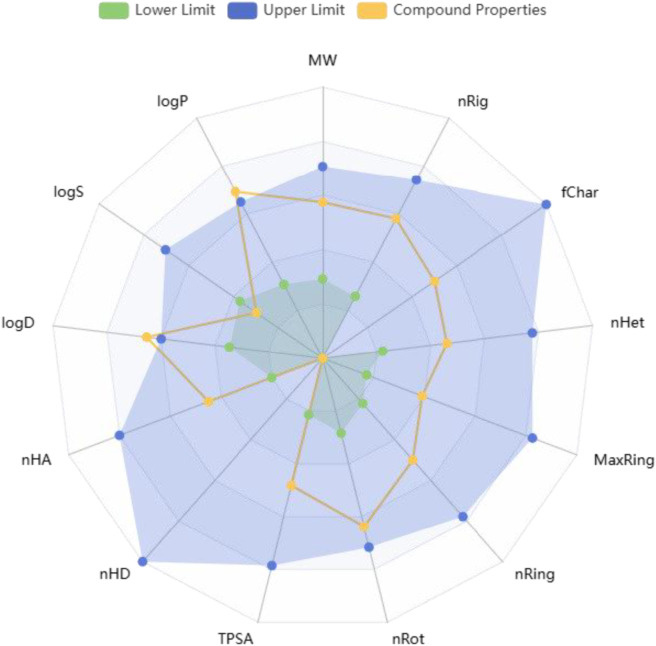
Bioavailability radar of compound 8m showing compliance with key drug-like regions.

*In silico* ADMET evaluation of compound **8m** using ADMETlab 3.0 (Tan et al., 2026) revealed a favorable drug-like profile with several properties supportive of oral bioavailability and lead-like behavior. The compound exhibited no Lipinski violations, a moderate logP (3.38), and a TPSA of 65.49 Å^2^, all within accepted bounds for passive permeability and intestinal absorption. The bioavailability radar (Figure 11) visually confirms this: lipophilicity, polarity, size, and electronic distribution fall well within optimal ranges, while saturation and flexibility are borderline, suggesting areas for potential scaffold refinement.

Compound **8m** displayed high predicted plasma protein binding (PPB = 98.3%) with low unbound fraction (Fu = 1.68%), which may influence distribution volume and free drug levels *in vivo*. Notably, no PAINS or BMS structural alerts were flagged, and reactivity and promiscuity scores were low, supporting specificity and chemical tractability. However, ALARM NMR returned multiple alerts, and the compound was predicted to inhibit several key CYP450 isoforms (1A2, 2C19, 2C9, 3A4), raising potential concerns for metabolic liability or drug–drug interactions. Clearance predictions also suggested a short half-life (T_1_/_2_ = 0.58 h) and moderate plasma clearance (6.3 mL/min/kg), consistent with its classification as human liver microsomal unstable.

Despite this, predicted cell permeability (Caco-2 and MDCK) and non-substrate status for P-gp efflux suggest that **8m** is likely to be absorbed if solubility and metabolic stability are improved. Toxicity predictions indicated low mutagenicity and hERG liability, but high probabilities for DILI and skin sensitization, which may warrant early *in vitro* follow-up. The compound also showed strong SR-p53 (0.897) and SR-ARE (0.741) pathway activation probabilities, possibly reflecting redox or DNA-damage linked stress responses relevant to its anticancer mode of action.

Together, these predictions suggest that while compound **8m** exhibits strong alignment with key drug-likeness and permeability criteria, optimization efforts may focus on metabolic stability and potential toxicity liabilities to improve its pharmacokinetic profile and therapeutic window.

### 3.4 Structure-activity relationship (SAR) analysis

**FIGURE 12:**
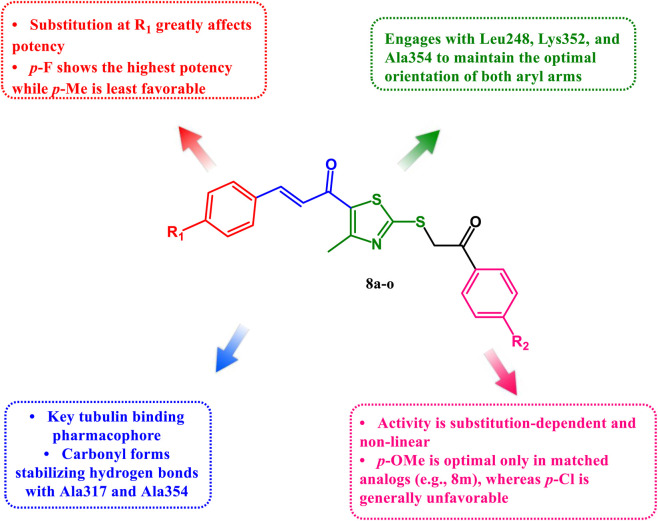
SAR overview of the target compounds 8a-o.

Figure 12 outline the SAR of new compounds **8a-o**.

## 4 Conclusion

This study presents the rational design, synthesis, and comprehensive biological evaluation of a new series of thiazole-based hybrids as microtubule destabilizing agents (MDAs) targeting the colchicine binding site of tubulin. All synthesized compounds (**8a**–**o**) were characterized and screened for antiproliferative activity across the NCI-60 human tumor cell line panel. Notably, compounds **8d** and **8m** emerged as the most significant analogs, displaying broad-spectrum cytotoxicity with low micromolar GI_50_ values, often progressing to full growth inhibition or cytotoxicity in diverse cancer types. Mechanistic investigations confirmed that both compounds inhibit tubulin polymerization *in vitro*, with compound **8m** exhibiting an IC_50_ of 4.10 µM, closely matching that of the reference agent CA-4. Cell cycle analysis further revealed that **8m** effectively induces G2/M phase arrest and apoptosis in MCF-7 cells, accompanied by a significant downregulation of tubulin levels. Molecular docking supported these findings by demonstrating favorable interactions with key residues within the colchicine-binding site, while *in silico* ADMET predictions highlighted acceptable oral bioavailability and drug-likeness. Taken together, biological activity, mechanistic validation, and computational profiling strongly support compounds **8d** and **8m** as promising lead candidates for further optimization in the development of tubulin-targeted anticancer agents.

## Data Availability

The original contributions presented in the study are included in the article/Supplementary Material, further inquiries can be directed to the corresponding authors.
