# Supplementary material for: Development of a novel series of thiazole-based compounds with enhanced antiproliferative properties as tubulin polymerization inhibitors
Source: Front Chem. 2026 Apr 22;14:1814119. doi: 10.3389/fchem.2026.1814119 (PMC13143880; doi:10.3389/fchem.2026.1814119)
Supplement: Supplementary file 1 [file DataSheet1.docx]

**Development of a novel series of thiazole-based compounds with enhanced antiproliferative properties as tubulin polymerization inhibitors**

Lamya H. Al-Wahaibi^1^, Ali M. Elshamsy^2^, Taha F.S. Ali^3^, Bahaa G. M. Youssif*^4^, Stefan Bräse^5^*, Mohamed Abdel-Aziz*^3,6^, Nawal A. El-Koussi^7^

^1^Department of Chemistry, College of Sciences, Princess Nourah bint Abdulrahman University, Riyadh 11671, Saudi Arabia; ^2^Pharmceutical Chemistry Department, Faculty of Pharmacy, Deraya University, Minia, Egypt; ^3^Medicinal Chemistry Department, Faculty of Pharmacy, Minia University, Minia 61519, Egypt; ^4^Department of Pharmaceutical Organic Chemistry, Faculty of Pharmacy, Assiut University, Assiut-71526, Egypt; ^5^Institute of Biological and Chemical Systems, IBCS-FMS, Karlsruhe Institute of Technology, 76131 Karlsruhe, Germany; ^6^Medicinal Chemistry Department, Faculty of Pharmacy, Minia national University, Minia, Egypt; ^6^Department of Pharmaceutical Medicinal Chemistry, Faculty of Pharmacy, Assiut University, Assiut, Egypt.

*To whom correspondence should be addressed:

**Mohamed Abdel-Aziz**, Ph.D. Department of Medicinal Chemistry, Faculty of Pharmacy, Minia University, 61519-Minia, Egypt and Medicinal Chemistry Department, Faculty of Pharmacy, Minia National University, Minia, Egypt.
Tel.: +2101003311327; E-mail address: [abulnil@](mailto:abulnil@)mu.edu.eg

**Bahaa G. M. Youssif**, Ph.D. Pharmaceutical Organic Chemistry Department, Faculty of Pharmacy, Assiut University, Assiut 71526, Egypt.
Tel.: +201044353895; E-mail address: [bgyoussif2@gmail.com](mailto:bgyoussif2@gmail.com)

**Stefan Bräse**

Institute of Biological and Chemical Systems, IBCS-FMS, Karlsruhe Institute of Technology, 76131 Karlsruhe, Germany. E-mail: [braese@kit.edu](mailto:braese@kit.edu)


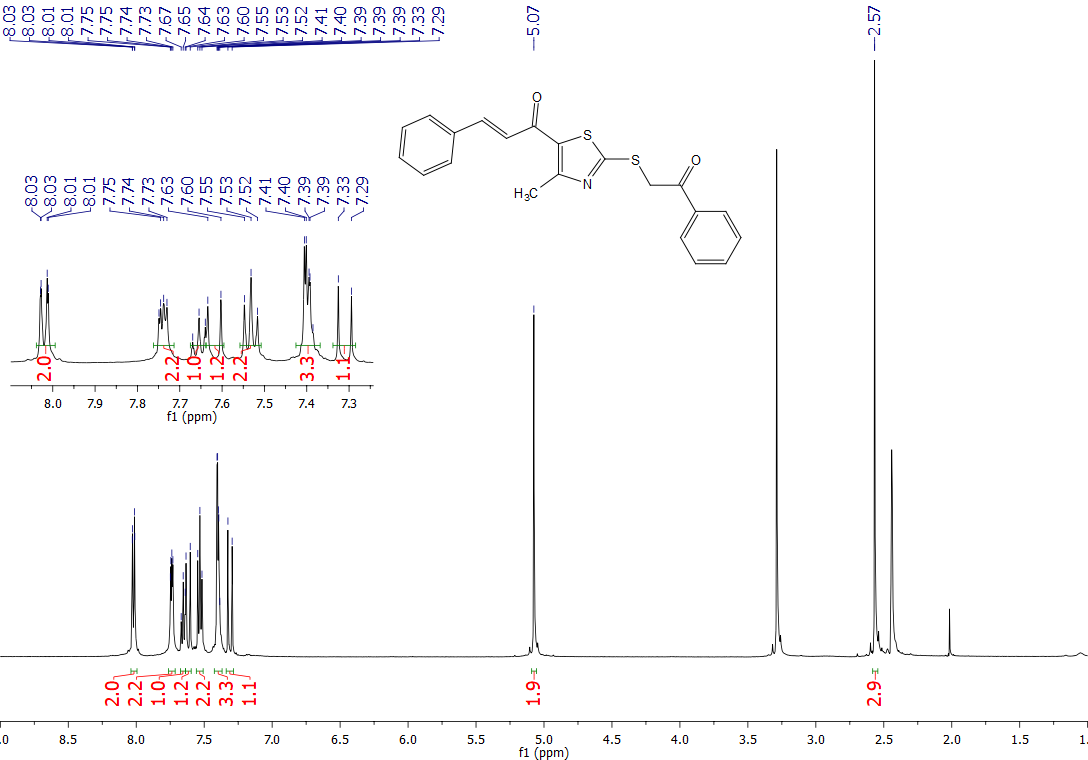


**Figure S1.** ^1^H NMR spectrum of compound **8a**


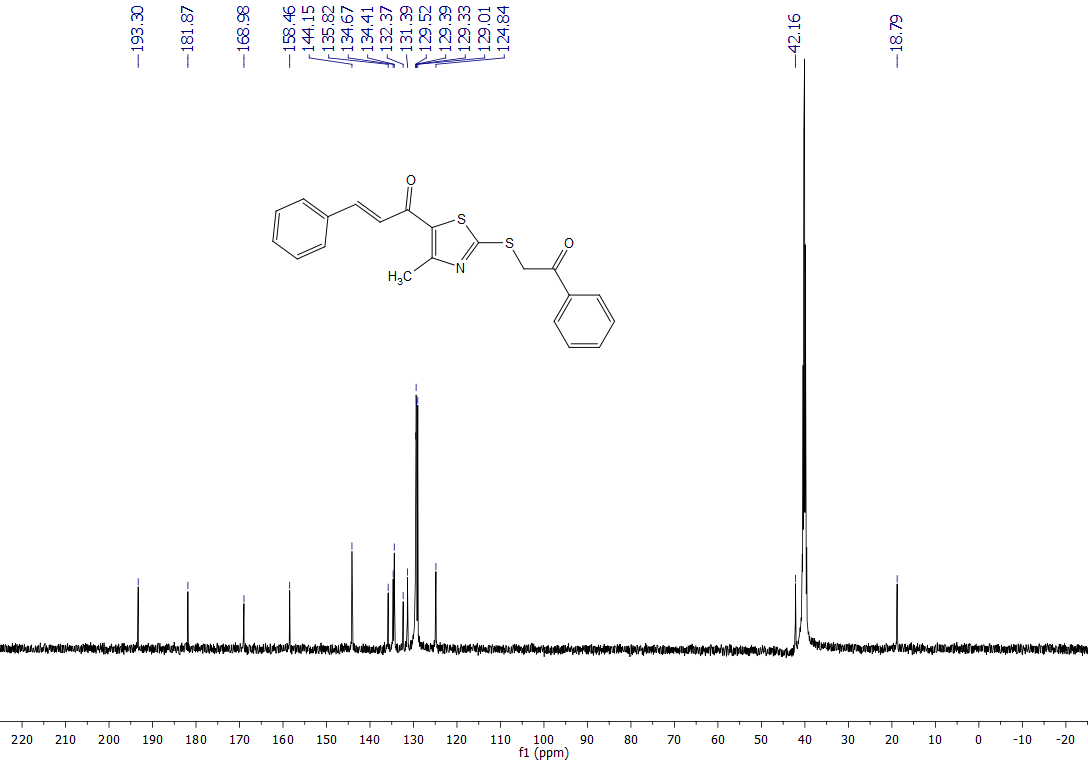


**Figure S2.** ^13^C NMR spectrum of compound **8a**


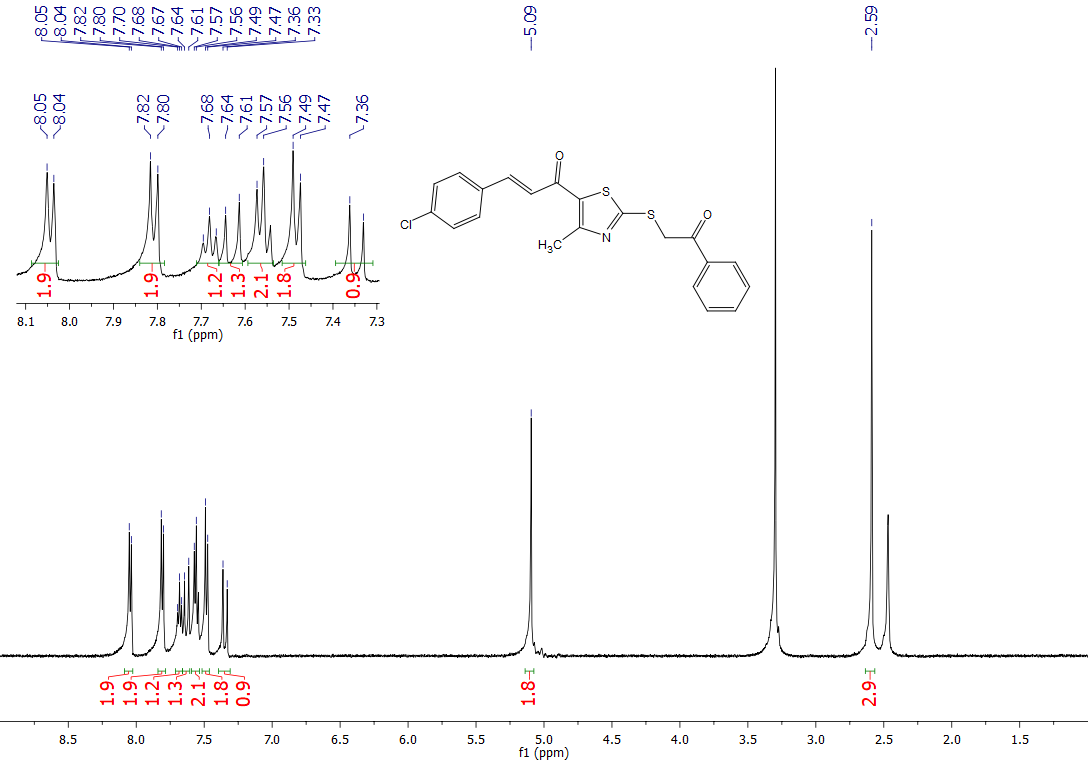


**Figure S3.** ^1^H NMR spectrum of compound **8b**


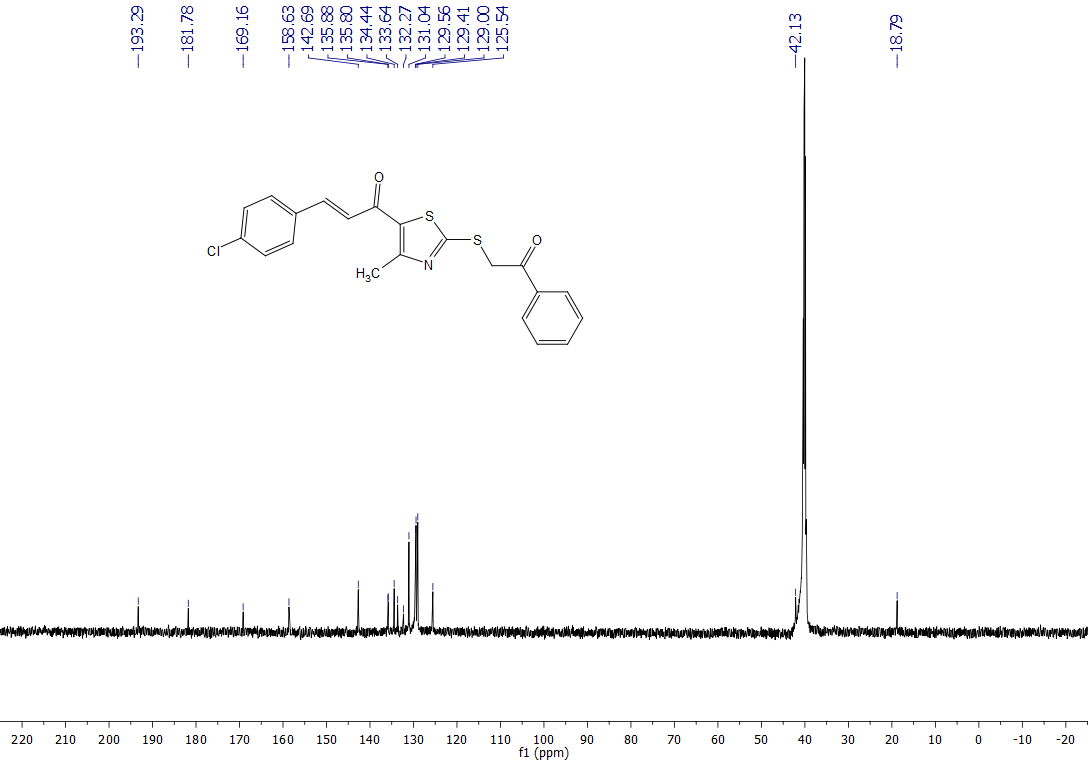


**Figure S4.** ^13^C NMR spectrum of compound **8b**


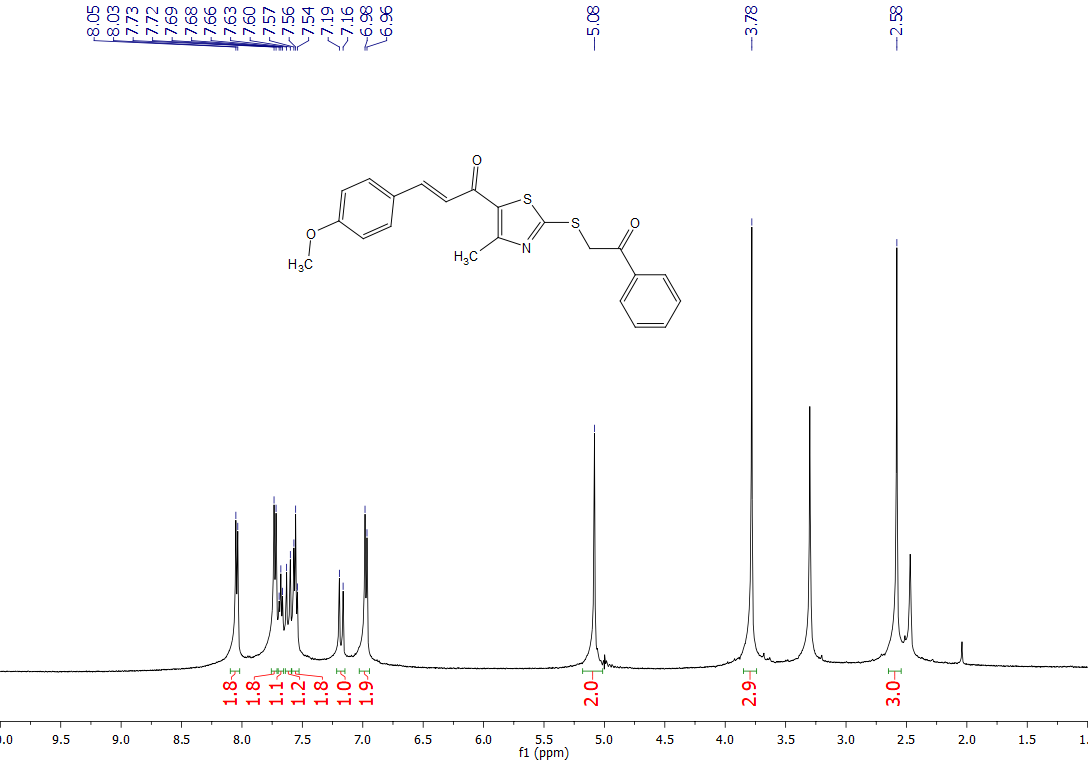


**Figure S5.** ^1^H NMR spectrum of compound **8c**


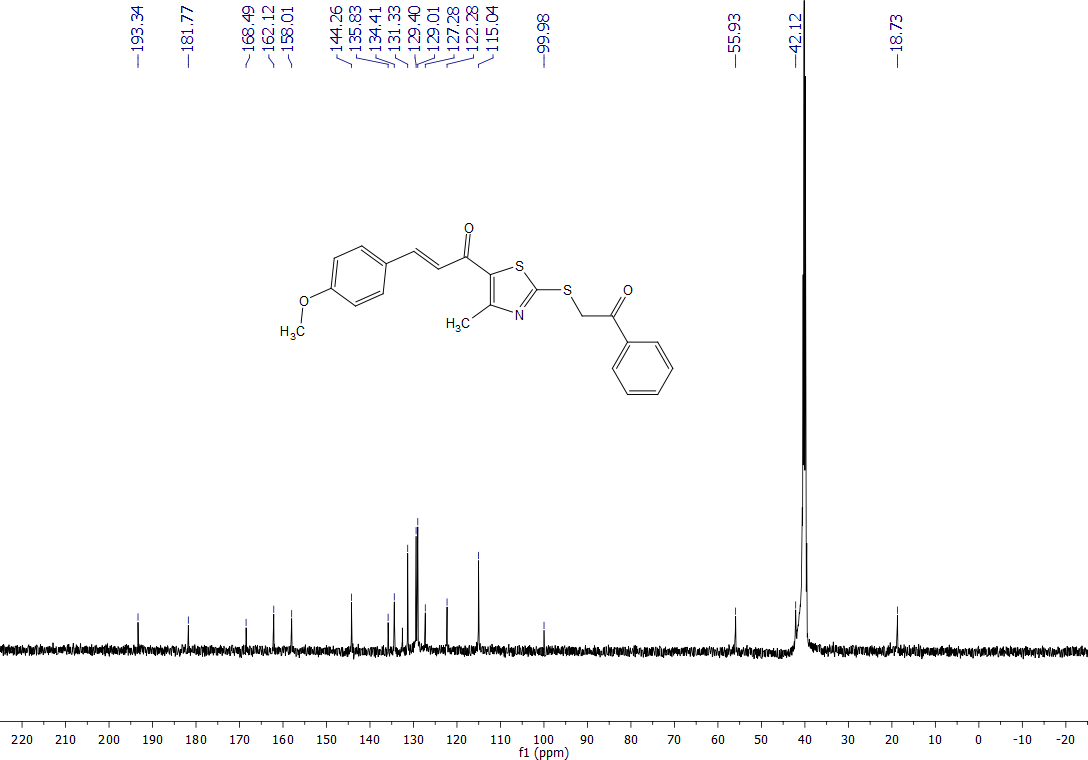


**Figure S6.** ^13^C NMR spectrum of compound **8c**


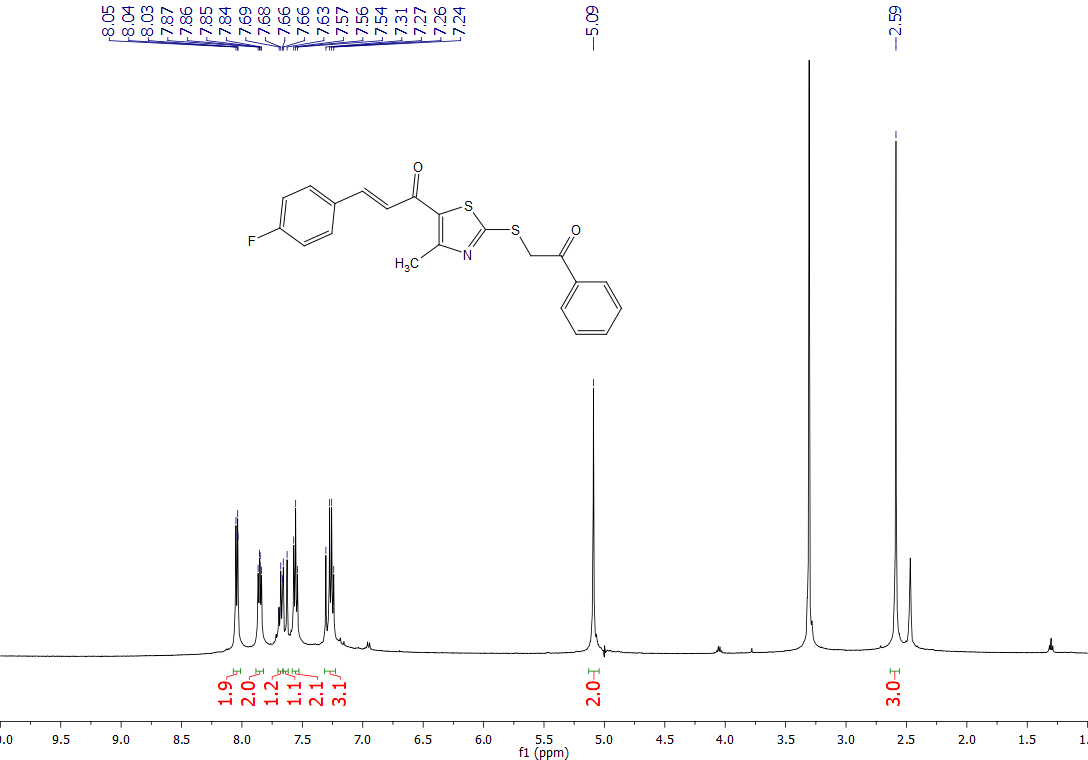


**Figure S7.** ^1^H NMR spectrum of compound **8d**


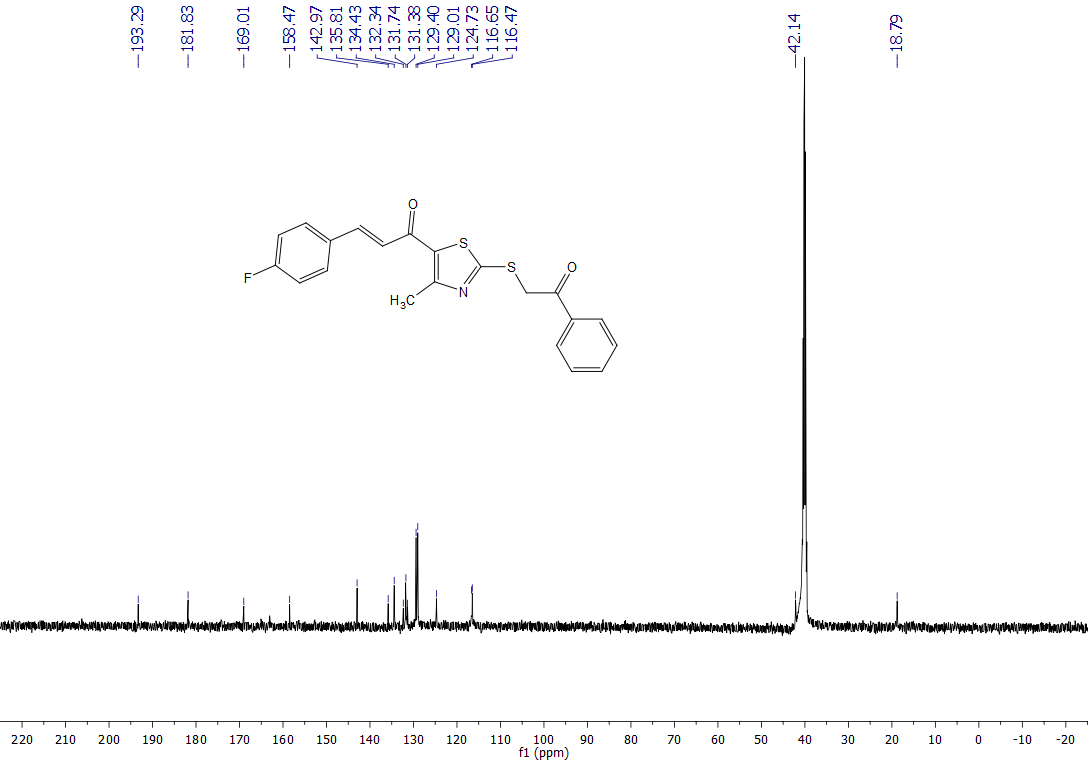


**Figure S8.** ^13^C NMR spectrum of compound **8d**


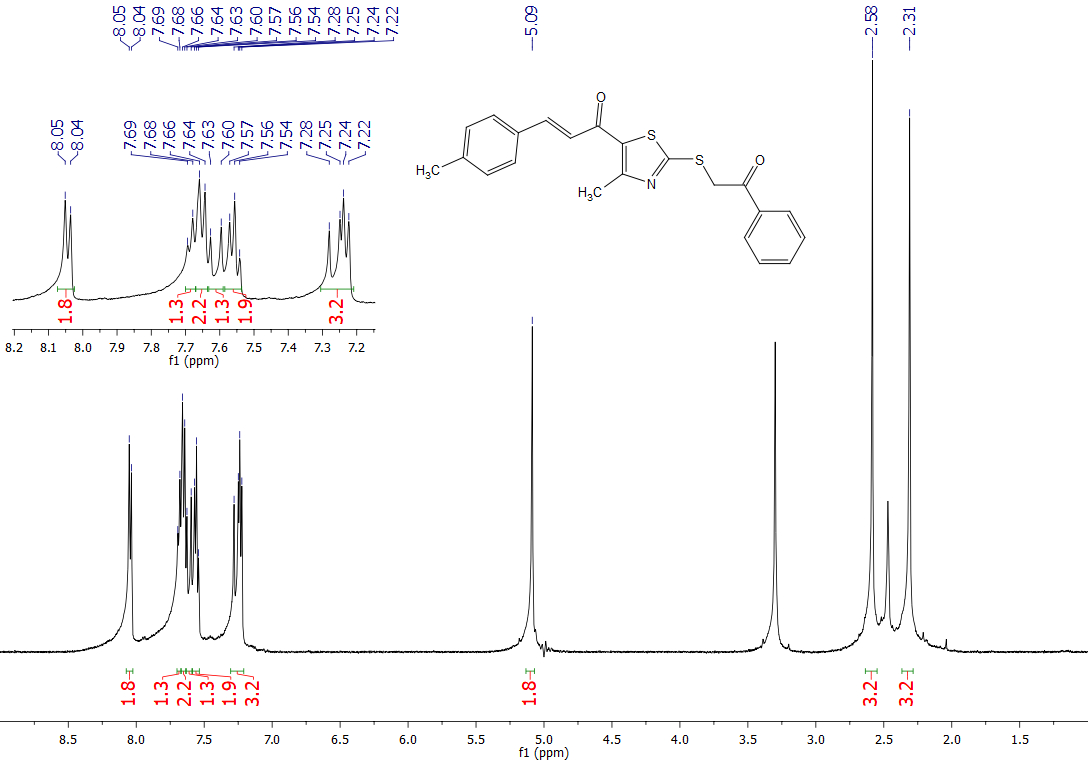


**Figure S9.** ^1^H NMR spectrum of compound **8e**


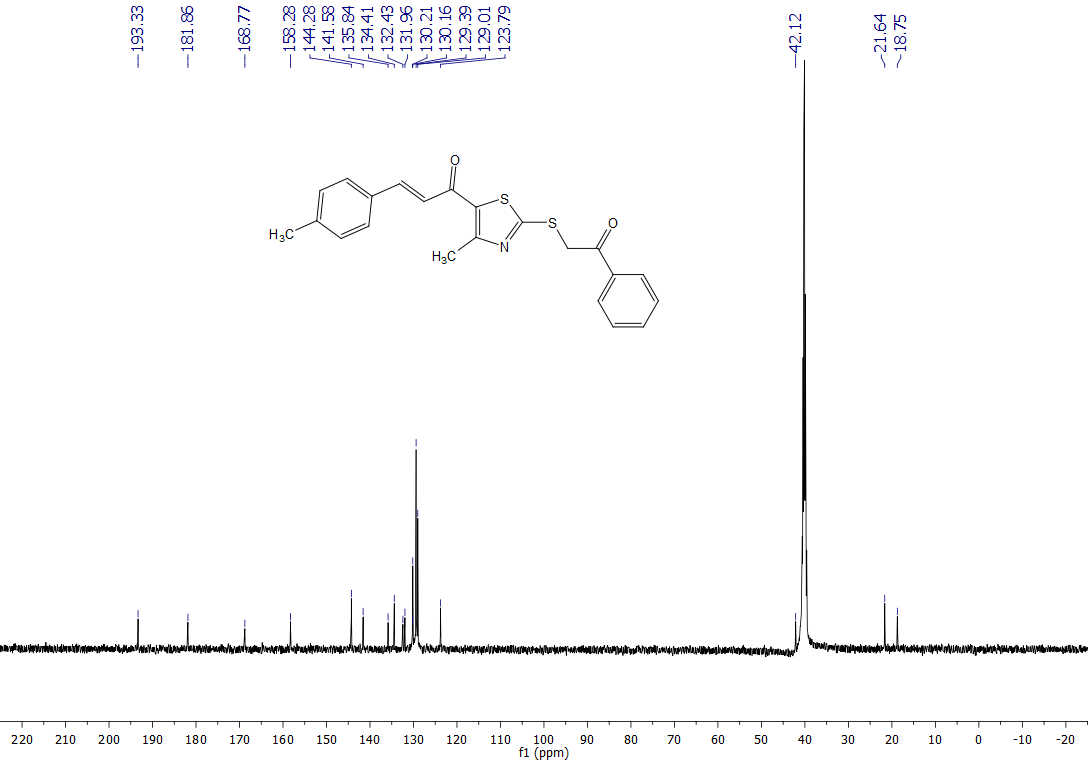


**Figure S10.** ^13^C NMR spectrum of compound **8e**


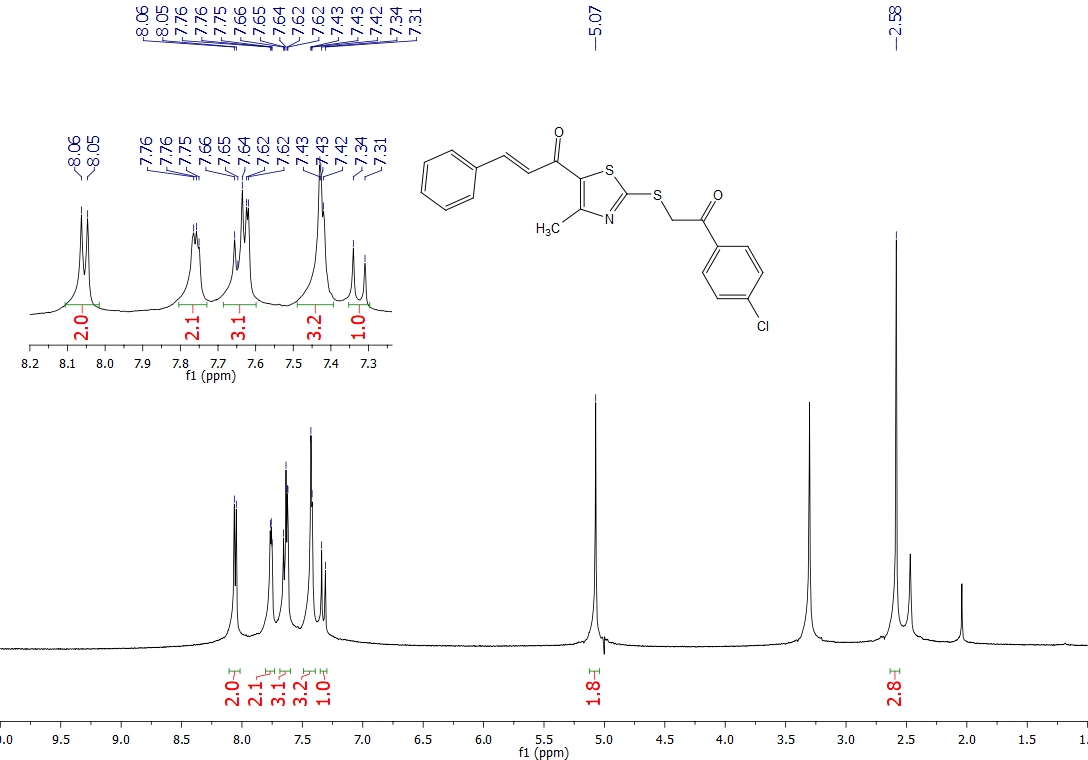


**Figure S11.** ^1^H NMR spectrum of compound **8f**


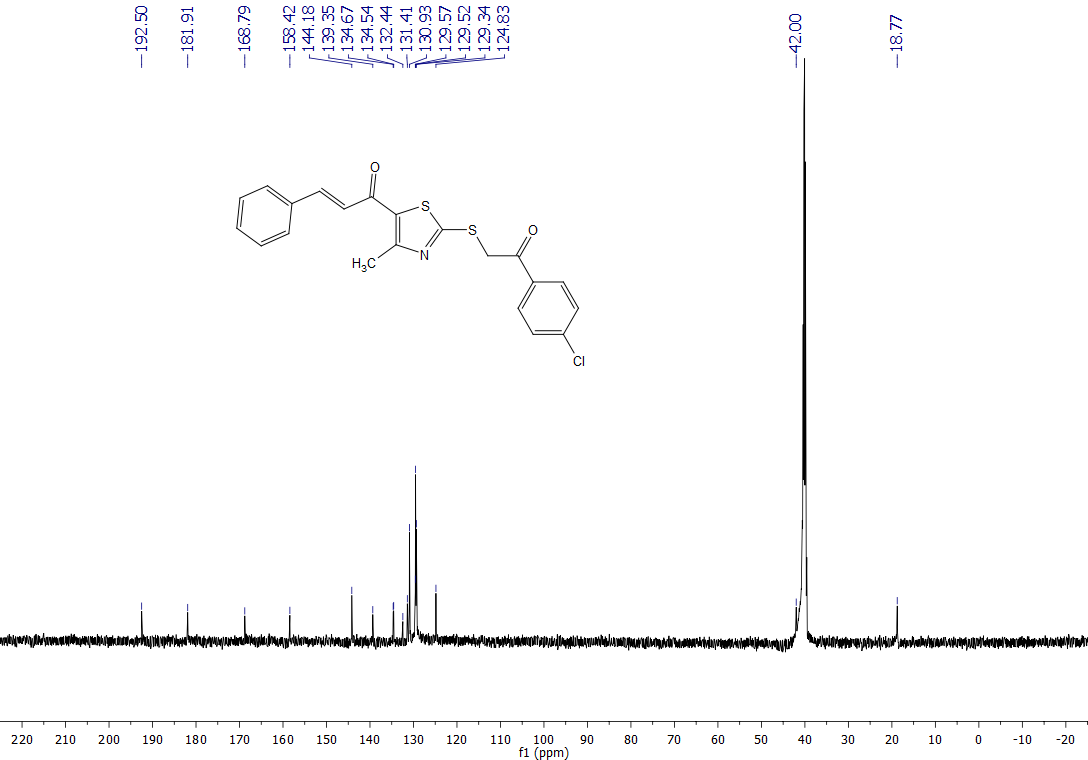


**Figure S12.** ^13^C NMR spectrum of compound **8f**


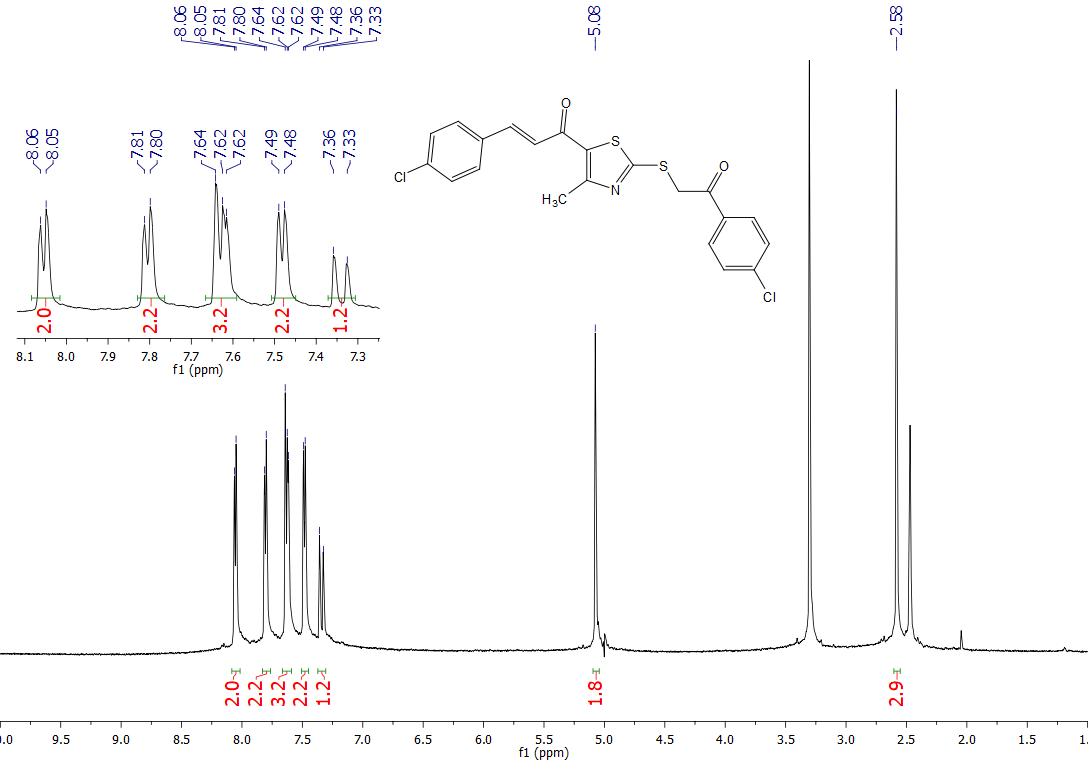


**Figure S13.** ^1^H NMR spectrum of compound **8g**


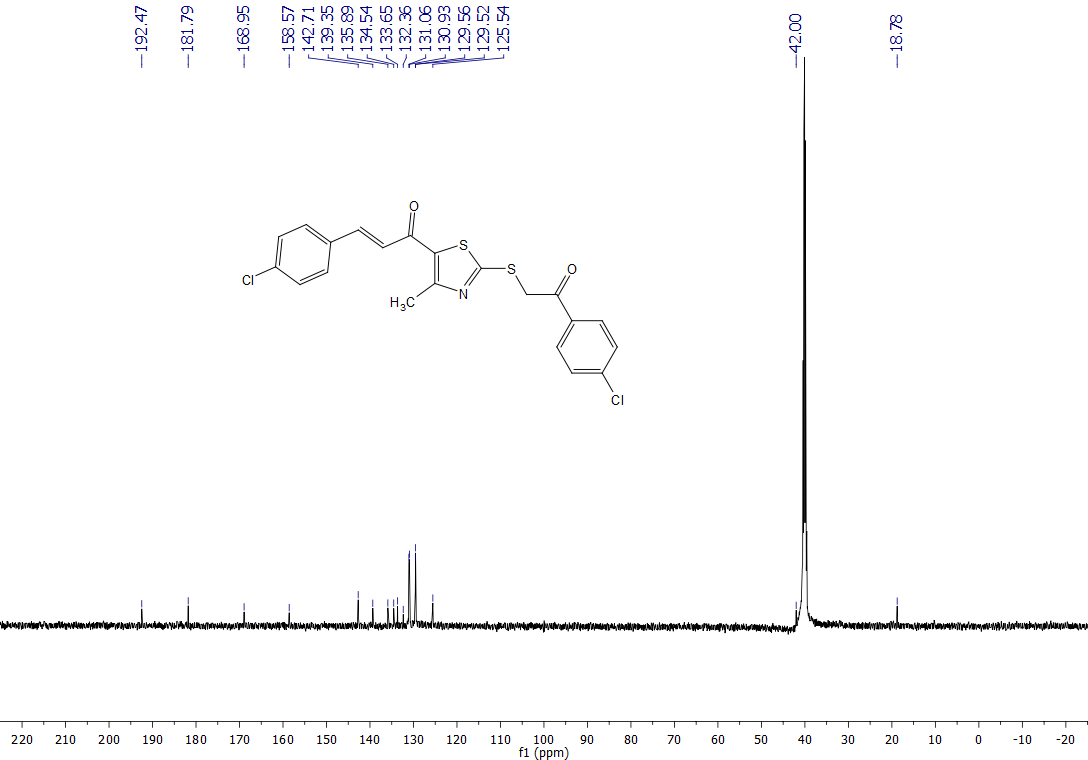


**Figure S14.** ^13^C NMR spectrum of compound **8g**


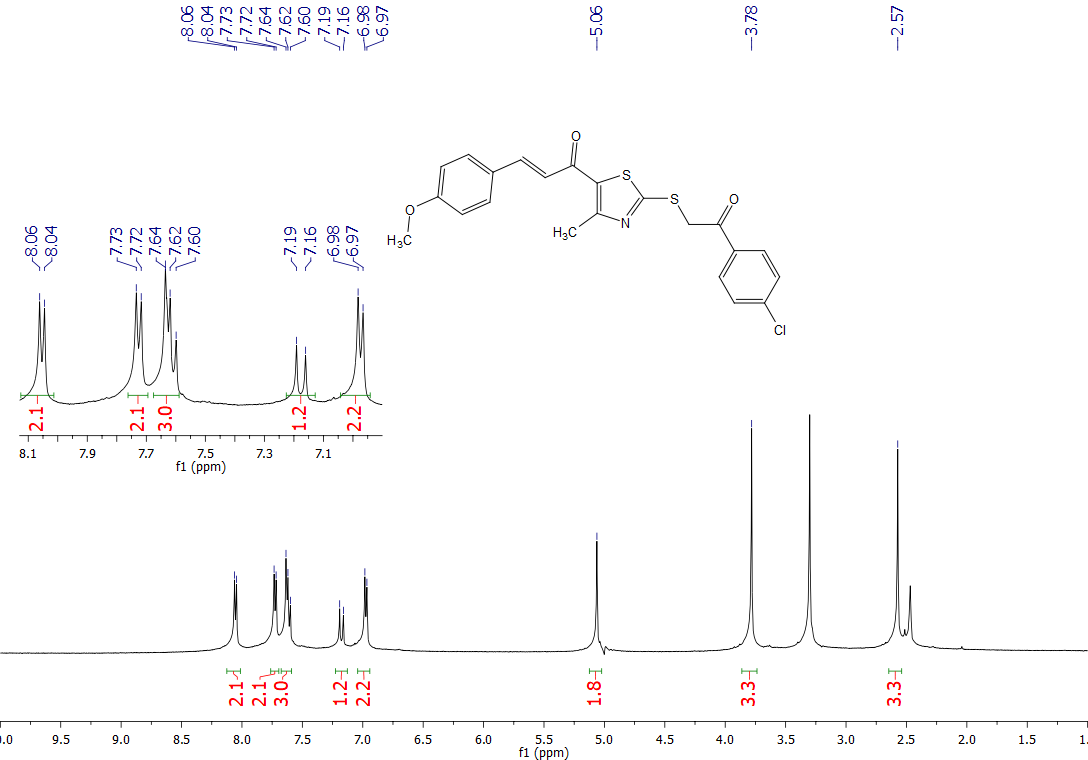


**Figure S15.** ^1^H NMR spectrum of compound **8h**


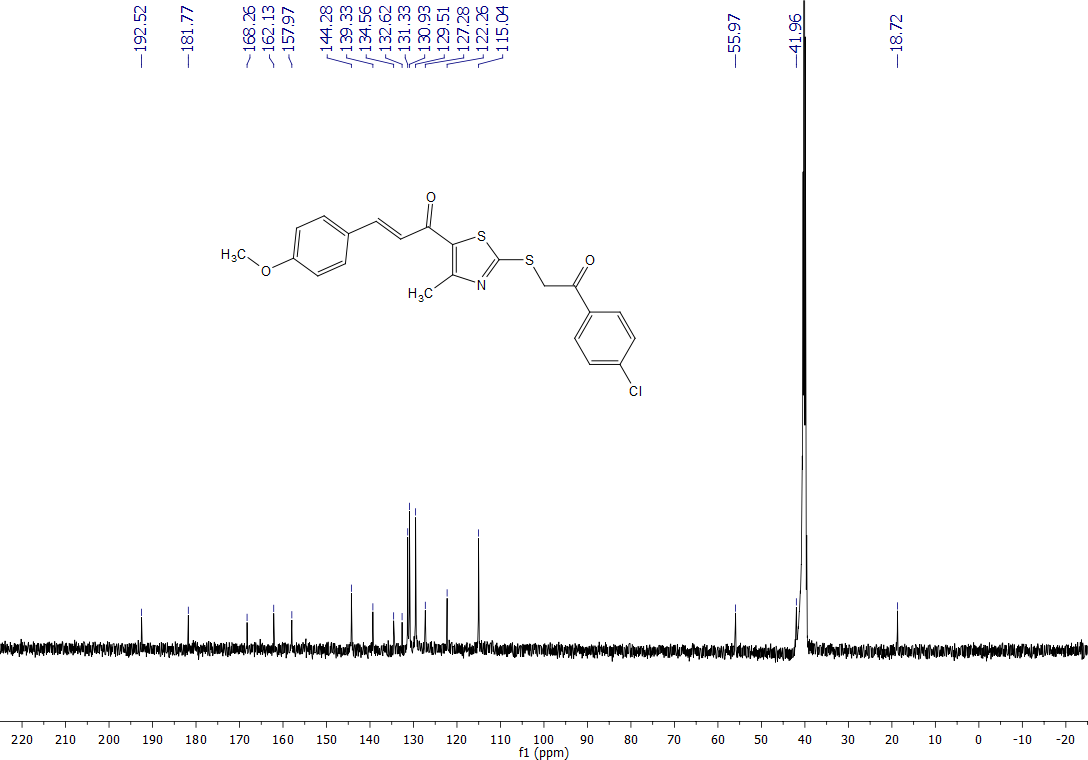


**Figure S16.** ^13^C NMR spectrum of compound **8h**


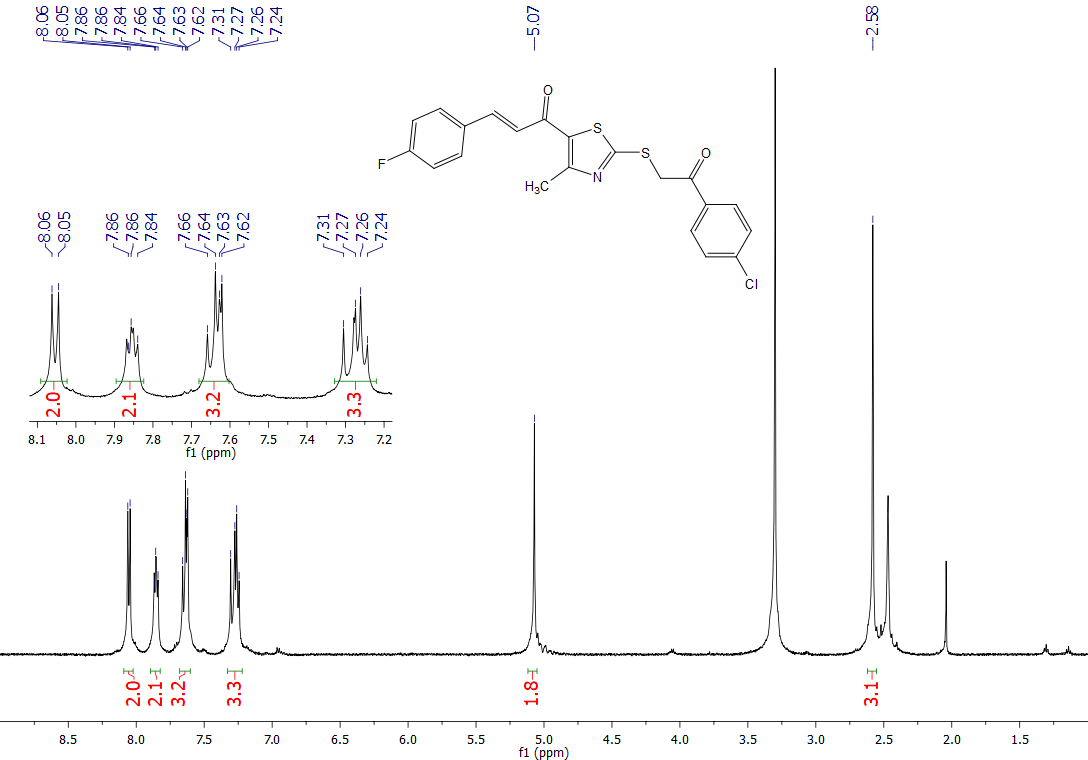


**Figure S17.** ^1^H NMR spectrum of compound **8i**


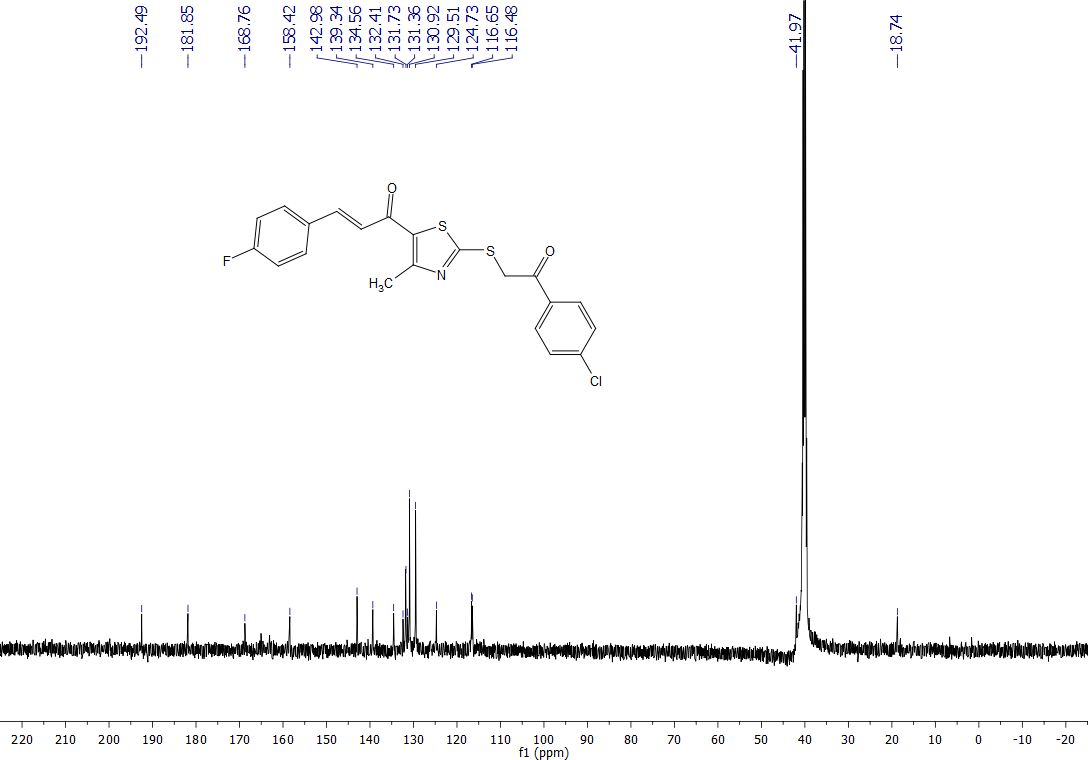


**Figure S18.** ^13^C NMR spectrum of compound **8i**


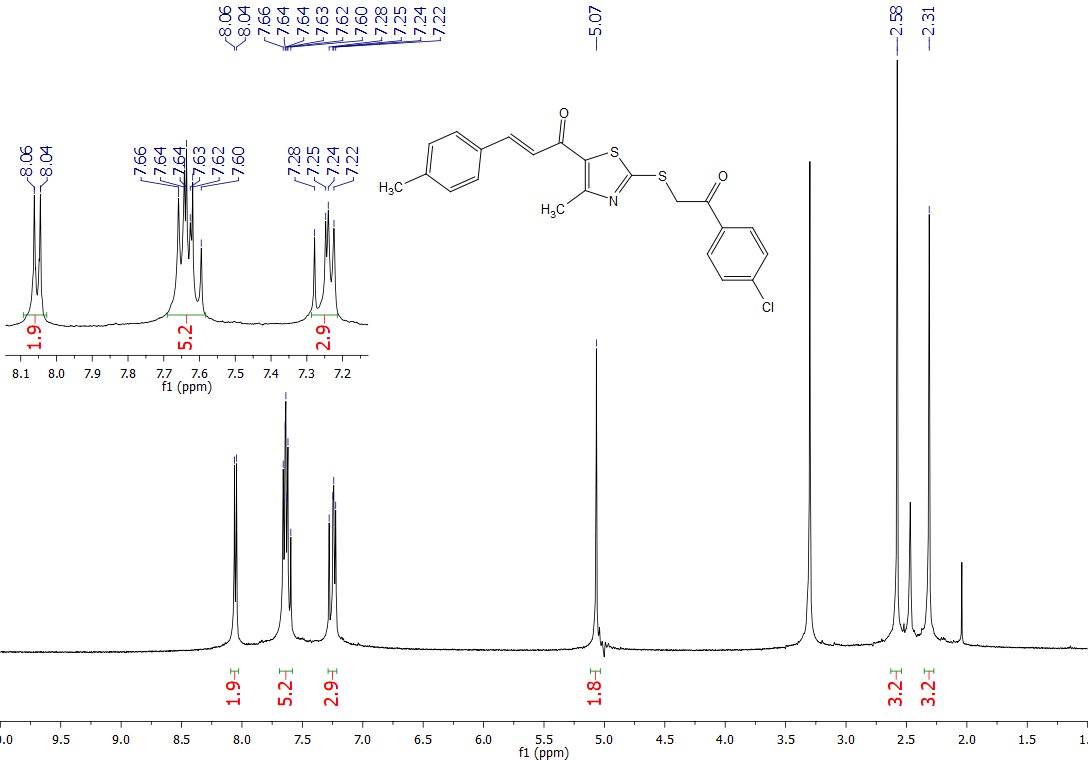


**Figure S19.** ^1^H NMR spectrum of compound **8j**


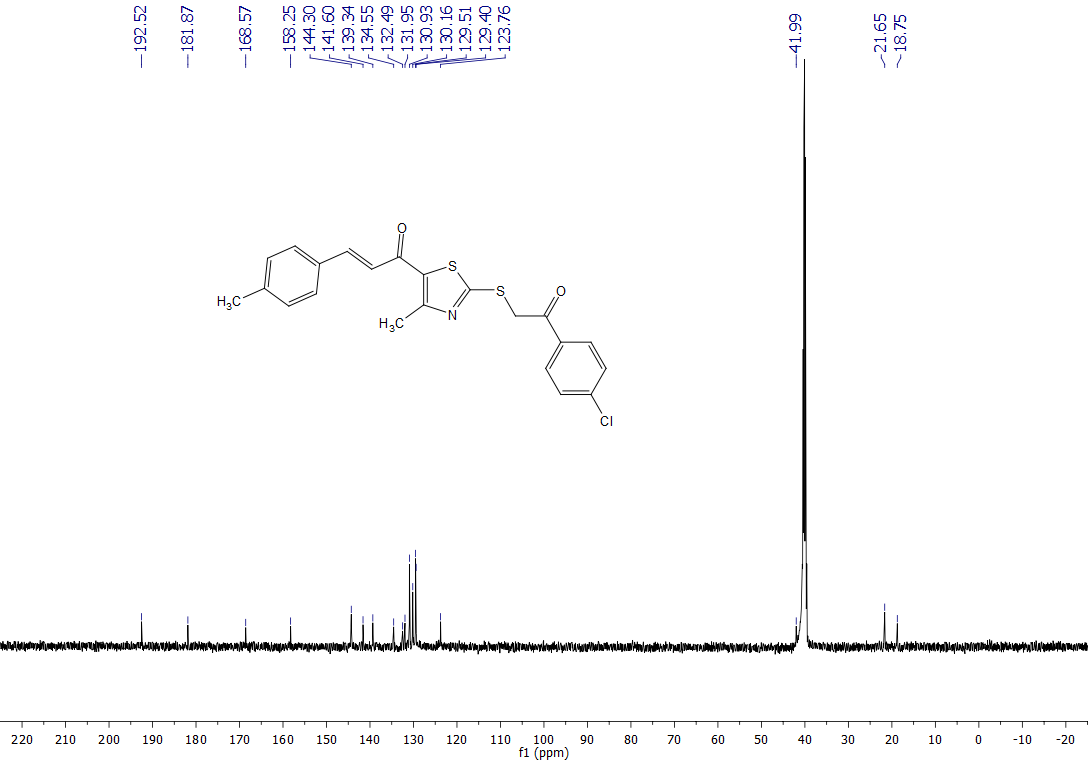


**Figure S20.** ^13^C NMR spectrum of compound **8j**


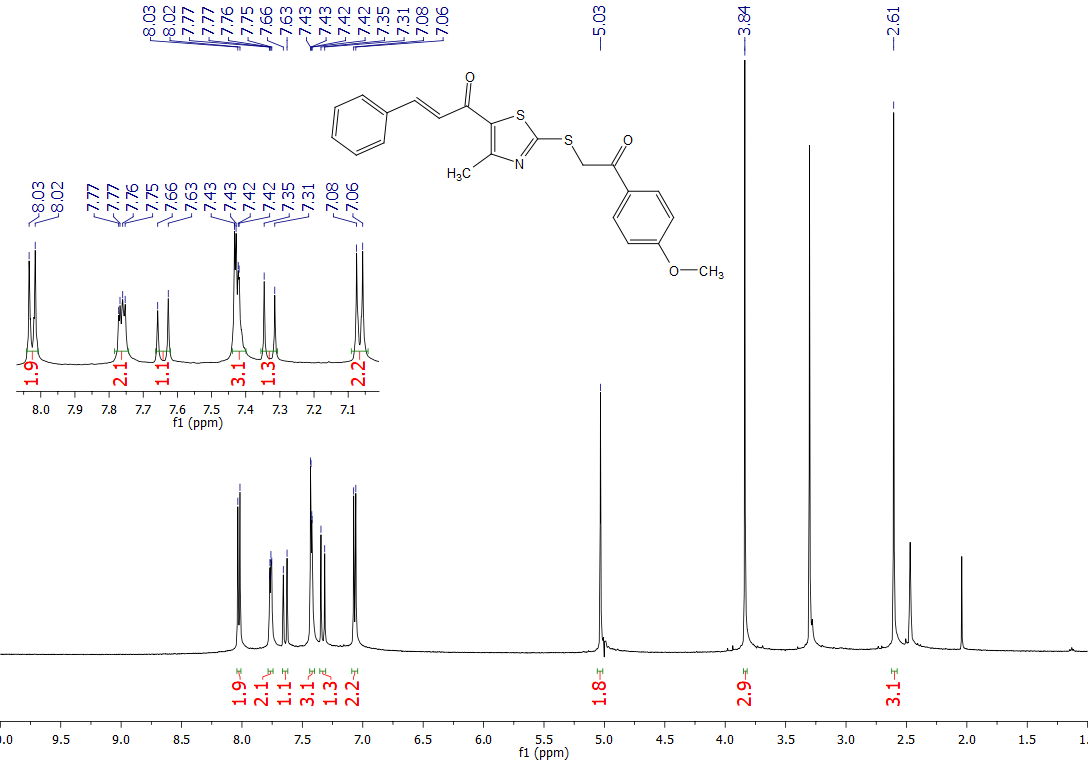


**Figure S21.** ^1^H NMR spectrum of compound **8k**


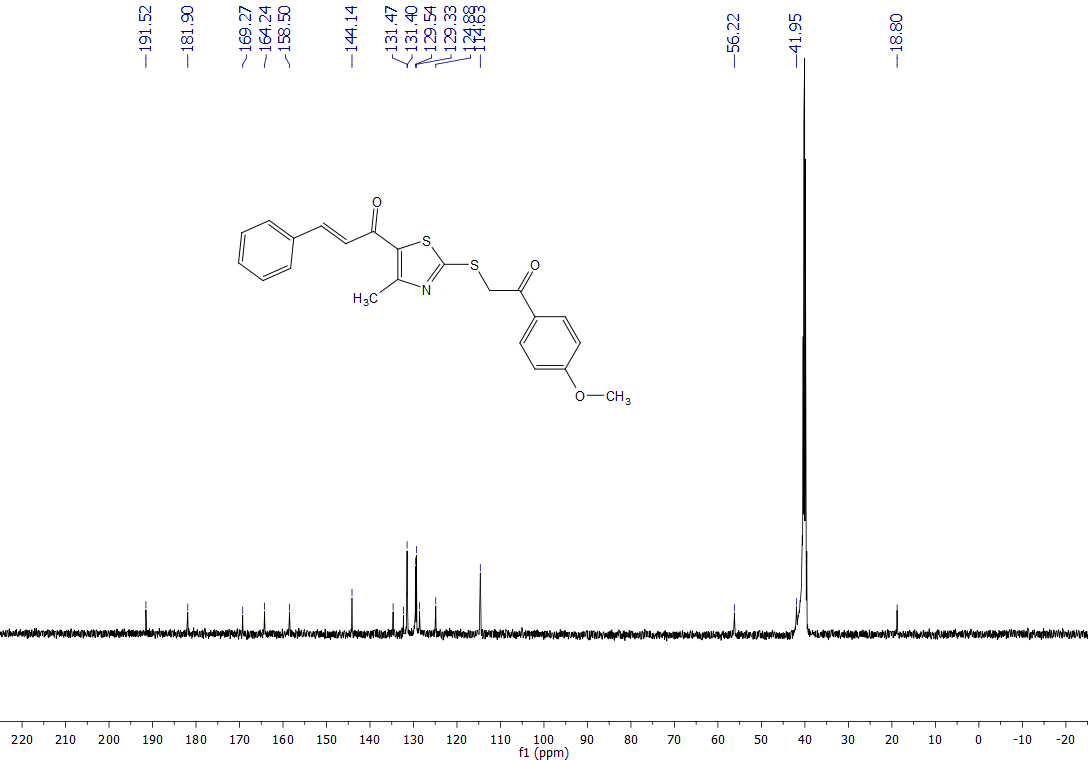


**Figure S22.** ^13^C NMR spectrum of compound **8k**


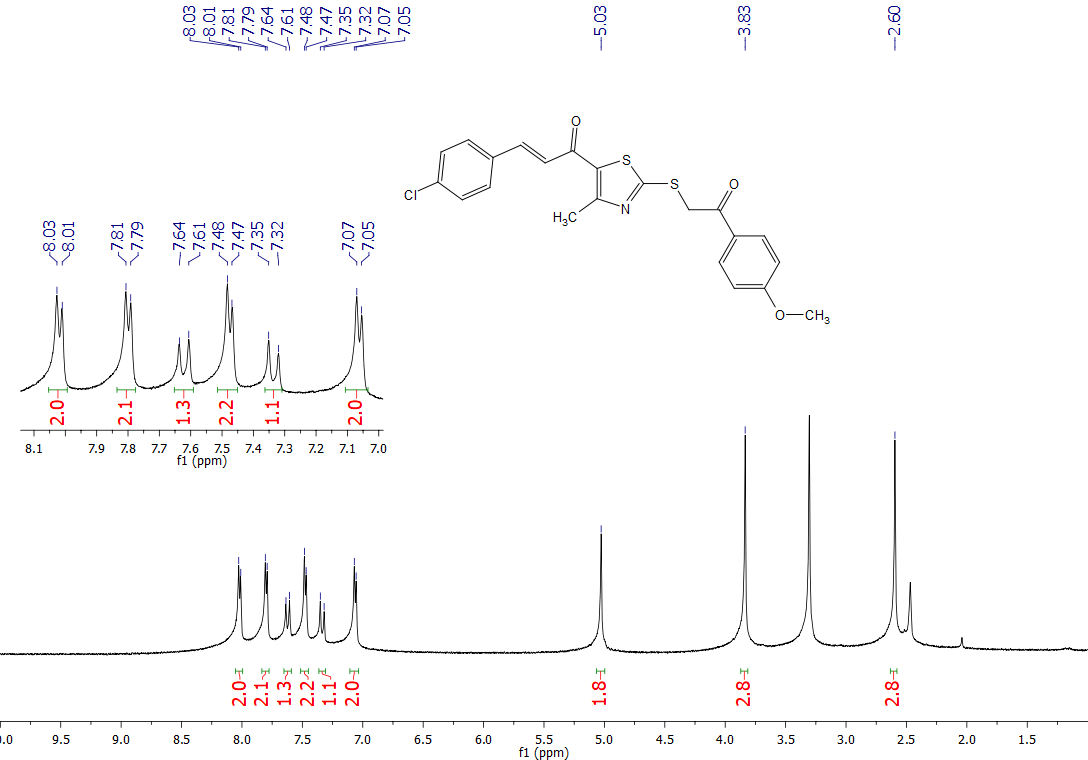


**Figure S23.** ^1^H NMR spectrum of compound **8l**


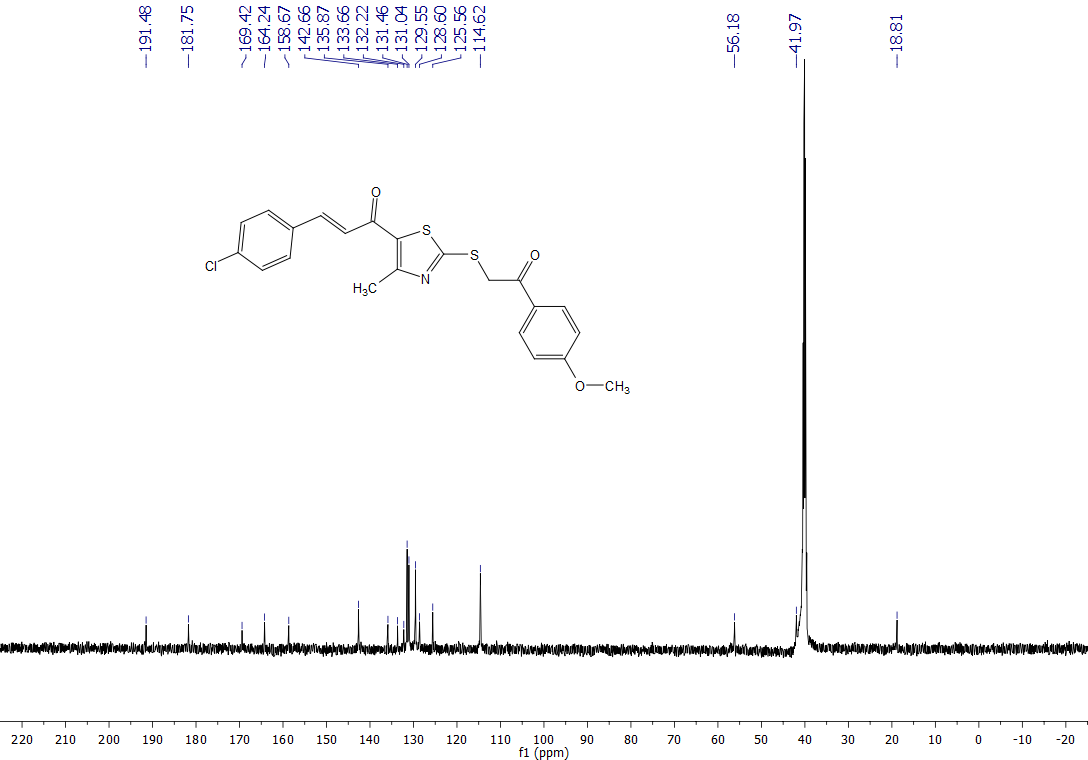


**Figure S24.** ^13^C NMR spectrum of compound **8l**


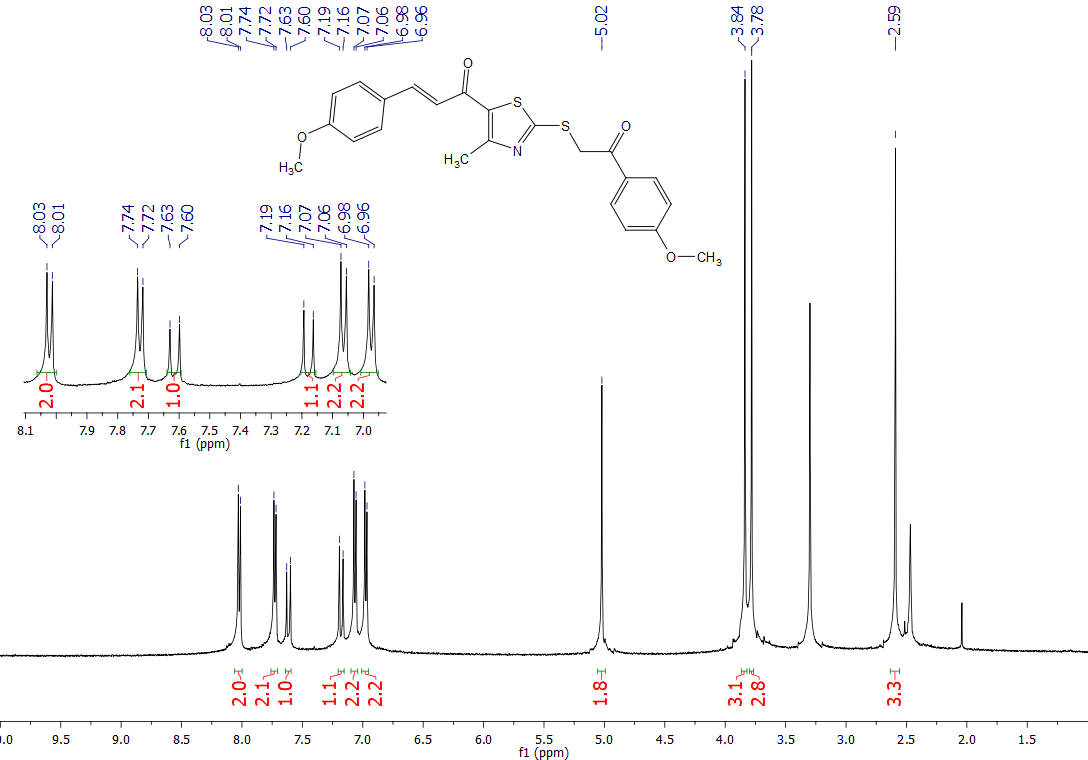


**Figure S25.** ^1^H NMR spectrum of compound **8m**


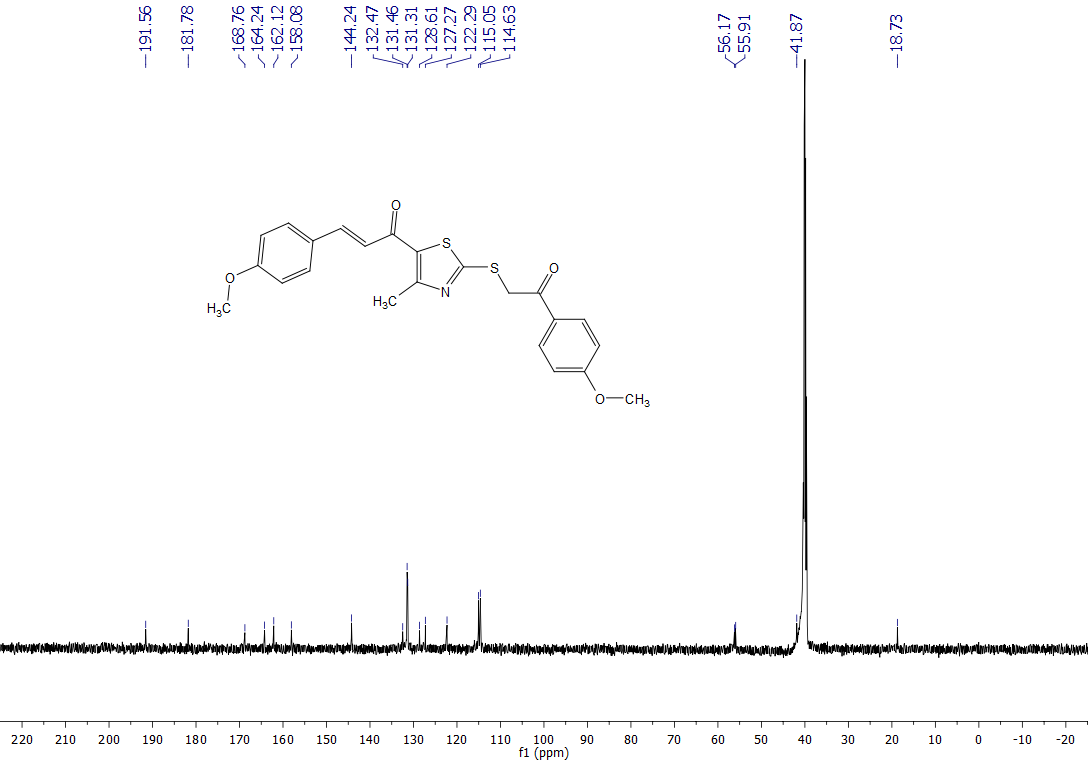


**Figure S26.** ^13^C NMR spectrum of compound **8m**


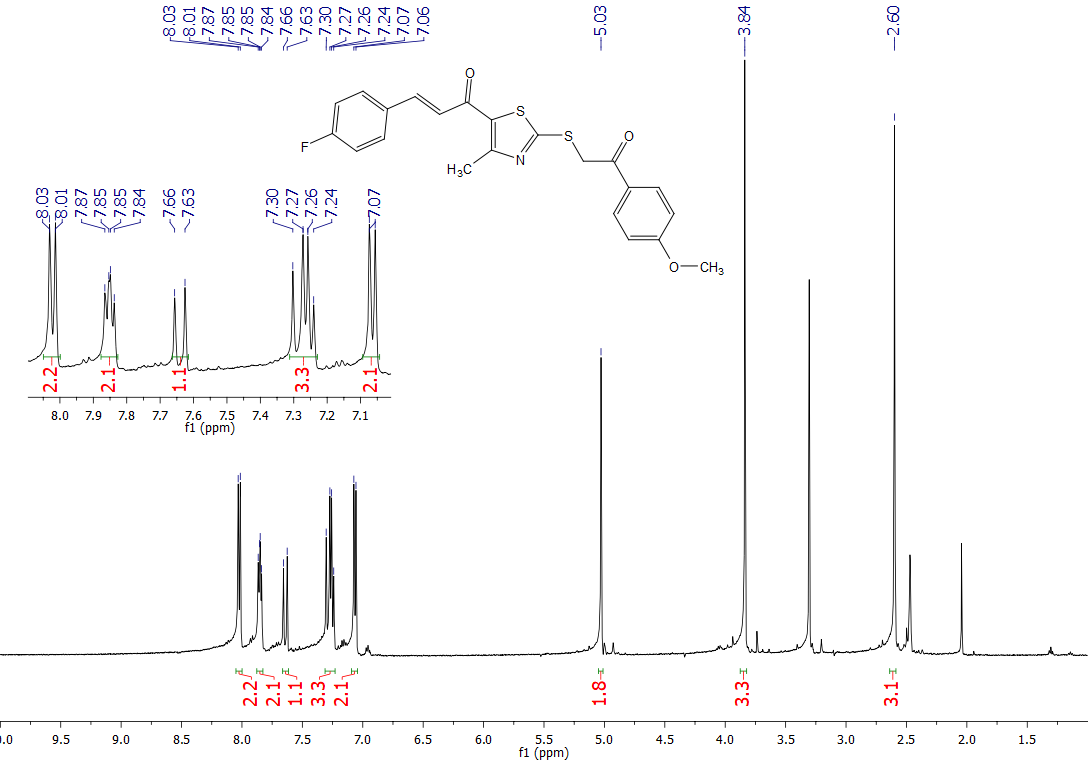


**Figure S27.** ^1^H NMR spectrum of compound **8n**


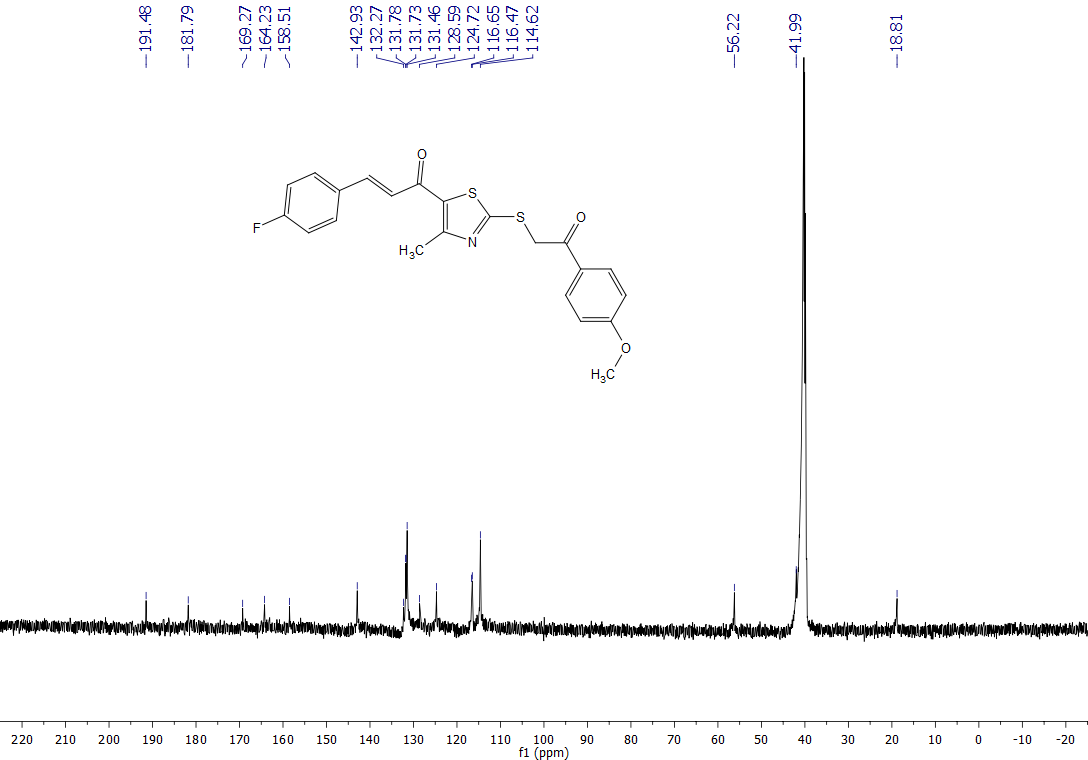


**Figure S28.** ^13^C NMR spectrum of compound **8n**


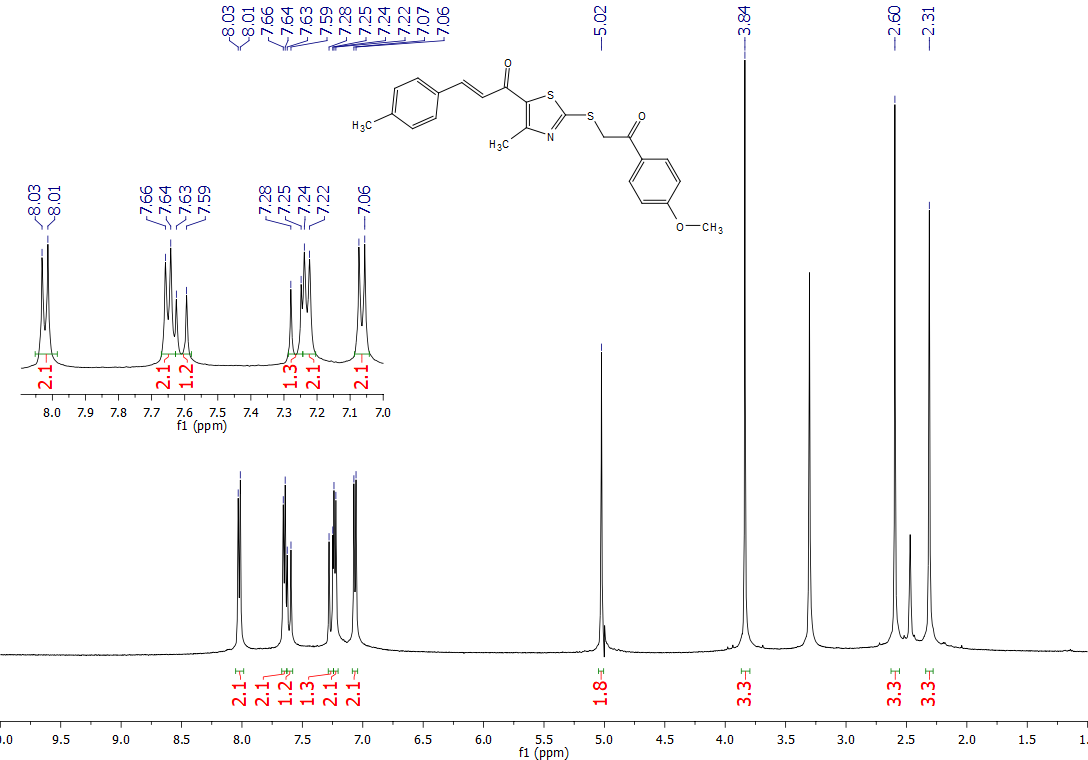


**Figure S29.** ^1^H NMR spectrum of compound **8o**


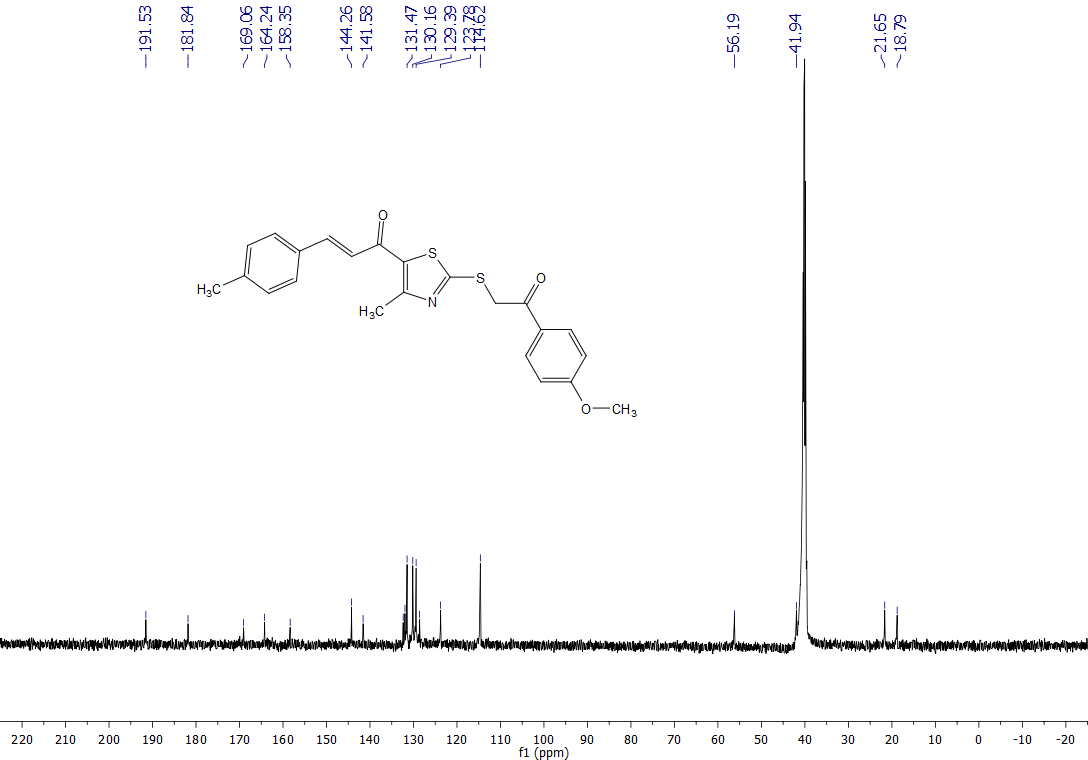


**Figure S30.** ^13^C NMR spectrum of compound **8o**


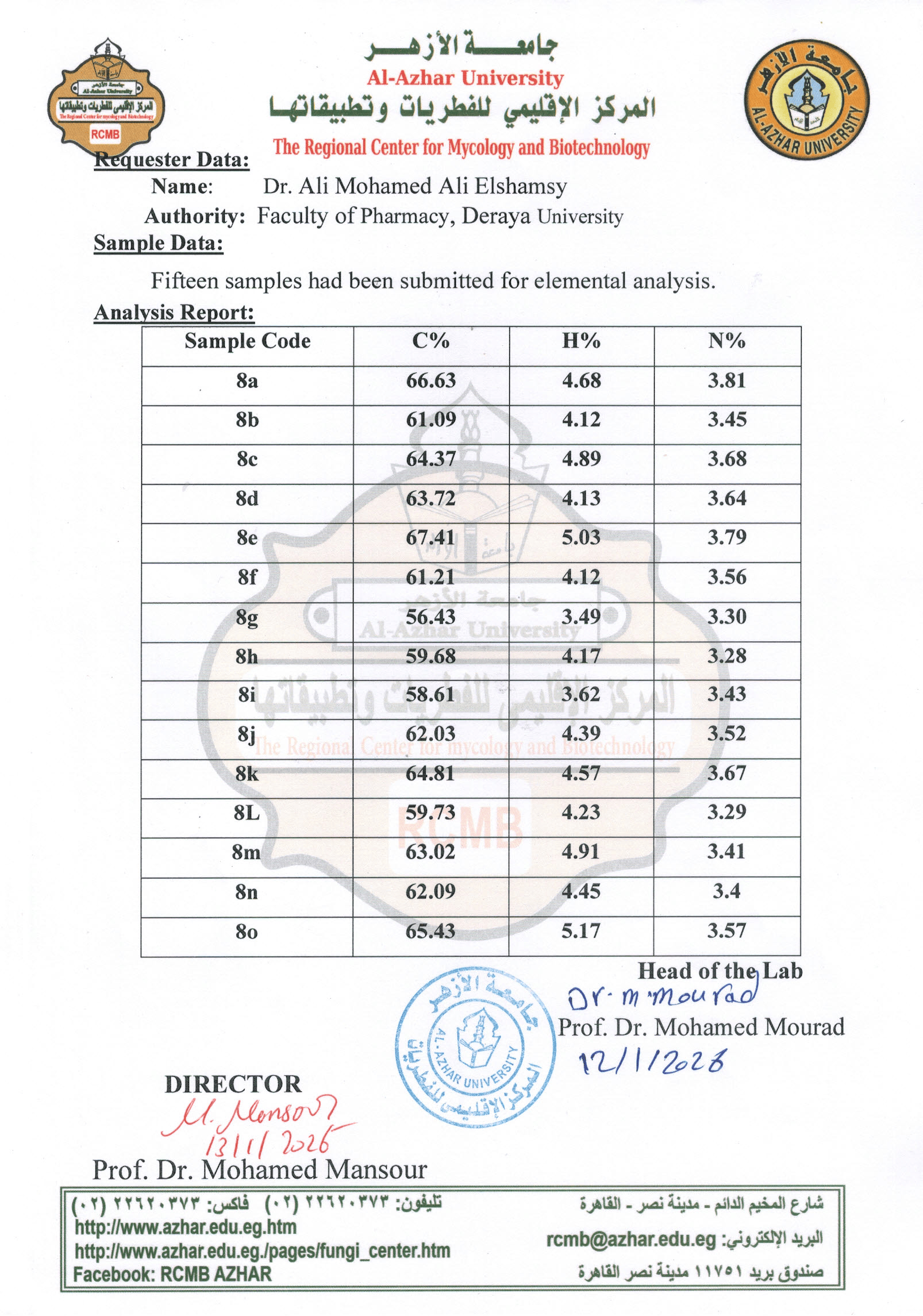


**Figure S31.** Elemental analysis results (C, H, N%) for compounds **8a–8o**

**General Details**

Chemicals and solvents used to prepare the target compounds are of commercial grade and purchased from Alfa Aesar, Sigma Aldrich, and El-Nasr Pharmaceutical Chemical Companies. The reactions progress monitored using precoated TLC plates (Kieselgel 60f254 Merck). The spots were detected by exposure to a UV lamp. Melting points were determined on Stuart's electro-thermal melting point apparatus and were uncorrected. The ¹H NMR spectra were recorded on a JEOL ECA-500 MHz spectrometer in DMSO-d₆ at the National Research Centre, Giza, Egypt. Chemical shifts are expressed as δ (ppm) scale relative to TMS as an internal standard and DMSO-d6 as solvent. Coupling constants (J) in Hz and the signal are designed as follows: s, singlet; d, doublet; t, triplet; q, quartet; m, multiplet. ¹³C NMR spectra were recorded on a JEOL ECA-100 MHz spectrometer in DMSO-d₆ at the National Research Centre, Giza, Egypt.; chemical shift (δ) in ppm relative to TMS (δ=0 ppm) as internal standard and DMSO as solvent. Elemental microanalyses were performed on elemental analyzer model flash 2000 thermo fisher at the regional center for mycology and biotechnology (RCMB), faculty of science, Al-Azhar university, Nasr city, Cairo, Egypt.


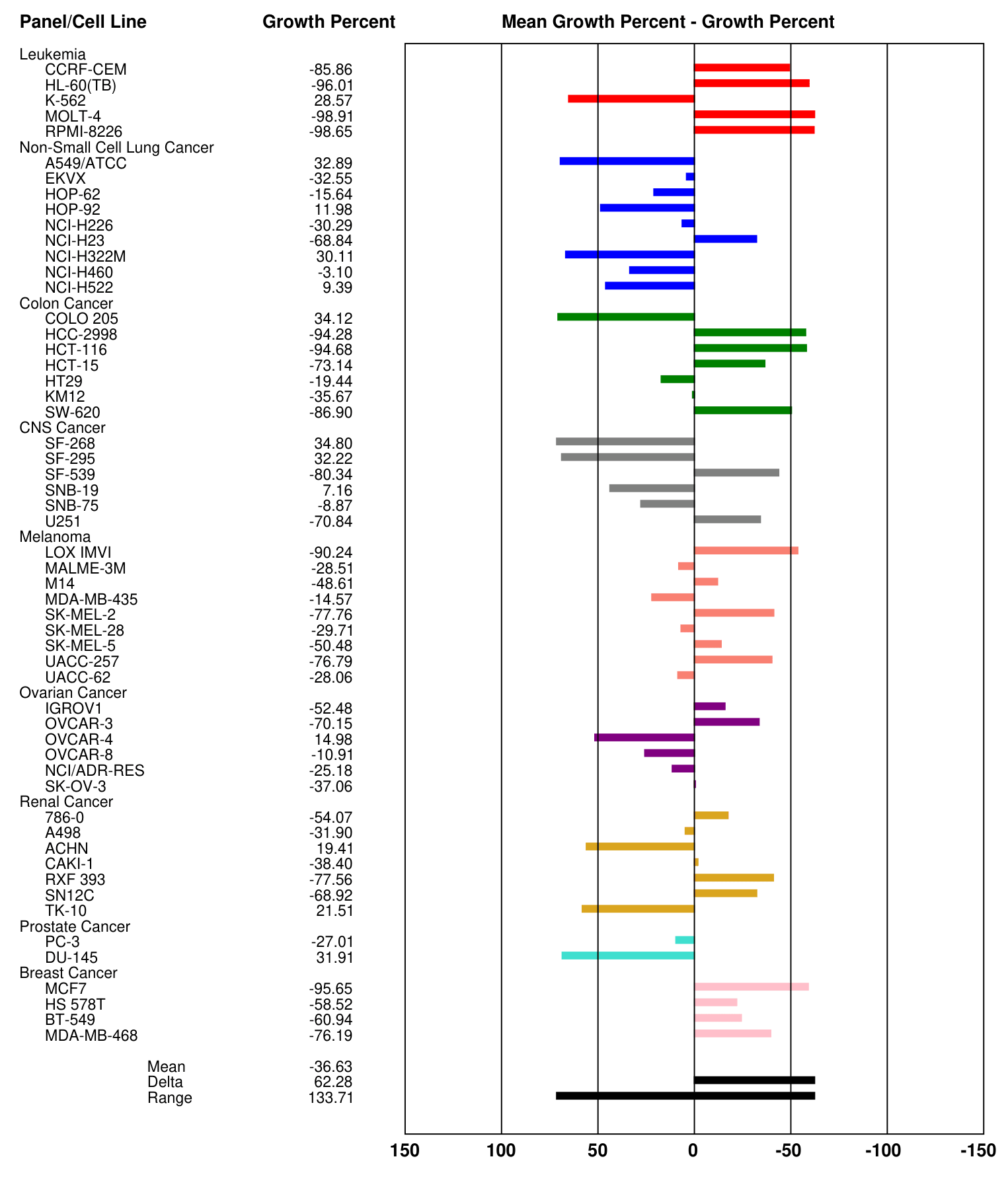


**Figure S32.** One-dose growth (%) and mean graph of compound **8a**


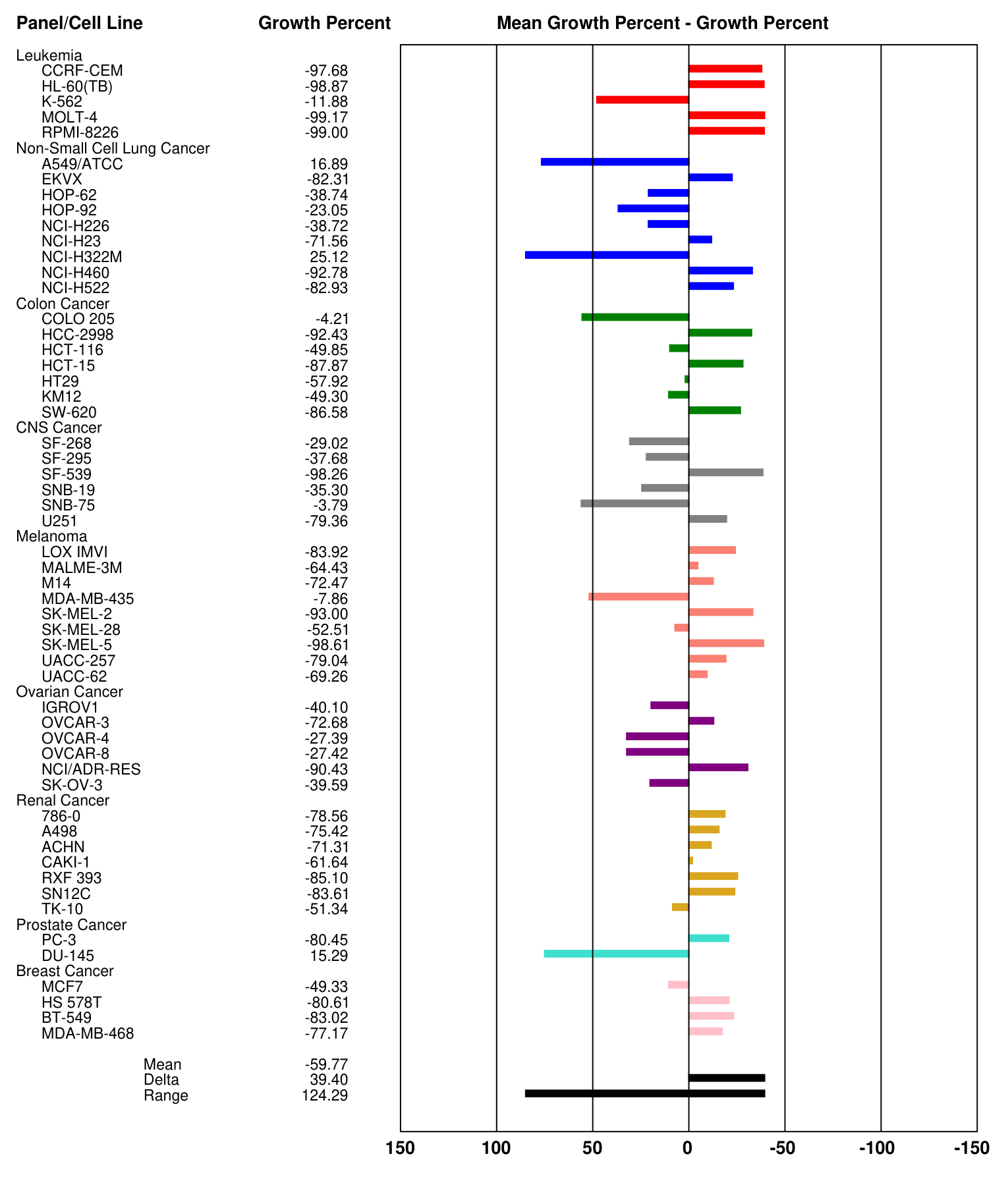


**Figure S33.** One-dose growth (%) and mean graph of compound **8b**


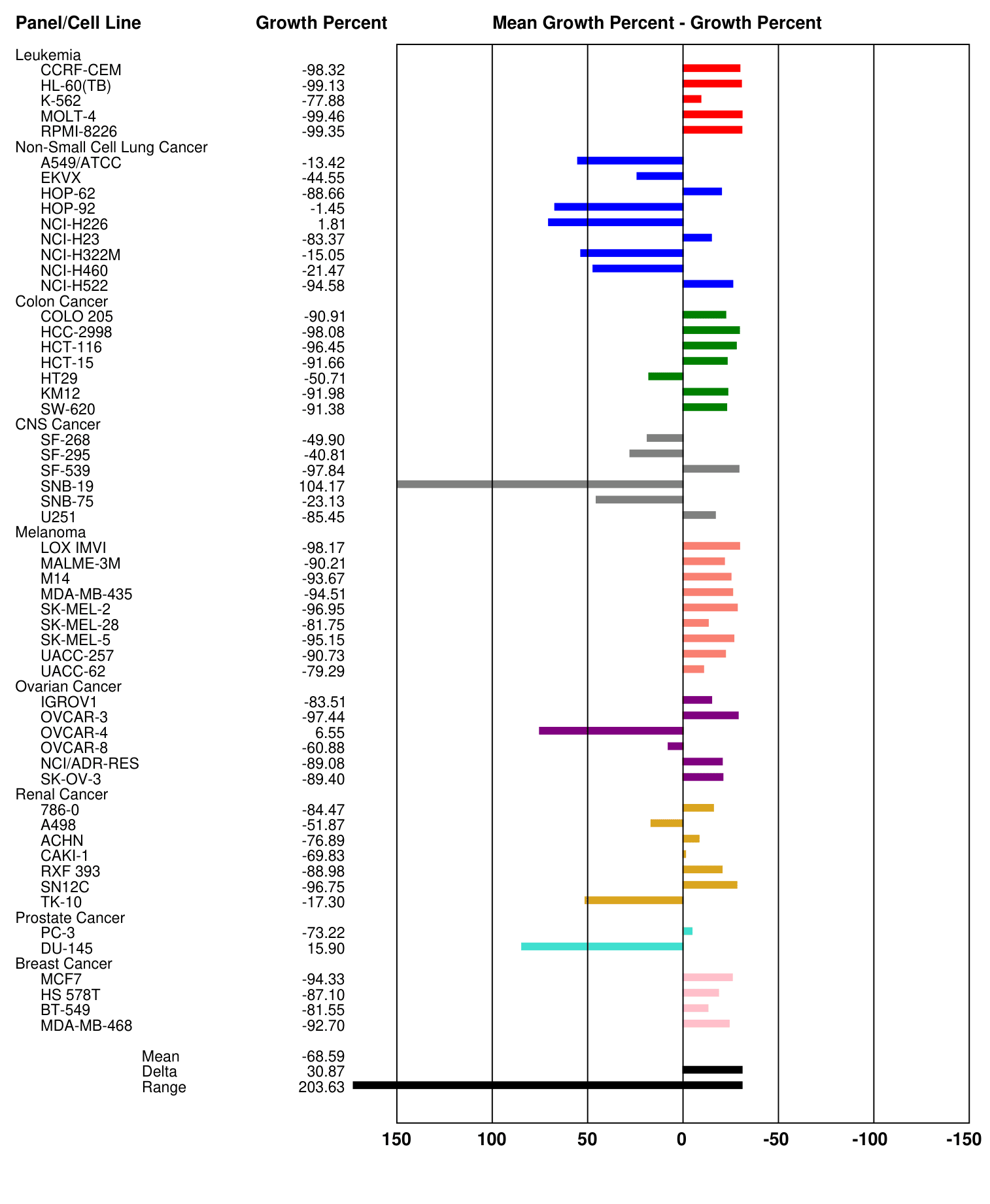


**Figure S34.** One-dose growth (%) and mean graph of compound **8c**


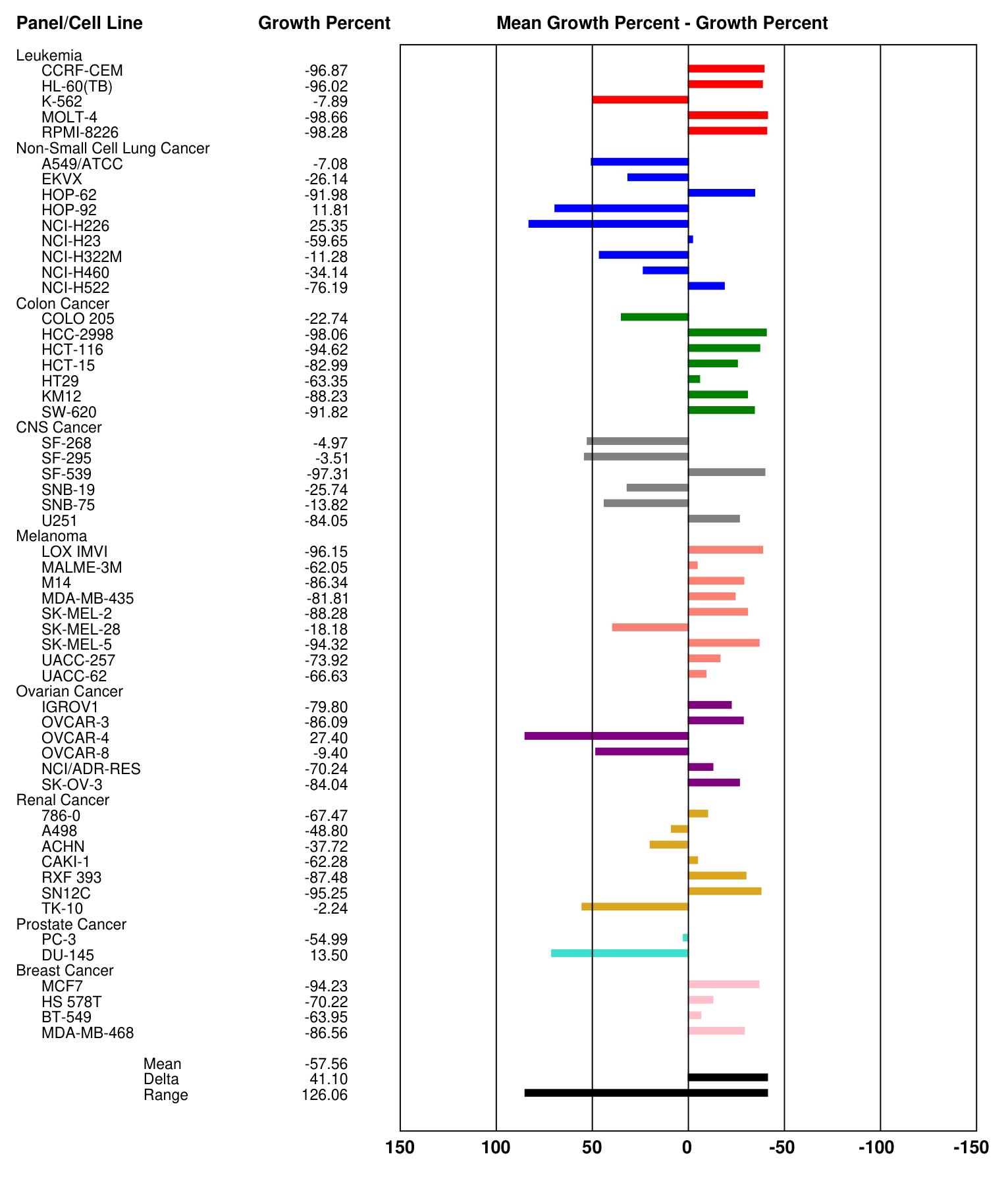


**Figure S35.** One-dose growth (%) and mean graph of compound **8d**


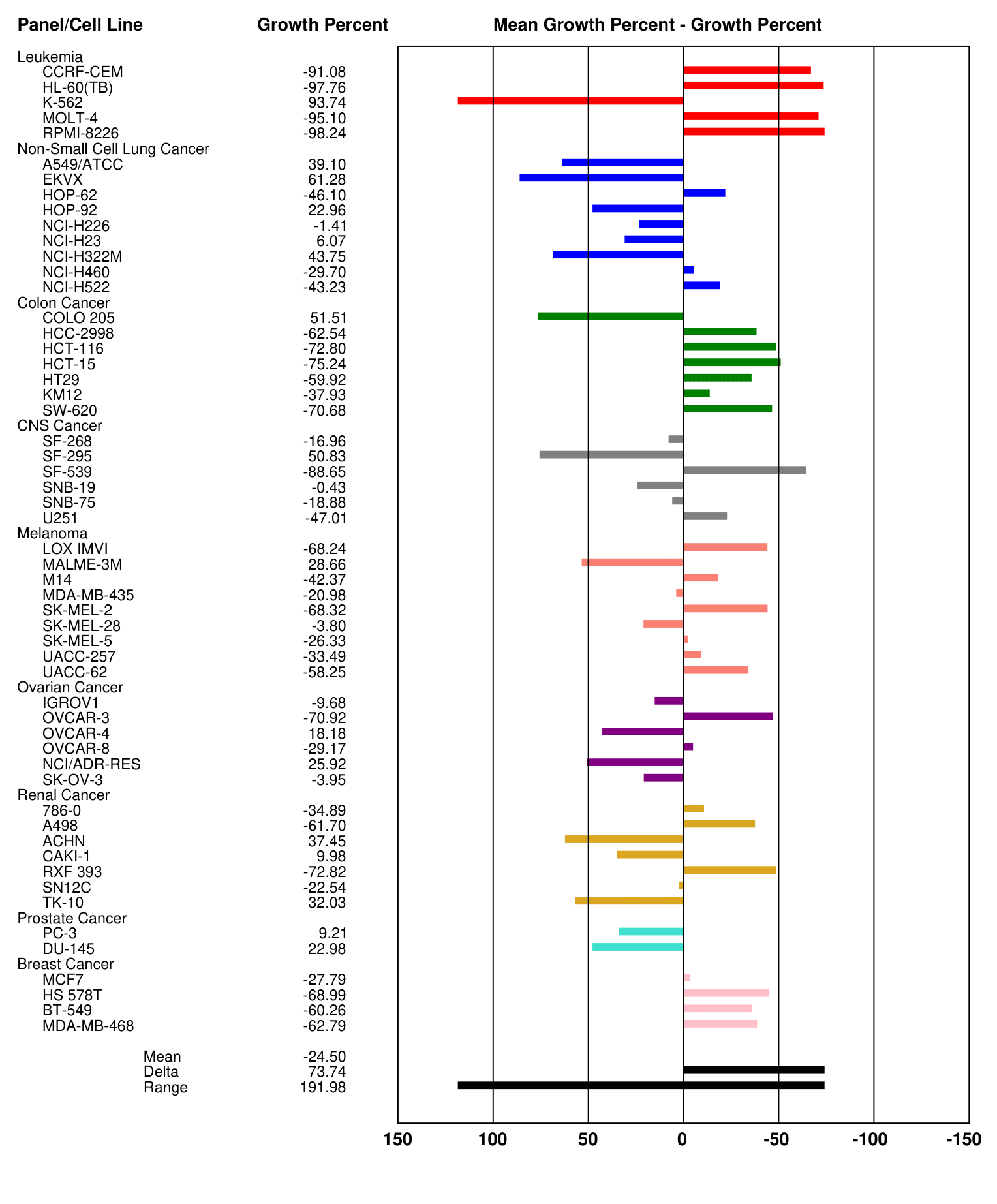


**Figure S36.** One-dose growth (%) and mean graph of compound **8e**


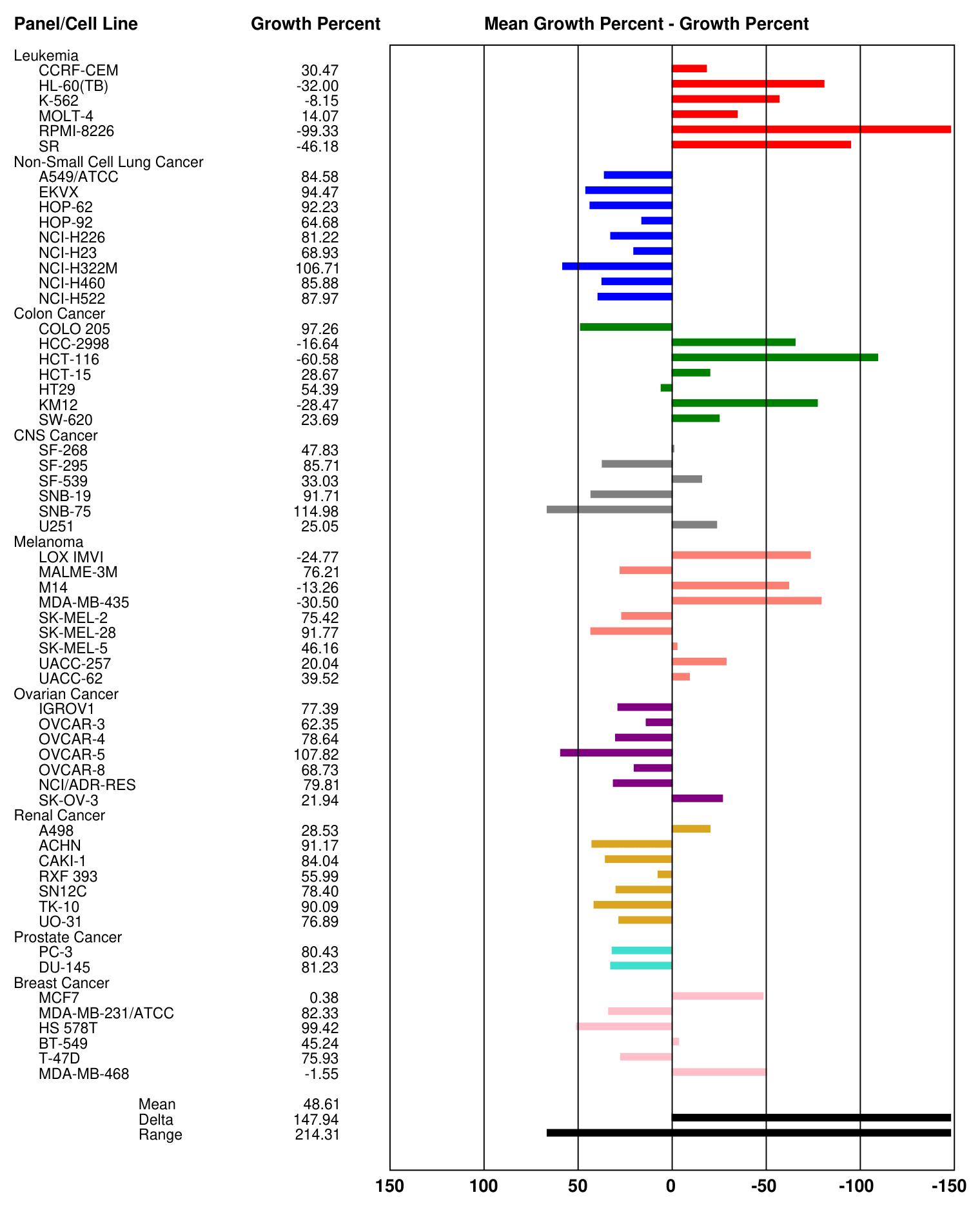


**Figure S37.** One-dose growth (%) and mean graph of compound **8f**


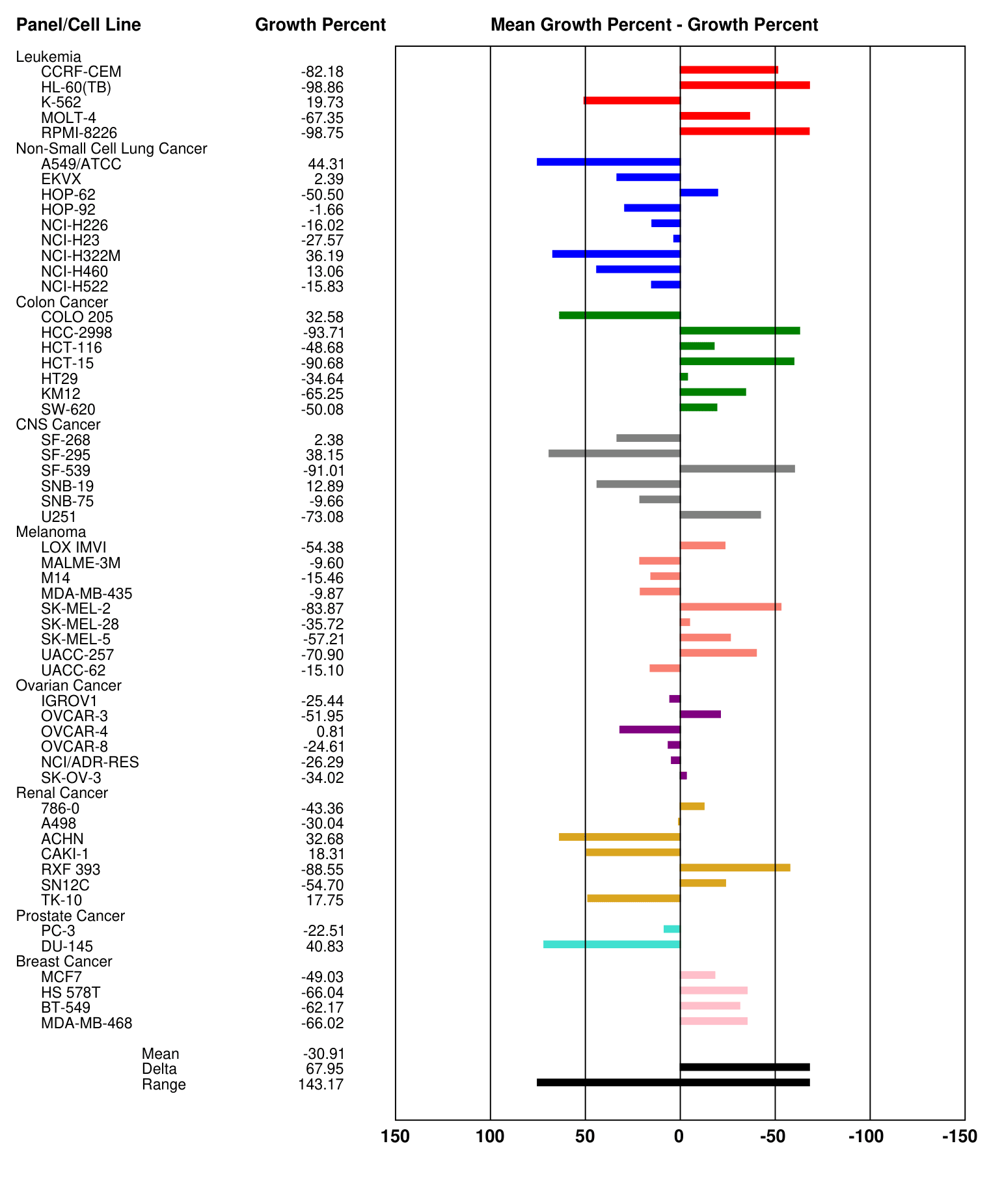


**Figure S38.** One-dose growth (%) and mean graph of compound **8g**


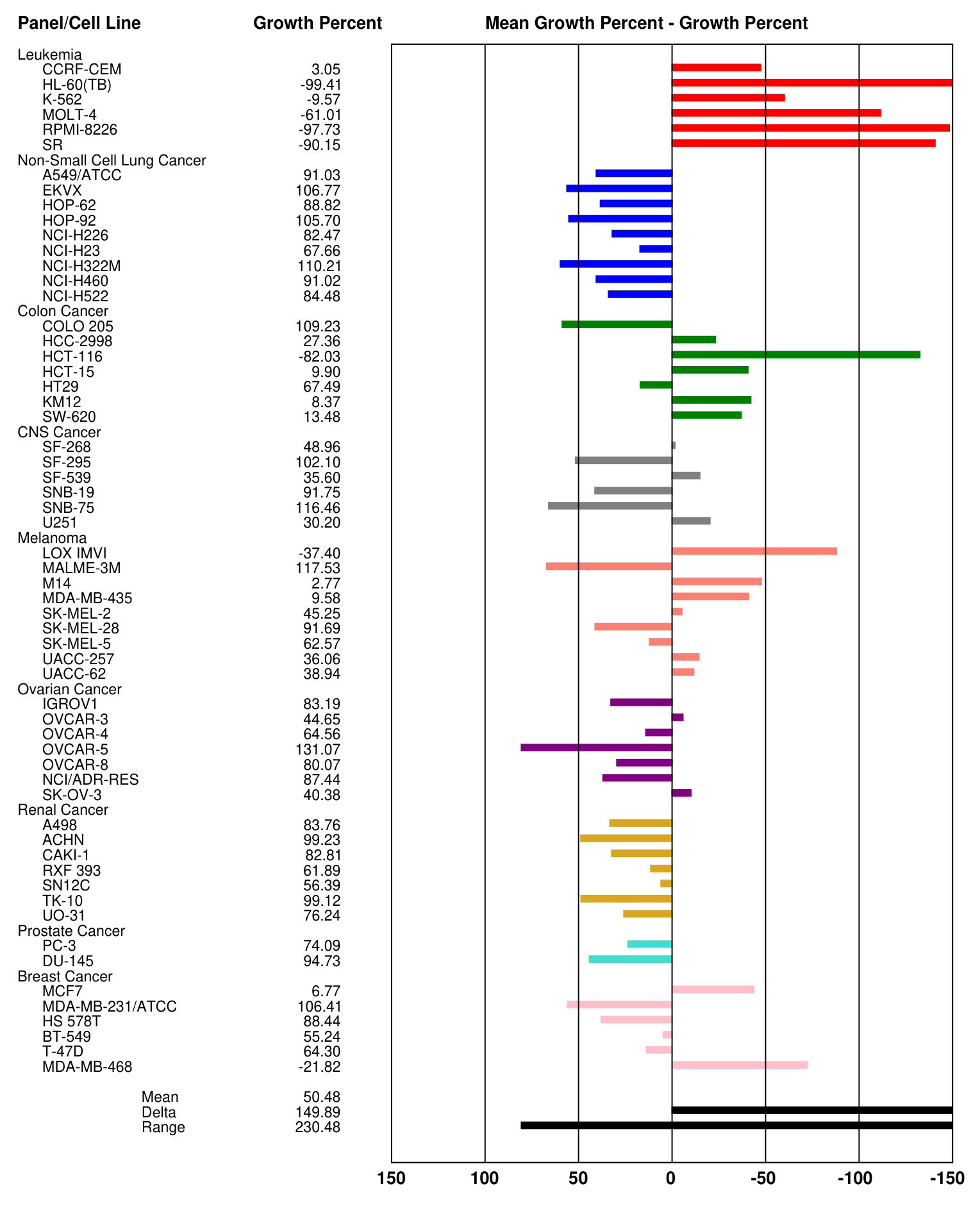


**Figure S39.** One-dose growth (%) and mean graph of compound **8h**


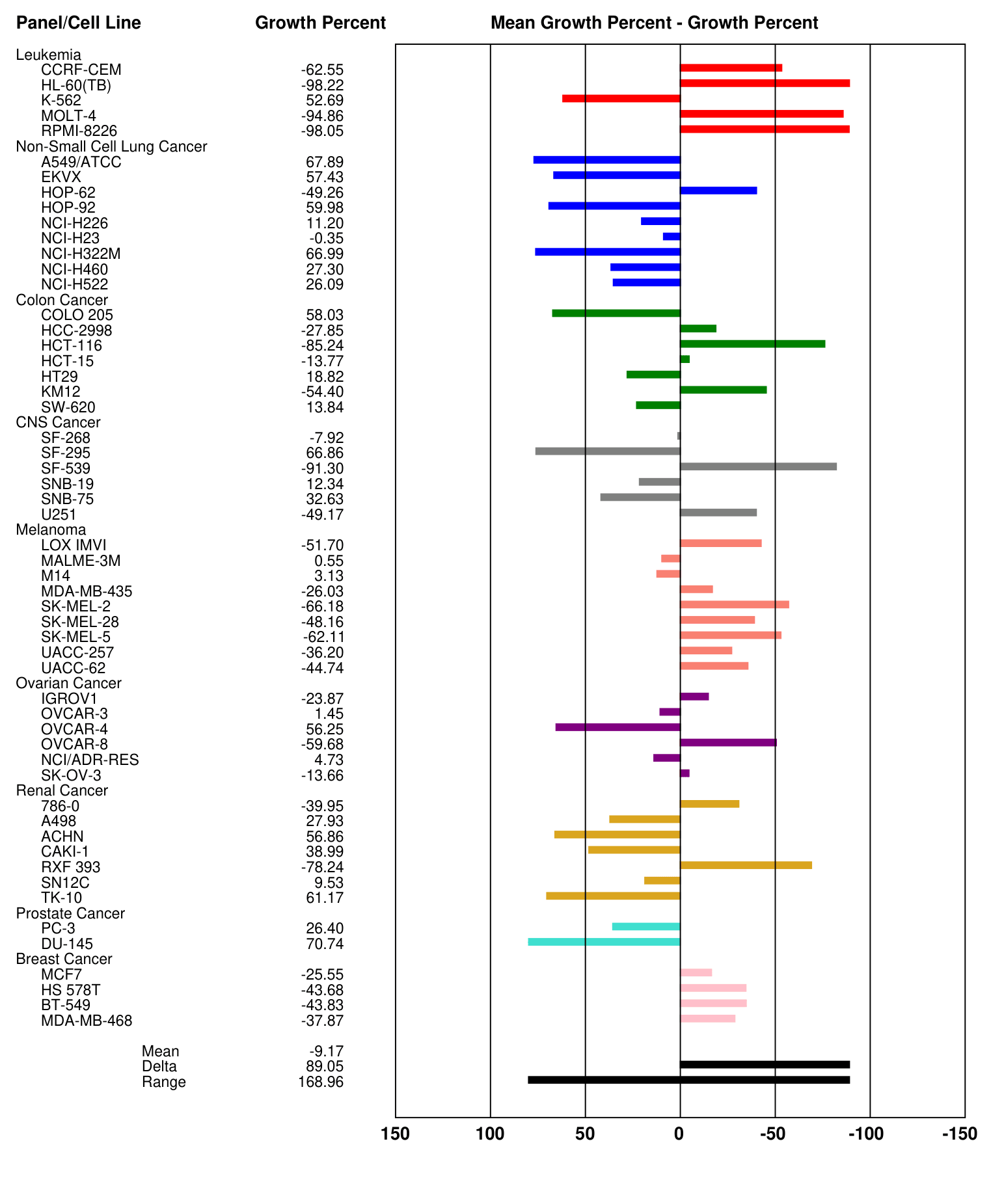


**Figure S40.** One-dose growth (%) and mean graph of compound **8i**


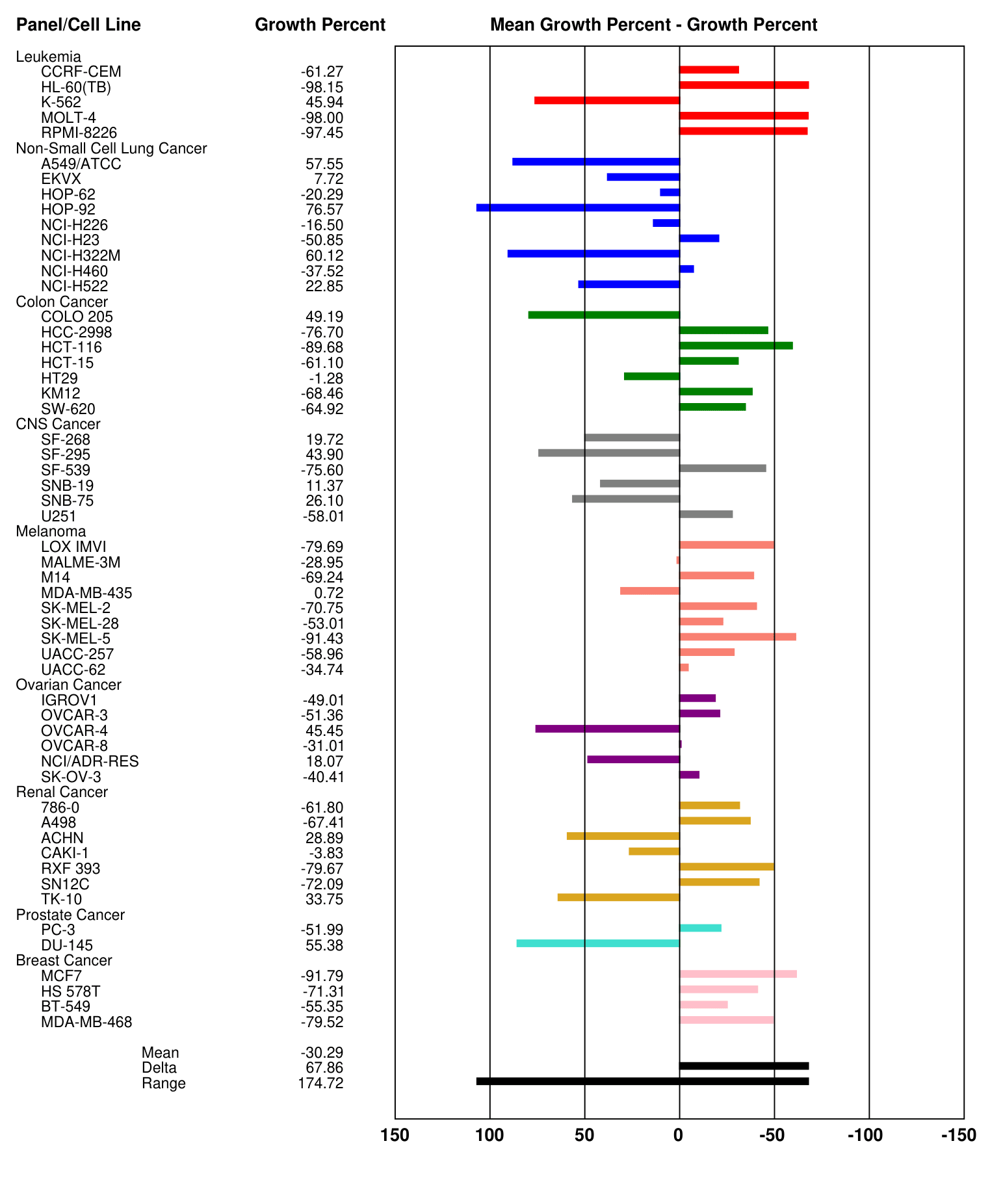


**Figure S41.** One-dose growth (%) and mean graph of compound **8j**


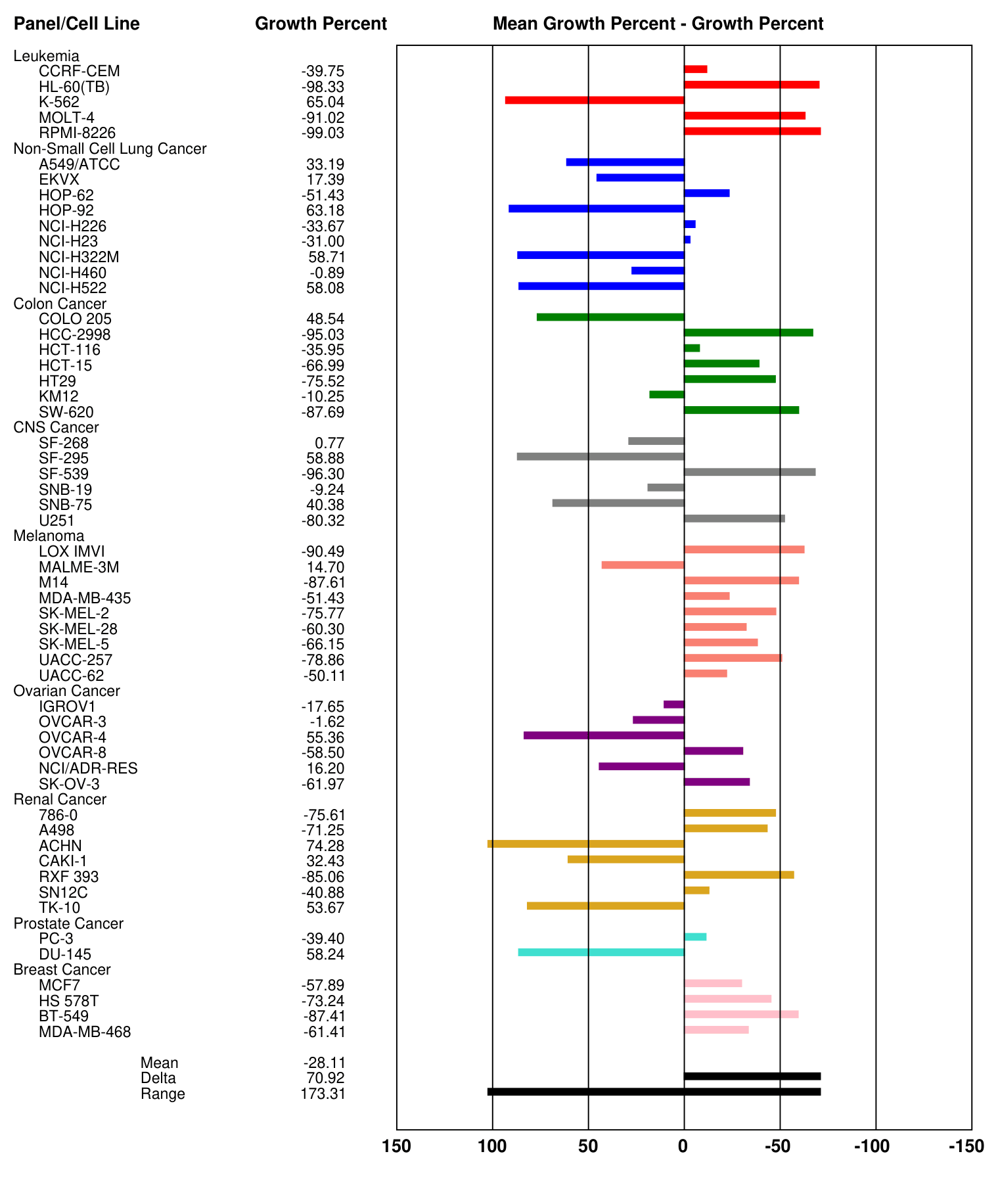


**Figure S42.** One-dose growth (%) and mean graph of compound **8k**


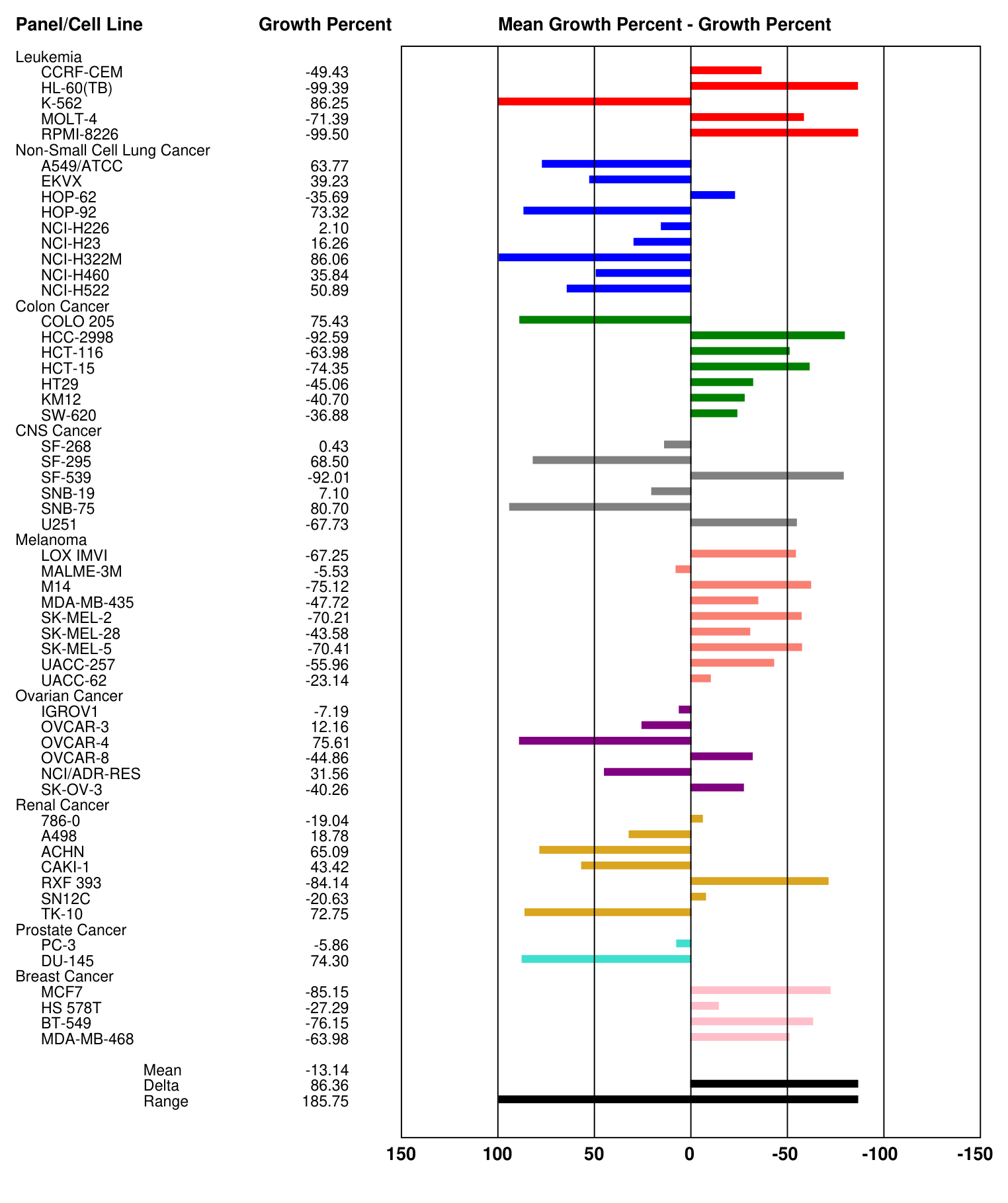


**Figure S43.** One-dose growth (%) and mean graph of compound **8l**


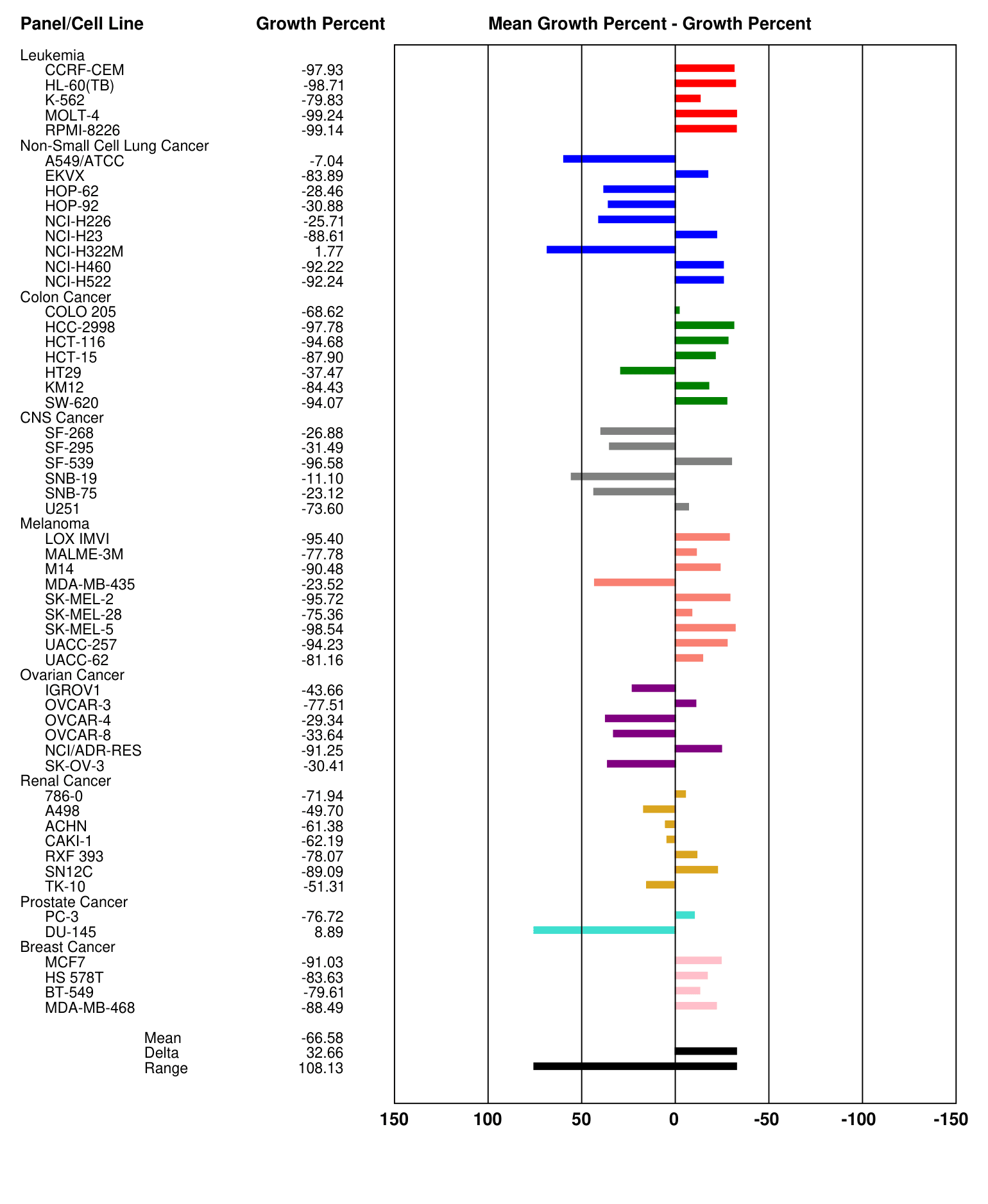


**Figure S44.** One-dose growth (%) and mean graph of compound **8m**


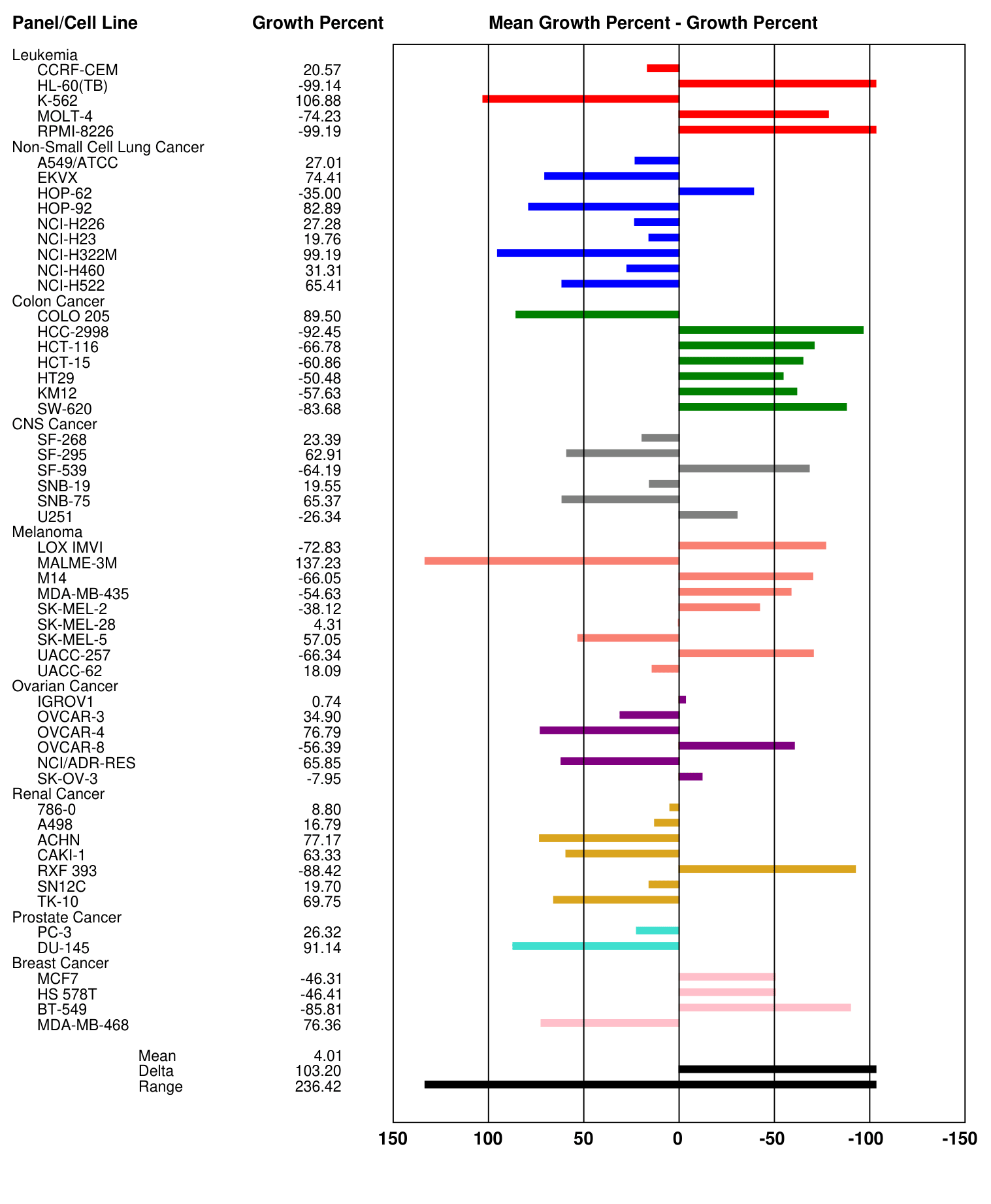


**Figure S45.** One-dose growth (%) and mean graph of compound **8n**


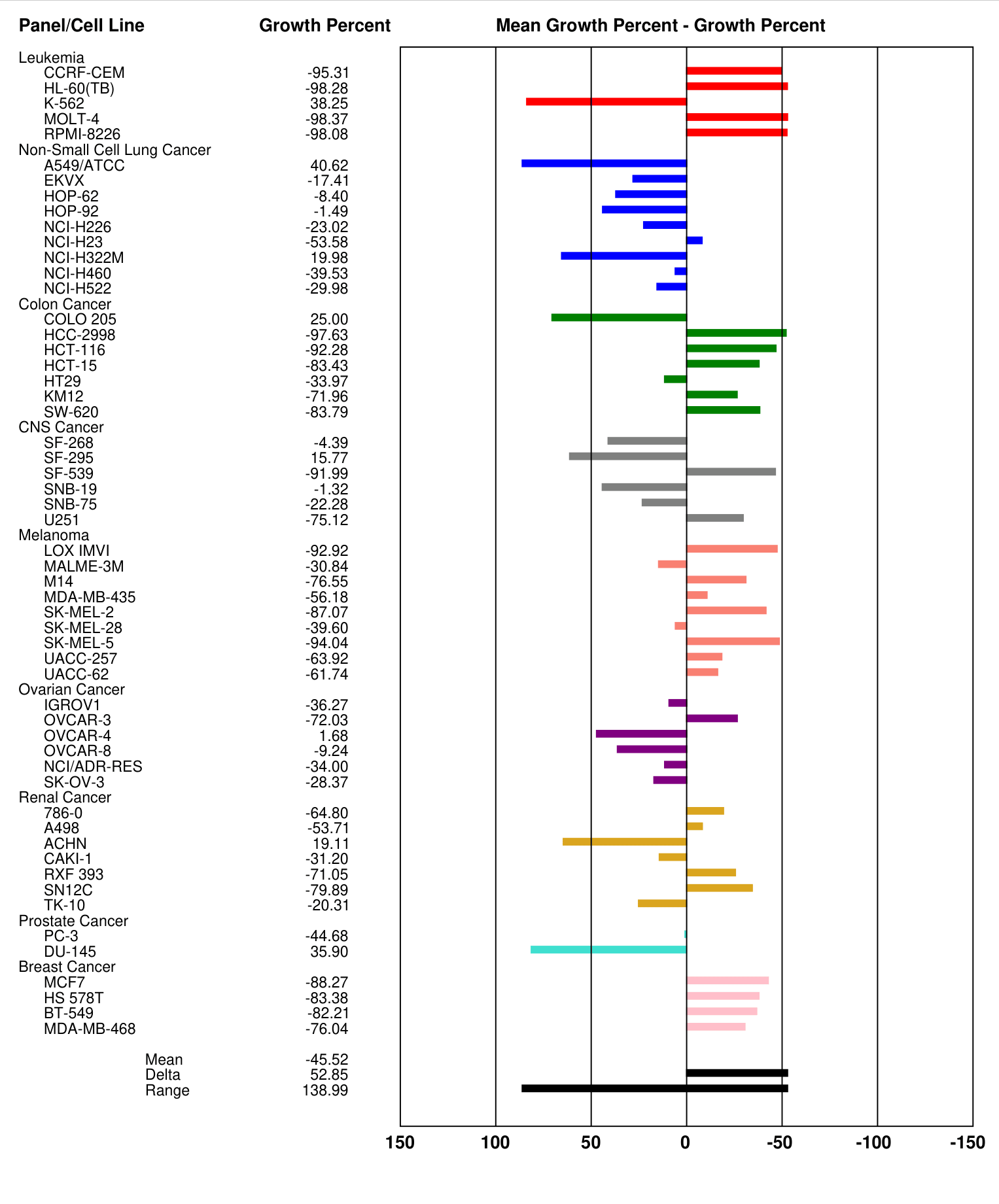


**Figure S46.** One-dose growth (%) and mean graph of compound **8o**


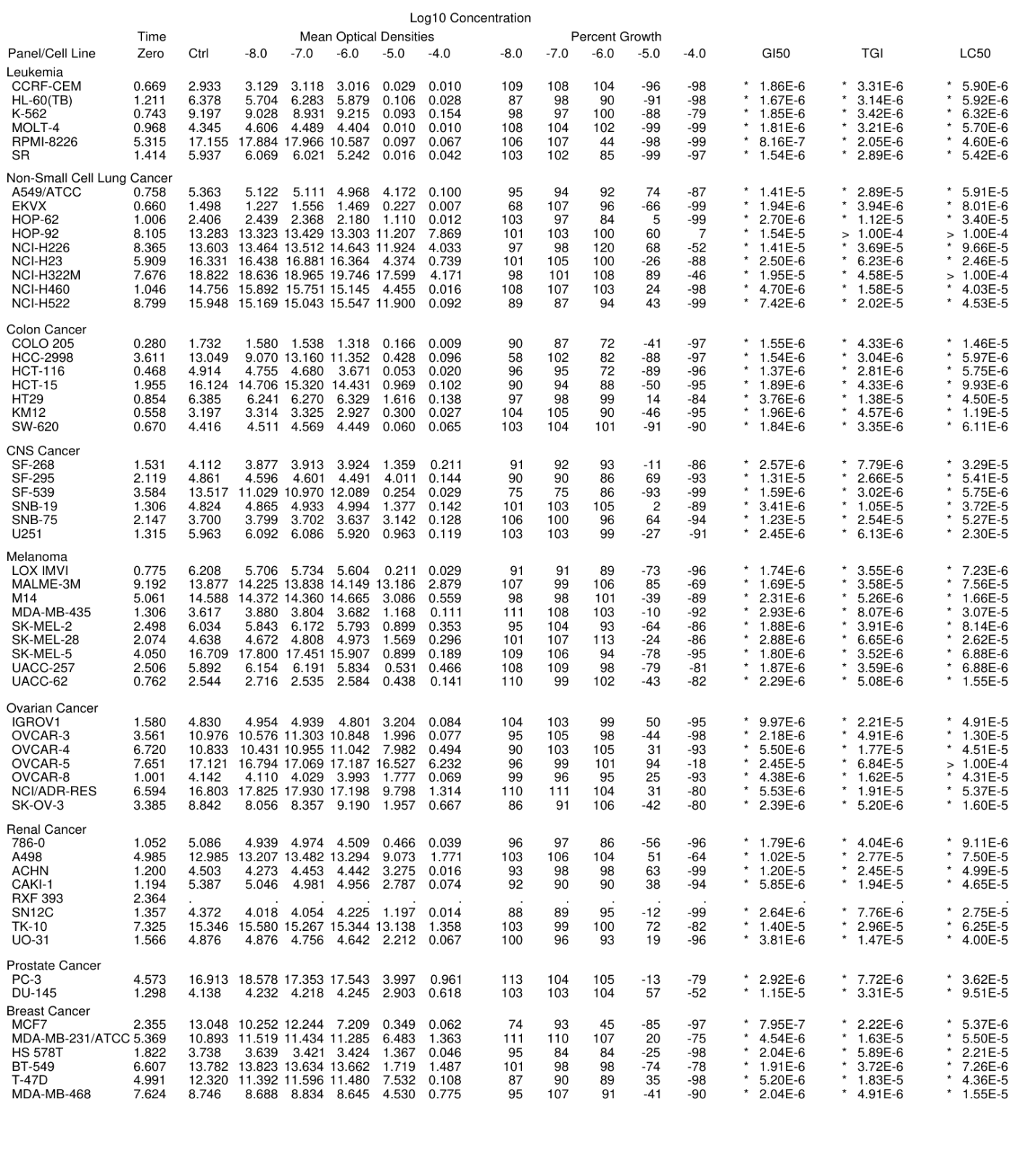


**Figure S47.** *In vitro* five dose analysis of compound **8a**


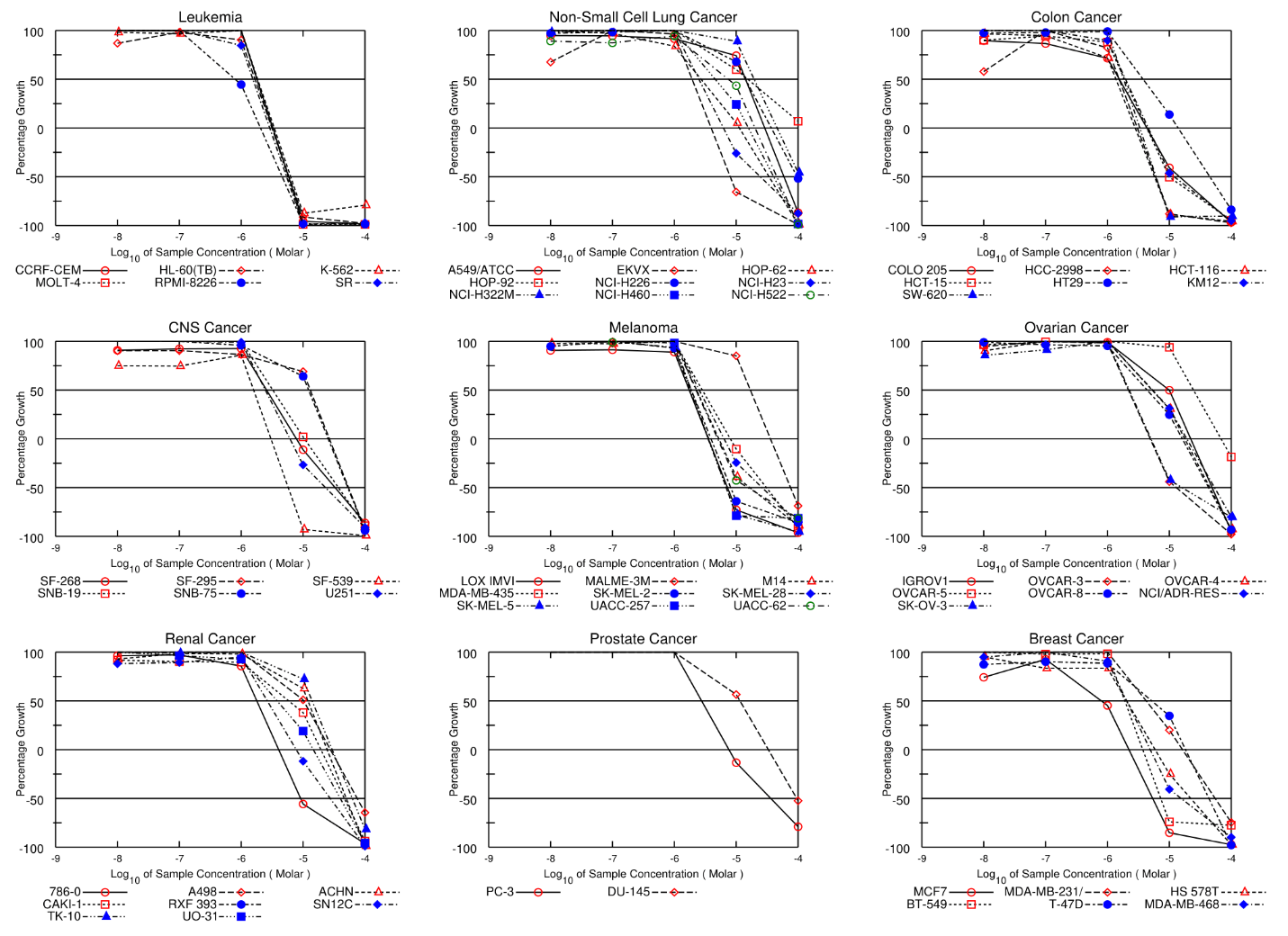


**Figure S48.** Dose response curves for compound **8a**


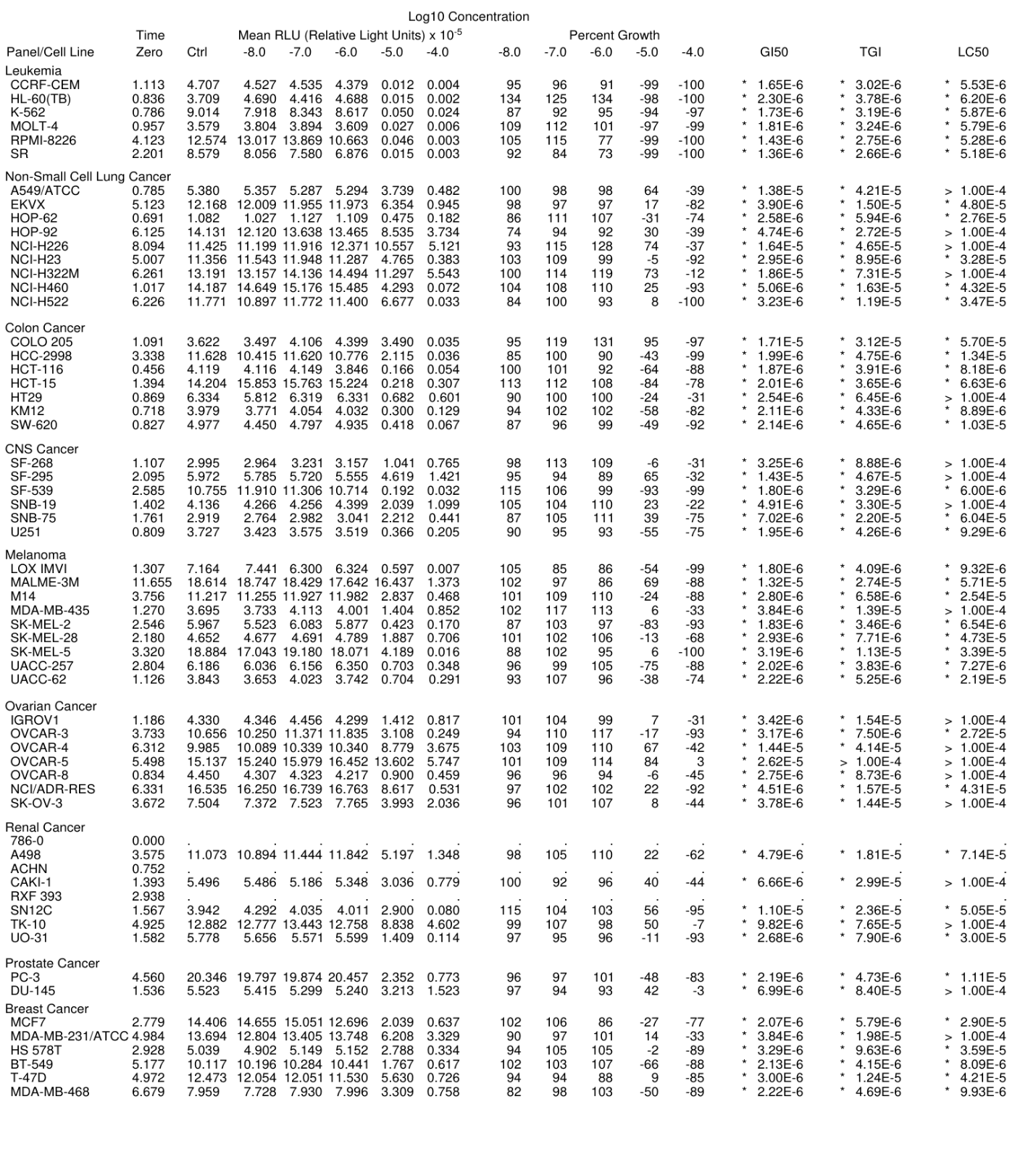


**Figure S49.** *In vitro* five dose analysis of compound **8b**


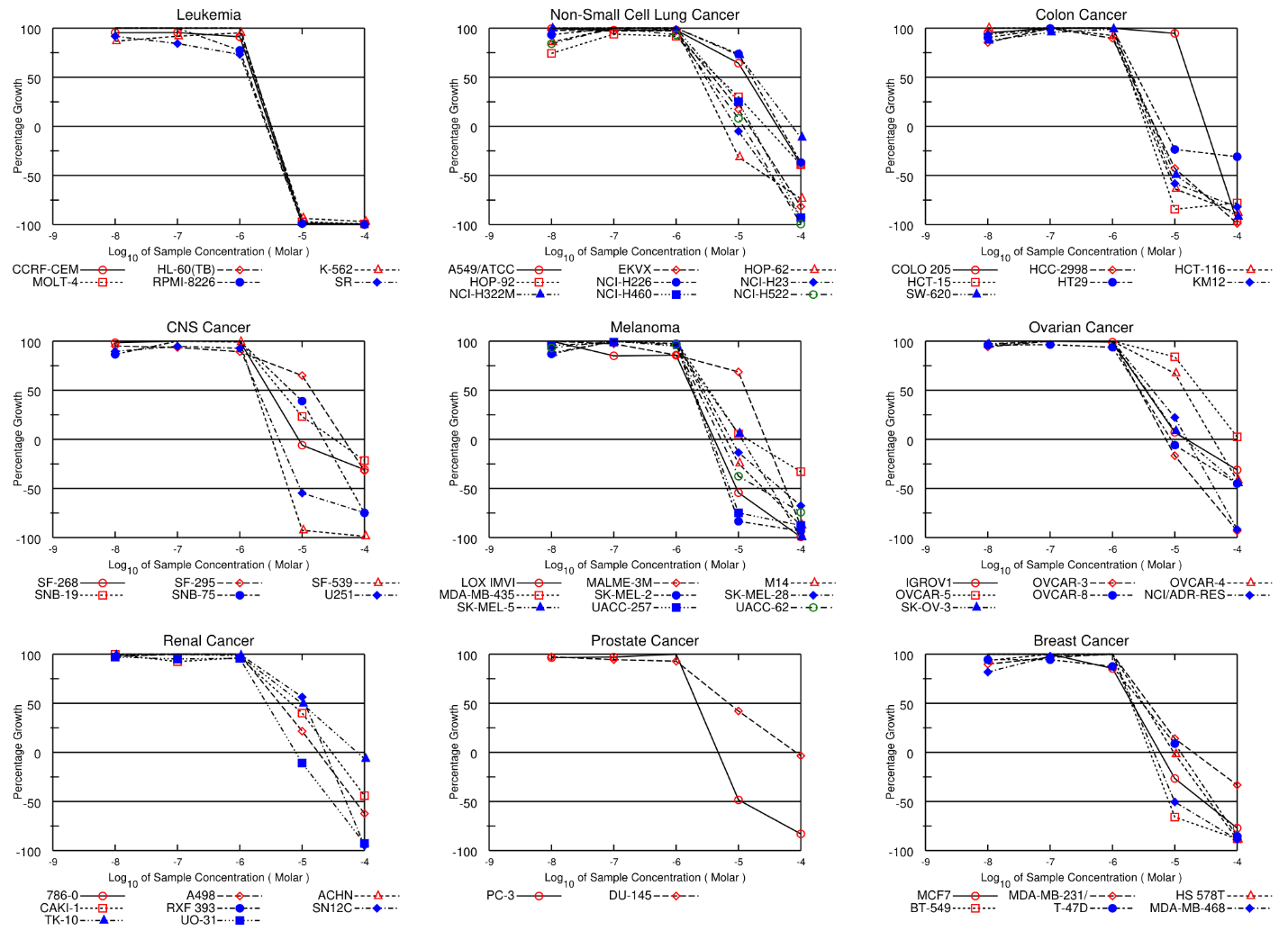


**Figure S50.** Dose response curves for compound **8b**


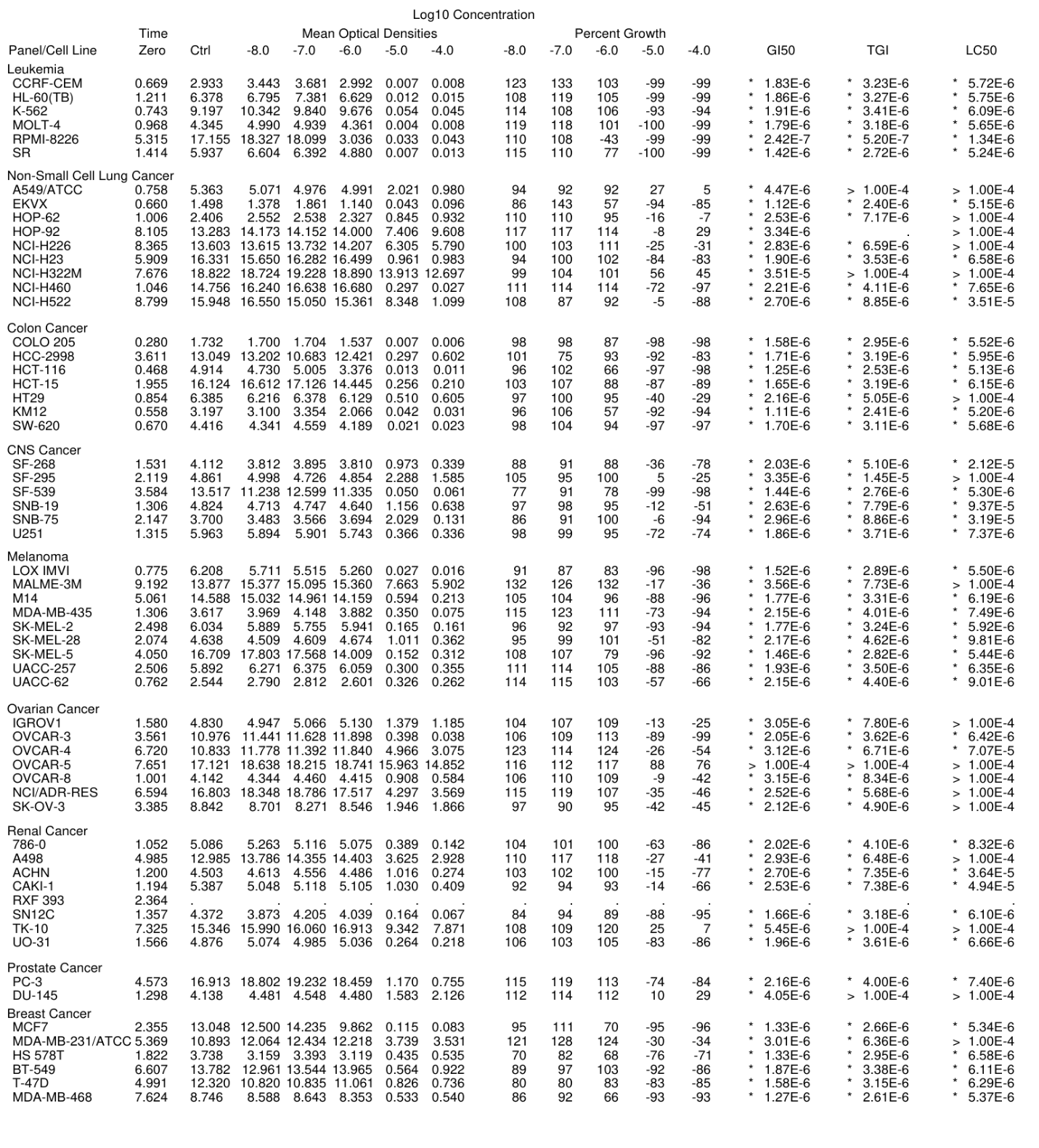


**Figure S51.** *In vitro* five dose analysis of compound **8c**


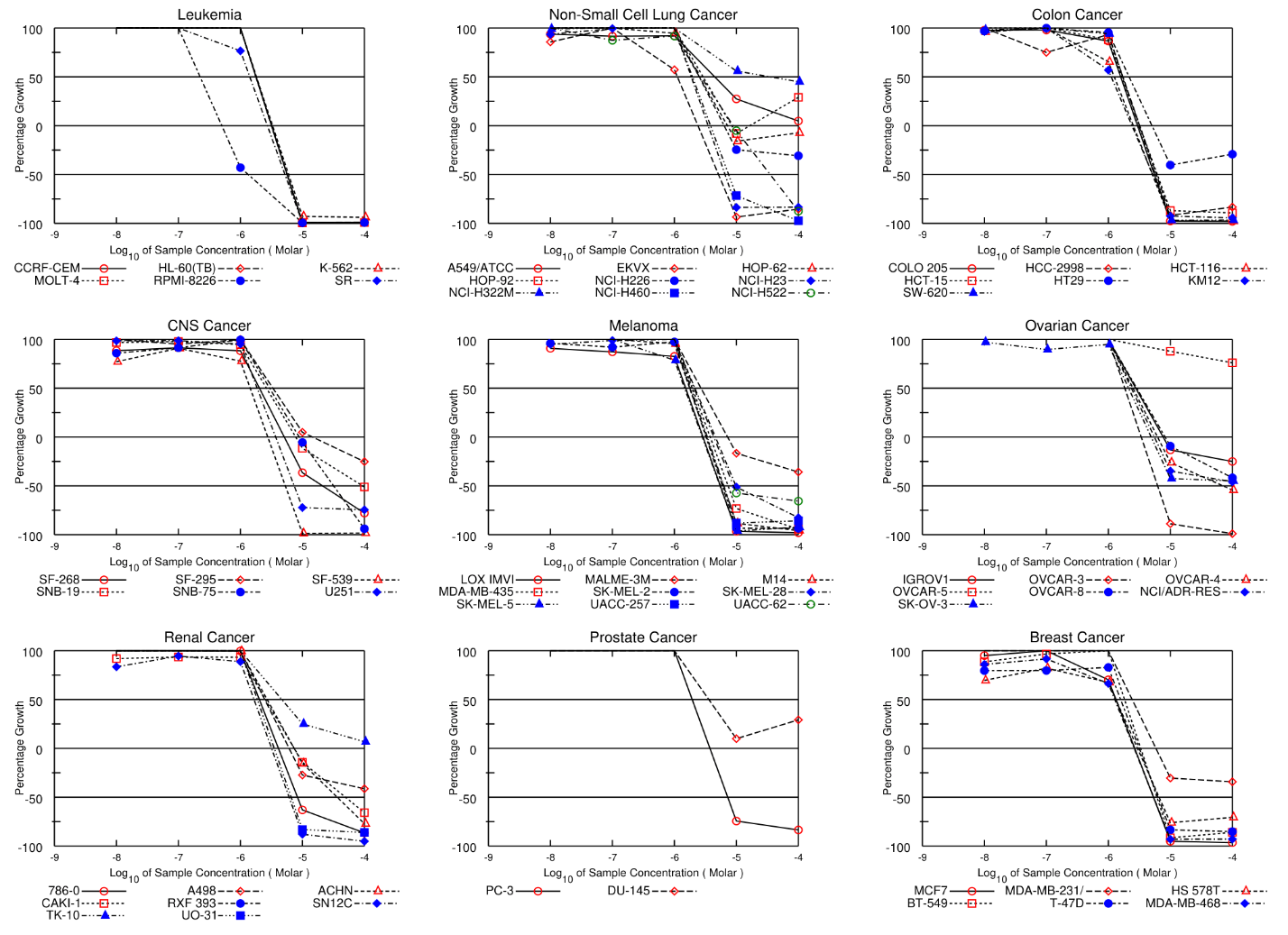


**Figure S52.** Dose response curves for compound **8c**


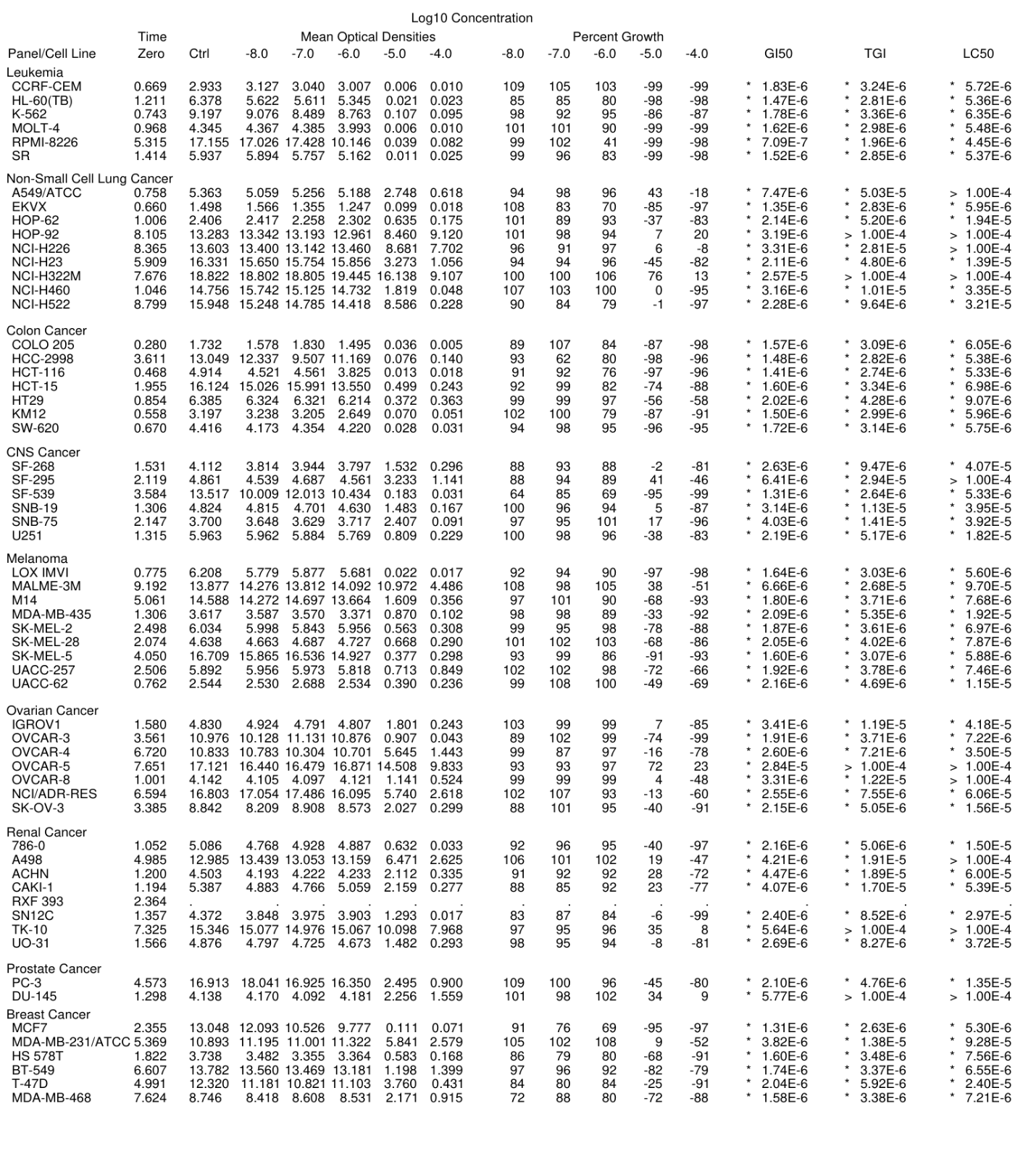


**Figure S53.** *In vitro* five dose analysis of compound **8d**


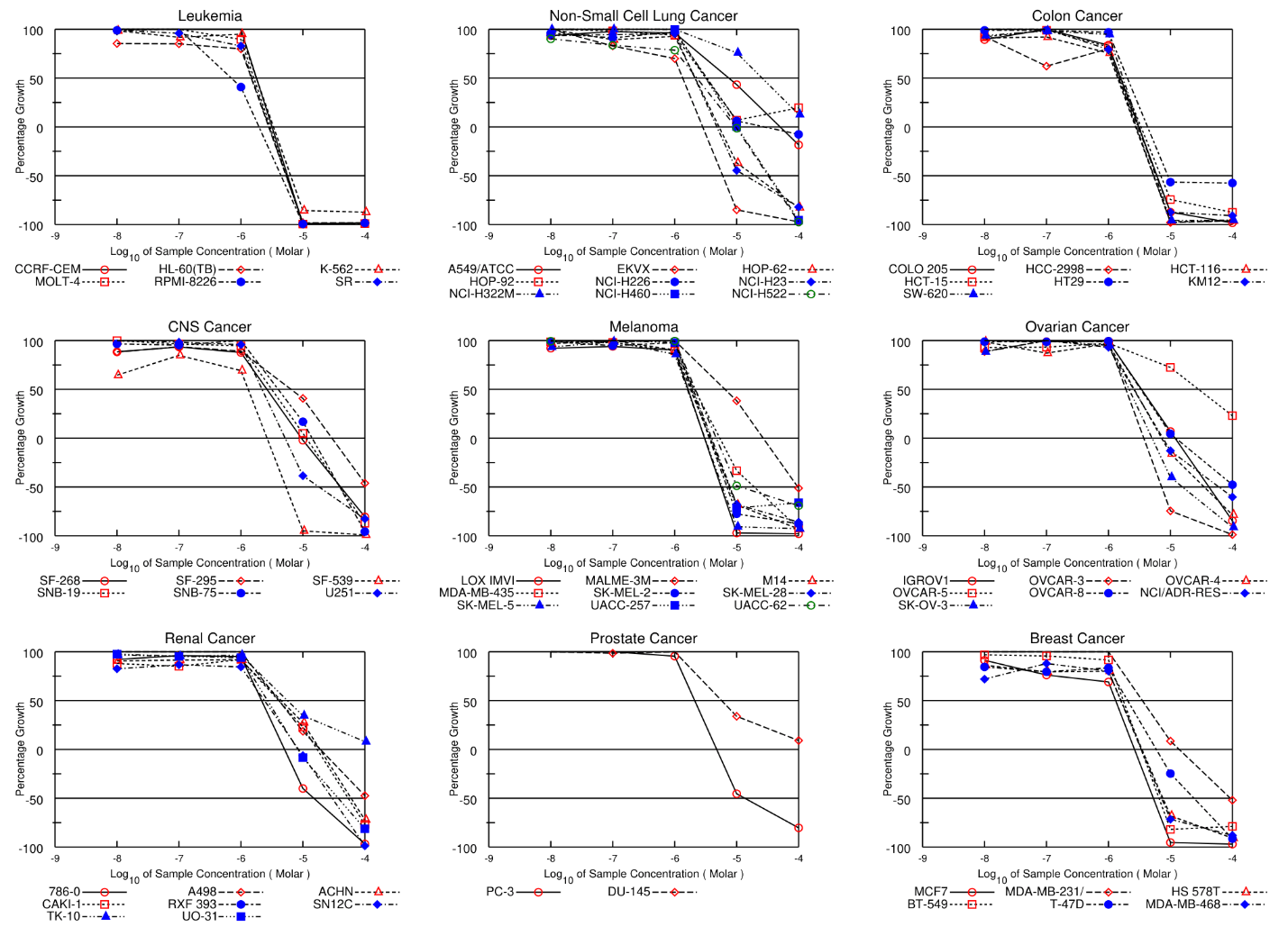


**Figure S54.** Dose response curves for compound **8d**


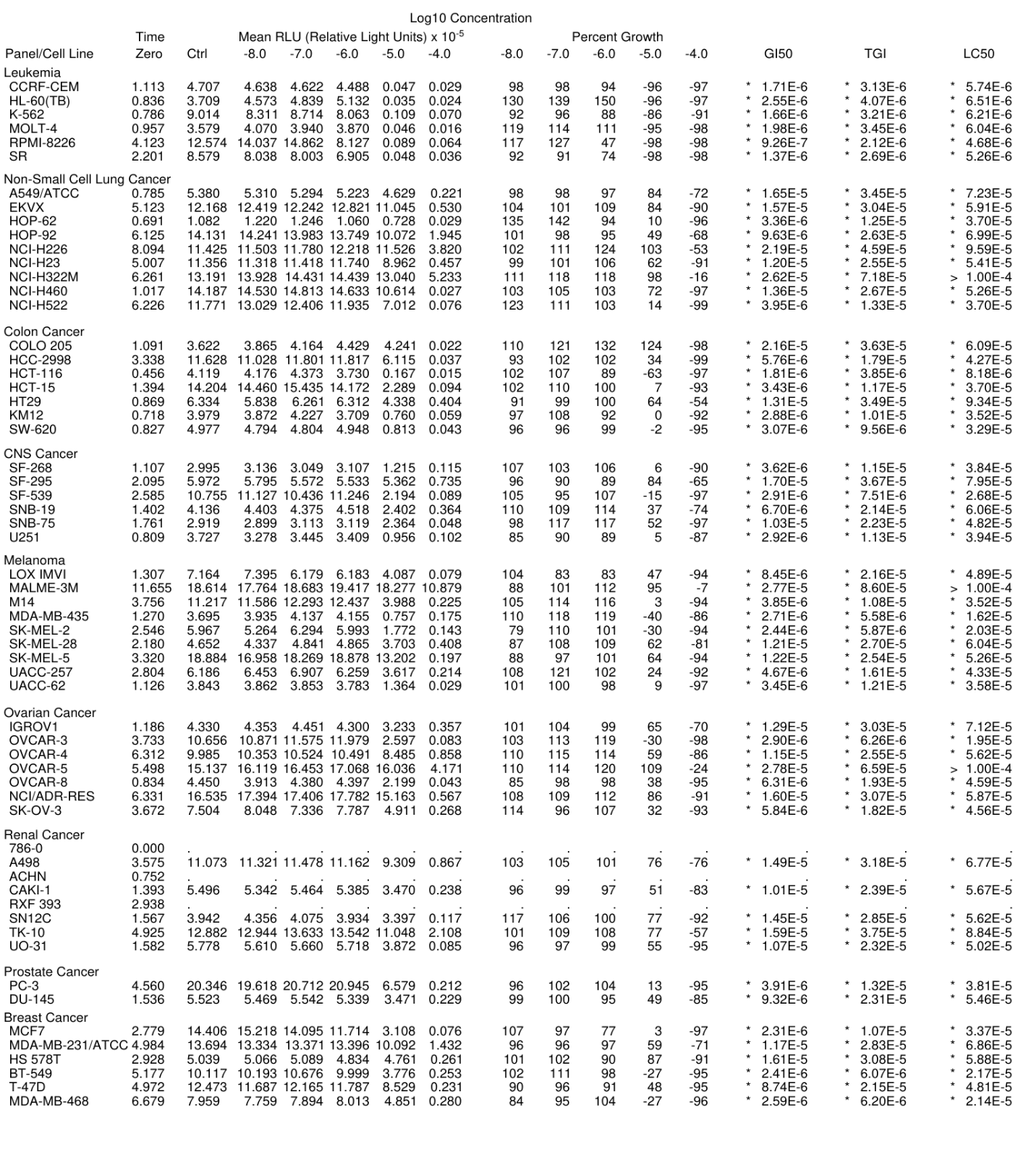


**Figure S55.** *In vitro* five dose analysis of compound **8e**


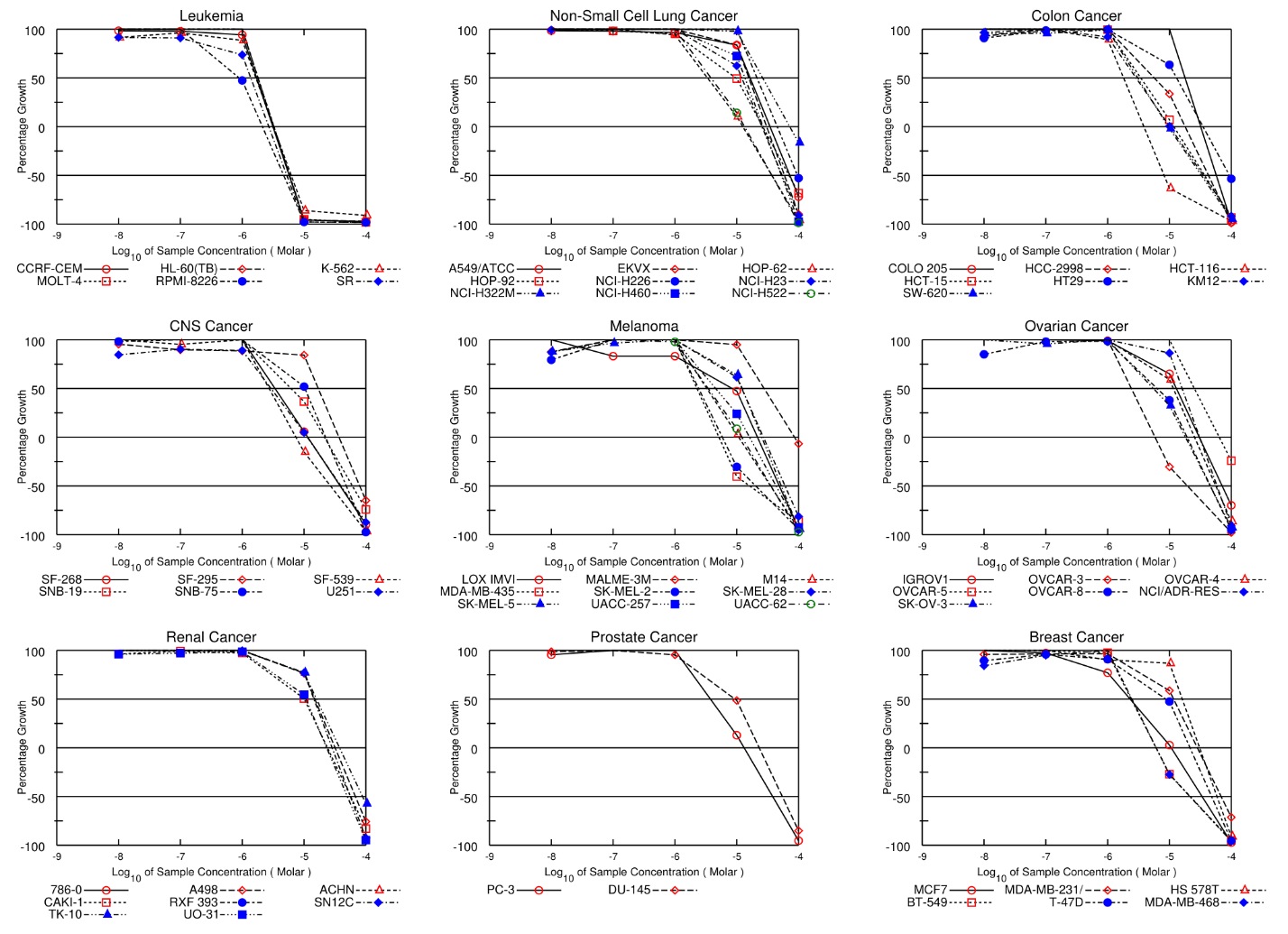


**Figure S56.** Dose response curves for compound **8e**


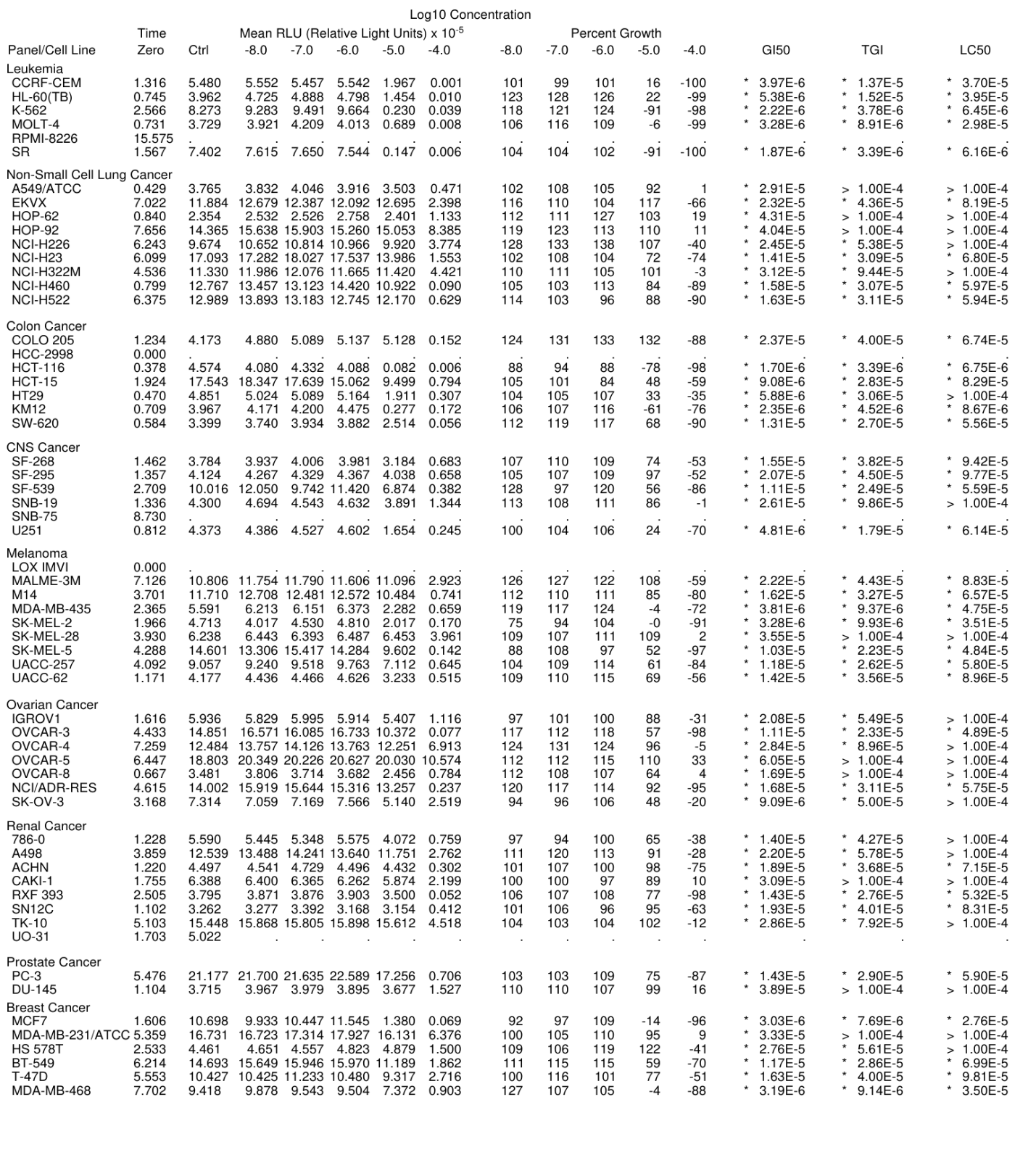


**Figure S57.** *In vitro* five dose analysis of compound **8f**


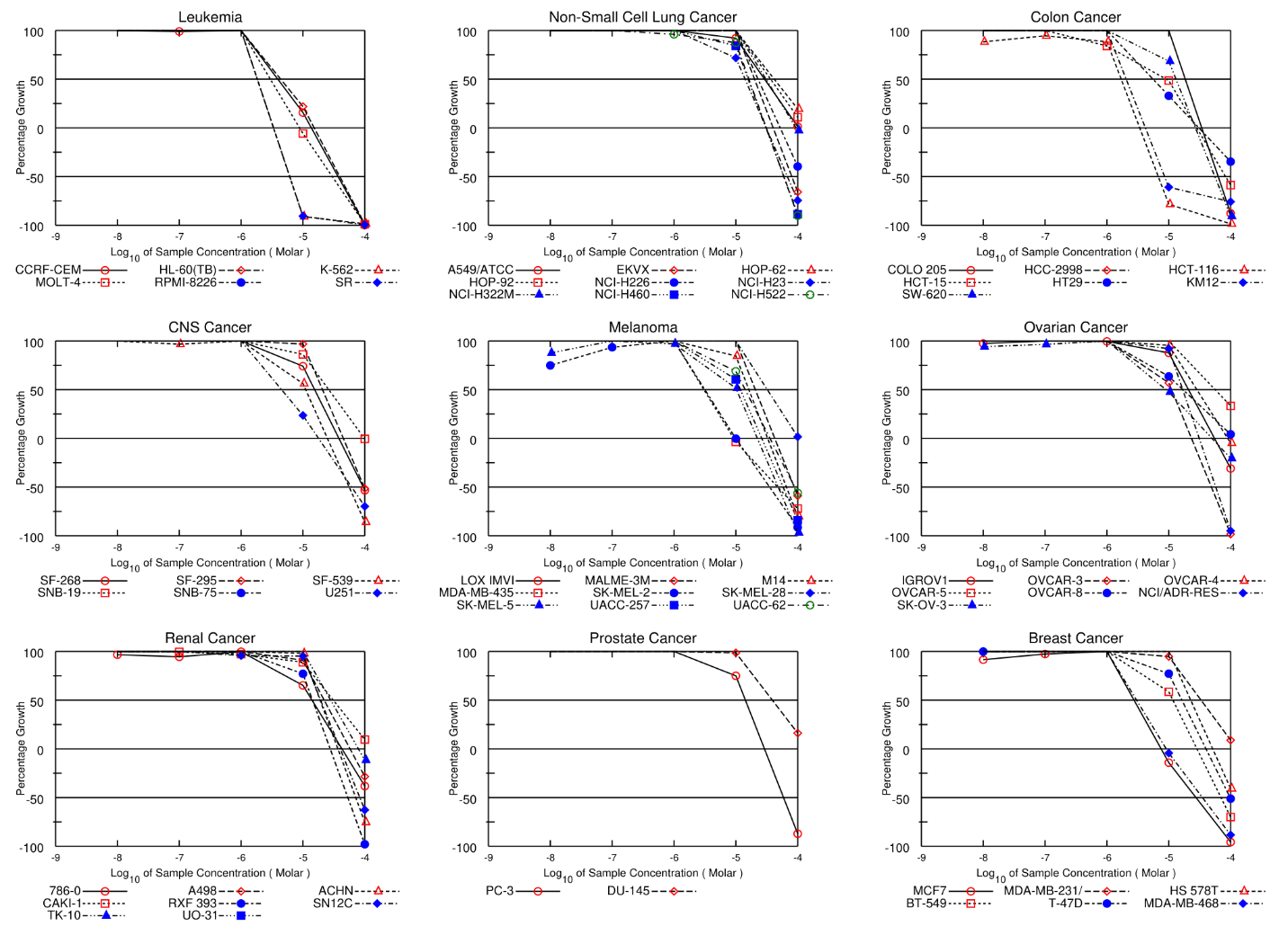


**Figure S58.** Dose response curves for compound **8f**


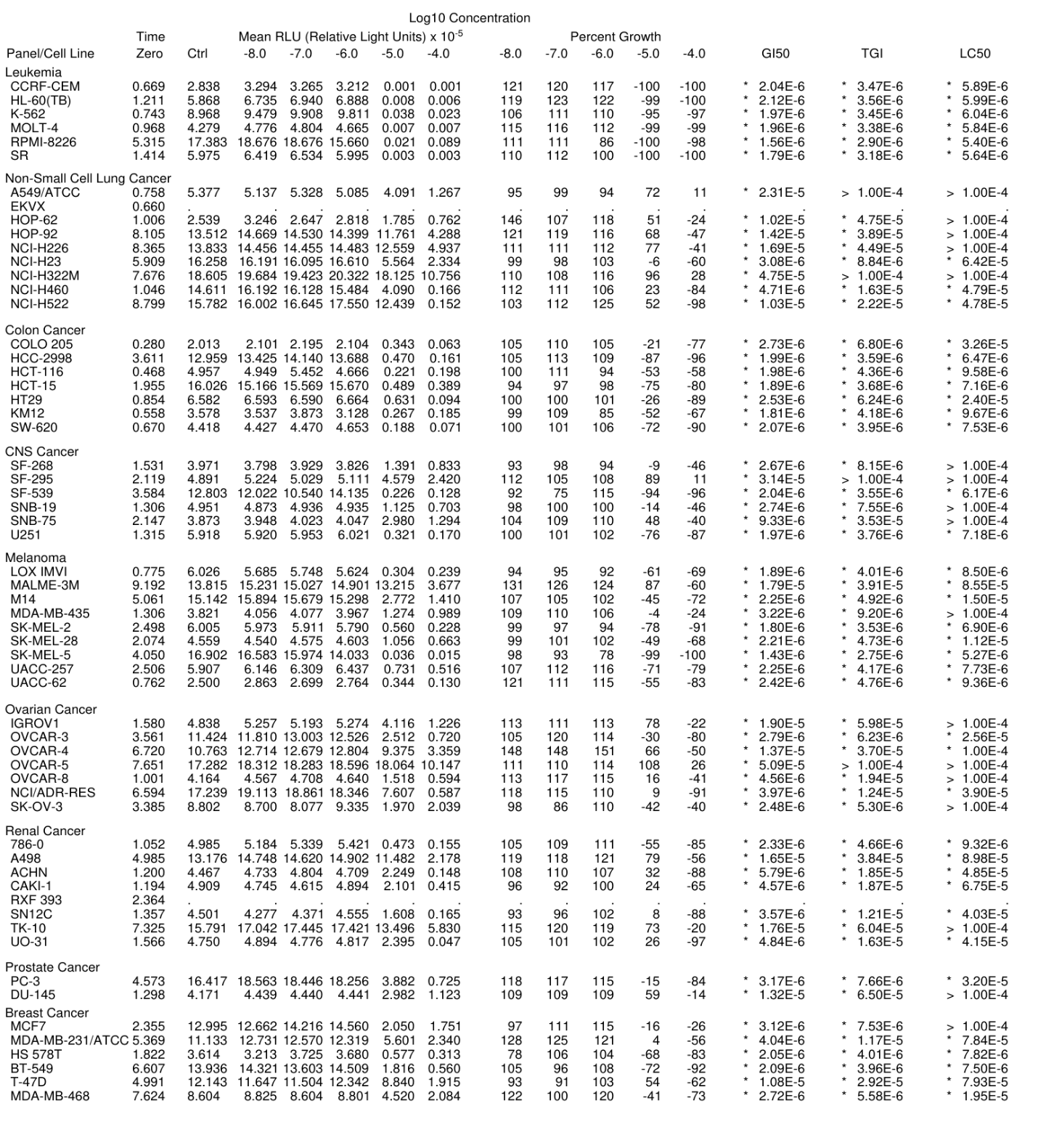


**Figure S59.** *In vitro* five dose analysis of compound **8g**


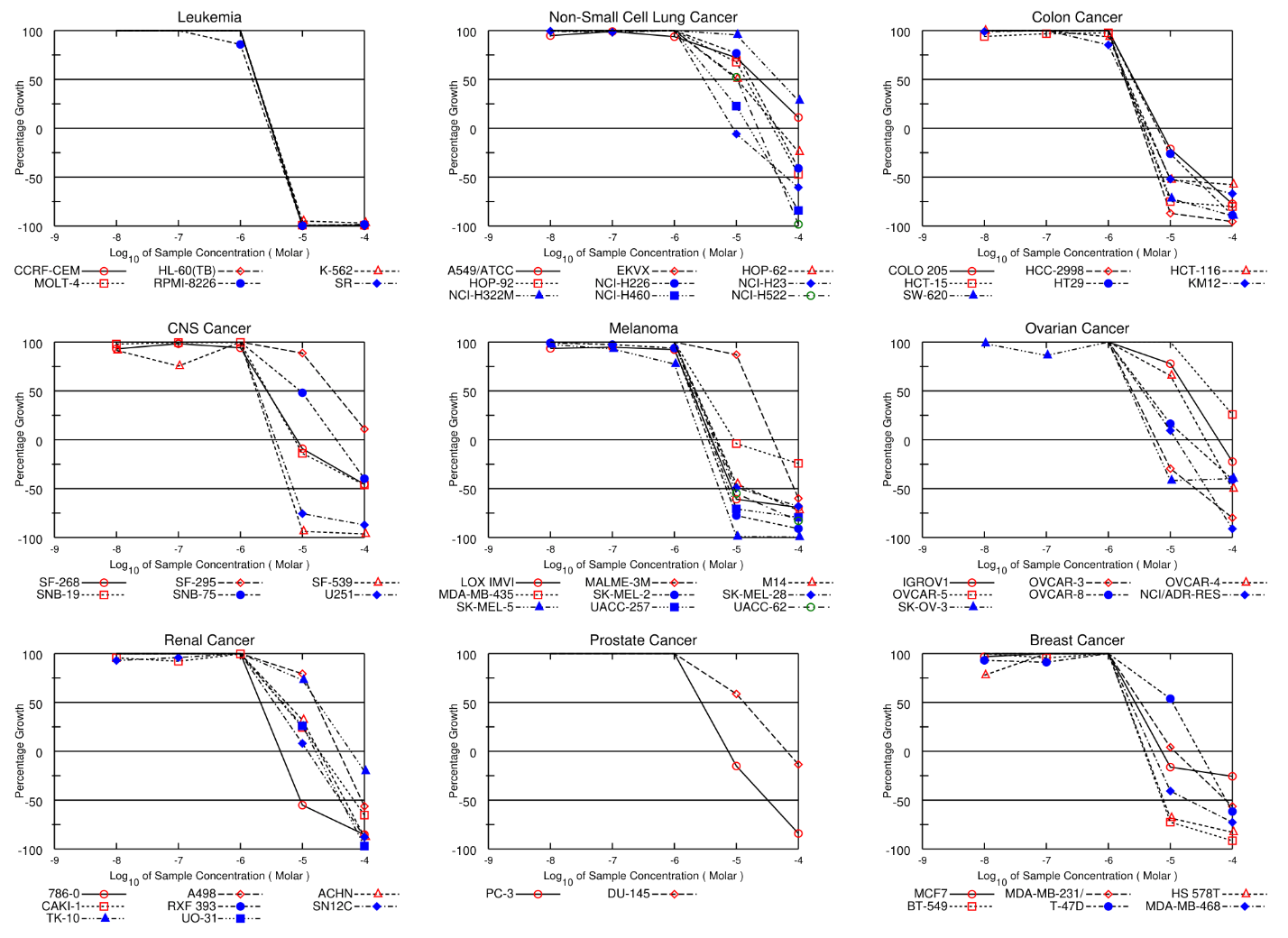


**Figure S60.** Dose response curves for compound **8g**


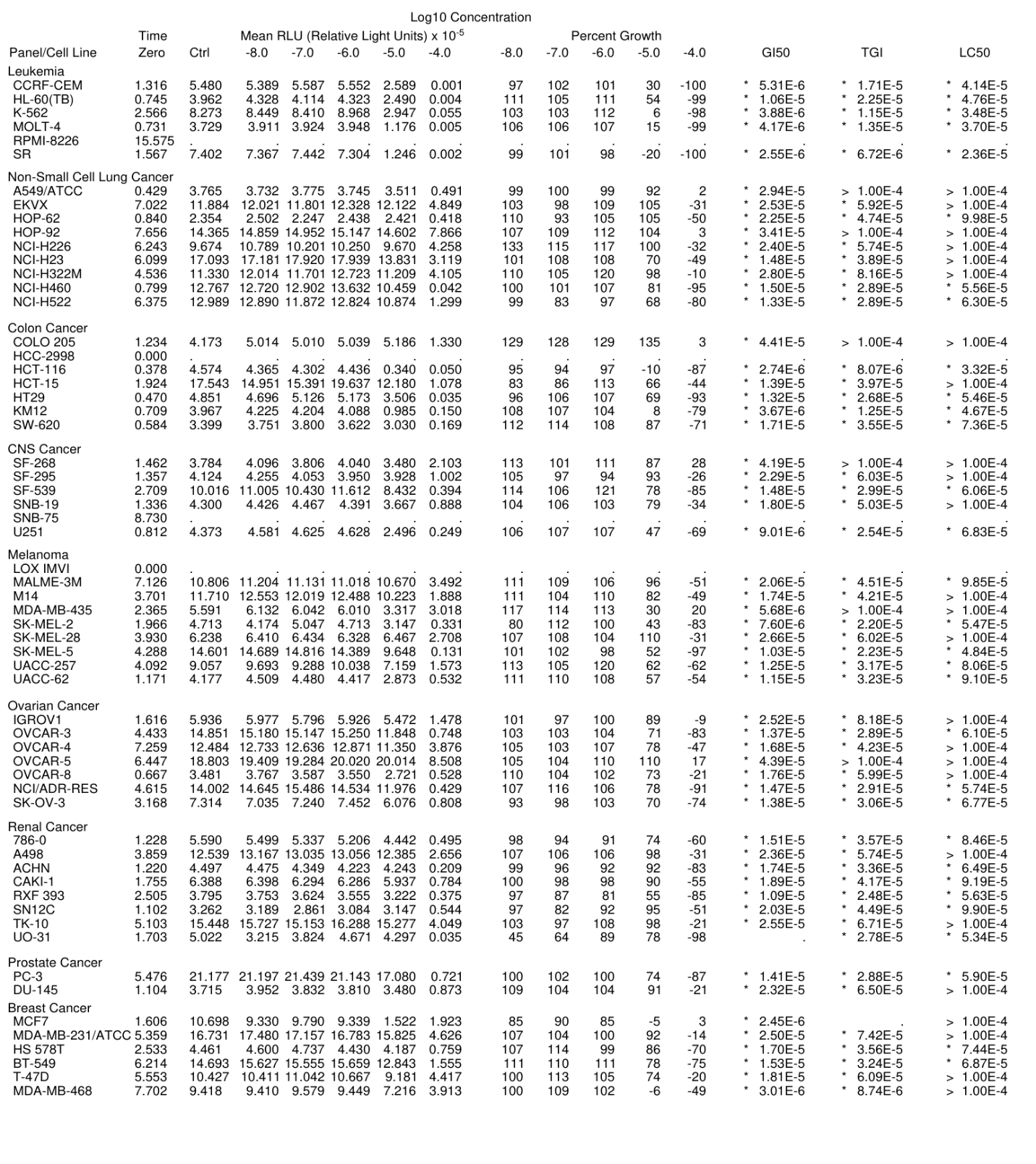


**Figure S61.** *In vitro* five dose analysis of compound **8h**


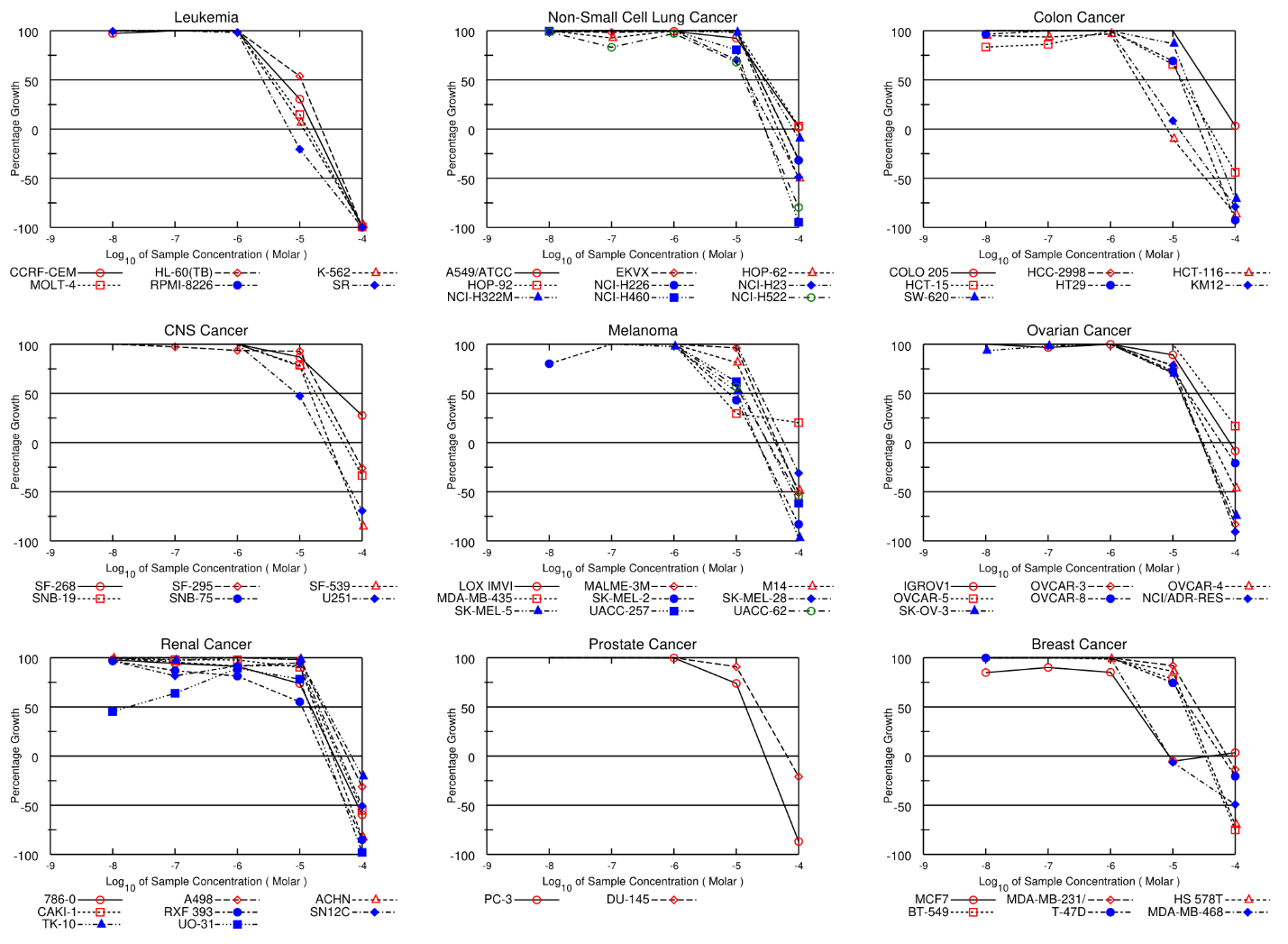


**Figure S62.** Dose response curves for compound **8h**


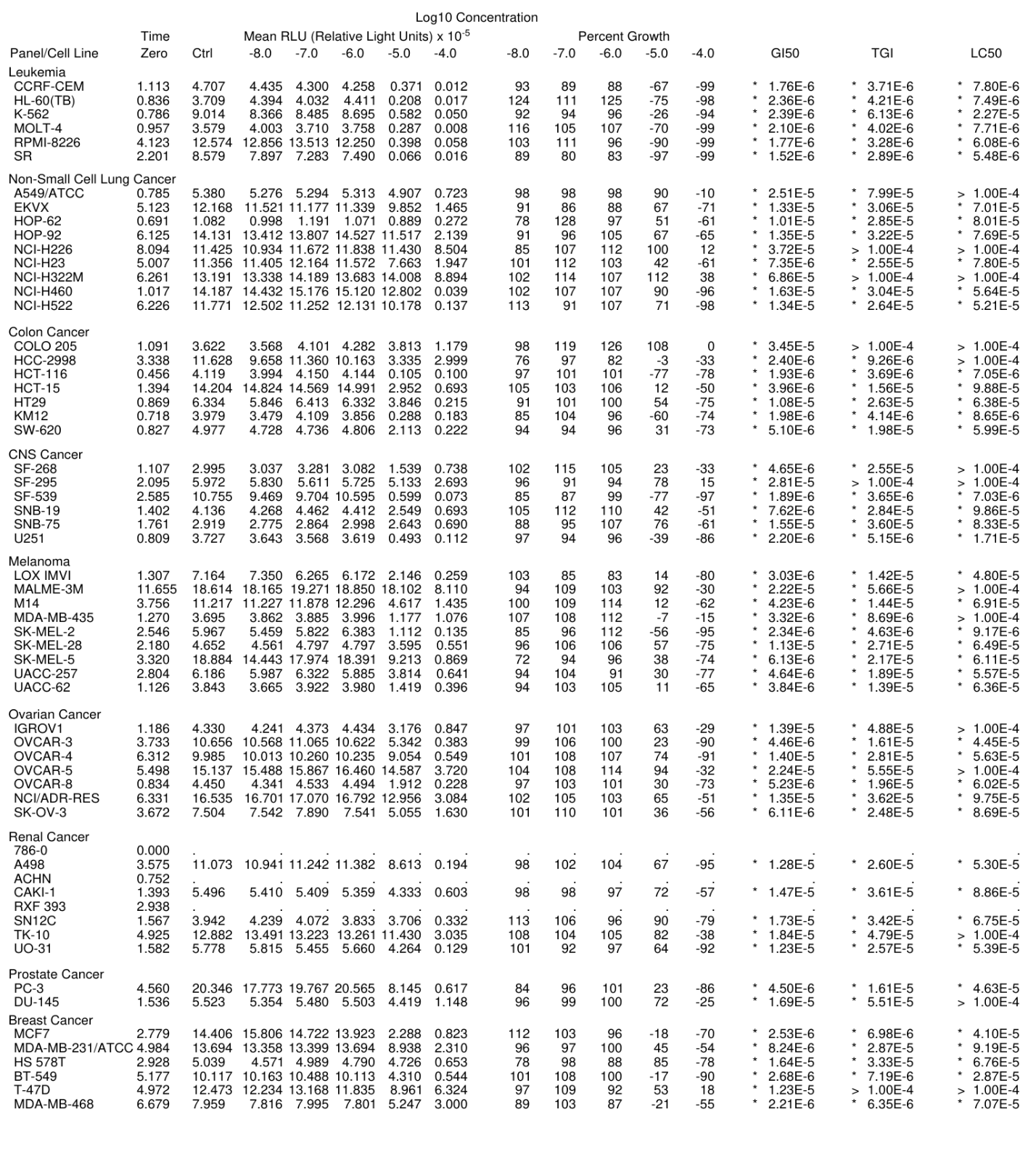


**Figure S63.** *In vitro* five dose analysis of compound **8i**


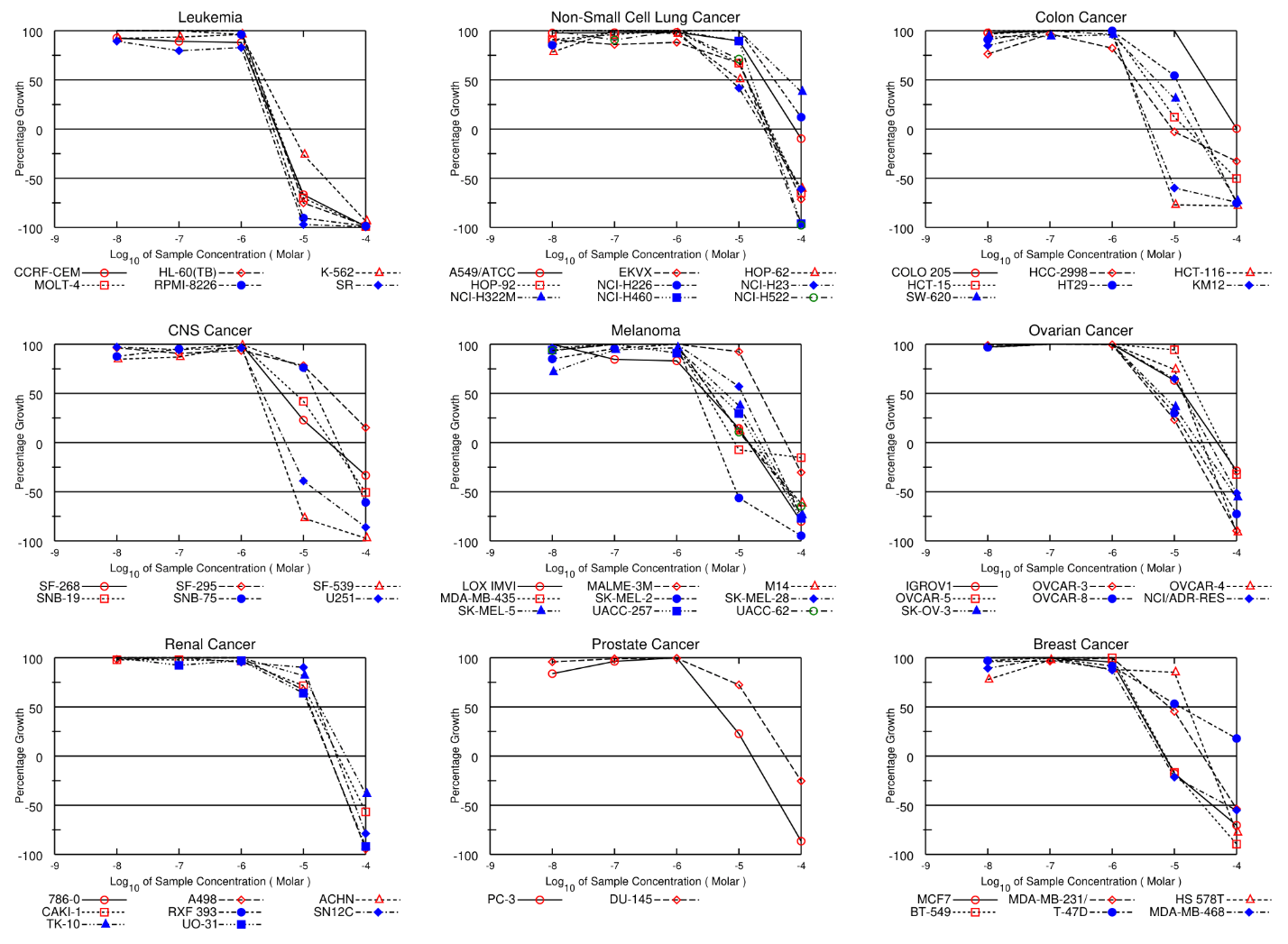


**Figure S64.** Dose response curves for compound **8i**


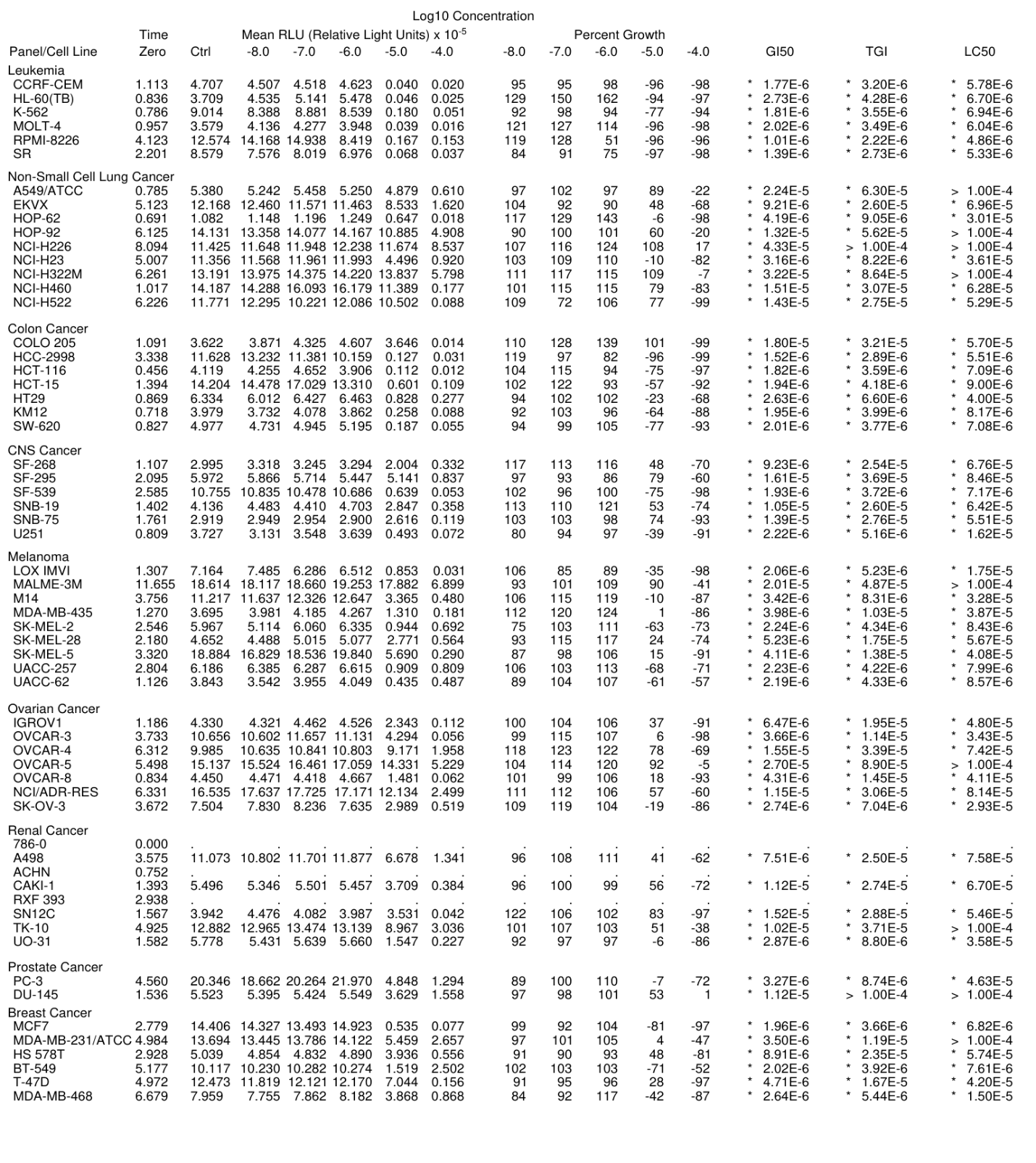


**Figure S65.** *In vitro* five dose analysis of compound **8j**


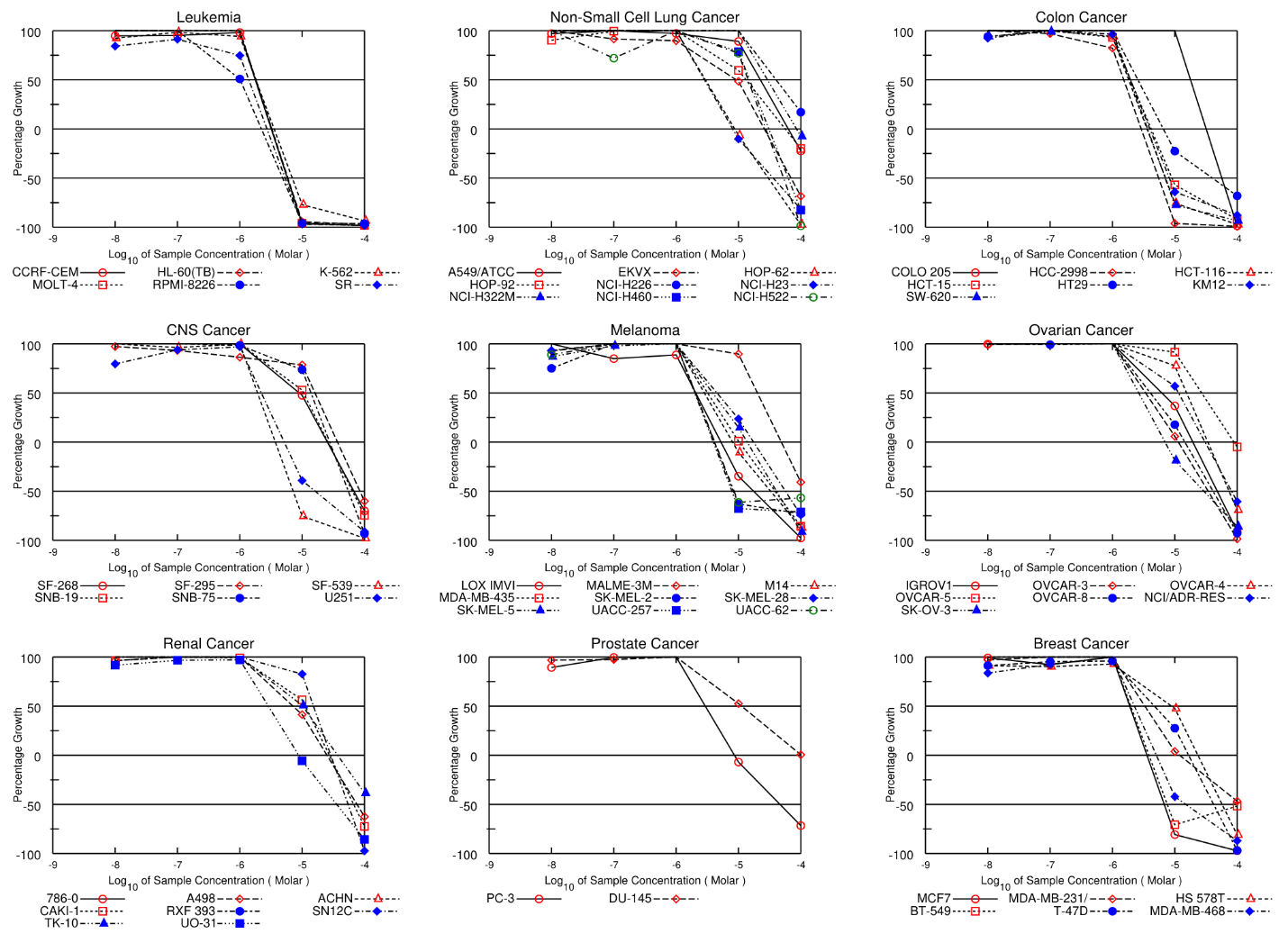


**Figure S66.** Dose response curves for compound **8j**


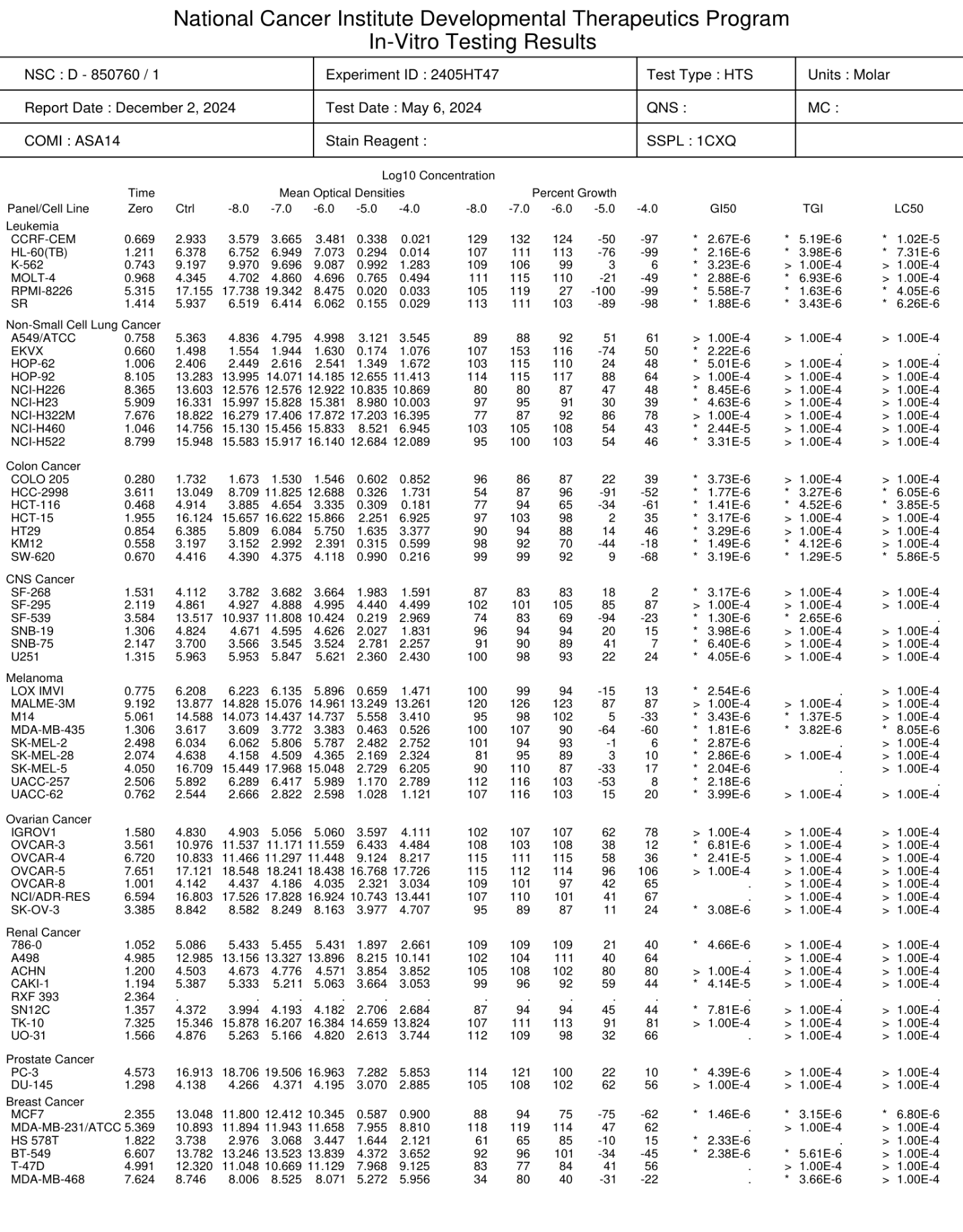


**Figure S67.** *In vitro* five dose analysis of compound **8k**


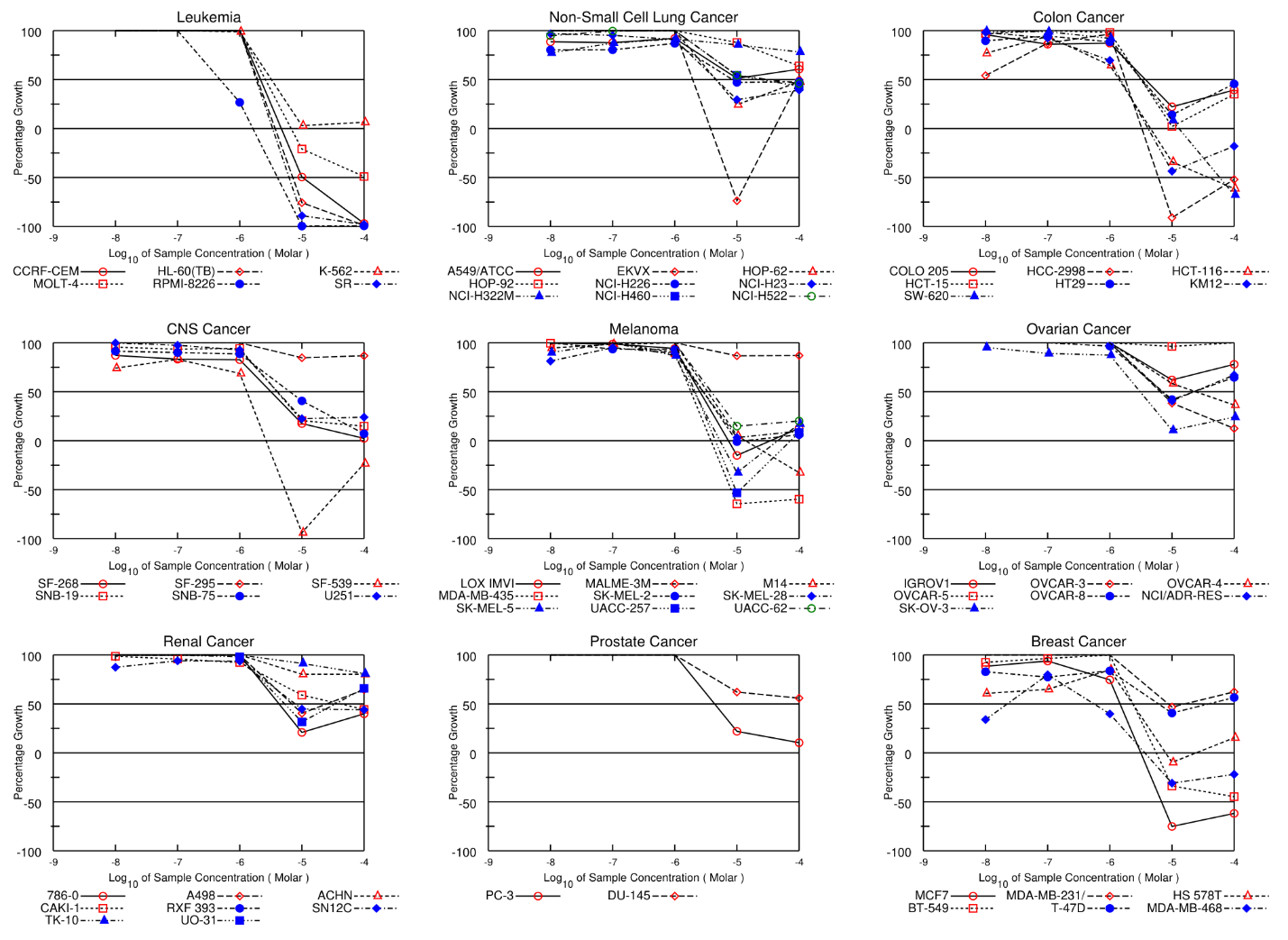


**Figure S68.** Dose response curves for compound **8k**


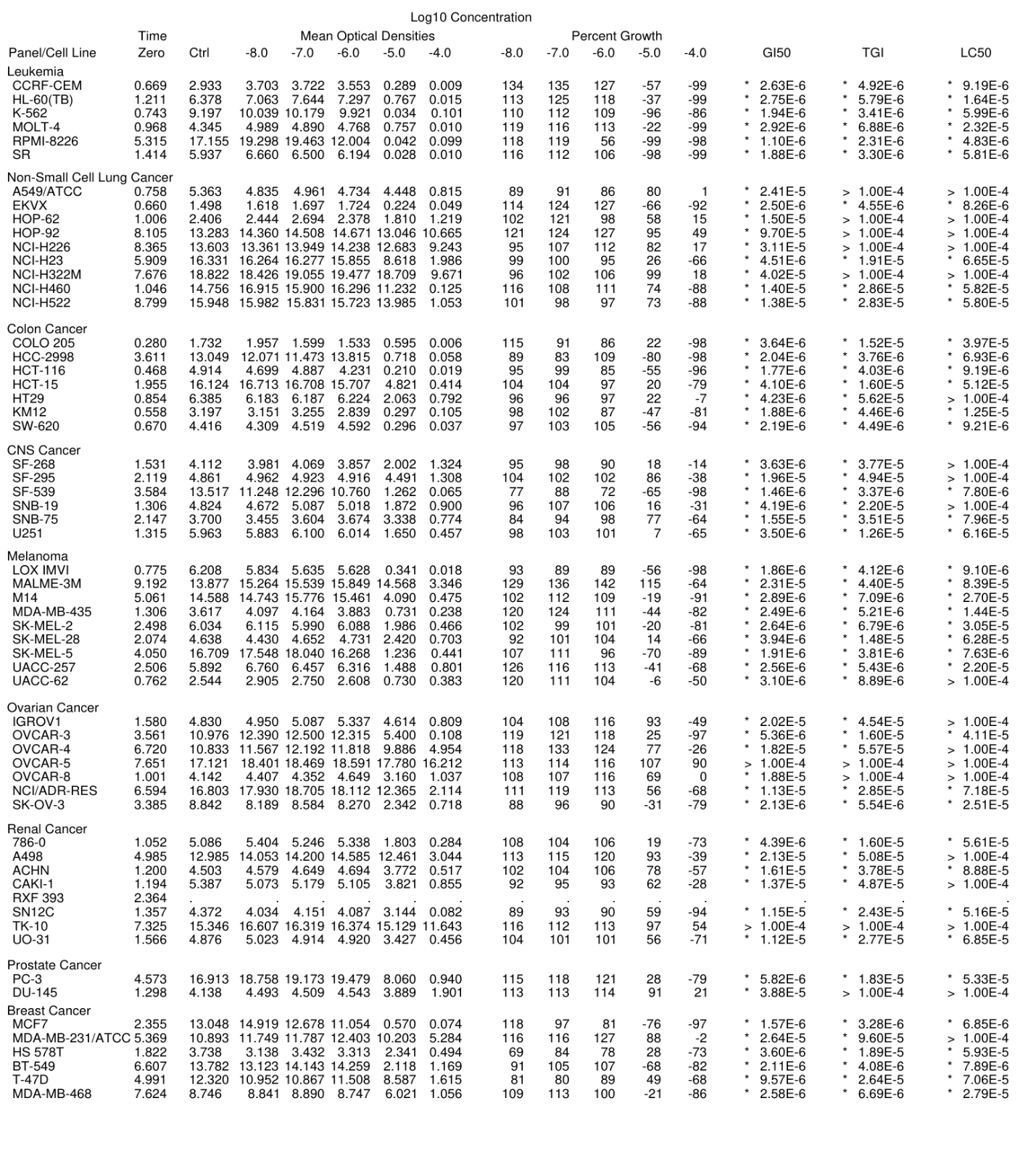


**Figure S69.** *In vitro* five dose analysis of compound **8l**


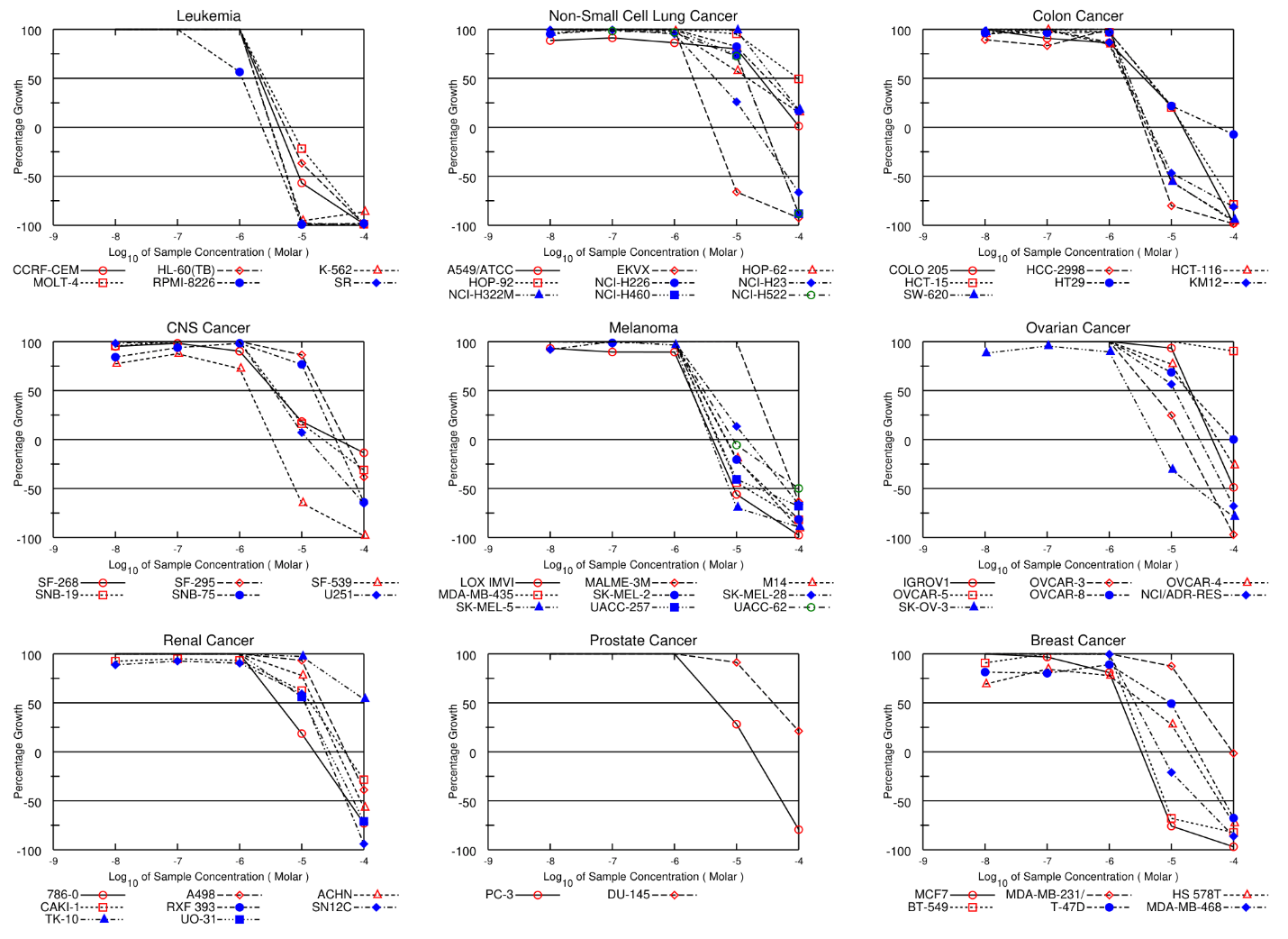


**Figure S70.** Dose response curves for compound **8l**


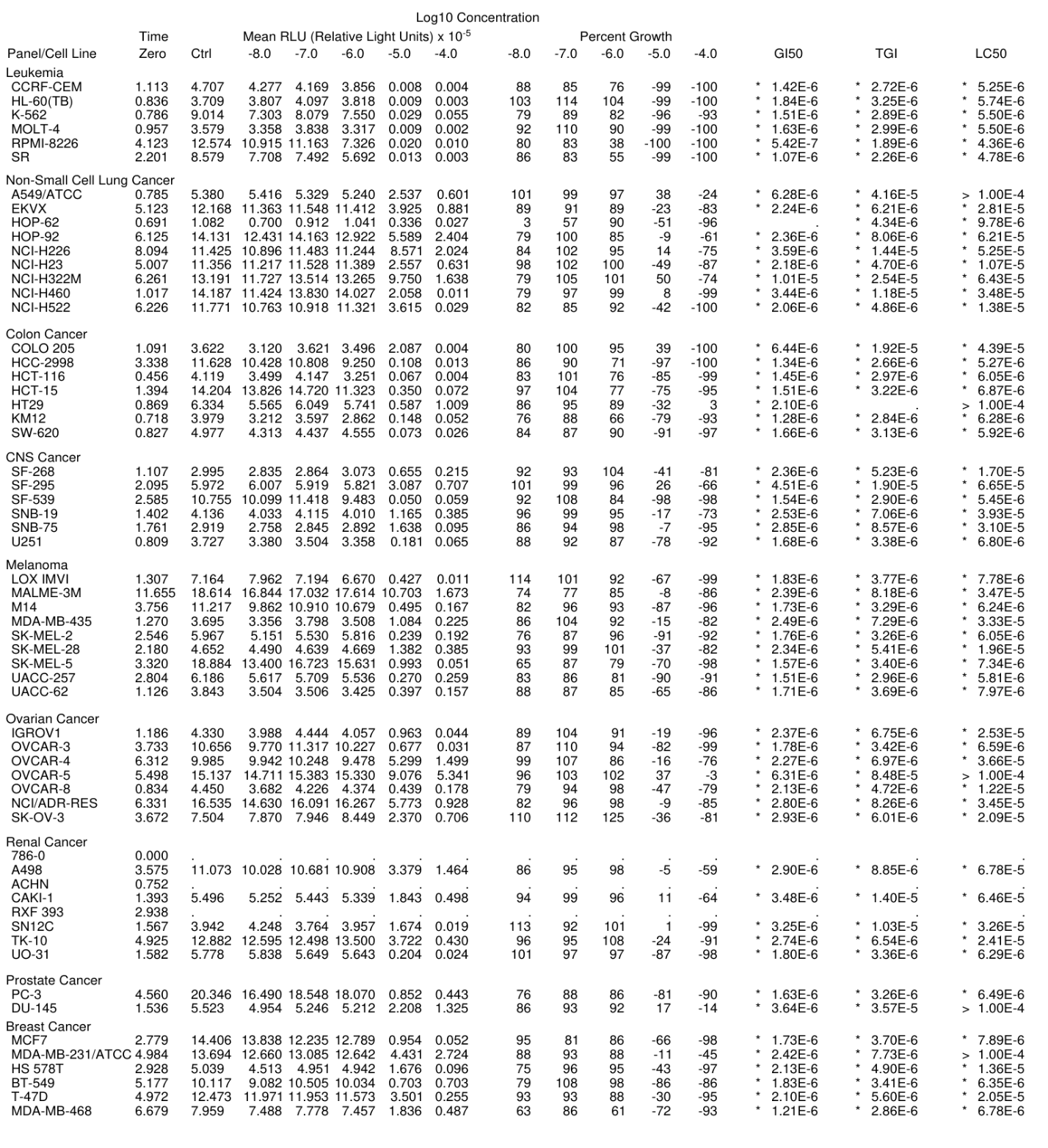


**Figure S71.** *In vitro* five dose analysis of compound **8m**


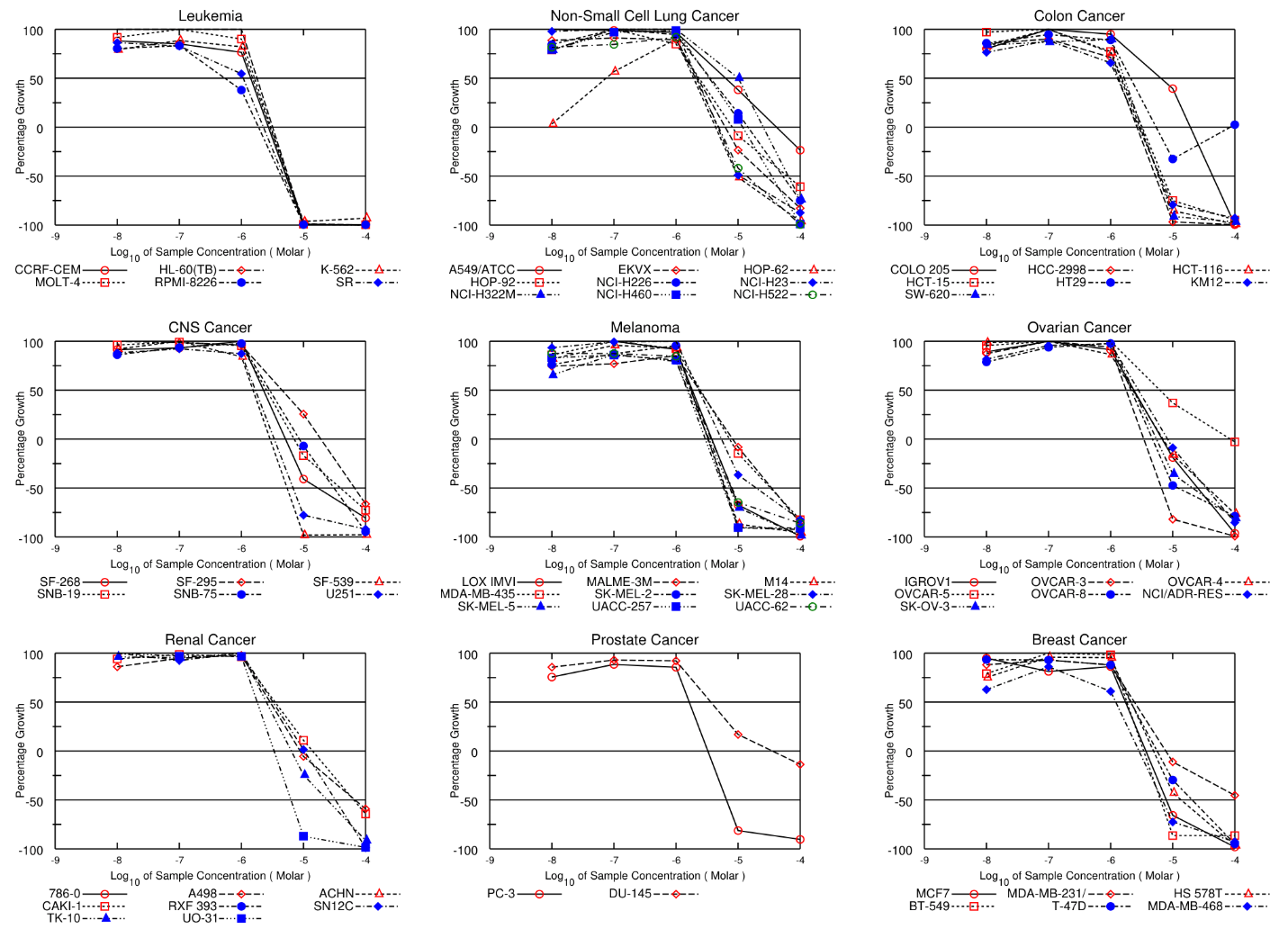


**Figure S72.** Dose response curves for compound **8m**


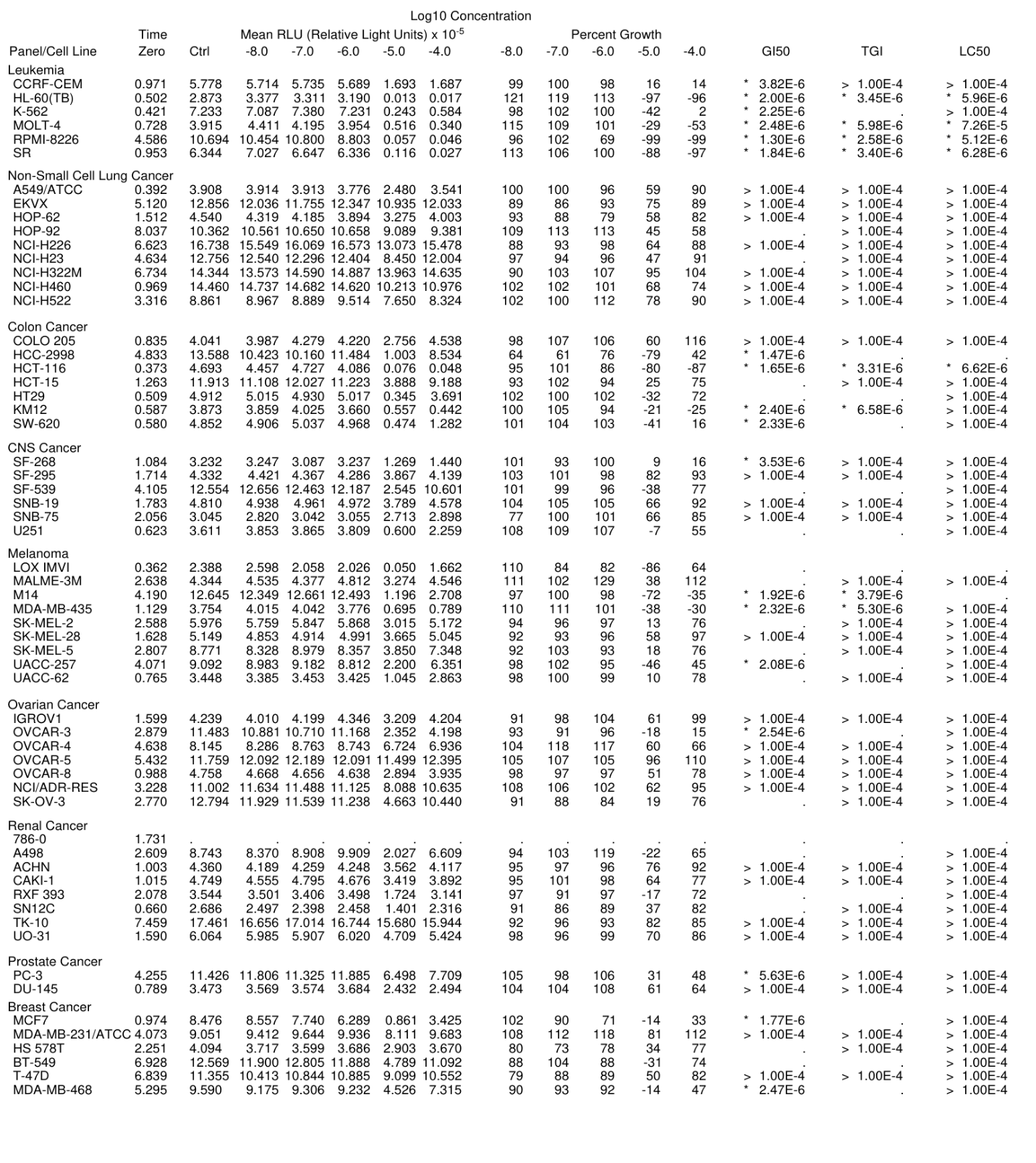


**Figure S73.** *In vitro* five dose analysis of compound **8n**


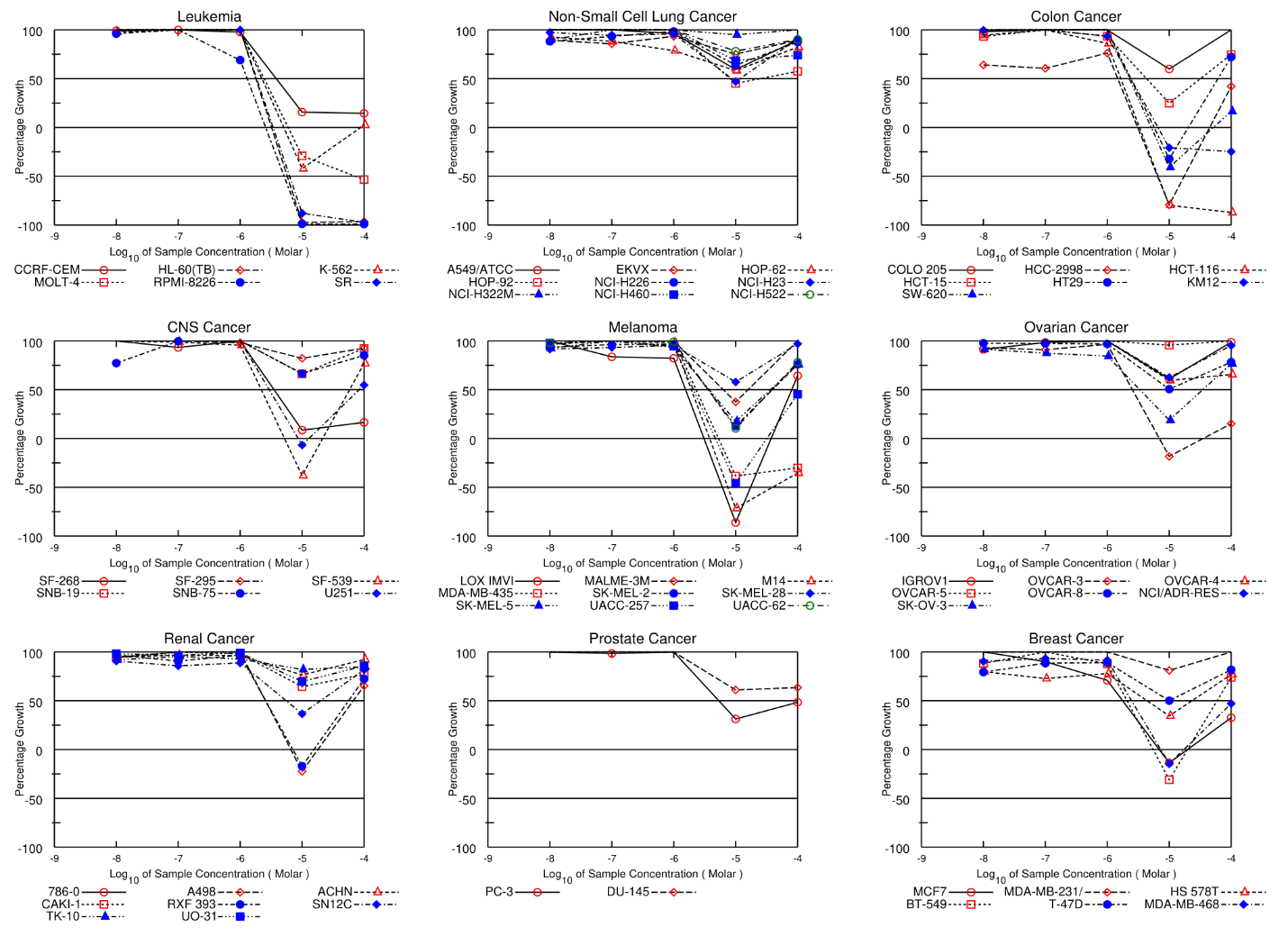


**Figure S74.** Dose response curves for compound **8n**


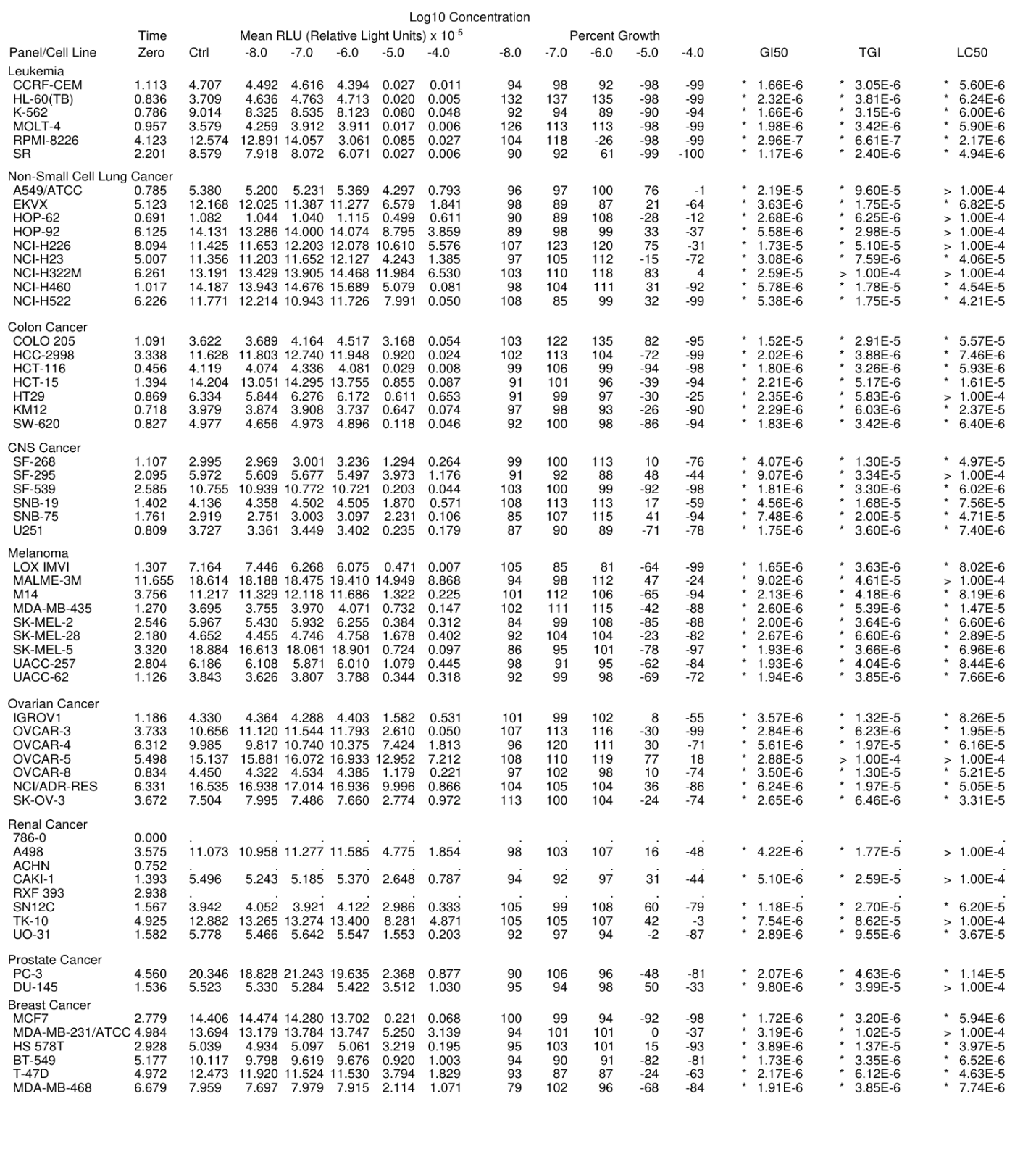


**Figure S75.** *In vitro* five dose analysis of compound **8o**


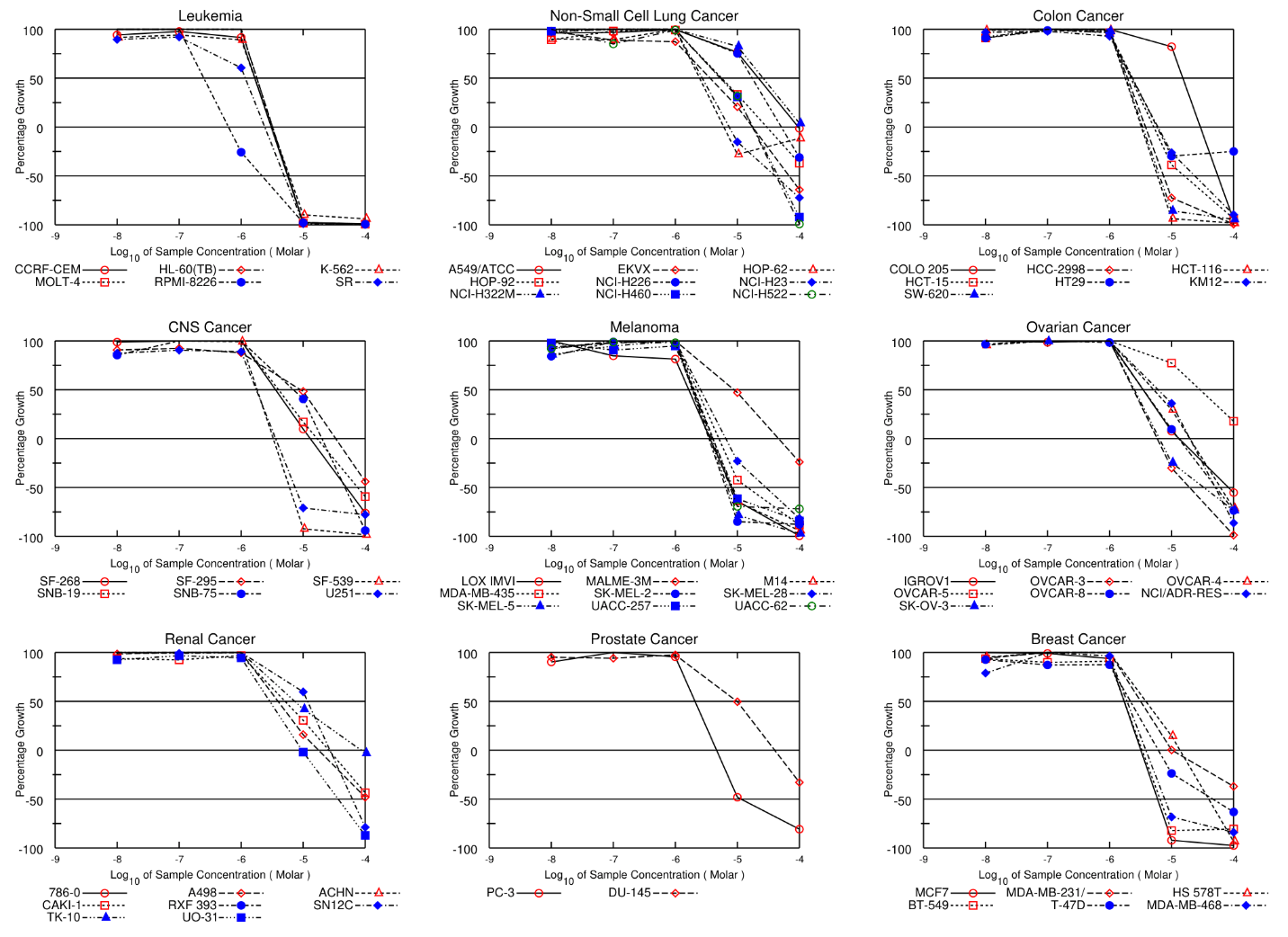


**Figure S76.** Dose response curves for compound **8o**

**Table S1:** NCI *in vitro* testing results of compound **8b** at five dose level in μM

| **Cancer Subpanel** | **Cell Line** | **8b** | | | | |
| --- | --- | --- | --- | --- | --- | --- |
|  |  | **GI_50_** | | | **TGI (Conc./cell line)** | **LC_50_ (Conc./cell line)** |
|  |  | **Conc./cell line** | **Subpanel MID^b^** | **Selectivity ratio (MID^a^/MID^b^)** |  |  |
| **Leukemia** | **CCRF-CEM** | 1.65 | 1.71 | 3.04 | 3.02 | 5.53 |
|  | **HL-60 (TB)** | 2.3 |  |  | 3.78 | 6.2 |
|  | **K-562** | 1.73 |  |  | 3.19 | 5.87 |
|  | **MOLT-4** | 1.81 |  |  | 3.24 | 5.79 |
|  | **RPMI-8226** | 1.43 |  |  | 2.75 | 5.28 |
|  | **SR** | 1.36 |  |  | 2.66 | 5.18 |
| **NSCLC** | **A549/ATCC** | 13.8 | 7.92 | 0.66 | 42.1 | ˃ 100 |
|  | **EKVX** | 3.9 |  |  | 15 | 48 |
|  | **HOP-62** | 2.58 |  |  | 5.94 | 27.6 |
|  | **HOP-92** | 4.74 |  |  | 27.2 | ˃ 100 |
|  | **NCI-H226** | 16.4 |  |  | 46.5 | ˃ 100 |
|  | **NCI-H23** | 2.95 |  |  | 8.95 | 32.8 |
|  | **NCI-H322M** | 18.6 |  |  | 73.1 | ˃ 100 |
|  | **NCI-H460** | 5.06 |  |  | 16.3 | 43.2 |
|  | **NCI-H522** | 3.23 |  |  | 11.9 | 34.7 |
| **Colon Cancer** | **COLO 205** | 17.1 | 4.25 | 1.23 | 31.2 | 57 |
|  | **HCC-2998** | 1.99 |  |  | 4.75 | 13.4 |
|  | **HCT-116** | 1.87 |  |  | 3.91 | 8.18 |
|  | **HCT-15** | 2.01 |  |  | 3.65 | 6.63 |
|  | **HT29** | 2.54 |  |  | 6.45 | ˃ 100 |
|  | **KM12** | 2.11 |  |  | 4.33 | 8.89 |
|  | **SW-620** | 2.14 |  |  | 4.65 | 10.3 |
| **CNS Cancer** | **SF-268** | 3.25 | 5.54 | 0.94 | 8.88 | ˃ 100 |
|  | **SF-295** | 14.3 |  |  | 46.7 | ˃ 100 |
|  | **SF-539** | 1.8 |  |  | 3.29 | 6 |
|  | **SNB-19** | 4.91 |  |  | 33 | ˃ 100 |
|  | **SNB-75** | 7.02 |  |  | 22 | 60.4 |
|  | **U251** | 1.95 |  |  | 4.26 | 9.29 |
| **Melanoma** | **LOX IMVI** | 1.8 | 3.76 | 1.39 | 4.09 | 9.32 |
|  | **MALME-3M** | 13.2 |  |  | 27.4 | 57.1 |
|  | **M14** | 2.8 |  |  | 6.58 | 25.4 |
|  | **MDA-MB-435** | 3.84 |  |  | 13.9 | ˃ 100 |
|  | **SK-MEL-2** | 1.83 |  |  | 3.46 | 6.54 |
|  | **SK-MEL-28** | 2.93 |  |  | 7.71 | 47.3 |
|  | **SK-MEL-5** | 3.19 |  |  | 11.3 | 33.9 |
|  | **UACC-257** | 2.02 |  |  | 3.83 | 7.27 |
|  | **UACC-62** | 2.22 |  |  | 5.25 | 21.9 |
| **Ovarian Cancer** | **IGROV1** | 3.42 | 8.32 | 0.63 | 15.4 | ˃ 100 |
|  | **OVCAR-3** | 3.17 |  |  | 7.5 | 27.2 |
|  | **OVCAR-4** | 14.4 |  |  | 41.4 | ˃ 100 |
|  | **OVCAR-5** | 26.2 |  |  | ˃ 100 | ˃ 100 |
|  | **OVCAR-8** | 2.75 |  |  | 8.73 | ˃ 100 |
|  | **NCI/ADR-RES** | 4.51 |  |  | 15.7 | 43.1 |
|  | **SK-OV-3** | 3.78 |  |  | 14.4 | ˃ 100 |
| **Renal Cancer** | **786-0** | ND | 6.99 | 0.75 | ND | ND |
|  | **A498** | 4.79 |  |  | 18.1 | 71.4 |
|  | **ACHN** | ND |  |  | ND | ND |
|  | **CAKI-1** | 6.66 |  |  | 29.9 | ˃ 100 |
|  | **RXF 393** | ND |  |  | ND | ND |
|  | **SN12C** | 11 |  |  | 23.6 | 50.5 |
|  | **TK-10** | 9.82 |  |  | 76.5 | ˃ 100 |
|  | **UO-31** | 2.68 |  |  | 7.9 | 30 |
| **Prostate Cancer** | **PC-3** | 2.19 | 4.59 | 1.14 | 4.73 | 11.1 |
|  | **DU-145** | 6.99 |  |  | 84 | ˃ 100 |
| **Breast Cancer** | **MCF7** | 2.07 | 2.76 | 1.89 | 5.79 | 29 |
|  | **MDA-MB-231/ATCC** | 3.84 |  |  | 19.8 | ˃ 100 |
|  | **HS 578T** | 3.29 |  |  | 9.63 | 35.9 |
|  | **BT-549** | 2.13 |  |  | 4.15 | 8.09 |
|  | **T-47D** | 3 |  |  | 12.4 | 42.1 |
|  | **MDA-MB-468** | 2.22 |  |  | 4.69 | 9.93 |
| **MID^a^** |  |  | 5.22 |  |  |  |

**Table S2:** NCI *in vitro* testing results of compound **8c** at five dose level in μM

| **Cancer Subpanel** | **Cell Line** | **8c** | | | | |
| --- | --- | --- | --- | --- | --- | --- |
|  |  | **GI_50_** | | | **TGI (Conc./cell line)** | **LC_50_ (Conc./cell line)** |
|  |  | **Conc./cell line** | **Subpanel MID^b^** | **Selectivity ratio (MID^a^/MID^b^)** |  |  |
| **Leukemia** | **CCRF-CEM** | 1.83 | 1.49 | 2.93 | 3.23 | 5.72 |
|  | **HL-60 (TB)** | 1.86 |  |  | 3.27 | 5.75 |
|  | **K-562** | 1.91 |  |  | 3.41 | 6.09 |
|  | **MOLT-4** | 1.79 |  |  | 3.18 | 5.65 |
|  | **RPMI-8226** | 0.24 |  |  | 0.52 | 1.34 |
|  | **SR** | 1.42 |  |  | 2.72 | 5.24 |
| **NSCLC** | **A549/ATCC** | 4.47 | 6.24 | 0.71 | ˃ 100 | ˃ 100 |
|  | **EKVX** | 1.12 |  |  | 2.4 | 5.15 |
|  | **HOP-62** | 2.53 |  |  | 7.17 | ˃ 100 |
|  | **HOP-92** | 3.34 |  |  | ND | ˃ 100 |
|  | **NCI-H226** | 2.83 |  |  | 6.59 | ˃ 100 |
|  | **NCI-H23** | 1.9 |  |  | 3.53 | 6.58 |
|  | **NCI-H322M** | 35.1 |  |  | ˃ 100 | ˃ 100 |
|  | **NCI-H460** | 2.21 |  |  | 4.11 | 7.65 |
|  | **NCI-H522** | 2.7 |  |  | 8.85 | 35.1 |
| **Colon Cancer** | **COLO 205** | 1.58 | 1.59 | 2.77 | 2.95 | 5.52 |
|  | **HCC-2998** | 1.71 |  |  | 3.19 | 5.95 |
|  | **HCT-116** | 1.25 |  |  | 2.53 | 5.13 |
|  | **HCT-15** | 1.65 |  |  | 3.19 | 6.15 |
|  | **HT29** | 2.16 |  |  | 5.05 | ˃ 100 |
|  | **KM12** | 1.11 |  |  | 2.41 | 5.2 |
|  | **SW-620** | 1.7 |  |  | 3.11 | 5.68 |
| **CNS Cancer** | **SF-268** | 2.03 | 2.38 | 1.86 | 5.1 | 21.2 |
|  | **SF-295** | 3.35 |  |  | 14.5 | ˃ 100 |
|  | **SF-539** | 1.44 |  |  | 2.76 | 5.3 |
|  | **SNB-19** | 2.63 |  |  | 7.79 | 93.7 |
|  | **SNB-75** | 2.96 |  |  | 8.86 | 31.9 |
|  | **U251** | 1.86 |  |  | 3.71 | 7.37 |
| **Melanoma** | **LOX IMVI** | 1.52 | 2.05 | 2.15 | 2.89 | 5.5 |
|  | **MALME-3M** | 3.56 |  |  | 7.73 | ˃ 100 |
|  | **M14** | 1.77 |  |  | 3.31 | 6.19 |
|  | **MDA-MB-435** | 2.15 |  |  | 4.01 | 7.49 |
|  | **SK-MEL-2** | 1.77 |  |  | 3.24 | 5.92 |
|  | **SK-MEL-28** | 2.17 |  |  | 4.62 | 9.81 |
|  | **SK-MEL-5** | 1.46 |  |  | 2.82 | 5.44 |
|  | **UACC-257** | 1.93 |  |  | 3.5 | 6.35 |
|  | **UACC-62** | 2.15 |  |  | 4.4 | 9.01 |
| **Ovarian Cancer** | **IGROV1** | 3.05 | 16.57 | 0.27 | 7.8 | ˃ 100 |
|  | **OVCAR-3** | 2.05 |  |  | 3.62 | 6.42 |
|  | **OVCAR-4** | 3.12 |  |  | 6.71 | 70.7 |
|  | **OVCAR-5** | ˃ 100 |  |  | ˃ 100 | ˃ 100 |
|  | **OVCAR-8** | 3.15 |  |  | 8.34 | ˃ 100 |
|  | **NCI/ADR-RES** | 2.52 |  |  | 5.68 | ˃ 100 |
|  | **SK-OV-3** | 2.12 |  |  | 4.9 | ˃ 100 |
| **Renal Cancer** | **786-0** | 2.02 | 2.75 | 1.61 | 4.1 | 8.32 |
|  | **A498** | 2.93 |  |  | 6.48 | ˃ 100 |
|  | **ACHN** | 2.7 |  |  | 7.35 | 36.4 |
|  | **CAKI-1** | 2.53 |  |  | 7.38 | 49.4 |
|  | **RXF 393** | ND |  |  | ND | ND |
|  | **SN12C** | 1.66 |  |  | 3.18 | 6.1 |
|  | **TK-10** | 5.45 |  |  | ˃ 100 | ˃ 100 |
|  | **UO-31** | 1.96 |  |  | 3.61 | 6.66 |
| **Prostate Cancer** | **PC-3** | 2.16 | 3.11 | 1.42 | 4 | 7.4 |
|  | **DU-145** | 4.05 |  |  | ˃ 100 | ˃ 100 |
| **Breast Cancer** | **MCF7** | 1.33 | 1.73 | 2.55 | 2.66 | 5.34 |
|  | **MDA-MB-231/ATCC** | 3.01 |  |  | 6.36 | ˃ 100 |
|  | **HS 578T** | 1.33 |  |  | 2.95 | 6.58 |
|  | **BT-549** | 1.87 |  |  | 3.38 | 6.11 |
|  | **T-47D** | 1.58 |  |  | 3.15 | 6.29 |
|  | **MDA-MB-468** | 1.27 |  |  | 2.61 | 5.37 |
| **MID^a^** |  |  | 4.42 |  |  |  |

**Table S3:** NCI *in vitro* testing results of compound **8d** at five dose level in μM

| **Cancer Subpanel** | **Cell Line** | **8d** | | | | |
| --- | --- | --- | --- | --- | --- | --- |
|  |  | **GI_50_** | | | **TGI (Conc./cell line)** | **LC_50_ (Conc./cell line)** |
|  |  | **Conc./cell line** | **Subpanel MID^b^** | **Selectivity ratio (MID^a^/MID^b^)** |  |  |
| **Leukemia** | **CCRF-CEM** | 1.83 | 1.49 | 2.3 | 3.24 | 5.72 |
|  | **HL-60 (TB)** | 1.47 |  |  | 2.81 | 5.36 |
|  | **K-562** | 1.78 |  |  | 3.36 | 6.35 |
|  | **MOLT-4** | 1.62 |  |  | 2.98 | 5.48 |
|  | **RPMI-8226** | 0.71 |  |  | 1.96 | 4.45 |
|  | **SR** | 1.52 |  |  | 2.85 | 5.37 |
| **NSCLC** | **A549/ATCC** | 7.47 | 5.63 | 0.61 | 50.3 | ˃ 100 |
|  | **EKVX** | 1.35 |  |  | 2.83 | 5.95 |
|  | **HOP-62** | 2.14 |  |  | 5.2 | 19.4 |
|  | **HOP-92** | 3.19 |  |  | ˃ 100 | ˃ 100 |
|  | **NCI-H226** | 3.31 |  |  | 28.1 | ˃ 100 |
|  | **NCI-H23** | 2.11 |  |  | 4.8 | 13.9 |
|  | **NCI-H322M** | 25.7 |  |  | ˃ 100 | ˃ 100 |
|  | **NCI-H460** | 3.16 |  |  | 10.1 | 33.5 |
|  | **NCI-H522** | 2.28 |  |  | 9.64 | 32.1 |
| **Colon Cancer** | **COLO 205** | 1.57 | 1.61 | 2.12 | 3.09 | 6.05 |
|  | **HCC-2998** | 1.48 |  |  | 2.82 | 5.38 |
|  | **HCT-116** | 1.41 |  |  | 2.74 | 5.33 |
|  | **HCT-15** | 1.6 |  |  | 3.34 | 6.98 |
|  | **HT29** | 2.02 |  |  | 4.28 | 9.07 |
|  | **KM12** | 1.5 |  |  | 2.99 | 5.96 |
|  | **SW-620** | 1.72 |  |  | 3.14 | 5.75 |
| **CNS Cancer** | **SF-268** | 2.63 | 3.29 | 1.04 | 9.47 | 40.7 |
|  | **SF-295** | 6.41 |  |  | 29.4 | ˃ 100 |
|  | **SF-539** | 1.31 |  |  | 2.64 | 5.33 |
|  | **SNB-19** | 3.14 |  |  | 11.3 | 39.5 |
|  | **SNB-75** | 4.03 |  |  | 14.1 | 39.2 |
|  | **U251** | 2.19 |  |  | 5.17 | 18.2 |
| **Melanoma** | **LOX IMVI** | 1.64 | 2.42 | 1.42 | 3.03 | 5.6 |
|  | **MALME-3M** | 6.66 |  |  | 26.8 | 97 |
|  | **M14** | 1.8 |  |  | 3.71 | 7.68 |
|  | **MDA-MB-435** | 2.09 |  |  | 5.35 | 19.2 |
|  | **SK-MEL-2** | 1.87 |  |  | 3.61 | 6.97 |
|  | **SK-MEL-28** | 2.05 |  |  | 4.02 | 7.87 |
|  | **SK-MEL-5** | 1.6 |  |  | 3.07 | 5.88 |
|  | **UACC-257** | 1.92 |  |  | 3.78 | 7.46 |
|  | **UACC-62** | 2.16 |  |  | 4.69 | 11.5 |
| **Ovarian Cancer** | **IGROV1** | 3.41 | 6.33 | 0.54 | 11.9 | 41.8 |
|  | **OVCAR-3** | 1.91 |  |  | 3.71 | 7.22 |
|  | **OVCAR-4** | 2.6 |  |  | 7.21 | 35 |
|  | **OVCAR-5** | 28.4 |  |  | ˃ 100 | ˃ 100 |
|  | **OVCAR-8** | 3.31 |  |  | 12.2 | ˃ 100 |
|  | **NCI/ADR-RES** | 2.55 |  |  | 7.55 | 60.6 |
|  | **SK-OV-3** | 2.15 |  |  | 5.05 | 15.6 |
| **Renal Cancer** | **786-0** | 2.16 | 3.66 | 0.94 | 5.06 | 15 |
|  | **A498** | 4.21 |  |  | 19.1 | ˃ 100 |
|  | **ACHN** | 4.47 |  |  | 18.9 | 60 |
|  | **CAKI-1** | 4.07 |  |  | 17 | 53.9 |
|  | **RXF 393** | ND |  |  | ND | ND |
|  | **SN12C** | 2.4 |  |  | 8.52 | 29.7 |
|  | **TK-10** | 5.64 |  |  | ˃ 100 | ˃ 100 |
|  | **UO-31** | 2.69 |  |  | 8.27 | 37.2 |
| **Prostate Cancer** | **PC-3** | 2.1 | 3.94 | 0.87 | 4.76 | 13.5 |
|  | **DU-145** | 5.77 |  |  | ˃ 100 | ˃ 100 |
| **Breast Cancer** | **MCF7** | 1.31 | 2.02 | 1.7 | 2.63 | 5.3 |
|  | **MDA-MB-231/ATCC** | 3.82 |  |  | 13.8 | 92.8 |
|  | **HS 578T** | 1.6 |  |  | 3.48 | 7.56 |
|  | **BT-549** | 1.74 |  |  | 3.37 | 6.55 |
|  | **T-47D** | 2.04 |  |  | 5.92 | 24 |
|  | **MDA-MB-468** | 1.58 |  |  | 3.38 | 7.21 |
| **MID^a^** |  |  | 3.43 |  |  |  |

**Table S4:** NCI *in vitro* testing results of compound **8m** at five dose level in μM

| **Cancer Subpanel** | **Cell Line** | **8m** | | | | |
| --- | --- | --- | --- | --- | --- | --- |
|  |  | **GI_50_** | | | **TGI (Conc./cell line)** | **LC_50_ (Conc./cell line)** |
|  |  | **Conc./cell line** | **Subpanel MID^b^** | **Selectivity ratio (MID^a^/MID^b^)** |  |  |
| **Leukemia** | **CCRF-CEM** | 1.42 | 1.34 | 1.88 | 2.72 | 5.25 |
|  | **HL-60 (TB)** | 1.84 |  |  | 3.25 | 5.74 |
|  | **K-562** | 1.51 |  |  | 2.89 | 5.5 |
|  | **MOLT-4** | 1.63 |  |  | 2.99 | 5.5 |
|  | **RPMI-8226** | 0.54 |  |  | 1.89 | 4.36 |
|  | **SR** | 1.07 |  |  | 2.26 | 4.78 |
| **NSCLC** | **A549/ATCC** | 6.28 | 4.03 | 0.62 | 41.6 | ˃ 100 |
|  | **EKVX** | 2.24 |  |  | 6.21 | 28.1 |
|  | **HOP-62** | ND |  |  | 4.34 | 9.78 |
|  | **HOP-92** | 2.36 |  |  | 8.06 | 62.1 |
|  | **NCI-H226** | 3.59 |  |  | 14.4 | 52.5 |
|  | **NCI-H23** | 2.18 |  |  | 4.7 | 10.7 |
|  | **NCI-H322M** | 10.1 |  |  | 25.4 | 64.3 |
|  | **NCI-H460** | 3.44 |  |  | 11.8 | 34.8 |
|  | **NCI-H522** | 2.06 |  |  | 4.86 | 13.8 |
| **Colon Cancer** | **COLO 205** | 6.44 | 2.25 | 1.11 | 19.2 | 43.9 |
|  | **HCC-2998** | 1.34 |  |  | 2.66 | 5.27 |
|  | **HCT-116** | 1.45 |  |  | 2.97 | 6.05 |
|  | **HCT-15** | 1.51 |  |  | 3.22 | 6.87 |
|  | **HT29** | 2.1 |  |  | ND | ˃ 100 |
|  | **KM12** | 1.28 |  |  | 2.84 | 6.28 |
|  | **SW-620** | 1.66 |  |  | 3.13 | 5.92 |
| **CNS Cancer** | **SF-268** | 2.36 | 2.58 | 0.97 | 5.23 | 17 |
|  | **SF-295** | 4.51 |  |  | 19 | 66.5 |
|  | **SF-539** | 1.54 |  |  | 2.9 | 5.45 |
|  | **SNB-19** | 2.53 |  |  | 7.06 | 39.3 |
|  | **SNB-75** | 2.85 |  |  | 8.57 | 31 |
|  | **U251** | 1.68 |  |  | 3.38 | 6.8 |
| **Melanoma** | **LOX IMVI** | 1.83 | 1.93 | 1.3 | 3.77 | 7.78 |
|  | **MALME-3M** | 2.39 |  |  | 8.18 | 34.7 |
|  | **M14** | 1.73 |  |  | 3.29 | 6.24 |
|  | **MDA-MB-435** | 2.49 |  |  | 7.29 | 33.3 |
|  | **SK-MEL-2** | 1.76 |  |  | 3.26 | 6.05 |
|  | **SK-MEL-28** | 2.34 |  |  | 5.41 | 19.6 |
|  | **SK-MEL-5** | 1.57 |  |  | 3.4 | 7.34 |
|  | **UACC-257** | 1.51 |  |  | 2.96 | 5.81 |
|  | **UACC-62** | 1.71 |  |  | 3.69 | 7.97 |
| **Ovarian Cancer** | **IGROV1** | 2.37 | 2.94 | 0.85 | 6.75 | 25.3 |
|  | **OVCAR-3** | 1.78 |  |  | 3.42 | 6.59 |
|  | **OVCAR-4** | 2.27 |  |  | 6.97 | 36.6 |
|  | **OVCAR-5** | 6.31 |  |  | 84.8 | ˃ 100 |
|  | **OVCAR-8** | 2.13 |  |  | 4.72 | 12.2 |
|  | **NCI/ADR-RES** | 2.8 |  |  | 8.26 | 34.5 |
|  | **SK-OV-3** | 2.93 |  |  | 6.01 | 20.9 |
| **Renal Cancer** | **786-0** | ND | 2.83 | 0.88 | ND | ND |
|  | **A498** | 2.9 |  |  | 8.85 | 67.8 |
|  | **ACHN** | ND |  |  | ND | ND |
|  | **CAKI-1** | 3.48 |  |  | 14 | 64.6 |
|  | **RXF 393** | ND |  |  | ND | ND |
|  | **SN12C** | 3.25 |  |  | 10.3 | 32.6 |
|  | **TK-10** | 2.74 |  |  | 6.54 | 24.1 |
|  | **UO-31** | 1.8 |  |  | 3.36 | 6.29 |
| **Prostate Cancer** | **PC-3** | 1.63 | 2.64 | 0.95 | 3.26 | 6.49 |
|  | **DU-145** | 3.64 |  |  | 35.7 | ˃ 100 |
| **Breast Cancer** | **MCF7** | 1.73 | 1.9 | 1.32 | 3.7 | 7.89 |
|  | **MDA-MB-231/ATCC** | 2.42 |  |  | 7.73 | ˃ 100 |
|  | **HS 578T** | 2.13 |  |  | 4.9 | 13.6 |
|  | **BT-549** | 1.83 |  |  | 3.41 | 6.35 |
|  | **T-47D** | 2.1 |  |  | 5.6 | 20.5 |
|  | **MDA-MB-468** | 1.21 |  |  | 2.86 | 6.78 |
| **MID^a^** |  |  | 2.51 |  |  |  |

**Table S5:** NCI *in vitro* testing results of compound **8o** at five dose level in μM

| **Cancer Subpanel** | **Cell Line** | **8o** | | | | |
| --- | --- | --- | --- | --- | --- | --- |
|  |  | **GI_50_** | | | **TGI (Conc./cell line)** | **LC_50_ (Conc./cell line)** |
|  |  | **Conc./cell line** | **Subpanel MID^b^** | **Selectivity ratio (MID^a^/MID^b^)** |  |  |
| **Leukemia** | **CCRF-CEM** | 1.66 | 1.52 | 3.4 | 3.05 | 5.6 |
|  | **HL-60 (TB)** | 2.32 |  |  | 3.81 | 6.24 |
|  | **K-562** | 1.66 |  |  | 3.15 | 6 |
|  | **MOLT-4** | 1.98 |  |  | 3.42 | 5.9 |
|  | **RPMI-8226** | 0.3 |  |  | 0.66 | 2.17 |
|  | **SR** | 1.17 |  |  | 2.4 | 4.94 |
| **NSCLC** | **A549/ATCC** | 21.9 | 10.14 | 0.51 | 96 | ˃ 100 |
|  | **EKVX** | 3.63 |  |  | 17.5 | 68.2 |
|  | **HOP-62** | 2.68 |  |  | 6.25 | ˃ 100 |
|  | **HOP-92** | 5.58 |  |  | 29.8 | ˃ 100 |
|  | **NCI-H226** | 17.3 |  |  | 51 | ˃ 100 |
|  | **NCI-H23** | 3.08 |  |  | 7.59 | 40.6 |
|  | **NCI-H322M** | 25.9 |  |  | ˃ 100 | ˃ 100 |
|  | **NCI-H460** | 5.78 |  |  | 17.8 | 45.4 |
|  | **NCI-H522** | 5.38 |  |  | 17.5 | 42.1 |
| **Colon Cancer** | **COLO 205** | 15.2 | 3.96 | 1.3 | 29.1 | 55.7 |
|  | **HCC-2998** | 2.02 |  |  | 3.88 | 7.46 |
|  | **HCT-116** | 1.8 |  |  | 3.26 | 5.93 |
|  | **HCT-15** | 2.21 |  |  | 5.17 | 16.1 |
|  | **HT29** | 2.35 |  |  | 5.83 | ˃ 100 |
|  | **KM12** | 2.29 |  |  | 6.03 | 23.7 |
|  | **SW-620** | 1.83 |  |  | 3.42 | 6.4 |
| **CNS Cancer** | **SF-268** | 4.07 | 4.79 | 1.08 | 13 | 49.7 |
|  | **SF-295** | 9.07 |  |  | 33.4 | ˃ 100 |
|  | **SF-539** | 1.81 |  |  | 3.3 | 6.02 |
|  | **SNB-19** | 4.56 |  |  | 16.8 | 75.6 |
|  | **SNB-75** | 7.48 |  |  | 20 | 47.1 |
|  | **U251** | 1.75 |  |  | 3.6 | 7.4 |
| **Melanoma** | **LOX IMVI** | 1.65 | 2.87 | 1.79 | 3.63 | 8.02 |
|  | **MALME-3M** | 9.02 |  |  | 46.1 | ˃ 100 |
|  | **M14** | 2.13 |  |  | 4.18 | 8.19 |
|  | **MDA-MB-435** | 2.6 |  |  | 5.39 | 14.7 |
|  | **SK-MEL-2** | 2 |  |  | 3.64 | 6.6 |
|  | **SK-MEL-28** | 2.67 |  |  | 6.6 | 28.9 |
|  | **SK-MEL-5** | 1.93 |  |  | 3.66 | 6.96 |
|  | **UACC-257** | 1.93 |  |  | 4.04 | 8.44 |
|  | **UACC-62** | 1.94 |  |  | 3.85 | 7.66 |
| **Ovarian Cancer** | **IGROV1** | 3.57 | 7.6 | 0.68 | 13.2 | 82.6 |
|  | **OVCAR-3** | 2.84 |  |  | 6.23 | 19.5 |
|  | **OVCAR-4** | 5.61 |  |  | 19.7 | 61.6 |
|  | **OVCAR-5** | 28.8 |  |  | ˃ 100 | ˃ 100 |
|  | **OVCAR-8** | 3.5 |  |  | 13 | 52.1 |
|  | **NCI/ADR-RES** | 6.24 |  |  | 19.7 | 50.5 |
|  | **SK-OV-3** | 2.65 |  |  | 6.46 | 33.1 |
| **Renal Cancer** | **786-0** | ND | 6.31 | 0.82 | ND | ND |
|  | **A498** | 4.22 |  |  | 17.7 | ˃ 100 |
|  | **ACHN** | ND |  |  | ND | ND |
|  | **CAKI-1** | 5.1 |  |  | 25.9 | ˃ 100 |
|  | **RXF 393** | ND |  |  | ND | ND |
|  | **SN12C** | 11.8 |  |  | 27 | 62 |
|  | **TK-10** | 7.54 |  |  | 86.2 | ˃ 100 |
|  | **UO-31** | 2.89 |  |  | 9.55 | 36.7 |
| **Prostate Cancer** | **PC-3** | 2.07 | 5.94 | 0.87 | 4.63 | 11.4 |
|  | **DU-145** | 9.8 |  |  | 39.9 | ˃ 100 |
| **Breast Cancer** | **MCF7** | 1.72 | 2.44 | 2.12 | 3.2 | 5.94 |
|  | **MDA-MB-231/ATCC** | 3.19 |  |  | 10.2 | ˃ 100 |
|  | **HS 578T** | 3.89 |  |  | 13.7 | 39.7 |
|  | **BT-549** | 1.73 |  |  | 3.35 | 6.52 |
|  | **T-47D** | 2.17 |  |  | 6.12 | 46.3 |
|  | **MDA-MB-468** | 1.91 |  |  | 3.85 | 7.74 |
| **MID^a^** |  |  | 5.16 |  |  |  |

**Molecular Docking**

The crystal structure of tubulin-colchicine complex (PDB code: 4O2B) was downloaded from the Protein Data Bank. Structures of compounds **8m** and **8d** were drawn and optimized using Marvin Sketch and Avogadro molecular editors. The protein was prepared using Autodock tools where the co-crystallized combretastatin A-4 and water molecules were removed then kollman charges and polar hydrogens were added. The grid dimensions for tubulin were set to 80x80x80. Autodock vina was used for molecular docking and the best docking poses were visualized using Discovery Studio Visualizer

**ADMET prediction**

The absorption, distribution, metabolism, excretion, and toxicity (ADMET) profile of compound **8m** was predicted using ADMETlab 3.0 (<https://admetmesh.scbdd.com/>), an integrated online platform that combines large curated datasets with multi-task graph neural network models for drug property assessment. The canonical SMILES of **8m** was submitted to the server, and predictions were generated across a wide range of pharmacokinetic and toxicity endpoints. The evaluation encompassed physicochemical descriptors, including molecular weight, topological polar surface area, lipophilicity, solubility, and flexibility, alongside drug-likeness assessment according to Lipinski’s rule of five, Pfizer 3/75, GSK, and Golden Triangle filters. Additional indices such as synthetic accessibility score, PAINS and BMS alerts, and a bioavailability radar were also examined. Absorption was assessed through predicted intestinal uptake, Caco-2, MDCK, and PAMPA permeabilities, as well as P-glycoprotein substrate and inhibition potential. Distribution parameters included plasma protein binding, volume of distribution, and blood–brain barrier penetration. The metabolic profile was predicted by evaluating substrate and inhibition tendencies against major cytochrome P450 isoforms (CYP1A2, CYP2C9, CYP2C19, CYP2D6, CYP3A4), together with hepatic microsomal stability. Excretion-related properties, such as clearance and half-life, were also estimated. Toxicological predictions comprised hERG inhibition liability, hepatotoxicity, drug-induced liver injury (DILI), genotoxicity, carcinogenicity, hematotoxicity, ototoxicity, and disruption of mitochondrial membrane potential, in addition to toxicophoric alerts. All predictions were obtained using the platform’s default settings, and the results were analyzed to provide a comprehensive in silico ADMET profile for compound **8m**, complementing the experimental findings.

**Table S1.** Docking interactions of compounds **8m** and **8d**

| **Compound** | **Binding Affinity (kcal/mol)** | **Classical Hydrogen Bonding** | **Non-Classical Hydrogen Bonding** | **Hydrophobic Interactions** |
| --- | --- | --- | --- | --- |
| **8m** | -9.2 | Ala317, Ala354 | Gln247, Tyr202, Val238 | Leu248, Leu255, Ala316, Tyr224, Ala250, Ala354, Cys241, Leu242, Leu252, Lys352, Val238 |
| **8d** | -8.3 | Ala317 | NA | Leu248, Leu255, Ala354, Lys352, Cys241 |

**Table S2.** Physicochemical Properties of compounds **8m** and **8d**

| **Property** | **8m** | **8d** | **Comment** |
| --- | --- | --- | --- |
| Molecular Weight | 439.09 | 397.06 | Contain hydrogen atoms. Optimal:100~600 |
| Volume | 434.869 | 388.765 | Van der Waals volume |
| Density | 1.01 | 1.021 | Density = MW / Volume |
| nHA | 5.0 | 3.0 | Number of hydrogen bond acceptors. Optimal:0~12 |
| nHD | 0.0 | 0.0 | Number of hydrogen bond donors. Optimal:0~7 |
| nRot | 9.0 | 7.0 | Number of rotatable bonds. Optimal:0~11 |
| nRing | 3.0 | 3.0 | Number of rings. Optimal:0~6 |
| MaxRing | 6.0 | 6.0 | Number of atoms in the biggest ring. Optimal:0~18 |
| nHet | 7.0 | 6.0 | Number of heteroatoms. Optimal:1~15 |
| fChar | 0.0 | 0.0 | Formal charge. Optimal:-4 ~4 |
| nRig | 20.0 | 20.0 | Number of rigid bonds. Optimal:0~30 |
| Flexibility | 0.45 | 0.35 | Flexibility = nRot /nRig |
| Stereo Centers | 0.0 | 0.0 | Stereo Centers. Optimal:  2 |
| TPSA | 65.49 | 47.03 | Topological Polar Surface Area. Optimal:0~140 |
| logS | -5.007 | -4.917 | The logarithm of aqueous solubility value. |
| logP | 3.377 | 3.647 | The logarithm of the n-octanol/water distribution coefficients at pH=7.4. |
| logD | 3.427 | 3.631 | The logarithm of the n-octanol/water distribution coefficient. |
| pka (Acid) | 8.976 | 8.919 | Acid-base dissociation constant (pKa) value represents the strength of a drug molecule's acidity or basicity. |
| pka (Base) | 3.315 | 3.755 | Acid-base dissociation constant (pKa) value represents the strength of a drug molecule's acidity or basicity. |
| Melting point | 115.82 | 103.271 | The predicted melting point of a compound is expressed in degrees Celsius (°C).  Melting points below 25°C are classified as liquids, while melting points above 25°C are classified as solids. |
| Boiling point | 335.351 | 330.657 | The predicted melting point of a compound is expressed in degrees Celsius (°C).  A normal boiling point below 25°C is categorized as a gas. |

**Table S3.** Medicinal Chemistry of compounds **8m** and **8d**

| **Property** | **8m** | **8d** | **Comment** |
| --- | --- | --- | --- |
| QED | 0.255 | 0.302 | - A measure of drug-likeness based on the concept of desirability; - Attractive: > 0.67; - unattractive: 0.49~0.67; - too complex: < 0.34 |
| GASA | 0.0 | 0.0 | - ES: Easy to synthesize; HS: Hard to synthesize; - The output value represents the probability of being difficult to synthesize, ranging from 0 to 1. |
| Synth | 2.0 | 2.0 | - Synthetic accessibility score is designed to estimate ease of synthesis of drug-like molecules. - SAscore ≥ 6, difficult to synthesize; SAscore <6, easy to synthesize |
| Fsp3 | 0.174 | 0.095 | - The number of sp3 hybridized carbons / total carbon count, correlating with melting point and solubility. - Fsp3 ≥0.42 is considered a suitable value. |
| MCE-18 | 18.0 | 17.0 | - MCE-18 stands for medicinal chemistry evolution. - MCE-18≥45 is considered a suitable value. |
| NPscore | -1.073 | -1.5 | - Natural product-likeness score. - This score is typically in the range from -5 to 5. - The higher the score is, the higher the probability is that the molecule is a NP. |
| Lipinski Rule | 0.0 | 0.0 | - MW  500; logP  5; Hacc  10; Hdon ≤ 5 - If two properties are out of range, a poor absorption or permeability is possible, one is acceptable. |
| Pfizer Rule | 1.0 | 1.0 | - logP > 3; TPSA < 75 - Compounds with a high log P (>3) and low TPSA (<75) are likely to be toxic. |
| GSK Rule | 1.0 | 0.0 | - MW  400; logP ≤ 4 - Compounds satisfying the GSK rule may have a more favorable ADMET profile |
| Golden Triangle | 0.0 | 0.0 | - 200  MW  500; -2  logD ≤ 5 - Compounds satisfying the Golden Triangle rule may have a more favorable ADMET profile. |
| PAINS | 0 alerts | 0 alerts | frequent hitters, Alpha-screen artifacts and reactive compound 480 substructures (J Med Chem 201053:2719-40) |
| ALARM NMR | 7 alerts | 5 alerts | Thiol reactive compounds. |
| BMS | 0 alerts | 0 alerts | undesirable, reactive compounds 176 substructures (J Chem Inf Model 200646:1060-8) |
| Chelator Rule | 0 alerts | 0 alerts | Chelating compounds. |
| Colloidal aggregators | 1.0 | 1.0 | - Category 0: non-colloidal aggregators; - Category 1: colloidal aggregators. - The output value is the probability of being colloidal aggregators, within the range of 0 to 1. |
| FLuc inhibitors | 0.999 | 0.999 | - Category 0: non-fLuc inhibitors; - Category 1: fLuc inhibitors.   The output value is the probability of being fLuc inhibitors, within the range of 0 to 1. |
| Blue fluorescence | 0.534 | 0.211 | - Category 0: non-blue fluorescence; - Category 1: blue fluorescence.   The output value is the probability of being blue fluorescence, within the range of 0 to 1. |
| Green fluorescence | 1.0 | 0.999 | - Category 0: non-green fluorescence; - Category 1: green fluorescence.   The output value is the probability of being green fluorescence, within the range of 0 to 1. |
| Reactive compounds | 0.071 | 0.065 | - Category 0: non-reactive compound; - Category 1: reactive compound.   The output value is the probability of being reactive compound, within the range of 0 to 1. |
| Promiscuous compounds | 0.026 | 0.001 | - Category 0: non-promiscuous compound; - Category 1: promiscuous compound.   The output value is the probability of being promiscuous compound, within the range of 0 to 1. |

**Table S4.** Absorption of compounds **8m** and **8d**

| **Property** | **8m** | **8d** | **Comment** |
| --- | --- | --- | --- |
| Caco-2 Permeability | -4.8 | -4.892 | Optimal: higher than -5.15 Log unit |
| MDCK  Permeability | -4.688 | -4.586 | - low permeability: < 2 × 10-6 cm/s - medium permeability: 2-20 × 10-6 cm/s - high passive permeability: > 20 × 10-6 cm/s |
| PAMPA | 0.002 | 0.005 | - The experimental data for Peff was logarithmically transformed (logPeff). - Molecules with log Peff values below 2.0 were classified as low-permeability (Category 0), while those with log Peff values exceeding 2.5 were classified as high-permeability (Category 1). |
| Pgp-inhibitor | 1.0 | 1.0 | - Category 1: Inhibitor; - Category 0: Non-inhibitor; - The output value is the probability of being Pgp-inhibitor |
| Pgp-substrate | 0.0 | 0.0 | - Category 1: substrate; - Category 0: Non-substrate; - The output value is the probability of being Pgp-substrate |
| HIA | 0.434 | 0.002 | - Human Intestinal Absorption - Category 1: HIA+( HIA < 30%); - Category 0: HIA-( HIA >= 30%); - The output value is the probability of being HIA+ |
| F20% | 0.021 | 0.0 | - 20% Bioavailability - Category 1: F 20% + (bioavailability < 20%); - Category 0: F 20% - (bioavailability ≥ 20%); - The output value is the probability of being F 20%   + |
| F30% | 0.984 | 0.559 | - 30% Bioavailability - Category 1: F 30% + (bioavailability < 30%); - Category 0: F 30% - (bioavailability ≥ 30%); - The output value is the probability of being F 30%   + |
| F50% | 0.945 | 0.129 | - 50% Bioavailability - Category 1: F 50% + (bioavailability < 50%); - Category 0: F 50% - (bioavailability ≥ 50%); - The output value is the probability of being F 50%   + |

**Table S5.** Distribution of compounds **8m** and **8d**

| **Property** | **8m** | **8d** | **Comment** |
| --- | --- | --- | --- |
| PPB | 98.278 | 98.592 | - Plasma Protein Binding Optimal: < 90%. - Drugs with high protein-bound may have a low therapeutic index. |
| VDss | -0.119 | 0.003 | - Volume Distribution - Optimal: 0.04-20L/kg |
| BBB | 0.0 | 0.037 | - Blood-Brain Barrier Penetration - Category 1: BBB+; Category 0: BBB-; - The output value is the probability of being BBB+ |
| Fu | 1.675 | 1.246 | - The fraction unbound in plasms - Low: <5%; Middle: 5~20%; High: > 20% |
| OATP1B1  inhibitor | 1.0 | 1.0 | - Category 0: Non-inhibitor; Category 1: inhibitor. - The output value is the probability of being inhibitor, within the range of 0 to 1. |
| OATP1B3  inhibitor | 1.0 | 1.0 | - Category 0: Non-inhibitor; Category 1: inhibitor. - The output value is the probability of being inhibitor, within the range of 0 to 1. |
| BCRP  inhibitor | 0.0 | 0.0 | - Category 0: Non-inhibitor; Category 1: inhibitor. - The output value is the probability of being inhibitor, within the range of 0 to 1. |
| MRP1  inhibitor | 0.014 | 0.559 | - Category 0: Non-inhibitor; Category 1: inhibitor. - The output value is the probability of being inhibitor, within the range of 0 to 1. |

**Table S6.** Metabolism of compounds **8m** and **8d**

| **Property** | **8m** | **8d** | **Comment** |
| --- | --- | --- | --- |
| CYP1A2  inhibitor | 1.0 | 1.0 | - Category 1: Inhibitor; Category 0: Non-inhibitor - The output value is the probability of being inhibitor. |
| CYP1A2 substrate | 0.016 | 0.0 | - Category 1: Substrate; Category 0: Non-substrate   The output value is the probability of being substrate. |
| CYP2C19 inhibitor | 1.0 | 0.998 | - Category 1: Inhibitor; Category 0: Non-inhibitor   The output value is the probability of being inhibitor. |
| CYP2C19 substrate | 0.001 | 0.0 | - Category 1: Substrate; Category 0: Non-substrate   The output value is the probability of being substrate. |
| CYP2C9 inhibitor | 1.0 | 0.997 | - Category 1: Inhibitor; Category 0: Non-inhibitor   The output value is the probability of being inhibitor. |
| CYP2C9 substrate | 0.112 | 0.005 | - Category 1: Substrate; Category 0: Non-substrate   The output value is the probability of being substrate. |
| CYP2D6 inhibitor | 0.181 | 0.131 | - Category 1: Inhibitor; Category 0: Non-inhibitor   The output value is the probability of being inhibitor. |
| CYP2D6 substrate | 0.644 | 0.003 | - Category 1: Substrate; Category 0: Non-substrate   The output value is the probability of being substrate. |
| CYP3A4 inhibitor | 0.995 | 0.604 | - Category 1: Inhibitor; Category 0: Non-inhibitor   The output value is the probability of being inhibitor. |
| CYP3A4 substrate | 0.0 | 0.0 | - Category 1: Substrate; Category 0: Non-substrate   The output value is the probability of being substrate. |
| CYP2B6 inhibitor | 0.997 | 1.0 | - Category 1: Inhibitor; Category 0: Non-inhibitor   The output value is the probability of being inhibitor. |
| CYP2B6 substrate | 0.0 | 0.0 | - Category 1: Substrate; Category 0: Non-substrate;   The output value is the probability of being substrate. |
| CYP2C8 inhibitor | 1.0 | 1.0 | - Category 1: Inhibitor; Category 0: Non-inhibitor;   The output value is the probability of being inhibitor. |
| HLM Stability | 0.999 | 0.402 | - human liver microsomal (HLM) stability   Category 0: stable+ (HLM > 30 min); Category 1: unstable- ( HLM ≤ 30 min). The output value is the probability of human liver microsomal instability, where a value closer to 1 indicates a higher likelihood of instability. The range is between 0 and 1. |

**Table S7.** Excretion of compounds **8m** and **8d**

| **Property** | **8m** | **8d** | **Comment** |
| --- | --- | --- | --- |
| CLplasma | 6.296 | 4.865 | - The unit of predicted CLplasma penetration is ml/min/kg. >15 ml/min/kg: high clearance; 5-15 ml/min/kg: moderate clearance; < 5 ml/min/kg: low clearance. |
| T1/2 | 0.585 | 0.613 | - The unit of predicted T1/2 is hours. - ultra-short half-life drugs: 1/2 < 1 hour; short   half-life drugs: T1/2 between 1-4 hours; intermediate short half-life drugs: T1/2 between 4-8 hours; long half-life drugs: T1/2 > 8 hours. |

**Table S8.** Toxicity of compounds **8m** and **8d**

| **Property** | **8m** | **8d** | **Comment** |
| --- | --- | --- | --- |
| hERG  Blockers | 0.587 | 0.422 | - Molecules with IC50 10μM or ≥50% inhibition at 10 μM were classified as hERG+ (Category 1), - while molecules with IC50 >10μM or < 50% inhibition at 10μM were classified as hERG - (Category 0). - The output value is the probability of being hERG+, within the range of 0 to 1. |
| hERG  Blockers (10um) | 0.586 | 0.676 | - Molecules with IC50 10 μM are classified as hERG+ (Category 1), - and molecules with IC50 > 10μM are classified as hERG- (Category 0). - The output value is the probability of being hERG+, within the range of 0 to 1. |
| DILI | 0.993 | 0.964 | - Drug Induced Liver Injury. - Category 1: drugs with a high risk of DILI; - Category 0: drugs with no risk of DILI. - The output value is the probability of being toxic. |
| AMES Mutagenicity | 0.536 | 0.37 | - AMES Toxicity - Category 1: Ames positive(+); - Category 0: Ames negative(-); - The output value is the probability of being toxic. |
| Rat Oral Acute Toxicity | 0.116 | 0.323 | - Rat Oral Acute Toxicity. - Category 0: low-toxicity, > 500 mg/kg; - Category 1: high-toxicity; < 500 mg/kg. - The output value is the probability of being toxic, within the range of 0 to 1. |
| FDAMDD | 0.136 | 0.203 | - FDA Maximum (Recommended) Daily Dose. - Category 1: FDAMDD (+); - Category 0: FDAMDD (-);   The output value is the probability of being positive. |
| Skin Sensitization | 0.95 | 0.977 | - Category 1: Sensitizer; - Category 0: Non-sensitizer. - The output value is the probability of being toxic, within the range of 0 to 1. |
| Carcinogenicity | 0.492 | 0.285 | - Category 1: carcinogens; - Category 0: non-carcinogens; - The output value is the probability of being toxic. |
| Eye Corrosion | 0.002 | 0.005 | - Eye Corrosion - Category 1: corrosives; Category 0: |
| Eye Irritation | 0.676 | 0.763 | - Eye Irritation - Category 1: irritants; Category 0: nonirritants;   The output value is the probability of being irritants. |
| Respiratory | 0.947 | 0.949 | - Category 1: respiratory toxicants; - Category 0: non-respiratory toxicants.   The output value is the probability of being toxic, within the range of 0 to 1. |
| Human Hepatotoxicity | 0.363 | 0.287 | - Human Hepatotoxicity - Category 1: H-HT positive(+); - Category 0: H-HT negative(-);   The output value is the probability of being toxic. |
| Drug-induced Nephrotoxicity | 0.691 | 0.783 | - Category 0: non-nephrotoxic (-); - Category 1: nephrotoxic (+).   The output value is the probability of being nephrotoxic (+), within the range of 0 to 1. |
| Ototoxicity | 0.187 | 0.107 | - Category 0: non-ototoxicity (-); - Category 1: ototoxicity (+).   The output value is the probability of being ototoxicity (+), within the range of 0 to 1. |
| Hematotoxicity | 0.336 | 0.232 | - Category 0: non-hematotoxicity (-); - Category 1: hematotoxicity (+).   The output value is the probability of being hematotoxicity (+), within the range of 0 to 1. |
| Genotoxicity | 0.713 | 0.962 | - Category 0: non-Genotoxicity (-); - Category 1: Genotoxicity (+).   The output value is the probability of being ototoxicity (+), within the range of 0 to 1. |
| RPMI-8226 Immunotoxicity | 0.055 | 0.018 | - Category 0: non-cytotoxicity (-); - Category 1: cytotoxicity (+).   The output value is the probability of being ototoxicity (+), within the range of 0 to 1. |
| A549 Cytotoxicity | 0.114 | 0.127 | - Category 0: non-cytotoxicity (-); - Category 1: cytotoxicity (+).   The output value is the probability of being ototoxicity (+), within the range of 0 to 1. |
| Hek293 Cytotoxicity | 0.668 | 0.665 | - Category 0: non-cytotoxicity (-); - Category 1: cytotoxicity (+).   The output value is the probability of being ototoxicity (+), within the range of 0 to 1. |
| Drug-induce d Neurotoxicity | 0.737 | 0.778 | - Category 0: non-neurotoxic (-); - Category 1: neurotoxic (+).   The output value is the probability of being neurotoxic (+), within the range of 0 to 1. |

**Table S9.** Environmental toxicity of compounds **8m** and **8d**

| **Property** | **8m** | **8d** | **Comment** |
| --- | --- | --- | --- |
| Bioconcentration Factors | 1.284 | 1.339 | - Bioconcentration factors are used for considering secondary poisoning potential and assessing risks to human health via the food chain.   The unit is −log10[(mg/L)/(1000*MW)] |
| IGC50 | 4.858 | 4.643 | - Tetrahymena pyriformis 50 percent growth inhibition concentration.   The unit is −log10[(mg/L)/(1000*MW)] |
| LC50FM | 6.282 | 5.919 | - 96-hour fathead minnow 50 percent lethal concentration.   The unit is −log10[(mg/L)/(1000*MW)] |
| LC50DM | 6.472 | 6.125 | - 48-hour daphnia magna 50 percent lethal concentration.   The unit is −log10[(mg/L)/(1000*MW)] |

**Table S10.** Tox21 pathway of compounds **8m** and **8d**

| **Property** | **8m** | **8d** | **Comment** |
| --- | --- | --- | --- |
| NR-AhR | 0.505 | 0.152 | - Aryl hydrocarbon receptor - Category 1: actives ; - Category 0: inactives; - The output value is the probability of being active. |
| NR-AR | 0.0 | 0.0 | - Androgen receptor - Category 1: actives ; - Category 0: inactives; - The output value is the probability of being active. |
| NR-AR-LBD | 0.048 | 0.089 | - Androgen receptor ligand-binding domain - Category 1: actives ; - Category 0: inactives; - The output value is the probability of being active. |
| NR-Aromatase | 0.004 | 0.028 | - Category 1: actives ; - Category 0: inactives; - The output value is the probability of being active. |
| NR-ER | 0.581 | 0.604 | - Estrogen receptor - Category 1: actives ; - Category 0: inactives; - The output value is the probability of being active. |
| NR-ER-LBD | 0.0 | 0.001 | - Estrogen receptor ligand-binding domain - Category 1: actives ; - Category 0: inactives; - The output value is the probability of being active. |
| NR-PPAR-gamma | 0.011 | 0.097 | - Peroxisome proliferator-activated receptor gamma - Category 1: actives ; - Category 0: inactives; - The output value is the probability of being active. |
| SR-ARE | 0.741 | 0.947 | - Antioxidant response element - Category 1: actives ; - Category 0: inactives; - The output value is the probability of being active. |
| SR-ATAD5 | 0.046 | 0.07 | - ATPase family AAA domain-containing protein 5 - Category 1: actives ; - Category 0: inactives;   The output value is the probability of being active. |
| SR-HSE | 0.749 | 0.87 | - Heat shock factor response element - Category 1: actives ; - Category 0: inactives;   The output value is the probability of being active. |
| SR-MMP | 0.357 | 0.851 | - Mitochondrial membrane potential - Category 1: actives ; - Category 0: inactives;   The output value is the probability of being active. |
| SR-p53 | 0.897 | 0.838 | - p53, a tumor suppressor protein - Category 1: actives ; - Category 0: inactives;   The output value is the probability of being active. |

**Table S11.** Toxicophoric Rules of compounds **8m** and **8d**

| **Property** | **8m** | **8d** | **Comment** |
| --- | --- | --- | --- |
| Acute Toxicity Rule | 0 | 0 | - 20 substructures;   acute toxicity during oral administration |
| Genotoxic Carcinogenicity Rule | 1 alerts | 1 alerts | - 117 substructures;   carcinogenicity or mutagenicity |
| NonGenotoxic Carcinogenicity Rule | 1 alerts | 2 alerts | - 23 substructures;   carcinogenicity through nongenotoxic mechanisms |
| Skin Sensitization Rule | 3 alerts | 3 alerts | - 155 substructures;   skin irritation |
| Aquatic Toxicity Rule | 2 alerts | 3 alerts | - 99 substructures;   toxicity to liquid(water) |
| NonBiodegradale Rule | 0 | 1 alerts | - 19 substructures;   non-biodegradable |
| SureChEMBL Rule | 0 | 0 | - 164 substructures;   MedChem unfriendly status |
| FAF-Drugs4 Rule | 3 alerts | 3 alerts | 154 toxic substructures from FAF-Drug4 |

Researcher : **Dr.Ali elshamsy**

email : [Ali.elshamsy@deraya.edu.eg](mailto:Ali.elshamsy@deraya.edu.eg) mob. 01019195283

Assay : Western Blot assay

Samples : 02 samples

Reference : ---

Date : 25-12-2025

Kit used : ---

Reader : ---

Solvent : DMSO

Assay samples : ---

**Lab Report**

| **s** | Compound | | | western blotting | **B-actin** |
| --- | --- | --- | --- | --- | --- |
|  | **code** | **cells** | **conc** | OD |  |
|  |  |  |  | **MCF7** |  |
|  |  |  |  | **Tub** |  |
| **I** | **8m** | **MCF7** | **---** | **2.62** | √ |
| **II** | **control** | **---** | **---** | **6.89** | √ |


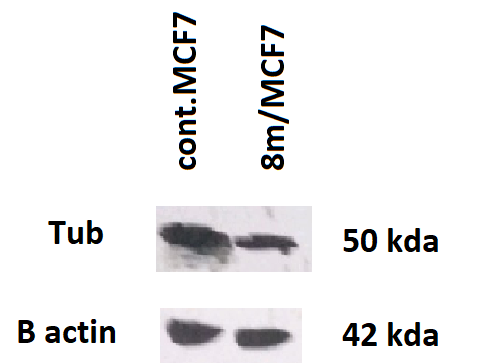


**Detailed Results:**

| **Tub-B** | 50 kda |  |  |  |
| --- | --- | --- | --- | --- |
| sample band | area | % | Rel.Density | adj. Density |
| Standared | 1739 | 17.39 | 1 | 1 |
| **8m/MCF7** | 2843 | 28.43 | 1.634847614 | 2.615756182 |
| **cont.MCF7** | 7492 | 74.92 | 4.308223117 | 6.893156987 |
|  |  |  |  |  |
|  |  |  |  |  |


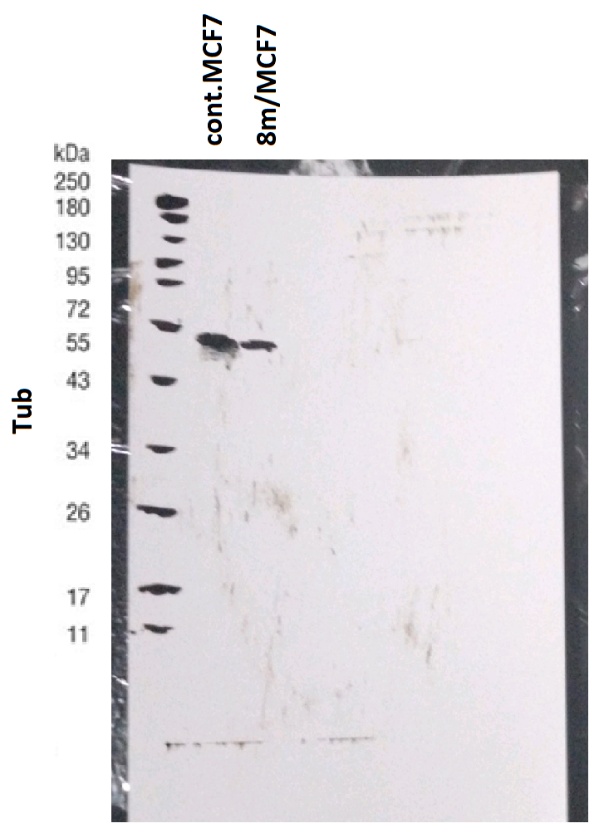


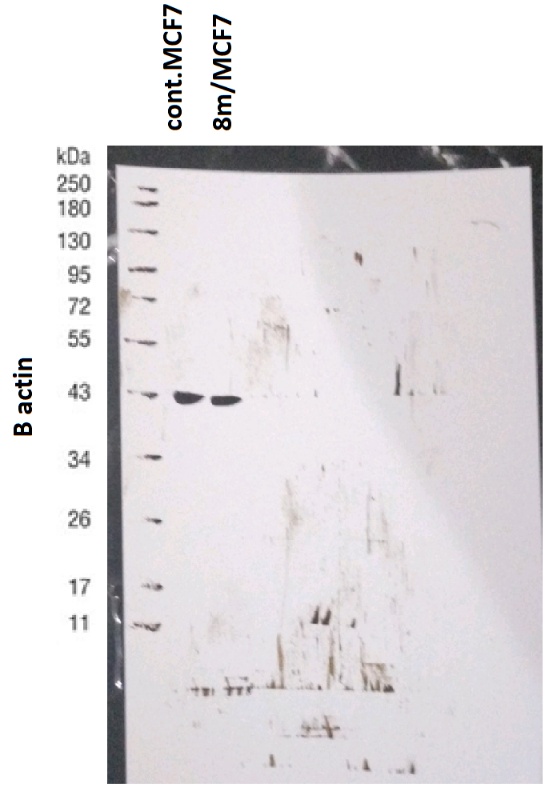


**Western Blot Protocol for Tubulin Analysis in MCF7 Cells**

**I. Materials and Device List**

**A. Equipment**

1. **Cell Culture & Lysis**
   - CO₂ incubator (37°C, 5% CO₂)
   - Biological safety cabinet
   - Centrifuge (refrigerated, capable of 12,000-14,000 × g)
   - Vortex mixer
   - Water bath (95-100°C)
   - NanoDrop or spectrophotometer
2. **Electrophoresis & Transfer**
   - Vertical electrophoresis system (e.g., Bio-Rad Mini-PROTEAN)
   - Power supply (300V, 400mA minimum)
   - Wet or semi-dry transfer system
   - Cooling system (optional for running)
3. **Detection**
   - Rocking platform shaker
   - Imaging system (chemiluminescence, e.g., Bio-Rad ChemiDoc)
   - Film processor or digital imager

**B. Reagents and Kits**

1. **Cell Culture**
   - MCF7 cell line (ATCC® HTB-22™)
   - DMEM high glucose medium
   - Fetal bovine serum (FBS)
   - Penicillin/streptomycin
   - Trypsin-EDTA
   - Test compounds and vehicle control (DMSO)
2. **Lysis Buffer**
   - Commercial RIPA buffer kit (e.g., **Thermo Fisher Scientific, Cat# 89900**) OR
   - Homemade RIPA: 50 mM Tris-HCl (pH 7.4), 150 mM NaCl, 1% NP-40, 0.5% sodium deoxycholate, 0.1% SDS, 1 mM EDTA + protease inhibitors
3. **Protein Quantification**
   - **BCA Protein Assay Kit** (Thermo Fisher Scientific, Cat# 23225)
4. **Electrophoresis**
   - **Precast gels**: 4-20% Tris-Glycine (Bio-Rad, Cat# 4561094) OR
   - Materials for handcast gels: Acrylamide/bis-acrylamide, Tris buffers, SDS, APS, TEMED
   - Running buffer (10X): 250 mM Tris, 1.92 M glycine, 1% SDS
   - Laemmli sample buffer (2X): 125 mM Tris-HCl (pH 6.8), 4% SDS, 20% glycerol, 0.01% bromophenol blue, 100 mM DTT (add fresh)
   - Prestained protein ladder (e.g., **Thermo Fisher Scientific, Cat# 26616**)
5. **Transfer**
   - Nitrocellulose (0.2 µm) or PVDF membrane (Bio-Rad, Cat# 1620112)
   - Transfer buffer: 25 mM Tris, 192 mM glycine, 20% methanol
   - Filter papers (Whatman)
6. **Antibodies**
   - **Primary antibodies**:
     - Anti-β-Tubulin Mouse monoclonal (Cell Signaling Technology, **Cat# 86298**), 1:1000
     - Anti-β-Actin Rabbit monoclonal (Cell Signaling Technology, **Cat# 4970**), 1:2000
   - **Secondary antibodies**:
     - Goat anti-Mouse IgG-HRP (Cell Signaling Technology, **Cat# 7076**), 1:2000
     - Goat anti-Rabbit IgG-HRP (Cell Signaling Technology, **Cat# 7074**), 1:2000
   - **Blocking buffer**: 5% non-fat dry milk in TBST
   - **TBS-T**: 20 mM Tris-HCl (pH 7.6), 150 mM NaCl, 0.1% Tween-20
7. **Detection**
   - Enhanced chemiluminescence (ECL) substrate (**SuperSignal West Pico PLUS**, Thermo Fisher Scientific, Cat# 34580)
   - Stripping buffer (optional): Mild stripping buffer (Thermo Fisher Scientific, Cat# 21059)

**II. Step-by-Step Protocol**

**Day 1: Cell Treatment and Lysis**

1. **Cell Culture**: Grow MCF7 cells to 70-80% confluency in 6-well plates (2×10⁵ cells/well).
2. **Treatment**:
   - Control: Vehicle (e.g., 0.1% DMSO)
   - Treated: Compound at desired concentrations (include triplicates)
   - Incubate for desired time (e.g., 24h) at 37°C, 5% CO₂
3. **Lysis**:
   - Place plate on ice, aspirate media, wash with ice-cold PBS
   - Add 100 µL RIPA + protease inhibitors per well
   - Scrape, transfer to pre-chilled microcentrifuge tubes
   - Incubate on ice 15 min, vortexing every 5 min
   - Centrifuge at 12,000 × g, 15 min, 4°C
   - Transfer supernatant to new tubes
4. **Protein Quantification**:
   - Use BCA assay per kit instructions
   - Dilute samples to equal concentrations with RIPA buffer
   - Prepare aliquots with 4X Laemmli buffer (3:1 sample:buffer)
   - Heat at 95°C for 5 min, snap-cool on ice
   - Store at -80°C or proceed immediately

**Day 2: SDS-PAGE and Transfer**

1. **Gel Setup**:
   - Assemble gel apparatus, fill with 1X running buffer
   - Load 20-30 µg protein per well alongside ladder
2. **Electrophoresis**:
   - Run at 80V through stacking gel, then 120V through resolving gel (~90 min)
   - Stop when dye front reaches bottom
3. **Transfer**:
   - Activate PVDF membrane in methanol (15 sec) if using
   - Assemble transfer stack: cathode-sponge-filter paper-gel-membrane-filter paper-sponge-anode
   - Transfer at 100V for 60 min (wet) or per semi-dry system recommendations (25V, 30 min)
   - Confirm transfer with Ponceau S staining (optional)

**Day 2-3: Immunoblotting**

1. **Blocking**: Incubate membrane in 5% milk/TBST, 1h, RT with gentle shaking
2. **Primary Antibody Incubation**:
   - Prepare primary antibodies in 5% milk/TBST
   - Incubate membrane with **anti-β-Tubulin** overnight at 4°C with shaking
   - Wash: 3 × 5 min with TBST
3. **Secondary Antibody Incubation**:
   - Incubate with anti-mouse HRP, 1h, RT
   - Wash: 3 × 5 min with TBST
4. **Detection**:
   - Mix ECL reagents per instructions
   - Incubate membrane 1 min, drain excess
   - Image using chemiluminescence settings (multiple exposures)

**Membrane Stripping and Re-probing for β-Actin**

1. **Stripping**:
   - Incubate membrane in mild stripping buffer, 15 min, RT
   - Wash: 3 × 5 min with TBST
2. **Re-block**: 5% milk/TBST, 30 min
3. **Repeat immunoblotting** with anti-β-Actin (1:2000) and anti-rabbit HRP

**III. Data Analysis**

1. **Image Analysis**:
   - Use ImageLab (Bio-Rad) or ImageJ software
   - Measure band intensity for tubulin and β-actin
2. **Normalization**:
   - Calculate ratio: Tubulin intensity / β-actin intensity for each sample
   - Normalize treated ratios to control (set control = 1)
3. **Statistics**:
   - Perform t-test or ANOVA with post-hoc test (n≥3)
   - Express as mean ± SEM

**IV. Critical Notes & Troubleshooting**

1. **Sample Preparation**:
   - Keep samples cold to prevent degradation
   - Ensure equal loading across lanes
2. **Antibody Optimization**:
   - Titrate antibodies for optimal signal-to-noise
   - Validate linear range for quantification
3. **Controls**:
   - Include loading control (β-actin) on same membrane
   - Verify compound doesn't affect β-actin expression
4. **Troubleshooting**:
   - **High background**: Increase wash time, optimize antibody concentration
   - **No signal**: Check antibody compatibility, ensure HRP activity
   - **Non-specific bands**: Optimize blocking conditions

**V. Expected Results**

- β-Tubulin migrates at ~50 kDa
- β-Actin migrates at ~42 kDa
- Clear bands with minimal background
- Quantitative differences between treated and control samples

**Researcher** : **Dr.Ali elshamsy**

email : [Ali.elshamsy@deraya.edu.eg](mailto:Ali.elshamsy@deraya.edu.eg) mob. 01019195283

Date : 25-12-2025

Assay : Cell Cycle Analysis

Samples : 02 samples

cell line : ---

Ref. : ---

Reader : BD FACSCalibur

Kit used : ab139418_PI Flow Cytometry Kit/BD

Solvent : DMSO

Assay samples : Cell culture

**Lab Report**

| **ser** | **Sample** | | **DNA content** | | | |  |
| --- | --- | --- | --- | --- | --- | --- | --- |
|  | **code** | **IC50**  **ug** | **%G0-G1** | **%S** | **%G2/M** | **Comment** |  |
| 1 | **8m/MCF7** | --- | **25.47** | **29.18** | **45.35** | Cell cycle arrest@G2/M |  |
| 2 | **cont. MCF7** | --- | **49.82** | **36.44** | **13.74** | --- |  |


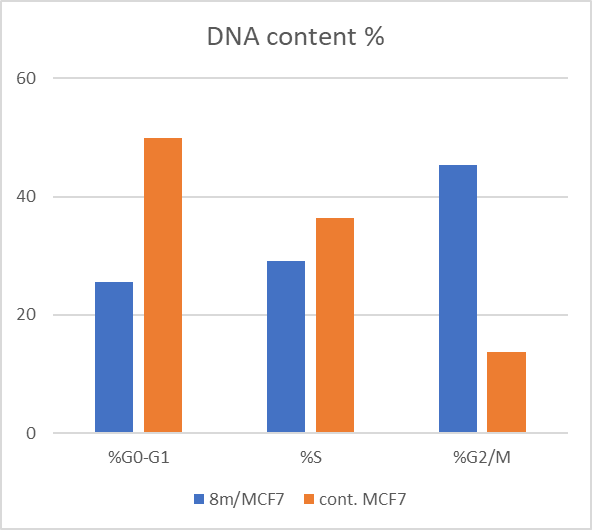


Apoptosis/necrosis :

| **s** | **code** | **conc** | **Apoptosis** | | | **Necrosis** |
| --- | --- | --- | --- | --- | --- | --- |
|  |  |  | Total | Early | Late |  |
| 1 | **8m/MCF7** | --- | **27.13** | **4.69** | **17.42** | **5.02** |
| 2 | **cont. MCF7** | --- | **3.29** | **0.67** | **0.29** | **2.33** |


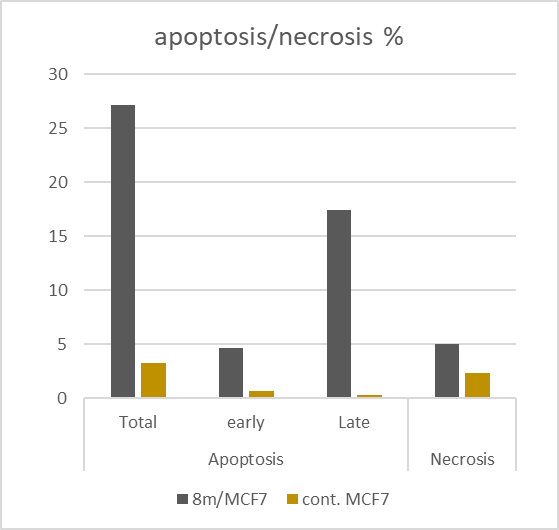


Detailed results :


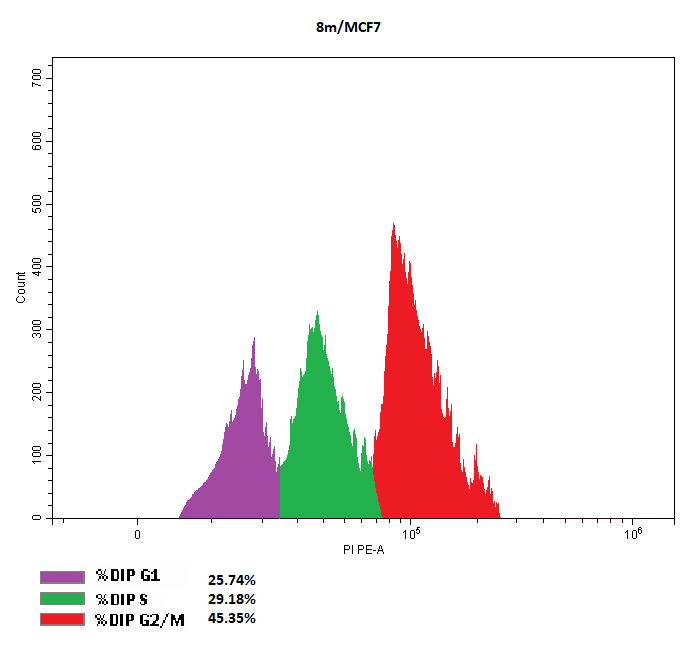


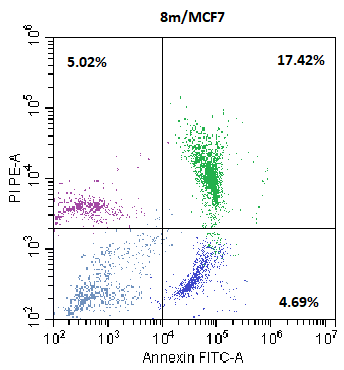


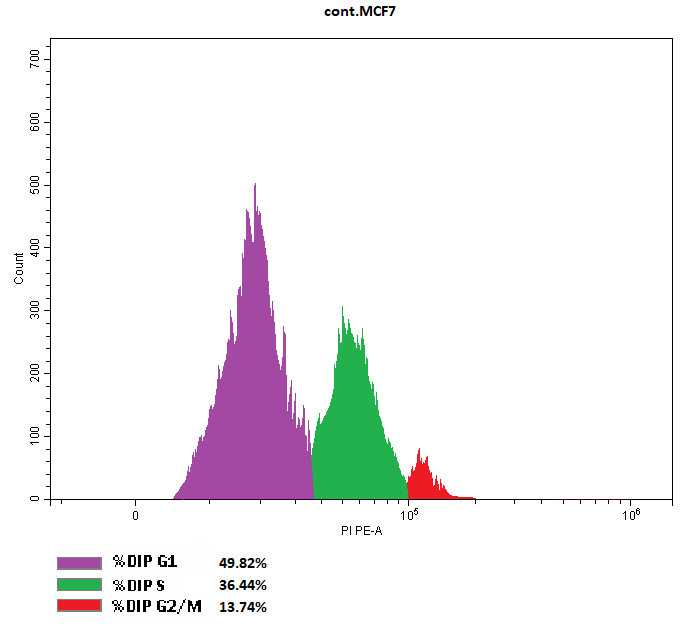


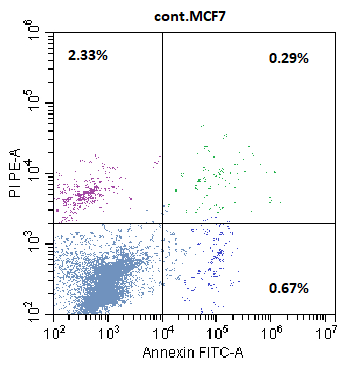


**Tubulin Polymerization assay**

**Researcher** : **Dr.Ali elshamsy**

email : [Ali.elshamsy@deraya.edu.eg](mailto:Ali.elshamsy@deraya.edu.eg) mob. 01019195283

Date : 25-12-2025

**Assay** : Tubulin **polymerization** assay

**Samples** : 05 compounds

**Ref**. : *--*

**Reader** : Tecan –spark READER wl Ex. 360 nm and Em. 450 nm.

**Kit used** : ---

**Solvent** : DMSO

**Assay samples** : --

**Lab Report**

| **ser** | **Compound** | | | **Tubulin polymerization inh** | **SD** |
| --- | --- | --- | --- | --- | --- |
|  | **ID** | **M.W**  **g/mol** | **conc** | **IC50**  **ug/ml** |  |
| 1 | **8b** | --- | --- | **7.19±**0.24 |  |
| 2 | **8c** | --- | --- | **16.54±**0.56 |  |
| 3 | **8d** | --- | --- | **4.105±**0.14 |  |
| 4 | **8m** | --- | --- | **3.862±**0.13 |  |
| 5 | **8o** | --- | --- | **12.81±**0.43 |  |
| *** | **Combretastatin A4** | --- | --- | **2.408±**0.08 |  |

Detailed results :

| **Tub** |  |  |  |  |  |  |  |  |  |  |  |  |  |  |  |  |  |
| --- | --- | --- | --- | --- | --- | --- | --- | --- | --- | --- | --- | --- | --- | --- | --- | --- | --- |
| code | IC50 | conc | log | %inh | T2 | T1 | ∆T | RFU2 | RFU1 | ∆RFU | slope | K.Activity | EC |  | RFU1 | RFU2 | RFU3 |
| 8b |  | 100 | 2 | 94.28 | 30 | 0 | 30 | 2444 | 0 | 2444.342 | 1423.9 | 6.866613 | 120 |  | 2447 | 2412 | 2474 |
| \|  \| \| --- \| |  | 25 | 1.398 | 78.9 | 30 | 0 | 30 | 9013 | 0 | 9013.201 | 1423.9 | 25.31976 | 120 |  | 9023 | 8896 | 9121 |
|  |  | 12.5 | 1.097 | 49.53 | 30 | 0 | 30 | 21558 | 0 | 21557.56 | 1423.9 | 60.5592 | 120 |  | 21581 | 21276 | 21815 |
|  |  | 3.13 | 0.496 | 29.95 | 30 | 0 | 30 | 29924 | 0 | 29924.47 | 1423.9 | 84.06339 | 120 |  | 29957 | 29534 | 30282 |
|  |  | 0.7 | -0.155 | 18.09 | 30 | 0 | 30 | 34991 | 0 | 34990.96 | 1423.9 | 98.29611 | 120 |  | 35029 | 34534 | 35410 |
|  |  |  |  | 0 | 30 | 0 | 30 | 42717 | 0 | 42716.56 | 1423.9 | 120 | 120 |  | 42763 | 42159 | 43228 |
|  |  |  |  |  |  |  |  |  |  |  |  |  |  |  |  |  |  |
| code | IC50 | conc | log | %inh | T2 | T1 | ∆T | RFU2 | RFU1 | ∆RFU | slope | K.Activity | EC |  |  |  |  |
| \| 8c \| \| --- \| |  | 100 | 2 | 88.33 | 30 | 0 | 30 | 4987 | 0 | 4986.578 | 1423.9 | 14.00823 | 120 |  | 4992 | 4922 | 5046 |
|  |  | 25 | 1.398 | 64.78 | 30 | 0 | 30 | 15046 | 0 | 15045.64 | 1423.9 | 42.26601 | 120 |  | 15062 | 14849 | 15226 |
|  |  | 12.5 | 1.097 | 31.16 | 30 | 0 | 30 | 29405 | 0 | 29405.03 | 1423.9 | 82.6042 | 120 |  | 29437 | 29021 | 29757 |
|  |  | 3.13 | 0.496 | 10.67 | 30 | 0 | 30 | 38161 | 0 | 38160.51 | 1423.9 | 107.2 | 120 |  | 38202 | 37662 | 38617 |
|  |  | 0.7 | -0.155 | 2.143 | 30 | 0 | 30 | 41802 | 0 | 41801.55 | 1423.9 | 117.4283 | 120 |  | 41847 | 41256 | 42302 |
| EC |  |  |  | 0 | 30 | 0 | 30 | 42717 | 0 | 42716.56 | 1423.9 | 120 | 120 |  | 42763 | 42159 | 43228 |
|  |  |  |  |  |  |  |  |  |  |  |  |  |  |  |  |  |  |
| code | IC50 | conc | log | %inh | T2 | T1 | ∆T | RFU2 | RFU1 | ∆RFU | slope | K.Activity | EC |  |  |  |  |
| 8o |  | 100 | 2 | 87.09 | 30 | 0 | 30 | 5513 | 0 | 5513.006 | 1423.9 | 15.48706 | 120 |  | 5519 | 5441 | 5579 |
| \|  \| \| --- \| |  | 25 | 1.398 | 68.61 | 30 | 0 | 30 | 13407 | 0 | 13407.42 | 1423.9 | 37.66395 | 120 |  | 13422 | 13232 | 13568 |
|  |  | 12.5 | 1.097 | 41.57 | 30 | 0 | 30 | 24960 | 0 | 24959.86 | 1423.9 | 70.1169 | 120 |  | 24987 | 24634 | 25258 |
|  |  | 3.13 | 0.496 | 18.99 | 30 | 0 | 30 | 34604 | 0 | 34604.38 | 1423.9 | 97.21013 | 120 |  | 34642 | 34153 | 35018 |
|  |  | 0.7 | -0.155 | 5.805 | 30 | 0 | 30 | 40237 | 0 | 40237.25 | 1423.9 | 113.0339 | 120 |  | 40281 | 39712 | 40719 |
|  |  |  |  | 0 | 30 | 0 | 30 | 42717 | 0 | 42716.56 | 1423.9 | 120 | 120 |  | 42763 | 42159 | 43228 |
|  |  |  |  |  |  |  |  |  |  |  |  |  |  |  |  |  |  |
| code | IC50 | conc | log | %inh | T2 | T1 | ∆T | RFU2 | RFU1 | ∆RFU | slope | K.Activity | EC |  |  |  |  |
| \| 8m \| \| --- \| |  | 100 | 2 | 95.59 | 30 | 0 | 30 | 1882 | 0 | 1881.954 | 1423.9 | 5.286759 | 120 |  | 1884 | 1857 | 1904 |
|  |  | 25 | 1.398 | 88.91 | 30 | 0 | 30 | 4736 | 0 | 4735.851 | 1423.9 | 13.30389 | 120 |  | 4741 | 4674 | 4793 |
|  |  | 12.5 | 1.097 | 73.07 | 30 | 0 | 30 | 11503 | 0 | 11503.49 | 1423.9 | 32.31545 | 120 |  | 11516 | 11353 | 11641 |
|  |  | 3.13 | 0.496 | 43.2 | 30 | 0 | 30 | 24265 | 0 | 24264.62 | 1423.9 | 68.16383 | 120 |  | 24291 | 23948 | 24555 |
|  |  | 0.7 | -0.155 | 20.53 | 30 | 0 | 30 | 33948 | 0 | 33948.09 | 1423.9 | 95.3665 | 120 |  | 33985 | 33505 | 34354 |
|  |  |  |  | 0 | 30 | 0 | 30 | 42717 | 0 | 42716.56 | 1423.9 | 120 | 120 |  | 42763 | 42159 | 43228 |
|  |  |  |  |  |  |  |  |  |  |  |  |  |  |  |  |  |  |
| code | IC50 | conc | log | %inh | T2 | T1 | ∆T | RFU2 | RFU1 | ∆RFU | slope | K.Activity | EC |  |  |  |  |
| \| 8d \| \| --- \| |  | 100 | 2 | 94.89 | 30 | 0 | 30 | 2184 | 0 | 2183.626 | 1423.9 | 6.134211 | 120 |  | 2186 | 2155 | 2210 |
|  |  | 25 | 1.398 | 85.35 | 30 | 0 | 30 | 6258 | 0 | 6258.196 | 1423.9 | 17.58044 | 120 |  | 6265 | 6177 | 6333 |
|  |  | 12.5 | 1.097 | 67.13 | 30 | 0 | 30 | 14042 | 0 | 14041.73 | 1423.9 | 39.44584 | 120 |  | 14057 | 13858 | 14210 |
|  |  | 3.13 | 0.496 | 46.25 | 30 | 0 | 30 | 22960 | 0 | 22960.04 | 1423.9 | 64.49902 | 120 |  | 22985 | 22660 | 23235 |
|  |  | 0.7 | -0.155 | 20.13 | 30 | 0 | 30 | 34120 | 0 | 34119.9 | 1423.9 | 95.84916 | 120 |  | 34157 | 33675 | 34528 |
|  |  |  |  | 0 | 30 | 0 | 30 | 42717 | 0 | 42716.56 | 1423.9 | 120 | 120 |  | 42763 | 42159 | 43228 |
|  |  |  |  |  |  |  |  |  |  |  |  |  |  |  |  |  |  |
| code | IC50 | conc | log | %inh | T2 | T1 | ∆T | RFU2 | RFU1 | ∆RFU | slope | K.Activity | EC |  |  |  |  |
| CA4 |  | 100 | 2 | 95.9 | 30 | 0 | 30 | 1750 | 0 | 1750.097 | 1423.9 | 4.916349 | 120 |  | 1752 | 1727 | 1771 |
|  |  | 25 | 1.398 | 90.76 | 30 | 0 | 30 | 3948 | 0 | 3947.708 | 1423.9 | 11.08985 | 120 |  | 3952 | 3896 | 3995 |
| \|  \| \| --- \| |  | 12.5 | 1.097 | 78.85 | 30 | 0 | 30 | 9034 | 0 | 9034.178 | 1423.9 | 25.37869 | 120 |  | 9044 | 8916 | 9142 |
|  |  | 3.13 | 0.496 | 58.72 | 30 | 0 | 30 | 17634 | 0 | 17633.83 | 1423.9 | 49.5367 | 120 |  | 17653 | 17404 | 17845 |
|  |  | 0.7 | -0.155 | 24.72 | 30 | 0 | 30 | 32158 | 0 | 32158.04 | 1423.9 | 90.33791 | 120 |  | 32193 | 31738 | 32543 |
| EC |  |  |  | 0 | 30 | 0 | 30 | 42717 | 0 | 42716.56 | 1423.9 | 120 | 120 |  | 42763 | 42159 | 43228 |
|  |  |  |  |  |  |  |  |  |  |  |  |  |  |  |  |  |  |
